# Supplementary material for: Fear of grazing rivals the toxin‐inducing effects of nutrients in two marine harmful algae – a meta‐analysis
Source: Biol Rev Camb Philos Soc. 2026 Mar 8;101(4):1904–21. doi: 10.1002/brv.70153 (PMC13326770; doi:10.1002/brv.70153)
Supplement: Supplementary file 2 — Code S1. All code used to produce the analyses and figures in this study, including the resulting output. [file BRV-101-1904-s001.html]

Supplementary code


# Supplementary code

### R code for statistical analysis and data visualisation

#### Milad Pourdanandeh

#### 2026-02-12

## **Purpose**

This is the analysis code used to perform all analyses and construct
the figures for the the manuscript “**Fear of grazing rivals the
toxin-inducing effects of nutrients in two marine harmful algae – a
meta-analysis**” by Pourdanandeh and Selander.

### **Datasets**

- “data\_MA\_rev.csv” contains all data used to perform analyses and
  visualise the results from the meta-analyses.
- “data\_prisma.csv” contains the data used visualise the bar chart of
  all papers fully screened in the process of identifying relevant and
  eligible papers to include in our synthesis.

# **Availability**

All the data here will be available at https://doi.org/10.5281/zenodo.14713104

## 1. Load packages

```
if(!require(pacman)){install.packages("pacman")}

# Load/install github hosted packages
pacman::p_load_gh("daniel1noble/orchaRd",
                  "NightingaleHealth/ggforestplot")


# Load/install CRAN hosted packages
pacman::p_load(tidyverse,
               readr, 
               devtools, 
               glmulti,
               patchwork,
               multcomp,
               emmeans,
               metafor,
               ggtext,
               ggridges)

#write_bib(.packages(), "packages.bib")
```

## 2. Import, modufy & clean data

```
setwd("C:/Users/xpoumi/OneDrive/1. PhD GU/1. Research/Meta-analysis - Demand vs. Resource driven toxin production/Analysis R")

data_MA_full <- read_delim("data_MA_rev.csv", 
                            delim = ";", 
                            escape_double = FALSE, 
                            locale = locale(), 
                            trim_ws = TRUE)


## Capitalise Calanoida
data_MA_full$Zooplankton_order[data_MA_full$Zooplankton_order == "calanoida"] <- "Calanoida"

data_MA_full <- data_MA_full %>%  filter(max_effect == "yes")

## Encode some of the variables as factors
### Re-encode all character columns as factor columns
data_MA_full <-  data_MA_full%>% 
  mutate_if(is.character, as.factor)

## Rename column "Strain" to " Phytoplankton_strain
data_MA_full <- rename(data_MA_full,
                  Phytoplankton_strain = Strain)
```

## 3. Calculate effect sizes and their variance

### 3.1 Calculation of the standard Log Response-Ratio (LRR) using `ecalc`

```
data_MA_full <- escalc(measure = "ROM", 
                  m2i = mean_tox_control, sd2i = sd_control, n2i = n_control,
                  m1i = mean_tox_ex, sd1i = sd_ex, n1i = n_ex,
                  data = data_MA_full)

### Rename logRR columns for ease of use
data_MA_full <- rename(data_MA_full, 
                  RR = yi,
                  Var_RR = vi)
```

### 3.2 Functions to calculate the small-sample bias corrected log response ratio (LRR\(^\Delta\)) proposed by Lajeunesse (2015)

```
attach(data_MA_full)

## Effect size function
delta_LRR <- function(mean_treatment, mean_control, sd_treatment, sd_control, n_treatment, n_control) {
  result <- log(mean_treatment / mean_control) + 0.5 * (
    (sd_treatment^2 / (n_treatment * mean_treatment^2)) - (sd_control^2 / (n_control * mean_control^2))
  )
  return(result)
}

## Effect size error function

Var_delta_LRR <- function(mean_treatment, mean_control, sd_treatment, sd_control, n_treatment, n_control) {
  result <- (sd_treatment^2 / (n_treatment * mean_treatment^2) + sd_control^2 / (n_control * mean_control^2) +
    0.5 * (sd_treatment^4 / (n_treatment^2 * mean_treatment^4) + sd_control^4 / (n_control^2 * mean_control^4)))
  return(result)
}
```

### 3.3 Calculation of small-sample bias corrected log response ratio using the function created above

```
## Effect size
data_MA_full$delta_RR <- delta_LRR(mean_tox_ex, mean_tox_control, sd_ex, sd_control, n_ex, n_control)

## Effext size error
data_MA_full$Var_delta_RR <- Var_delta_LRR(mean_tox_ex, mean_tox_control, sd_ex, sd_control, n_ex, n_control)
```

### 3.4 Calculation of small sample bias corrected Hedges’ *d* (Hedges, 1981) using `escalc`

```
## Calculate bias corrected Hedges' d using escalc
data_MA_full <- escalc(measure = "SMD", 
                  m2i = mean_tox_control, sd2i = sd_control, n2i = n_control,
                  m1i = mean_tox_ex, sd1i = sd_ex, n1i = n_ex,
                  data = data_MA_full)

### Rename Hedges'd columns for ease of use
data_MA_full <- rename(data_MA_full, 
                  Hedges_d = yi,
                  Var_Hedges_d = vi)
```

### 3.5 Data subsets

```
# Create new subsets of data----------------------------------------------------
## Passed modified Geary's rule (>3)
data_MA_geary <- data_MA_full %>%  filter(Pass_Geary == 1)

## Only Alexandrium 
data_Alex_full <- data_MA_full %>%  filter(Phytoplankton_group == "Alexandrium")

## Only Alexandrium that passed Geary
data_Alex_Geary <- data_Alex_full %>%  filter(Pass_Geary == 1)

## Only Pseudo-nitzschia
data_Pseudo_full <- data_MA_full  %>%   filter(Phytoplankton_group == "Pseudo-nitzschia")

## Only Pseudo-nitzschia that passed Geary
data_Pseudo_Geary <- data_Pseudo_full %>%  filter(Pass_Geary == 1)
```

## 4. Initial meta-analyses

### 4.1 Random effect of case (each effect) nested within studies

```
rand <- list(~ 1 | Article_ID/Effect_ID)
```

### 4.2 Random effects meta-analyses

#### 4.2.1 Full dataset, including cases that violate Geary’s rule.

```
Full_REMA_reml <- rma.mv(yi = delta_RR, V = Var_delta_RR,
                      data = data_MA_full,
                      random = rand,
                      test = "t",
                      method = "REML")

summary.rma(Full_REMA_reml)
```

```
## 
## Multivariate Meta-Analysis Model (k = 113; method: REML)
## 
##    logLik   Deviance        AIC        BIC       AICc   
## -179.1953   358.3906   364.3906   372.5461   364.6128   
## 
## Variance Components:
## 
##             estim    sqrt  nlvls  fixed                factor 
## sigma^2.1  0.4227  0.6502     37     no            Article_ID 
## sigma^2.2  1.0908  1.0444    113     no  Article_ID/Effect_ID 
## 
## Test for Heterogeneity:
## Q(df = 112) = 18824.5558, p-val < .0001
## 
## Model Results:
## 
## estimate      se    tval   df    pval   ci.lb   ci.ub      
##   1.4513  0.1549  9.3676  112  <.0001  1.1443  1.7582  *** 
## 
## ---
## Signif. codes:  0 '***' 0.001 '**' 0.01 '*' 0.05 '.' 0.1 ' ' 1
```

```
i2_ml(Full_REMA_reml)
```

```
##                I2_Total           I2_Article_ID I2_Article_ID/Effect_ID 
##                99.46153                27.77906                71.68246
```

```
r2_ml(Full_REMA_reml)
```

```
##    R2_marginal R2_conditional 
##      0.0000000      0.2792946
```

```
## Orchard plot
orchard_plot(Full_REMA_reml,
             data = data_MA_full,
             group = "Article_ID", # Colour of the bubbles in the plot & number in parentheses.
             xlab = "lnRR (effect size)",
             angle = 0,
             alpha = 0.4,
             cb = FALSE,
             k = TRUE,
             g = TRUE,
             trunk.size = 8,
             branch.size = 2,
             twig.size = 0.5,
             colour = TRUE,
             fill = TRUE,
             legend.pos = "bottom.right"
             ) +
  
  scale_y_continuous(n.breaks = 10)
```

#### 4.2.2 Cases that violate Geary’s rule removed

```
### Excluding failed Geary
Full_REMA_reml_geary <- rma.mv(yi = delta_RR, V = Var_delta_RR, 
                            random = rand,
                            data = data_MA_geary,
                            test = "t",
                            method = "REML")

summary.rma(Full_REMA_reml_geary)
```

```
## 
## Multivariate Meta-Analysis Model (k = 101; method: REML)
## 
##    logLik   Deviance        AIC        BIC       AICc   
## -155.5170   311.0339   317.0339   324.8495   317.2839   
## 
## Variance Components:
## 
##             estim    sqrt  nlvls  fixed                factor 
## sigma^2.1  0.4934  0.7024     37     no            Article_ID 
## sigma^2.2  0.9538  0.9766    101     no  Article_ID/Effect_ID 
## 
## Test for Heterogeneity:
## Q(df = 100) = 18573.2247, p-val < .0001
## 
## Model Results:
## 
## estimate      se    tval   df    pval   ci.lb   ci.ub      
##   1.3630  0.1609  8.4731  100  <.0001  1.0438  1.6821  *** 
## 
## ---
## Signif. codes:  0 '***' 0.001 '**' 0.01 '*' 0.05 '.' 0.1 ' ' 1
```

```
i2_ml(Full_REMA_reml_geary)
```

```
##                I2_Total           I2_Article_ID I2_Article_ID/Effect_ID 
##                99.48904                33.91690                65.57215
```

```
r2_ml(Full_REMA_reml_geary)
```

```
##    R2_marginal R2_conditional 
##      0.0000000      0.3409109
```

```
## Orchard plot
orchard_plot(Full_REMA_reml_geary,
             data = data_MA_geary,
             group = "Article_ID", # Colour of the bubbles in the plot & number in parentheses.
             xlab = "lnRR (effect size)",
             angle = 0,
             alpha = 0.4,
             cb = FALSE,
             k = TRUE,
             g = TRUE,
             trunk.size = 8,
             branch.size = 2,
             twig.size = 0.5,
             colour = TRUE,
             fill = TRUE,
             legend.pos = "bottom.right"
             ) +
  
  scale_y_continuous(n.breaks = 10)
```

Excluding the 12 effects that violate Geary’s rule decreases the
grand mean effect with ~35 percentage points but does not differ from
the full dataset. 27.7% of the total variance is estimated to be due to
**between-cluster** (study/paper/Article\_ID) heterogeneity
and 71.7% due to **within-cluster** heterogeneity. Sampling
variance account for ~0.5% of the total variance.

## 5. Fitting multilevel mixed effects models

These models encode effects within moderators as random effects and
between moderators as fixed effects, while estimating and using the
heteroscedastic variances of moderator levels, i.e., the models do not
assume that all levels of moderators have equal variances (which is what
standard random effects models do, but this is almost never the case in
ecology). The model code has been adapted from the `orchaRd`
(Nakagawa *et al.*,
2021) vignette by Nakagawa and colleagues (2023).

### 5.1 Assessing underlying structures/patterns - moderator analyses

We primarily use moderators that are relevant for both experiment
types, in practice this means variables that contain less than 20% NAs
and are likely to contain differing levels. Here, we are mainly
interested in investigating and describing any structures hidden in the
data that complicates the general cunclusions we want to draw down the
line. We will also check to see if there is an effect of zooplankton
taxa on toxin induction, this will only include effects from
“Demand/Top-down” experiments. Also, we ignore the experiment type
(`Exp_type`) moderator for now, because it will be the main
moderator used in the final analysis regardless. Because
`metafor` automatically removes cases that has NAs in the
moderator used, we do th same. When the specified optimizer and method
cannot reach convergence they are omitted and defaults used instead, the
difference in results are negligible (~0.01%) for the moderators we have
tested (e.g., Phytoplankton\_group)

#### 5.1.1 Variables containing less than 20% NAs

```
NA_list <-
colSums(is.na(data_MA_full)) %>% 
  as.data.frame() %>%  
  dplyr::mutate(NAs = colSums(is.na(data_MA_full))) %>%  
  dplyr::select(NAs) %>%   
  dplyr::filter(NAs < 113*0.2)

NA_list
```

```
##                       NAs
## Pass_Geary              0
## Reviewer                0
## Article_ID              0
## Effect_ID               0
## ID_1                    0
## ID_2                    0
## Year                    0
## Exp_type                0
## Ecol_relevant           0
## max_effect              0
## Group                   0
## Phytoplankton_species   0
## Phytoplankton_strain    1
## Phytoplankton_group     0
## Toxin                   0
## Analogues               0
## Culture_type            0
## Experiment_medium       0
## Starting_cell_conc      6
## Light                  13
## LD_cycle                3
## Temperature             0
## Hours                   9
## added_.N_source         0
## added_P_source          0
## tot_N_conc_control      1
## tot_P_conc_control      0
## N_conc_ex               0
## P_conc_ex               0
## Nutrient_conc_unit      0
## NP_ratio_control        1
## NP_ratio_ex             2
## NP_ratio_diff           3
## NPR_ratio               3
## AbsDiff_LnNP            3
## n_control               0
## n_ex                    0
## Tot_N                   0
## mean_tox_control        0
## mean_tox_ex             0
## sd_control              0
## sd_ex                   0
## calc_se_control         0
## calc_se_ex              0
## unit_tox                0
## modGeary_control        0
## modGeary_ex             0
## Pseudo_nitzschia        1
## Microcystis             1
## Alexandrium             1
## Nutrient                1
## No_grazing              1
## Indirect_grazing        1
## direct_grazing          1
## Info_chemical           1
## Zooplankton             1
## Simultaneous_ig_dg      1
## RR                      0
## Var_RR                  0
## delta_RR                0
## Var_delta_RR            0
## Hedges_d                0
## Var_Hedges_d            0
```

#### 5.1.2 Phytoplankton species

```
mod_1 <- rma.mv(yi = delta_RR,
                V = Var_delta_RR, 
                mods = ~ 1 + Phytoplankton_species, 
                method = "REML", 
                test = "t", 
                random = list(~1 | Article_ID, 
                              ~1 + Phytoplankton_species| Effect_ID),
                rho = 0, 
                struc = "HCS", 
                data = data_MA_full, 
                #control = list(optimizer="optim", optmethod="Nelder-Mead")
                )

summary.rma(mod_1)
```

```
## 
## Multivariate Meta-Analysis Model (k = 113; method: REML)
## 
##    logLik   Deviance        AIC        BIC       AICc   
## -136.9424   273.8848   311.8848   362.1283   320.9324   
## 
## Variance Components:
## 
##             estim    sqrt  nlvls  fixed      factor 
## sigma^2    0.0000  0.0001     37     no  Article_ID 
## 
## outer factor: Effect_ID             (nlvls = 113)
## inner factor: Phytoplankton_species (nlvls = 9)
## 
##             estim    sqrt  k.lvl  fixed                         level 
## tau^2.1    0.9150  0.9565     12     no         Alexandrium catenella 
## tau^2.2    0.0840  0.2899      9     no         Alexandrium fundyense 
## tau^2.3    1.0481  1.0238     37     no           Alexandrium minutum 
## tau^2.4    0.2225  0.4717     17     no         Alexandrium tamarense 
## tau^2.5    4.3598  2.0880      6     no    Pseudo-nitzschia australis 
## tau^2.6    0.9883  0.9941      5     no  Pseudo-nitzschia fraudulenta 
## tau^2.7    3.8508  1.9623      2     no       Pseudo-nitzschia obtusa 
## tau^2.8    0.2605  0.5104      5     no      Pseudo-nitzschia pungens 
## tau^2.9    1.5846  1.2588     20     no      Pseudo-nitzschia seriata 
## rho        0.0000                   yes                               
## 
## Test for Residual Heterogeneity:
## QE(df = 104) = 6677.1777, p-val < .0001
## 
## Test of Moderators (coefficients 2:9):
## F(df1 = 8, df2 = 104) = 9.5588, p-val < .0001
## 
## Model Results:
## 
##                                                    estimate      se     tval 
## intrcpt                                              1.2438  0.2781   4.4733 
## Phytoplankton_speciesAlexandrium fundyense          -0.8280  0.2990  -2.7691 
## Phytoplankton_speciesAlexandrium minutum             0.2176  0.3266   0.6662 
## Phytoplankton_speciesAlexandrium tamarense          -0.5547  0.3028  -1.8316 
## Phytoplankton_speciesPseudo-nitzschia australis      1.2554  0.9023   1.3913 
## Phytoplankton_speciesPseudo-nitzschia fraudulenta    0.4235  0.5568   0.7606 
## Phytoplankton_speciesPseudo-nitzschia obtusa        -0.7655  1.4287  -0.5358 
## Phytoplankton_speciesPseudo-nitzschia pungens        0.0589  0.3978   0.1480 
## Phytoplankton_speciesPseudo-nitzschia seriata        1.3210  0.3997   3.3052 
##                                                     df    pval    ci.lb 
## intrcpt                                            104  <.0001   0.6924 
## Phytoplankton_speciesAlexandrium fundyense         104  0.0067  -1.4209 
## Phytoplankton_speciesAlexandrium minutum           104  0.5068  -0.4301 
## Phytoplankton_speciesAlexandrium tamarense         104  0.0699  -1.1552 
## Phytoplankton_speciesPseudo-nitzschia australis    104  0.1671  -0.5339 
## Phytoplankton_speciesPseudo-nitzschia fraudulenta  104  0.4486  -0.6807 
## Phytoplankton_speciesPseudo-nitzschia obtusa       104  0.5932  -3.5988 
## Phytoplankton_speciesPseudo-nitzschia pungens      104  0.8826  -0.7299 
## Phytoplankton_speciesPseudo-nitzschia seriata      104  0.0013   0.5284 
##                                                      ci.ub      
## intrcpt                                             1.7952  *** 
## Phytoplankton_speciesAlexandrium fundyense         -0.2350   ** 
## Phytoplankton_speciesAlexandrium minutum            0.8653      
## Phytoplankton_speciesAlexandrium tamarense          0.0459    . 
## Phytoplankton_speciesPseudo-nitzschia australis     3.0447      
## Phytoplankton_speciesPseudo-nitzschia fraudulenta   1.5278      
## Phytoplankton_speciesPseudo-nitzschia obtusa        2.0677      
## Phytoplankton_speciesPseudo-nitzschia pungens       0.8477      
## Phytoplankton_speciesPseudo-nitzschia seriata       2.1136   ** 
## 
## ---
## Signif. codes:  0 '***' 0.001 '**' 0.01 '*' 0.05 '.' 0.1 ' ' 1
```

```
i2_ml(mod_1)
```

```
##      I2_Total I2_Article_ID 
##  4.452816e-05  4.452816e-05
```

```
100 - i2_ml(mod_1)[1] # Sampling variance as proportion of total unaccounted variance
```

```
## I2_Total 
## 99.99996
```

```
r2_ml(mod_1)
```

```
##    R2_marginal R2_conditional 
##              1              1
```

```
## Orchard plot
orchard_plot(mod_1,
             data = data_MA_full,
             mod = "Phytoplankton_species",
             group = "Article_ID", # Colour of the bubbles in the plot & number in parentheses.
             xlab = bquote(LRR^~Delta),
             angle = 0,
             alpha = 0.4,
             cb = FALSE,
             k = TRUE,
             g = TRUE,
             trunk.size = 8,
             branch.size = 1.5,
             twig.size = 0.5,
             colour = TRUE,
             fill = TRUE,
             legend.pos = "bottom.right"
             ) +
  
  scale_y_continuous(n.breaks = 10)+
  theme(legend.position = "top",
        axis.text.y = element_text(face = "italic"))
```

```
# ggsave(path = "figures_supplementary", "orchard_phyto_species.png", width = 130, height = 125, units = "mm", dpi=700)
```

*Pseudo-nitzschia* spp. are generally induced more than than
*Alexandrium* spp., but several of the species differ from each
other. Most of the variation is due to sampling variance.

#### 5.1.3 Phytoplankton strain

Contains an NA, we therefore remove the row with the NA

```
mod_2 <- rma.mv(yi = delta_RR,
                V = Var_delta_RR, 
                mods = ~ 1 + Phytoplankton_strain, 
                method = "REML", 
                test = "t", 
                random = list(~1 | Article_ID, 
                              ~1 + Phytoplankton_strain| Effect_ID),
                rho = 0, 
                struc = "HCS", 
                data = data_MA_full %>%  drop_na(Phytoplankton_strain), 
                #control = list(optimizer="optim", optmethod="Nelder-Mead")
                )

summary.rma(mod_2)
```

```
## 
## Multivariate Meta-Analysis Model (k = 112; method: REML)
## 
##    logLik   Deviance        AIC        BIC       AICc   
##  -80.2204   160.4407   302.4407   468.8509  2347.2407   
## 
## Variance Components:
## 
##             estim    sqrt  nlvls  fixed      factor 
## sigma^2    0.2479  0.4979     36     no  Article_ID 
## 
## outer factor: Effect_ID            (nlvls = 112)
## inner factor: Phytoplankton_strain (nlvls = 35)
## 
##              estim    sqrt  k.lvl  fixed        level 
## tau^2.1     0.0183  0.1353      1     no        1877C 
## tau^2.2     0.6210  0.7881      2     no        A-11c 
## tau^2.3     0.0183  0.1353      1     no        AI420 
## tau^2.4     0.3250  0.5701     23     no        AL-1V 
## tau^2.5     0.6562  0.8100      2     no         AL3T 
## tau^2.6     0.0151  0.1228      5     no        Alex2 
## tau^2.7     0.0168  0.1296      3     no        Alex5 
## tau^2.8     0.6605  0.8127      3     no       AM89BM 
## tau^2.9     0.0183  0.1353      1     no       AMAD16 
## tau^2.10    0.2681  0.5178      3     no       AmKB02 
## tau^2.11    0.4824  0.6945      8     no  ATKR-020415 
## tau^2.12    0.7754  0.8806     10     no         BF-5 
## tau^2.13    0.0183  0.1353      1     no          CA2 
## tau^2.14    0.1112  0.3335      3     no       Clone5 
## tau^2.15    1.9887  1.4102      5     no    CNR AMIA5 
## tau^2.16    1.2202  1.1046     11     no      Disko 8 
## tau^2.17    0.0183  0.1353      1     no          Hv5 
## tau^2.18    0.0183  0.1353      1     no           I1 
## tau^2.19    0.0000  0.0000      2     no  IFR-PAU-010 
## tau^2.20    0.1427  0.3778      2     no          Kci 
## tau^2.21    0.0000  0.0000      3     no         No.1 
## tau^2.22    0.0183  0.1353      1     no         No.9 
## tau^2.23    1.1785  1.0856      2     no         P1D2 
## tau^2.24    0.0183  0.1353      1     no         P3B2 
## tau^2.25    0.0258  0.1606      4     no         P5G3 
## tau^2.26    0.0183  0.1353      1     no         P6B3 
## tau^2.27    0.0467  0.2160      2     no       PNfra2 
## tau^2.28    0.0183  0.1353      1     no      PNfra29 
## tau^2.29    0.0000  0.0002      2     no      PNfra31 
## tau^2.30    0.0183  0.1353      1     no     PNpun102 
## tau^2.31    0.0183  0.1353      1     no     PNpun103 
## tau^2.32    0.0000  0.0000      2     no      PNpun47 
## tau^2.33    0.0183  0.1353      1     no      PNpun66 
## tau^2.34    0.0183  0.1353      1     no         PSH1 
## tau^2.35    0.0183  0.1353      1     no       SKC620 
## rho         0.0000                   yes              
## 
## Test for Residual Heterogeneity:
## QE(df = 77) = 3766.6687, p-val < .0001
## 
## Test of Moderators (coefficients 2:35):
## F(df1 = 34, df2 = 77) = 19.8340, p-val < .0001
## 
## Model Results:
## 
##                                  estimate      se     tval  df    pval    ci.lb 
## intrcpt                            1.6425  0.4943   3.3228  77  0.0014   0.6582 
## Phytoplankton_strainA-11c          0.6734  0.8969   0.7508  77  0.4551  -1.1126 
## Phytoplankton_strainAI420         -0.9419  0.7426  -1.2683  77  0.2085  -2.4207 
## Phytoplankton_strainAL-1V         -0.3704  0.5303  -0.6985  77  0.4870  -1.4263 
## Phytoplankton_strainAL3T          -1.1187  0.9100  -1.2294  77  0.2227  -2.9308 
## Phytoplankton_strainAlex2         -1.0209  0.6110  -1.6710  77  0.0988  -2.2375 
## Phytoplankton_strainAlex5         -1.4608  0.6214  -2.3509  77  0.0213  -2.6981 
## Phytoplankton_strainAM89BM         1.2845  0.7962   1.6132  77  0.1108  -0.3010 
## Phytoplankton_strainAMAD16         0.2632  0.7555   0.3484  77  0.7285  -1.2412 
## Phytoplankton_strainAmKB02        -1.0385  0.6834  -1.5196  77  0.1327  -2.3994 
## Phytoplankton_strainATKR-020415   -0.9760  0.6324  -1.5434  77  0.1268  -2.2352 
## Phytoplankton_strainBF-5          -0.6370  0.6401  -0.9952  77  0.3228  -1.9116 
## Phytoplankton_strainCA2           -2.5485  0.7348  -3.4682  77  0.0009  -4.0116 
## Phytoplankton_strainClone5        -1.3402  0.7303  -1.8351  77  0.0704  -2.7944 
## Phytoplankton_strainCNR AMIA5     -0.3412  0.8409  -0.4057  77  0.6861  -2.0157 
## Phytoplankton_strainDisko 8        1.1278  0.5147   2.1910  77  0.0315   0.1028 
## Phytoplankton_strainHv5            0.4646  0.3230   1.4383  77  0.1544  -0.1786 
## Phytoplankton_strainI1             0.0674  0.9167   0.0735  77  0.9416  -1.7580 
## Phytoplankton_strainIFR-PAU-010   -0.7535  0.6456  -1.1671  77  0.2468  -2.0389 
## Phytoplankton_strainKci           -0.8772  0.7729  -1.1349  77  0.2599  -2.4161 
## Phytoplankton_strainNo.1          -0.9151  0.7091  -1.2905  77  0.2008  -2.3272 
## Phytoplankton_strainNo.9          -0.4327  0.7331  -0.5902  77  0.5568  -1.8925 
## Phytoplankton_strainP1D2           1.1399  0.9875   1.1543  77  0.2519  -0.8265 
## Phytoplankton_strainP3B2           5.1819  0.6598   7.8542  77  <.0001   3.8681 
## Phytoplankton_strainP5G3           1.8947  0.6217   3.0475  77  0.0032   0.6567 
## Phytoplankton_strainP6B3          -0.8049  0.6818  -1.1806  77  0.2414  -2.1625 
## Phytoplankton_strainPNfra2         0.6356  0.7027   0.9045  77  0.3685  -0.7637 
## Phytoplankton_strainPNfra29       -0.1853  0.7473  -0.2479  77  0.8049  -1.6734 
## Phytoplankton_strainPNfra31       -0.8273  0.6460  -1.2807  77  0.2041  -2.1136 
## Phytoplankton_strainPNpun102       0.6747  0.7533   0.8957  77  0.3732  -0.8252 
## Phytoplankton_strainPNpun103      -0.5596  0.6786  -0.8246  77  0.4121  -1.9108 
## Phytoplankton_strainPNpun47       -0.4035  0.6904  -0.5845  77  0.5606  -1.7782 
## Phytoplankton_strainPNpun66       -1.4536  0.7126  -2.0398  77  0.0448  -2.8726 
## Phytoplankton_strainPSH1           0.8501  0.4671   1.8201  77  0.0726  -0.0799 
## Phytoplankton_strainSKC620         2.9216  0.8405   3.4759  77  0.0008   1.2479 
##                                    ci.ub      
## intrcpt                           2.6268   ** 
## Phytoplankton_strainA-11c         2.4594      
## Phytoplankton_strainAI420         0.5369      
## Phytoplankton_strainAL-1V         0.6855      
## Phytoplankton_strainAL3T          0.6933      
## Phytoplankton_strainAlex2         0.1957    . 
## Phytoplankton_strainAlex5        -0.2235    * 
## Phytoplankton_strainAM89BM        2.8700      
## Phytoplankton_strainAMAD16        1.7677      
## Phytoplankton_strainAmKB02        0.3223      
## Phytoplankton_strainATKR-020415   0.2832      
## Phytoplankton_strainBF-5          0.6376      
## Phytoplankton_strainCA2          -1.0853  *** 
## Phytoplankton_strainClone5        0.1141    . 
## Phytoplankton_strainCNR AMIA5     1.3333      
## Phytoplankton_strainDisko 8       2.1528    * 
## Phytoplankton_strainHv5           1.1078      
## Phytoplankton_strainI1            1.8928      
## Phytoplankton_strainIFR-PAU-010   0.5320      
## Phytoplankton_strainKci           0.6618      
## Phytoplankton_strainNo.1          0.4970      
## Phytoplankton_strainNo.9          1.0271      
## Phytoplankton_strainP1D2          3.1063      
## Phytoplankton_strainP3B2          6.4956  *** 
## Phytoplankton_strainP5G3          3.1327   ** 
## Phytoplankton_strainP6B3          0.5527      
## Phytoplankton_strainPNfra2        2.0349      
## Phytoplankton_strainPNfra29       1.3029      
## Phytoplankton_strainPNfra31       0.4590      
## Phytoplankton_strainPNpun102      2.1747      
## Phytoplankton_strainPNpun103      0.7916      
## Phytoplankton_strainPNpun47       0.9711      
## Phytoplankton_strainPNpun66      -0.0346    * 
## Phytoplankton_strainPSH1          1.7801    . 
## Phytoplankton_strainSKC620        4.5953  *** 
## 
## ---
## Signif. codes:  0 '***' 0.001 '**' 0.01 '*' 0.05 '.' 0.1 ' ' 1
```

```
i2_ml(mod_2)
```

```
##      I2_Total I2_Article_ID 
##      96.49613      96.49613
```

```
100 - i2_ml(mod_2)[1] # Sampling variance as proportion of total unaccounted variance
```

```
## I2_Total 
## 3.503872
```

```
r2_ml(mod_2)
```

```
##    R2_marginal R2_conditional 
##      0.8216717      0.8216717
```

```
## Orchard plot
orchard_plot(mod_2,
             data = data_MA_full %>%  drop_na(Phytoplankton_strain),
             mod = "Phytoplankton_strain",
             group = "Article_ID", # Colour of the bubbles in the plot & number in parentheses.
             xlab = bquote(LRR^~Delta),
             angle = 0,
             alpha = 0.4,
             cb = FALSE,
             k = TRUE,
             g = TRUE,
             trunk.size = 8,
             branch.size = 2,
             twig.size = 0.5,
             colour = TRUE,
             fill = TRUE,
             legend.pos = "bottom.right"
             ) +
  
  scale_y_continuous(n.breaks = 10)+
  theme(legend.position = "top")
```

```
# ggsave(path = "figures_supplementary", "orchard_phyto_strain.png", width = 130, height = 205, units = "mm", dpi=700)
```

Some strains are different from others, but this is less informative
than species differences due to the very small sample sizes (n = 1-4)
for many strains.

#### 5.1.4 Phytoplankton group (genus)

```
mod_3 <- rma.mv(yi = delta_RR,
                V = Var_delta_RR, 
                mods = ~ 1 + Phytoplankton_group, 
                method = "REML", 
                test = "t", 
                random = list(~1 | Article_ID, 
                              ~1 + Phytoplankton_group| Effect_ID),
                rho = 0, 
                struc = "HCS", 
                data = data_MA_full, 
                control = list(optimizer="optim", optmethod="Nelder-Mead")
                )

summary.rma(mod_3)
```

```
## 
## Multivariate Meta-Analysis Model (k = 113; method: REML)
## 
##    logLik   Deviance        AIC        BIC       AICc   
## -160.6477   321.2953   331.2953   344.8430   331.8668   
## 
## Variance Components:
## 
##             estim    sqrt  nlvls  fixed      factor 
## sigma^2    0.3515  0.5928     37     no  Article_ID 
## 
## outer factor: Effect_ID           (nlvls = 113)
## inner factor: Phytoplankton_group (nlvls = 2)
## 
##             estim    sqrt  k.lvl  fixed             level 
## tau^2.1    0.4833  0.6952     75     no       Alexandrium 
## tau^2.2    1.8312  1.3532     38     no  Pseudo-nitzschia 
## rho        0.0000                   yes                   
## 
## Test for Residual Heterogeneity:
## QE(df = 111) = 10515.0012, p-val < .0001
## 
## Test of Moderators (coefficient 2):
## F(df1 = 1, df2 = 111) = 15.6692, p-val = 0.0001
## 
## Model Results:
## 
##                                      estimate      se    tval   df    pval 
## intrcpt                                1.0757  0.1454  7.3989  111  <.0001 
## Phytoplankton_groupPseudo-nitzschia    1.2979  0.3279  3.9584  111  0.0001 
##                                       ci.lb   ci.ub      
## intrcpt                              0.7876  1.3638  *** 
## Phytoplankton_groupPseudo-nitzschia  0.6482  1.9476  *** 
## 
## ---
## Signif. codes:  0 '***' 0.001 '**' 0.01 '*' 0.05 '.' 0.1 ' ' 1
```

```
i2_ml(mod_3)
```

```
##      I2_Total I2_Article_ID 
##      97.72178      97.72178
```

```
100 - i2_ml(mod_3)[1] # Sampling variance as proportion of total unaccounted variance
```

```
## I2_Total 
## 2.278221
```

```
r2_ml(mod_3)
```

```
##    R2_marginal R2_conditional 
##      0.5190654      0.5190654
```

```
## Orchard plot
orchard_plot(mod_3,
             data = data_MA_full,
             mod = "Phytoplankton_group",
             group = "Article_ID", # Colour of the bubbles in the plot & number in parentheses.
             xlab = bquote(LRR^~Delta),
             angle = 0,
             alpha = 0.4,
             cb = FALSE,
             k = TRUE,
             g = TRUE,
             trunk.size = 8,
             branch.size = 2,
             twig.size = 0.5,
             colour = TRUE,
             fill = TRUE,
             legend.pos = "bottom.right"
             ) +
  
  scale_y_continuous(n.breaks = 10)+
  theme(legend.position = "top",
        axis.text.y = element_text(face = "italic"))
```

```
# ggsave(path = "figures_supplementary", "orchard_phyto_genus.png", width = 130, height = 65, units = "mm", dpi=700)
```

*Pseudo-nitzschia* are more induced than *Alexandrium*,
but the estimate is much less precise.

#### 5.1.5 Culturing method

```
mod_4 <- rma.mv(yi = delta_RR,
                V = Var_delta_RR, 
                mods = ~ 1 + Culture_type, 
                method = "REML", 
                test = "t", 
                random = list(~1 | Article_ID, 
                              ~1 + Culture_type| Effect_ID),
                rho = 0, 
                struc = "HCS", 
                data = data_MA_full, 
                #control = list(optimizer="optim", optmethod="Nelder-Mead")
                )

summary.rma(mod_4)
```

```
## 
## Multivariate Meta-Analysis Model (k = 113; method: REML)
## 
##    logLik   Deviance        AIC        BIC       AICc   
## -172.6125   345.2250   359.2250   378.1284   360.3230   
## 
## Variance Components:
## 
##             estim    sqrt  nlvls  fixed      factor 
## sigma^2    0.5468  0.7394     37     no  Article_ID 
## 
## outer factor: Effect_ID    (nlvls = 113)
## inner factor: Culture_type (nlvls = 3)
## 
##             estim    sqrt  k.lvl  fixed            level 
## tau^2.1    1.1387  1.0671    101     no            Batch 
## tau^2.2    0.0000  0.0000      3     no       Continuous 
## tau^2.3    0.3149  0.5612      9     no  Semi-continuous 
## rho        0.0000                   yes                  
## 
## Test for Residual Heterogeneity:
## QE(df = 110) = 17480.2062, p-val < .0001
## 
## Test of Moderators (coefficients 2:3):
## F(df1 = 2, df2 = 110) = 0.9593, p-val = 0.3863
## 
## Model Results:
## 
##                              estimate      se    tval   df    pval    ci.lb 
## intrcpt                        1.3930  0.1785  7.8037  110  <.0001   1.0393 
## Culture_typeContinuous         0.4310  0.6284  0.6858  110  0.4943  -0.8144 
## Culture_typeSemi-continuous    0.5814  0.4647  1.2512  110  0.2135  -0.3395 
##                               ci.ub      
## intrcpt                      1.7468  *** 
## Culture_typeContinuous       1.6764      
## Culture_typeSemi-continuous  1.5023      
## 
## ---
## Signif. codes:  0 '***' 0.001 '**' 0.01 '*' 0.05 '.' 0.1 ' ' 1
```

```
i2_ml(mod_4)
```

```
##      I2_Total I2_Article_ID 
##      98.52356      98.52356
```

```
100 - i2_ml(mod_4)[1] # Sampling variance as proportion of total unaccounted variance
```

```
## I2_Total 
## 1.476436
```

```
r2_ml(mod_4)
```

```
##    R2_marginal R2_conditional 
##     0.04999354     0.04999354
```

```
## Orchard plot
orchard_plot(mod_4,
             data = data_MA_full,
             mod = "Culture_type",
             group = "Article_ID", # Colour of the bubbles in the plot & number in parentheses.
             xlab = bquote(LRR^~Delta),
             angle = 0,
             alpha = 0.4,
             cb = FALSE,
             k = TRUE,
             g = TRUE,
             trunk.size = 8,
             branch.size = 2,
             twig.size = 0.5,
             colour = TRUE,
             fill = TRUE,
             legend.pos = "bottom.right"
             ) +
  
  scale_y_continuous(n.breaks = 10)+
  theme(legend.position = "top")
```

~90% of effect sizes are from batch culture experiments, but none of
the groups differ from each other.

#### 5.1.6 Culture medium

```
mod_5 <- rma.mv(yi = delta_RR,
                V = Var_delta_RR, 
                mods = ~ 1 + Experiment_medium, 
                method = "REML", 
                test = "t", 
                random = list(~1 | Article_ID,
                              ~1 + Experiment_medium| Effect_ID),
                rho = 0, 
                struc = "HCS", 
                data = data_MA_full, 
                #control = list(optimizer="optim", optmethod="Nelder-Mead")
                )

summary.rma(mod_5)
```

```
## 
## Multivariate Meta-Analysis Model (k = 113; method: REML)
## 
##    logLik   Deviance        AIC        BIC       AICc   
## -135.2554   270.5107   328.5107   403.7692   353.7281   
## 
## Variance Components:
## 
##             estim    sqrt  nlvls  fixed      factor 
## sigma^2    0.5077  0.7126     37     no  Article_ID 
## 
## outer factor: Effect_ID         (nlvls = 113)
## inner factor: Experiment_medium (nlvls = 14)
## 
##              estim    sqrt  k.lvl  fixed                              level 
## tau^2.1     0.1477  0.3843      2     no  B1 (reduced nitrogen, 60 µM NO3-) 
## tau^2.2     0.0682  0.2612      2     no  B1 (reduced nitrogen, 80 µM NO3-) 
## tau^2.3     1.3504  1.1621     48     no                             Custom 
## tau^2.4     0.0741  0.2722      1     no                          Diluted K 
## tau^2.5     0.3880  0.6229      9     no                                F/2 
## tau^2.6     0.0026  0.0509      3     no                                F/4 
## tau^2.7     0.0741  0.2722      1     no                                FSW 
## tau^2.8     0.1045  0.3233      9     no                                  K 
## tau^2.9     0.5673  0.7532     17     no                               K/10 
## tau^2.10    0.0000  0.0018      3     no               K/10 w 25% phosphate 
## tau^2.11    1.3921  1.1799      8     no                                 L1 
## tau^2.12    0.0741  0.2722      1     no                           L1 + NH4 
## tau^2.13    0.0240  0.1550      2     no                        L1/10 + NH4 
## tau^2.14    0.8330  0.9127      7     no                         L1/4 + NH4 
## rho         0.0000                   yes                                    
## 
## Test for Residual Heterogeneity:
## QE(df = 99) = 8672.3535, p-val < .0001
## 
## Test of Moderators (coefficients 2:14):
## F(df1 = 13, df2 = 99) = 2.7119, p-val = 0.0026
## 
## Model Results:
## 
##                                                     estimate      se     tval 
## intrcpt                                               1.4638  0.7714   1.8977 
## Experiment_mediumB1 (reduced nitrogen, 80 µM NO3-)    0.1950  0.3928   0.4963 
## Experiment_mediumCustom                              -0.2987  0.8115  -0.3680 
## Experiment_mediumDiluted K                           -0.5878  1.0901  -0.5392 
## Experiment_mediumF/2                                 -0.1377  0.8682  -0.1586 
## Experiment_mediumF/4                                  0.0631  0.9235   0.0683 
## Experiment_mediumFSW                                  0.6393  0.9413   0.6792 
## Experiment_mediumK                                   -0.0016  0.9293  -0.0017 
## Experiment_mediumK/10                                -0.5661  0.8772  -0.6454 
## Experiment_mediumK/10 w 25% phosphate                -0.7183  1.0520  -0.6828 
## Experiment_mediumL1                                   2.2633  0.9463   2.3918 
## Experiment_mediumL1 + NH4                             0.4890  1.1036   0.4431 
## Experiment_mediumL1/10 + NH4                          1.8568  1.0596   1.7522 
## Experiment_mediumL1/4 + NH4                           0.4395  0.9615   0.4571 
##                                                     df    pval    ci.lb   ci.ub 
## intrcpt                                             99  0.0607  -0.0668  2.9944 
## Experiment_mediumB1 (reduced nitrogen, 80 µM NO3-)  99  0.6208  -0.5845  0.9744 
## Experiment_mediumCustom                             99  0.7136  -1.9090  1.3116 
## Experiment_mediumDiluted K                          99  0.5910  -2.7507  1.5752 
## Experiment_mediumF/2                                99  0.8743  -1.8604  1.5850 
## Experiment_mediumF/4                                99  0.9457  -1.7692  1.8954 
## Experiment_mediumFSW                                99  0.4986  -1.2285  2.5071 
## Experiment_mediumK                                  99  0.9986  -1.8455  1.8424 
## Experiment_mediumK/10                               99  0.5202  -2.3067  1.1744 
## Experiment_mediumK/10 w 25% phosphate               99  0.4963  -2.8058  1.3691 
## Experiment_mediumL1                                 99  0.0187   0.3857  4.1410 
## Experiment_mediumL1 + NH4                           99  0.6586  -1.7007  2.6787 
## Experiment_mediumL1/10 + NH4                        99  0.0828  -0.2458  3.9593 
## Experiment_mediumL1/4 + NH4                         99  0.6486  -1.4683  2.3473 
##                                                       
## intrcpt                                             . 
## Experiment_mediumB1 (reduced nitrogen, 80 µM NO3-)    
## Experiment_mediumCustom                               
## Experiment_mediumDiluted K                            
## Experiment_mediumF/2                                  
## Experiment_mediumF/4                                  
## Experiment_mediumFSW                                  
## Experiment_mediumK                                    
## Experiment_mediumK/10                                 
## Experiment_mediumK/10 w 25% phosphate                 
## Experiment_mediumL1                                 * 
## Experiment_mediumL1 + NH4                             
## Experiment_mediumL1/10 + NH4                        . 
## Experiment_mediumL1/4 + NH4                           
## 
## ---
## Signif. codes:  0 '***' 0.001 '**' 0.01 '*' 0.05 '.' 0.1 ' ' 1
```

```
i2_ml(mod_5)
```

```
##      I2_Total I2_Article_ID 
##      98.41183      98.41183
```

```
100 - i2_ml(mod_5)[1] # Sampling variance as proportion of total unaccounted variance
```

```
## I2_Total 
## 1.588175
```

```
r2_ml(mod_5)
```

```
##    R2_marginal R2_conditional 
##      0.5205634      0.5205634
```

```
# ---- Breaks (raw levels in the data) ----
x_breaks <- c(
  "L1/4 + NH4",
  "L1/10 + NH4",
  "L1 + NH4",
  "L1",
  "K/10 w 25% phosphate",
  "K/10",
  "K",
  "FSW",
  "F/4",
  "F/2",
  "Diluted K",
  "Custom",
  "B1 (reduced nitrogen, 80 µM NO3-)",
  "B1 (reduced nitrogen, 60 µM NO3-)"
)

# ---- Labels (plotmath; keep L1 as literal text) ----
x_labels <- expression(
  "L1/4"  ~ "+" ~ NH[4],
  "L1/10" ~ "+" ~ NH[4],
  "L1"    ~ "+" ~ NH[4],
  "L1",

  "K/10 w 25% phosphate",
  "K/10",
  "K",
  "FSW",
  "F/4",
  "F/2",
  "Diluted K",
  "Custom",

  "B1 (reduced nitrogen, 80 " * mu * "M " * NO[3]^"–" * ")",
  "B1 (reduced nitrogen, 60 " * mu * "M " * NO[3]^"–" * ")"
)

## Orchard plot
orchard_plot(mod_5,
             data = data_MA_full,
             mod = "Experiment_medium",
             group = "Article_ID", # Colour of the bubbles in the plot & number in parentheses.
             xlab = bquote(LRR^~Delta),
             angle = 0,
             alpha = 0.4,
             cb = FALSE,
             k = TRUE,
             g = TRUE,
             trunk.size = 8,
             branch.size = 2,
             twig.size = 0.5,
             colour = TRUE,
             fill = TRUE,
             legend.pos = "bottom.right"
             ) +
  
    # ---- Apply manual labels ----
  scale_x_discrete(
    breaks = x_breaks,    # the “raw” labels
    labels = x_labels     # the “pretty” labels
  ) +
  
  scale_y_continuous(n.breaks = 10)+
  theme(legend.position = "top")
```

```
# ggsave(path = "figures_supplementary", "orchard_culture_medium_13.png", width = 150, height = 130, units = "mm", dpi=700)
```

Approximately 43% of effect sizes came from experimental contrasts
with custom/ill defined culture mediums. However, there are some
structure in the data, e.g., standard L1 medium effects are
significantly higher than K & F mediums. It is important to note the
small sample size in several subgroups.

#### 5.1.7 Zooplankton species

```
mod_6 <- rma.mv(yi = delta_RR,
                V = Var_delta_RR, 
                mods = ~ 1 + Zooplankton_species, 
                method = "REML", 
                test = "t", 
                random = list(~1 | Article_ID, 
                              ~1 + Zooplankton_species| Effect_ID),
                rho = 0, 
                struc = "HCS", 
                data = data_MA_full %>%   drop_na(Zooplankton_species), 
                #control = list(optimizer="optim", optmethod="Nelder-Mead")
                )


summary.rma(mod_6)
```

```
## 
## Multivariate Meta-Analysis Model (k = 50; method: REML)
## 
##   logLik  Deviance       AIC       BIC      AICc   
## -32.8610   65.7220  119.7220  163.2168  287.7220   
## 
## Variance Components:
## 
##             estim    sqrt  nlvls  fixed      factor 
## sigma^2    0.8010  0.8950     17     no  Article_ID 
## 
## outer factor: Effect_ID           (nlvls = 50)
## inner factor: Zooplankton_species (nlvls = 13)
## 
##              estim    sqrt  k.lvl  fixed                   level 
## tau^2.1     0.1909  0.4369      4     no          Acartia clausi 
## tau^2.2     0.3598  0.5999      6     no       Acartia hudsonica 
## tau^2.3     0.0061  0.0781      7     no           Acartia tonsa 
## tau^2.4     0.2780  0.5273      8     no    Calanus finmarchicus 
## tau^2.5     0.0366  0.1914      1     no   Calanus helgolandicus 
## tau^2.6     0.0366  0.1914      1     no     Calanus hyperboreus 
## tau^2.7     0.0584  0.2416      3     no              Calanus sp 
## tau^2.8     0.0000  0.0000      3     no     Centropages hamatus 
## tau^2.9     0.2536  0.5036     11     no     Centropages typicus 
## tau^2.10    0.0000  0.0001      2     no     Eurytemora herdmani 
## tau^2.11    0.0366  0.1914      1     no         Oithona similis 
## tau^2.12    0.0366  0.1914      1     no  Paraeuchaeta norvegica 
## tau^2.13    0.0101  0.1006      2     no        Pseudocalanus sp 
## rho         0.0000                   yes                         
## 
## Test for Residual Heterogeneity:
## QE(df = 37) = 1296.2418, p-val < .0001
## 
## Test of Moderators (coefficients 2:13):
## F(df1 = 12, df2 = 37) = 6.1260, p-val < .0001
## 
## Model Results:
## 
##                                            estimate      se     tval  df 
## intrcpt                                     -0.0117  0.4499  -0.0261  37 
## Zooplankton_speciesAcartia hudsonica         1.6264  0.8202   1.9830  37 
## Zooplankton_speciesAcartia tonsa             1.3179  0.4925   2.6760  37 
## Zooplankton_speciesCalanus finmarchicus      2.4600  0.5636   4.3650  37 
## Zooplankton_speciesCalanus helgolandicus     0.5174  0.4690   1.1031  37 
## Zooplankton_speciesCalanus hyperboreus       2.4341  0.7810   3.1168  37 
## Zooplankton_speciesCalanus sp                1.5849  0.5455   2.9057  37 
## Zooplankton_speciesCentropages hamatus       1.7900  0.5297   3.3794  37 
## Zooplankton_speciesCentropages typicus       1.6265  0.4272   3.8074  37 
## Zooplankton_speciesEurytemora herdmani       0.6830  1.0037   0.6805  37 
## Zooplankton_speciesOithona similis          -0.1448  0.4710  -0.3075  37 
## Zooplankton_speciesParaeuchaeta norvegica    0.1404  0.5928   0.2369  37 
## Zooplankton_speciesPseudocalanus sp         -0.2138  0.3222  -0.6635  37 
##                                              pval    ci.lb   ci.ub      
## intrcpt                                    0.9793  -0.9234  0.8999      
## Zooplankton_speciesAcartia hudsonica       0.0548  -0.0355  3.2882    . 
## Zooplankton_speciesAcartia tonsa           0.0110   0.3200  2.3157    * 
## Zooplankton_speciesCalanus finmarchicus    <.0001   1.3181  3.6019  *** 
## Zooplankton_speciesCalanus helgolandicus   0.2771  -0.4329  1.4676      
## Zooplankton_speciesCalanus hyperboreus     0.0035   0.8517  4.0165   ** 
## Zooplankton_speciesCalanus sp              0.0062   0.4797  2.6901   ** 
## Zooplankton_speciesCentropages hamatus     0.0017   0.7168  2.8632   ** 
## Zooplankton_speciesCentropages typicus     0.0005   0.7609  2.4920  *** 
## Zooplankton_speciesEurytemora herdmani     0.5004  -1.3506  2.7167      
## Zooplankton_speciesOithona similis         0.7602  -1.0992  0.8096      
## Zooplankton_speciesParaeuchaeta norvegica  0.8141  -1.0607  1.3415      
## Zooplankton_speciesPseudocalanus sp        0.5111  -0.8667  0.4391      
## 
## ---
## Signif. codes:  0 '***' 0.001 '**' 0.01 '*' 0.05 '.' 0.1 ' ' 1
```

```
i2_ml(mod_6)
```

```
##      I2_Total I2_Article_ID 
##      98.42738      98.42738
```

```
100 - i2_ml(mod_6)[1] # Sampling variance as proportion of total unaccounted variance
```

```
## I2_Total 
## 1.572618
```

```
r2_ml(mod_6)
```

```
##    R2_marginal R2_conditional 
##       0.436123       0.436123
```

```
## Orchard plot
orchard_plot(mod_6,
             data = data_MA_full %>%   drop_na(Zooplankton_species),
             mod = "Zooplankton_species",
             group = "Article_ID", # Colour of the bubbles in the plot & number in parentheses.
             xlab = bquote(LRR^~Delta),
             angle = 0,
             alpha = 0.4,
             cb = FALSE,
             k = TRUE,
             g = TRUE,
             trunk.size = 8,
             branch.size = 2,
             twig.size = 0.5,
             colour = TRUE,
             fill = TRUE,
             legend.pos = "bottom.right"
             ) +
  
  
  
  scale_y_continuous(n.breaks = 10)+
      scale_x_discrete(labels = c("Acartia clausi" = "*Acartia clausi*",
                              "Acartia hudsonica" = "*Acartia hudsonica*",
                              "Acartia tonsa" = "*Acartia tonsa*",
                              "Calanus finmarchicus" = "*Calanus finmarchicus*",
                              "Calanus helgolandicus" = "*Calanus helgolandicus*",
                              "Calanus hyperboreus" = "*Calanus hyperboreus*",
                              "Calanus sp" = "*Calanus* sp.",
                              "Centropages hamatus" = "*Centropages hamatus*",
                              "Centropages typicus" = "*Centropages typicus*",
                              "Eurytemora herdmani" = "*Eurytemora herdmani*",
                              "Oithona similis" = "*Oithona similis*",
                              "Paraeuchaeta norvegica" = "*Paraeuchaeta norvegica*",
                              "Pseudocalanus sp" = "*Pseudocalanus* sp."))+
  theme(legend.position = "top",
        axis.text.y = element_markdown(size = 11, colour = "black"))
```

```
# ggsave(path = "figures_supplementary", "orchard_zooplankton.png", width = 130, height = 120, units = "mm", dpi=700)
```

Several of the copepod species differ from each other.

#### 5.1.8 Resource type (nitrogen surplus vs phosphorous limitation) and grazing pressure (direct grazing, indirect grazing, starving grazer or chemical cues)

```
mod_7 <- rma.mv(yi = delta_RR,
                V = Var_delta_RR, 
                mods = ~ 1 + Group, 
                method = "REML", 
                test = "t", 
                random = list(~1 | Article_ID,
                              ~1 + Group| Effect_ID),
                rho = 0, 
                struc = "HCS", 
                data = data_MA_full, 
                #control = list(optimizer="optim", optmethod="Nelder-Mead")
                )


summary.rma(mod_7)
```

```
## 
## Multivariate Meta-Analysis Model (k = 113; method: REML)
## 
##    logLik   Deviance        AIC        BIC       AICc   
## -160.6048   321.2096   347.2096   381.9564   351.1236   
## 
## Variance Components:
## 
##             estim    sqrt  nlvls  fixed      factor 
## sigma^2    0.3111  0.5577     37     no  Article_ID 
## 
## outer factor: Effect_ID (nlvls = 113)
## inner factor: Group     (nlvls = 6)
## 
##             estim    sqrt  k.lvl  fixed             level 
## tau^2.1    1.4485  1.2035     20     no     Chemical cues 
## tau^2.2    0.0535  0.2313     17     no    Direct grazing 
## tau^2.3    1.1740  1.0835     24     no  Indirect grazing 
## tau^2.4    0.9738  0.9868     16     no          Nitrogen 
## tau^2.5    1.7042  1.3054     31     no       Phosphorous 
## tau^2.6    0.4999  0.7070      5     no   Starving grazer 
## rho        0.0000                   yes                   
## 
## Test for Residual Heterogeneity:
## QE(df = 107) = 9814.4478, p-val < .0001
## 
## Test of Moderators (coefficients 2:6):
## F(df1 = 5, df2 = 107) = 1.8394, p-val = 0.1113
## 
## Model Results:
## 
##                        estimate      se     tval   df    pval    ci.lb    ci.ub 
## intrcpt                  2.0981  0.3738   5.6121  107  <.0001   1.3570   2.8392 
## GroupDirect grazing     -0.6927  0.4064  -1.7044  107  0.0912  -1.4984   0.1130 
## GroupIndirect grazing   -0.7570  0.4620  -1.6384  107  0.1043  -1.6730   0.1590 
## GroupNitrogen           -1.3378  0.4679  -2.8592  107  0.0051  -2.2653  -0.4102 
## GroupPhosphorous        -0.5047  0.4831  -1.0448  107  0.2985  -1.4623   0.4529 
## GroupStarving grazer    -0.6346  0.5237  -1.2118  107  0.2282  -1.6728   0.4035 
##                            
## intrcpt                *** 
## GroupDirect grazing      . 
## GroupIndirect grazing      
## GroupNitrogen           ** 
## GroupPhosphorous           
## GroupStarving grazer       
## 
## ---
## Signif. codes:  0 '***' 0.001 '**' 0.01 '*' 0.05 '.' 0.1 ' ' 1
```

```
i2_ml(mod_7)
```

```
##      I2_Total I2_Article_ID 
##       97.4334       97.4334
```

```
100 - i2_ml(mod_7)[1] # Sampling variance as proportion of total unaccounted variance
```

```
## I2_Total 
## 2.566595
```

```
r2_ml(mod_7)
```

```
##    R2_marginal R2_conditional 
##      0.3264425      0.3264425
```

```
## Orchard plot
orchard_plot(mod_7,
             data = data_MA_full,
             mod = "Group",
             group = "Article_ID", # Colour of the bubbles in the plot & number in parentheses.
             xlab = bquote(LRR^~Delta),
             angle = 0,
             alpha = 0.4,
             cb = FALSE,
             k = TRUE,
             g = TRUE,
             trunk.size = 8,
             branch.size = 1.5,
             twig.size = 0.5,
             colour = TRUE,
             fill = TRUE,
             legend.pos = "bottom.right"
             ) +
  
  scale_y_continuous(n.breaks = 10)+
  theme(legend.position = "top")
```

None of the groups differ from each other but phosphorous limitation
seems to induce more toxicity than nitrogen surplus, and chemical cues
seem to be a slightly more potent toxicity inducer than living
copepods.

#### 5.1.9 Illumination

```
mod_8 <- rma.mv(yi = delta_RR,
                V = Var_delta_RR, 
                mods = ~ 1 + Light, 
                method = "REML", 
                test = "t", 
                random = list(~1 | Article_ID,
                              ~1 + Light| Effect_ID),
                rho = 0, 
                struc = "HCS", 
                data = data_MA_full %>%   drop_na(Light), 
                #control = list(optimizer="optim", optmethod="Nelder-Mead")
                )

summary.rma(mod_8)
```

```
## 
## Multivariate Meta-Analysis Model (k = 100; method: REML)
## 
##    logLik   Deviance        AIC        BIC       AICc   
## -147.1519   294.3037   322.3037   358.4933   327.3640   
## 
## Variance Components:
## 
##             estim    sqrt  nlvls  fixed      factor 
## sigma^2    0.1102  0.3319     33     no  Article_ID 
## 
## outer factor: Effect_ID (nlvls = 100)
## inner factor: Light     (nlvls = 11)
## 
##              estim    sqrt  k.lvl  fixed  level 
## tau^2.1     1.2131  1.1014      5     no     50 
## tau^2.2     3.7547  1.9377      2     no     53 
## tau^2.3     3.4507  1.8576      5     no     60 
## tau^2.4     2.1551  1.4680     16     no     80 
## tau^2.5     0.8139  0.9022      2     no     90 
## tau^2.6     1.2790  1.1309     29     no    100 
## tau^2.7     0.1718  0.4145      2     no    120 
## tau^2.8     0.5432  0.7370     28     no    150 
## tau^2.9     0.4284  0.6545      1     no    180 
## tau^2.10    0.5559  0.7456      2     no    200 
## tau^2.11    0.4710  0.6863      8     no    350 
## rho         0.0000                   yes        
## 
## Test for Residual Heterogeneity:
## QE(df = 98) = 13195.7930, p-val < .0001
## 
## Test of Moderators (coefficient 2):
## F(df1 = 1, df2 = 98) = 8.5375, p-val = 0.0043
## 
## Model Results:
## 
##          estimate      se     tval  df    pval    ci.lb    ci.ub      
## intrcpt    1.8729  0.2420   7.7388  98  <.0001   1.3926   2.3531  *** 
## Light     -0.0041  0.0014  -2.9219  98  0.0043  -0.0068  -0.0013   ** 
## 
## ---
## Signif. codes:  0 '***' 0.001 '**' 0.01 '*' 0.05 '.' 0.1 ' ' 1
```

```
i2_ml(mod_8)
```

```
##      I2_Total I2_Article_ID 
##      93.30783      93.30783
```

```
100 - i2_ml(mod_8)[1] # Sampling variance as proportion of total unaccounted variance
```

```
## I2_Total 
## 6.692166
```

```
r2_ml(mod_8)
```

```
##    R2_marginal R2_conditional 
##      0.4533782      0.4533782
```

```
SI_15A <-
# Bubble plot
bubble_plot(mod_8, 
            mod = "Light",
            data = data_MA_full %>%  drop_na(Light),
            group = "Article_ID", # Colour of the bubbles in the plot & number in parentheses.
            xlab =  bquote(Illumination~(µmol~photons~m^-2~s^-1)),
            ylab = bquote(LRR^~Delta),
            cb = FALSE,
            k = TRUE,
            g = TRUE,
            ci.lwd = 0.8,
            pi.lwd = 0.5,
            legend.pos = "bottom.right")+
  
  scale_x_continuous(n.breaks = 10)+
  theme(legend.position = "top")

SI_15A
```

```
# ggsave(path = "figures_supplementary", "bubble_light.png", width = 130, height = 100, units = "mm", dpi=700)
```

Significant negative effect of light on effect size.

#### 5.1.10 Light:dark cycle

```
mod_9 <- rma.mv(yi = delta_RR,
                V = Var_delta_RR, 
                mods = ~ 1 + LD_cycle, 
                method = "REML", 
                test = "t", 
                random = list(~1 | Article_ID,
                              ~1 + LD_cycle| Effect_ID),
                rho = 0, 
                struc = "HCS", 
                data = data_MA_full %>%   drop_na(LD_cycle), 
                #control = list(optimizer="optim", optmethod="Nelder-Mead")
                )

summary.rma(mod_9)
```

```
## 
## Multivariate Meta-Analysis Model (k = 110; method: REML)
## 
##    logLik   Deviance        AIC        BIC       AICc   
## -162.6188   325.2375   343.2375   367.3767   345.0742   
## 
## Variance Components:
## 
##             estim    sqrt  nlvls  fixed      factor 
## sigma^2    0.5770  0.7596     35     no  Article_ID 
## 
## outer factor: Effect_ID (nlvls = 110)
## inner factor: LD_cycle  (nlvls = 6)
## 
##             estim    sqrt  k.lvl  fixed  level 
## tau^2.1    1.1744  1.0837     56     no     12 
## tau^2.2    0.9226  0.9605     26     no     14 
## tau^2.3    0.1187  0.3445      3     no     15 
## tau^2.4    0.5081  0.7128     18     no     16 
## tau^2.5    0.0000  0.0001      2     no     18 
## tau^2.6    0.0598  0.2446      5     no     22 
## rho        0.0000                   yes        
## 
## Test for Residual Heterogeneity:
## QE(df = 108) = 11797.5749, p-val < .0001
## 
## Test of Moderators (coefficient 2):
## F(df1 = 1, df2 = 108) = 20.8421, p-val < .0001
## 
## Model Results:
## 
##           estimate      se     tval   df    pval    ci.lb   ci.ub      
## intrcpt    -1.1896  0.6277  -1.8952  108  0.0607  -2.4337  0.0546    . 
## LD_cycle    0.1895  0.0415   4.5653  108  <.0001   0.1072  0.2718  *** 
## 
## ---
## Signif. codes:  0 '***' 0.001 '**' 0.01 '*' 0.05 '.' 0.1 ' ' 1
```

```
i2_ml(mod_9)
```

```
##      I2_Total I2_Article_ID 
##      98.48647      98.48647
```

```
100 - i2_ml(mod_9)[1] # Sampling variance as proportion of total unaccounted variance
```

```
## I2_Total 
## 1.513532
```

```
r2_ml(mod_9)
```

```
##    R2_marginal R2_conditional 
##      0.2692177      0.2692177
```

```
# Bubble plot
bubble_plot(mod_9, 
            mod = "LD_cycle",
            data = data_MA_full %>%   drop_na(LD_cycle),
            group = "Article_ID", # Colour of the bubbles in the plot & number in parentheses.
            xlab = "Light hours",
            ylab = bquote(LRR^~Delta),
            cb = FALSE,
            k = TRUE,
            g = TRUE,
            legend.pos = "bottom.right")+
  
  scale_x_continuous(n.breaks = 10)
```

Significant positive effect of light hours on effect size.

#### 5.1.11 Temperature

```
mod_10 <- rma.mv(yi = delta_RR,
                V = Var_delta_RR, 
                mods = ~ 1 + Temperature, 
                method = "REML", 
                test = "t", 
                random = list(~1 | Article_ID, 
                              ~1 + Temperature| Effect_ID),
                rho = 0, 
                struc = "HCS", 
                data = data_MA_full, # %>%   drop_na(Temperature), 
                #control = list(optimizer="optim", optmethod="Nelder-Mead")
                )

summary.rma(mod_10)
```

```
## 
## Multivariate Meta-Analysis Model (k = 113; method: REML)
## 
##    logLik   Deviance        AIC        BIC       AICc   
## -159.3836   318.7673   346.7673   384.7007   351.1423   
## 
## Variance Components:
## 
##             estim    sqrt  nlvls  fixed      factor 
## sigma^2    0.4312  0.6566     37     no  Article_ID 
## 
## outer factor: Effect_ID   (nlvls = 113)
## inner factor: Temperature (nlvls = 11)
## 
##              estim    sqrt  k.lvl  fixed  level 
## tau^2.1     1.0521  1.0257     20     no      4 
## tau^2.2     0.0000  0.0002      1     no      5 
## tau^2.3     8.3560  2.8907      1     no      8 
## tau^2.4     0.2028  0.4503      7     no     15 
## tau^2.5     1.9593  1.3998     20     no     16 
## tau^2.6     0.5180  0.7197      8     no     17 
## tau^2.7     0.5649  0.7516     46     no     18 
## tau^2.8     0.0000  0.0000      1     no     19 
## tau^2.9     0.3491  0.5908      5     no     20 
## tau^2.10    0.0000  0.0000      1     no     22 
## tau^2.11    0.2535  0.5035      3     no     25 
## rho         0.0000                   yes        
## 
## Test for Residual Heterogeneity:
## QE(df = 111) = 12205.8844, p-val < .0001
## 
## Test of Moderators (coefficient 2):
## F(df1 = 1, df2 = 111) = 19.0448, p-val < .0001
## 
## Model Results:
## 
##              estimate      se     tval   df    pval    ci.lb    ci.ub      
## intrcpt        2.8576  0.3711   7.7000  111  <.0001   2.1222   3.5930  *** 
## Temperature   -0.0956  0.0219  -4.3640  111  <.0001  -0.1391  -0.0522  *** 
## 
## ---
## Signif. codes:  0 '***' 0.001 '**' 0.01 '*' 0.05 '.' 0.1 ' ' 1
```

```
i2_ml(mod_10)
```

```
##      I2_Total I2_Article_ID 
##      98.13516      98.13516
```

```
100 - i2_ml(mod_10)[1] # Sampling variance as proportion of total unaccounted variance
```

```
## I2_Total 
## 1.864839
```

```
r2_ml(mod_10)
```

```
##    R2_marginal R2_conditional 
##      0.4003781      0.4003781
```

```
SI_15B <-
bubble_plot(mod_10, 
            mod = "Temperature",
            data = data_MA_full,
            group = "Article_ID",
            xlab =  "Temperature (°C)",
            ylab = bquote(LRR^~Delta),
            cb = FALSE,
            k = TRUE,
            g = TRUE,
            ci.lwd = 0.8,
            pi.lwd = 0.5,
            legend.pos = "bottom.right")+
  
  scale_x_continuous(n.breaks = 10)+
  theme(legend.position = "top")

SI_15B
```

```
# ggsave(path = "figures_supplementary", "bubble_temp.png", width = 130, height = 100, units = "mm", dpi=700)
```

Significant negative effect of temperature on effect size.

#### 5.1.12 Experimental time

```
mod_11 <- rma.mv(yi = delta_RR,
                V = Var_delta_RR, 
                mods = ~ 1 + Hours, 
                method = "REML", 
                test = "t", 
                random = list(~1 | Article_ID, 
                              ~1 + Hours| Effect_ID),
                rho = 0, 
                struc = "HCS", 
                data = data_MA_full  %>%   drop_na(Hours), 
                #control = list(optimizer="optim", optmethod="Nelder-Mead")
                )

summary.rma(mod_11)
```

```
## 
## Multivariate Meta-Analysis Model (k = 104; method: REML)
## 
##    logLik   Deviance        AIC        BIC       AICc   
## -142.6428   285.2857   321.2857   368.5352   329.5266   
## 
## Variance Components:
## 
##             estim    sqrt  nlvls  fixed      factor 
## sigma^2    0.3523  0.5935     36     no  Article_ID 
## 
## outer factor: Effect_ID (nlvls = 104)
## inner factor: Hours     (nlvls = 15)
## 
##              estim    sqrt  k.lvl  fixed  level 
## tau^2.1     0.0903  0.3006     17     no     48 
## tau^2.2     8.0131  2.8307      4     no     60 
## tau^2.3     0.6751  0.8217     27     no     72 
## tau^2.4     0.5815  0.7626      2     no     96 
## tau^2.5     1.8982  1.3777      7     no    120 
## tau^2.6     0.6218  0.7885      6     no    144 
## tau^2.7     2.7479  1.6577      5     no    168 
## tau^2.8     3.0093  1.7347     13     no    192 
## tau^2.9     0.2052  0.4530      4     no    240 
## tau^2.10    0.3023  0.5498     11     no    336 
## tau^2.11    0.0952  0.3086      3     no    360 
## tau^2.12    0.0000  0.0001      1     no    432 
## tau^2.13    5.5608  2.3581      1     no    456 
## tau^2.14    0.0000  0.0001      1     no    504 
## tau^2.15    0.6184  0.7864      2     no   5760 
## rho         0.0000                   yes        
## 
## Test for Residual Heterogeneity:
## QE(df = 102) = 17971.1817, p-val < .0001
## 
## Test of Moderators (coefficient 2):
## F(df1 = 1, df2 = 102) = 1.5216, p-val = 0.2202
## 
## Model Results:
## 
##          estimate      se    tval   df    pval    ci.lb   ci.ub      
## intrcpt    1.1889  0.1430  8.3124  102  <.0001   0.9052  1.4726  *** 
## Hours      0.0002  0.0001  1.2336  102  0.2202  -0.0001  0.0005      
## 
## ---
## Signif. codes:  0 '***' 0.001 '**' 0.01 '*' 0.05 '.' 0.1 ' ' 1
```

```
i2_ml(mod_11)
```

```
##      I2_Total I2_Article_ID 
##      97.79858      97.79858
```

```
100 - i2_ml(mod_11)[1] # Sampling variance as proportion of total unaccounted variance
```

```
## I2_Total 
## 2.201423
```

```
r2_ml(mod_11)
```

```
##    R2_marginal R2_conditional 
##      0.0534134      0.0534134
```

```
bubble_plot(mod_11, 
            mod = "Hours",
            data = data_MA_full %>%   drop_na(Hours),
            group = "Article_ID",
            xlab = "Duration (h)",
            cb = FALSE,
            k = TRUE,
            g = TRUE,
            legend.pos = "top.left")+
  
  scale_x_continuous(n.breaks = 10)
```

```
# Removing extreme values (>1000)
mod_11 <- rma.mv(yi = delta_RR,
                V = Var_delta_RR, 
                mods = ~ 1 + Hours, 
                method = "REML", 
                test = "t", 
                random = list(~1 | Article_ID, 
                              ~1 + Hours| Effect_ID),
                rho = 0, 
                struc = "HCS", 
                data = data_MA_full  %>%   drop_na(Hours) %>%  filter(Hours <500), 
                #control = list(optimizer="optim", optmethod="Nelder-Mead")
                )

bubble_plot(mod_11, 
            mod = "Hours",
            data = data_MA_full %>%   drop_na(Hours) %>%  filter(Hours<500),
            group = "Article_ID",
            xlab = "Duration (h)",
            ylab = bquote(LRR^~Delta),
            cb = FALSE,
            k = TRUE,
            g = TRUE,
            ci.lwd = 0.8,
            pi.lwd = 0.5,
            legend.pos = "bottom.right")+
  
  scale_x_continuous(n.breaks = 10)+
  theme(legend.position = "top")+

  ggtitle("Duration < 500h")
```

No effect of experiment duration on effect size.

#### 5.1.13 N:P ratio difference in treatment compared to control

```
mod_12 <- rma.mv(yi = delta_RR,
                V = Var_delta_RR, 
                mods = ~ 1 + NP_ratio_diff, 
                method = "REML", 
                test = "t", 
                random = list(~1 | Article_ID, 
                              ~1 + NP_ratio_diff| Effect_ID),
                rho = 0, 
                struc = "HCS", 
                data = data_MA_full  %>%   
                  drop_na(NP_ratio_diff) ,
                #control = list(optimizer="optim", optmethod="Nelder-Mead")
                )


summary.rma(mod_12)
```

```
## 
## Multivariate Meta-Analysis Model (k = 110; method: REML)
## 
##    logLik   Deviance        AIC        BIC       AICc   
## -158.6267   317.2533   383.2533   471.7637   413.5777   
## 
## Variance Components:
## 
##             estim    sqrt  nlvls  fixed      factor 
## sigma^2    0.4065  0.6375     35     no  Article_ID 
## 
## outer factor: Effect_ID     (nlvls = 110)
## inner factor: NP_ratio_diff (nlvls = 30)
## 
##              estim    sqrt  k.lvl  fixed   level 
## tau^2.1     0.8585  0.9265     67     no       0 
## tau^2.2     0.0000  0.0001      1     no    0.35 
## tau^2.3     0.5771  0.7597      1     no    0.52 
## tau^2.4     1.8414  1.3570      1     no    1.21 
## tau^2.5     2.4387  1.5616      1     no    1.38 
## tau^2.6     5.9730  2.4440      1     no    2.19 
## tau^2.7     0.0000  0.0001      1     no    2.59 
## tau^2.8     0.0000  0.0000      1     no     2.6 
## tau^2.9     0.4191  0.6474      2     no    5.95 
## tau^2.10    4.3051  2.0749      7     no    11.2 
## tau^2.11    0.3166  0.5627      1     no   13.47 
## tau^2.12    0.0000  0.0000      1     no   14.86 
## tau^2.13    0.3484  0.5903      1     no   16.12 
## tau^2.14    1.4116  1.1881      1     no    18.3 
## tau^2.15    0.0000  0.0001      1     no   27.24 
## tau^2.16    0.4872  0.6980      1     no    28.2 
## tau^2.17    0.0000  0.0000      1     no   37.33 
## tau^2.18    0.4357  0.6601      1     no      40 
## tau^2.19    0.4310  0.6565      2     no      48 
## tau^2.20    0.5228  0.7230      1     no   60.25 
## tau^2.21    0.0000  0.0000      2     no   62.67 
## tau^2.22    0.5884  0.7671      2     no      64 
## tau^2.23    2.1682  1.4725      1     no   76.84 
## tau^2.24    4.0091  2.0023      1     no    78.4 
## tau^2.25    2.5396  1.5936      1     no     100 
## tau^2.26    0.5954  0.7716      1     no  103.14 
## tau^2.27    0.3577  0.5981      3     no     112 
## tau^2.28    0.5659  0.7523      3     no     144 
## tau^2.29    0.3448  0.5872      1     no  191.44 
## tau^2.30    0.0000  0.0020      1     no  668.94 
## rho         0.0000                   yes         
## 
## Test for Residual Heterogeneity:
## QE(df = 108) = 18606.1965, p-val < .0001
## 
## Test of Moderators (coefficient 2):
## F(df1 = 1, df2 = 108) = 14.4982, p-val = 0.0002
## 
## Model Results:
## 
##                estimate      se     tval   df    pval   ci.lb   ci.ub      
## intrcpt          1.4107  0.1401  10.0720  108  <.0001  1.1331  1.6884  *** 
## NP_ratio_diff    0.0021  0.0006   3.8076  108  0.0002  0.0010  0.0032  *** 
## 
## ---
## Signif. codes:  0 '***' 0.001 '**' 0.01 '*' 0.05 '.' 0.1 ' ' 1
```

```
i2_ml(mod_12)
```

```
##      I2_Total I2_Article_ID 
##      98.06783      98.06783
```

```
100 - i2_ml(mod_12)[1] # Sampling variance as proportion of total unaccounted variance
```

```
## I2_Total 
## 1.932171
```

```
r2_ml(mod_12)
```

```
##    R2_marginal R2_conditional 
##     0.05483201     0.05483201
```

```
bubble_plot(mod_12, 
            mod = "NP_ratio_diff",
            data = data_MA_full %>%   drop_na(NP_ratio_diff),
            group = "Article_ID",
            xlab = "Absolute N:P ratio difference",
            ylab = bquote(LRR^~Delta),
            cb = FALSE,
            k = TRUE,
            g = TRUE,
            ci.lwd = 0.8,
            pi.lwd = 0.5,
            legend.pos = "bottom.right")+
  
  scale_x_continuous(n.breaks = 10)+
  theme(legend.position = "top")
```

Significant positive effect of N:P ratio difference, despite all of
the “Demand” effects having zero N:P ratio differences. There is most
certainly an interaction effect with driver.

#### 5.1.14 Publication year

```
mod_year <- rma.mv(yi = delta_RR,
                V = Var_delta_RR, 
                mods = ~ 1 + Year, 
                method = "REML", 
                test = "t", 
                random = list(~1 | Article_ID, 
                              ~1 + Year| Effect_ID),
                rho = 0, 
                struc = "HCS", 
                data = data_MA_full  %>%   drop_na(Year) ,
                #control = list(optimizer="optim", optmethod="Nelder-Mead")
                )

summary.rma(mod_year)
```

```
## 
## Multivariate Meta-Analysis Model (k = 113; method: REML)
## 
##    logLik   Deviance        AIC        BIC       AICc   
## -150.6668   301.3336   343.3336   400.2337   353.7156   
## 
## Variance Components:
## 
##             estim    sqrt  nlvls  fixed      factor 
## sigma^2    0.6241  0.7900     37     no  Article_ID 
## 
## outer factor: Effect_ID (nlvls = 113)
## inner factor: Year      (nlvls = 18)
## 
##              estim    sqrt  k.lvl  fixed  level 
## tau^2.1     6.0302  2.4556      2     no   1999 
## tau^2.2     0.0000  0.0020      1     no   2002 
## tau^2.3     0.0000  0.0041      2     no   2003 
## tau^2.4     0.9746  0.9872      3     no   2004 
## tau^2.5     0.0000  0.0000      5     no   2006 
## tau^2.6     1.3353  1.1556     10     no   2008 
## tau^2.7     0.1121  0.3348      3     no   2010 
## tau^2.8     0.4462  0.6680      3     no   2011 
## tau^2.9     0.1284  0.3583     10     no   2012 
## tau^2.10    0.3202  0.5659      4     no   2013 
## tau^2.11    0.2199  0.4689     16     no   2015 
## tau^2.12    0.3970  0.6301      2     no   2016 
## tau^2.13    0.2330  0.4827     15     no   2017 
## tau^2.14    0.7145  0.8453      7     no   2018 
## tau^2.15    2.5780  1.6056     22     no   2019 
## tau^2.16    2.9859  1.7280      2     no   2020 
## tau^2.17    0.0802  0.2832      4     no   2021 
## tau^2.18    4.6318  2.1522      2     no   2022 
## rho         0.0000                   yes        
## 
## Test for Residual Heterogeneity:
## QE(df = 111) = 16532.4228, p-val < .0001
## 
## Test of Moderators (coefficient 2):
## F(df1 = 1, df2 = 111) = 1.6793, p-val = 0.1977
## 
## Model Results:
## 
##          estimate       se     tval   df    pval      ci.lb    ci.ub    
## intrcpt  -70.5482  55.4802  -1.2716  111  0.2062  -180.4859  39.3895    
## Year       0.0357   0.0276   1.2959  111  0.1977    -0.0189   0.0904    
## 
## ---
## Signif. codes:  0 '***' 0.001 '**' 0.01 '*' 0.05 '.' 0.1 ' ' 1
```

```
i2_ml(mod_year)
```

```
##      I2_Total I2_Article_ID 
##      98.70418      98.70418
```

```
100 - i2_ml(mod_year)[1] # Sampling variance as proportion of total unaccounted variance
```

```
## I2_Total 
## 1.295815
```

```
r2_ml(mod_year)
```

```
##    R2_marginal R2_conditional 
##     0.05437802     0.05437802
```

```
bubble_plot(mod_year, 
            mod = "Year",
            data = data_MA_full %>%   drop_na(Year),
            group = "Article_ID",
            xlab = "Publication year",
            cb = FALSE,
            k = TRUE,
            g = TRUE,
            legend.pos = "bottom.right")+
   scale_x_continuous(n.breaks = 10)
```

Non-significant positive trend effect size as a function of
publication year.

#### 5.1.15 Experiment type / Driver (Demand vs Resource)

This is the main analysis we are interested in and will use to
evaluate the relative effects demand and resource on toxin induction in
the two chosen taxa. We might add other variables to the model depending
on any potential interactions with experiment type further down the
line.

```
mod_13 <- rma.mv(yi = delta_RR,
                V = Var_delta_RR, 
                mods = ~ 1 + Exp_type, 
                method = "REML", 
                test = "t", 
                random = list(~1 | Article_ID,
                              ~1 + Exp_type | Effect_ID),
                rho = 0, 
                struc = "HCS", 
                data = data_MA_full,
                control = list(optimizer="optim", optmethod="Nelder-Mead")
                )


summary.rma(mod_13)
```

```
## 
## Multivariate Meta-Analysis Model (k = 113; method: REML)
## 
##    logLik   Deviance        AIC        BIC       AICc   
## -176.3887   352.7774   362.7774   376.3250   363.3488   
## 
## Variance Components:
## 
##             estim    sqrt  nlvls  fixed      factor 
## sigma^2    0.4779  0.6913     37     no  Article_ID 
## 
## outer factor: Effect_ID (nlvls = 113)
## inner factor: Exp_type  (nlvls = 2)
## 
##             estim    sqrt  k.lvl  fixed     level 
## tau^2.1    0.8489  0.9214     66     no    Demand 
## tau^2.2    1.3796  1.1745     47     no  Resource 
## rho        0.0000                   yes           
## 
## Test for Residual Heterogeneity:
## QE(df = 111) = 15817.4113, p-val < .0001
## 
## Test of Moderators (coefficient 2):
## F(df1 = 1, df2 = 111) = 0.8810, p-val = 0.3500
## 
## Model Results:
## 
##                   estimate      se     tval   df    pval    ci.lb   ci.ub      
## intrcpt             1.5855  0.1972   8.0384  111  <.0001   1.1947  1.9763  *** 
## Exp_typeResource   -0.2856  0.3043  -0.9386  111  0.3500  -0.8886  0.3174      
## 
## ---
## Signif. codes:  0 '***' 0.001 '**' 0.01 '*' 0.05 '.' 0.1 ' ' 1
```

```
i2_ml(mod_13)
```

```
##      I2_Total I2_Article_ID 
##      98.31432      98.31432
```

```
100 - i2_ml(mod_13)[1] # Sampling variance as proportion of total unaccounted variance
```

```
## I2_Total 
## 1.685683
```

```
r2_ml(mod_13)
```

```
##    R2_marginal R2_conditional 
##     0.04015862     0.04015862
```

```
res_mod_13 <- 
orchaRd::mod_results(mod_13,
                     mod = "Exp_type",
                     group = "Exp_type",
                     data = data_MA_full)$mod_table %>%  
  rename(Driver = name)

res_mod_13
```

```
##     Driver estimate   lowerCL  upperCL    lowerPR upperPR
## 1   Demand 1.585498 1.1946523 1.976345 -0.7302527 3.90125
## 2 Resource 1.299885 0.8115082 1.788262 -1.4445592 4.04433
```

```
orchard_plot(mod_13,
             data = data_MA_full,
             mod = "Exp_type",
             group = "Article_ID", # Colour of the bubbles in the plot & number in parentheses.
             xlab = "lnRR (effect size)",
             angle = 0,
             alpha = 0.4,
             cb = FALSE,
             k = TRUE,
             g = TRUE,
             trunk.size = 8,
             branch.size = 1.5,
             twig.size = 0.5,
             colour = TRUE,
             fill = TRUE,
             legend.pos = "bottom.right"
             ) +
  
  scale_y_continuous(n.breaks = 10)
```

Both resource availability (increased N:P ratio) and grazing pressure
(grazer or their chemical cues) increase toxicity several hundred
percent, but not to significantly different magnitudes compared to each
other.

### 5.2 Assessing interactions between driver and the other moderators

Here we construct multifactor models and test for significant
interaction effects using the `anova` function.

#### 5.2.1 Driver x phytoplankton species

```
mod_14 <- rma.mv(yi = delta_RR,
                V = Var_delta_RR, 
                mods = ~ 1 + (Exp_type * Phytoplankton_species), 
                method = "REML", 
                test = "t", 
                random = list(~1 | Article_ID,
                              ~1 + Exp_type| Effect_ID),
                rho = 0, 
                struc = "HCS", 
                data = data_MA_full,
                control = list(optimizer="optim", optmethod="Nelder-Mead")
                  )


summary.rma(mod_14)
```

```
## 
## Multivariate Meta-Analysis Model (k = 113; method: REML)
## 
##    logLik   Deviance        AIC        BIC       AICc   
## -139.6053   279.2106   313.2106   357.3276   320.7661   
## 
## Variance Components:
## 
##             estim    sqrt  nlvls  fixed      factor 
## sigma^2    0.4239  0.6511     37     no  Article_ID 
## 
## outer factor: Effect_ID (nlvls = 113)
## inner factor: Exp_type  (nlvls = 2)
## 
##             estim    sqrt  k.lvl  fixed     level 
## tau^2.1    0.4860  0.6972     66     no    Demand 
## tau^2.2    1.2253  1.1069     47     no  Resource 
## rho        0.0000                   yes           
## 
## Test for Residual Heterogeneity:
## QE(df = 99) = 6296.4560, p-val < .0001
## 
## Test of Moderators (coefficients 2:14):
## F(df1 = 13, df2 = 99) = 4.1470, p-val < .0001
## 
## Model Results:
## 
##                                                                 estimate 
## intrcpt                                                           1.4995 
## Exp_typeResource                                                 -0.7044 
## Phytoplankton_speciesAlexandrium fundyense                       -1.1080 
## Phytoplankton_speciesAlexandrium minutum                         -0.5231 
## Phytoplankton_speciesAlexandrium tamarense                       -0.6697 
## Phytoplankton_speciesPseudo-nitzschia australis                   1.7253 
## Phytoplankton_speciesPseudo-nitzschia fraudulenta                 0.8915 
## Phytoplankton_speciesPseudo-nitzschia obtusa                      1.6799 
## Phytoplankton_speciesPseudo-nitzschia pungens                     0.5454 
## Phytoplankton_speciesPseudo-nitzschia seriata                     1.4176 
## Exp_typeResource:Phytoplankton_speciesAlexandrium minutum         1.4586 
## Exp_typeResource:Phytoplankton_speciesAlexandrium tamarense       0.5230 
## Exp_typeResource:Phytoplankton_speciesPseudo-nitzschia obtusa    -3.3811 
## Exp_typeResource:Phytoplankton_speciesPseudo-nitzschia seriata   -0.5029 
##                                                                     se     tval 
## intrcpt                                                         0.4473   3.3522 
## Exp_typeResource                                                0.7279  -0.9677 
## Phytoplankton_speciesAlexandrium fundyense                      0.6885  -1.6092 
## Phytoplankton_speciesAlexandrium minutum                        0.5189  -1.0081 
## Phytoplankton_speciesAlexandrium tamarense                      0.8690  -0.7707 
## Phytoplankton_speciesPseudo-nitzschia australis                 0.9477   1.8204 
## Phytoplankton_speciesPseudo-nitzschia fraudulenta               0.9827   0.9072 
## Phytoplankton_speciesPseudo-nitzschia obtusa                    0.9692   1.7333 
## Phytoplankton_speciesPseudo-nitzschia pungens                   0.9811   0.5559 
## Phytoplankton_speciesPseudo-nitzschia seriata                   0.5275   2.6875 
## Exp_typeResource:Phytoplankton_speciesAlexandrium minutum       0.8521   1.7118 
## Exp_typeResource:Phytoplankton_speciesAlexandrium tamarense     1.1232   0.4656 
## Exp_typeResource:Phytoplankton_speciesPseudo-nitzschia obtusa   1.7169  -1.9693 
## Exp_typeResource:Phytoplankton_speciesPseudo-nitzschia seriata  1.6084  -0.3127 
##                                                                 df    pval 
## intrcpt                                                         99  0.0011 
## Exp_typeResource                                                99  0.3355 
## Phytoplankton_speciesAlexandrium fundyense                      99  0.1108 
## Phytoplankton_speciesAlexandrium minutum                        99  0.3159 
## Phytoplankton_speciesAlexandrium tamarense                      99  0.4427 
## Phytoplankton_speciesPseudo-nitzschia australis                 99  0.0717 
## Phytoplankton_speciesPseudo-nitzschia fraudulenta               99  0.3665 
## Phytoplankton_speciesPseudo-nitzschia obtusa                    99  0.0862 
## Phytoplankton_speciesPseudo-nitzschia pungens                   99  0.5795 
## Phytoplankton_speciesPseudo-nitzschia seriata                   99  0.0084 
## Exp_typeResource:Phytoplankton_speciesAlexandrium minutum       99  0.0901 
## Exp_typeResource:Phytoplankton_speciesAlexandrium tamarense     99  0.6425 
## Exp_typeResource:Phytoplankton_speciesPseudo-nitzschia obtusa   99  0.0517 
## Exp_typeResource:Phytoplankton_speciesPseudo-nitzschia seriata  99  0.7552 
##                                                                   ci.lb   ci.ub 
## intrcpt                                                          0.6119  2.3871 
## Exp_typeResource                                                -2.1486  0.7399 
## Phytoplankton_speciesAlexandrium fundyense                      -2.4742  0.2582 
## Phytoplankton_speciesAlexandrium minutum                        -1.5527  0.5065 
## Phytoplankton_speciesAlexandrium tamarense                      -2.3940  1.0546 
## Phytoplankton_speciesPseudo-nitzschia australis                 -0.1552  3.6057 
## Phytoplankton_speciesPseudo-nitzschia fraudulenta               -1.0583  2.8413 
## Phytoplankton_speciesPseudo-nitzschia obtusa                    -0.2432  3.6031 
## Phytoplankton_speciesPseudo-nitzschia pungens                   -1.4013  2.4921 
## Phytoplankton_speciesPseudo-nitzschia seriata                    0.3710  2.4643 
## Exp_typeResource:Phytoplankton_speciesAlexandrium minutum       -0.2321  3.1493 
## Exp_typeResource:Phytoplankton_speciesAlexandrium tamarense     -1.7058  2.7517 
## Exp_typeResource:Phytoplankton_speciesPseudo-nitzschia obtusa   -6.7878  0.0256 
## Exp_typeResource:Phytoplankton_speciesPseudo-nitzschia seriata  -3.6943  2.6885 
##                                                                    
## intrcpt                                                         ** 
## Exp_typeResource                                                   
## Phytoplankton_speciesAlexandrium fundyense                         
## Phytoplankton_speciesAlexandrium minutum                           
## Phytoplankton_speciesAlexandrium tamarense                         
## Phytoplankton_speciesPseudo-nitzschia australis                  . 
## Phytoplankton_speciesPseudo-nitzschia fraudulenta                  
## Phytoplankton_speciesPseudo-nitzschia obtusa                     . 
## Phytoplankton_speciesPseudo-nitzschia pungens                      
## Phytoplankton_speciesPseudo-nitzschia seriata                   ** 
## Exp_typeResource:Phytoplankton_speciesAlexandrium minutum        . 
## Exp_typeResource:Phytoplankton_speciesAlexandrium tamarense        
## Exp_typeResource:Phytoplankton_speciesPseudo-nitzschia obtusa    . 
## Exp_typeResource:Phytoplankton_speciesPseudo-nitzschia seriata     
## 
## ---
## Signif. codes:  0 '***' 0.001 '**' 0.01 '*' 0.05 '.' 0.1 ' ' 1
```

```
i2_ml(mod_14)
```

```
##      I2_Total I2_Article_ID 
##      98.10392      98.10392
```

```
100 - i2_ml(mod_14)[1] # Sampling variance as proportion of total unaccounted variance
```

```
## I2_Total 
## 1.896076
```

```
r2_ml(mod_14)
```

```
##    R2_marginal R2_conditional 
##      0.6415002      0.6415002
```

```
anova(mod_14, btt = c(11:14))
```

```
## 
## Test of Moderators (coefficients 11:14):
## F(df1 = 4, df2 = 99) = 2.7426, p-val = 0.0327
```

```
#orchard_plot(mod_14, 
 #            mod = "Exp_type",
  #           data = data_MA_full,
   #          group = "Article_ID",
    #         by =  "Phytoplankton_species",
     #        xlab = "lnRR (effect size)",
      #       angle = 0,
       #      alpha = 0.4,
       #      cb = FALSE,
      #       k = FALSE,
    #         g = FALSE,
    #         trunk.size = 6,
  #           branch.size = 1.5,
   #          twig.size = 0.7,
    #         colour = TRUE,
     #        legend.pos = "bottom.right") 

orchard_plot(mod_1,
             data = data_MA_full,
             mod = "Phytoplankton_species",
             group = "Exp_type", # Colour of the bubbles in the plot & number in parentheses.
             xlab = "lnRR (effect size)",
             angle = 0,
             alpha = 0.4,
             cb = FALSE,
             k = TRUE,
             g = TRUE,
             trunk.size = 8,
             branch.size = 1.5,
             twig.size = 0.5,
             colour = TRUE,
             fill = TRUE,
             legend.pos = "bottom.right"
             ) +
  
  scale_y_continuous(n.breaks = 10)
```

There is a significant interaction effect between driver and
phytoplankton species (p = 0.033), but an unknown issue with
visualisation prevents us from examining/identifying likely causes
easily. However, it is important to note that the model drops all cases
that does not include information on both driver and species. therefore,
if all effects for a species are contained wihin one level of the driver
moderator (e.g. demand), that species will be dropped as a predictor in
the interaction terms of the model.

#### 5.2.2 Driver x phytoplankton strain

```
mod_15 <- rma.mv(yi = delta_RR,
                V = Var_delta_RR, 
                mods = ~ 1 + (Exp_type * Phytoplankton_strain), 
                method = "REML", 
                test = "t", 
                random = list(~1 | Article_ID,
                              ~1 + Exp_type| Effect_ID),
                rho = 0, 
                struc = "HCS", 
                data = data_MA_full,
                control = list(optimizer="optim", optmethod="Nelder-Mead")
                  )


summary.rma(mod_15)
```

```
## 
## Multivariate Meta-Analysis Model (k = 112; method: REML)
## 
##   logLik  Deviance       AIC       BIC      AICc   
## -85.2984  170.5967  254.5967  350.7960  374.9967   
## 
## Variance Components:
## 
##             estim    sqrt  nlvls  fixed      factor 
## sigma^2    0.4529  0.6730     36     no  Article_ID 
## 
## outer factor: Effect_ID (nlvls = 112)
## inner factor: Exp_type  (nlvls = 2)
## 
##             estim    sqrt  k.lvl  fixed     level 
## tau^2.1    0.3905  0.6249     66     no    Demand 
## tau^2.2    0.4167  0.6455     46     no  Resource 
## rho        0.0000                   yes           
## 
## Test for Residual Heterogeneity:
## QE(df = 73) = 3150.7954, p-val < .0001
## 
## Test of Moderators (coefficients 2:39):
## F(df1 = 38, df2 = 73) = 4.3923, p-val < .0001
## 
## Model Results:
## 
##                                             estimate      se     tval  df 
## intrcpt                                       2.2424  0.7793   2.8774  73 
## Exp_typeResource                              1.0270  0.8040   1.2772  73 
## Phytoplankton_strainA-11c                    -0.9538  1.3829  -0.6897  73 
## Phytoplankton_strainAI420                    -1.5418  1.2214  -1.2624  73 
## Phytoplankton_strainAL-1V                    -1.2785  0.7997  -1.5988  73 
## Phytoplankton_strainAL3T                     -2.7438  1.3851  -1.9810  73 
## Phytoplankton_strainAlex2                    -1.4618  1.0957  -1.3342  73 
## Phytoplankton_strainAlex5                    -2.0900  1.0983  -1.9028  73 
## Phytoplankton_strainAM89BM                   -0.4180  1.2878  -0.3246  73 
## Phytoplankton_strainAMAD16                   -1.3637  1.4762  -0.9237  73 
## Phytoplankton_strainAmKB02                   -2.6640  1.2760  -2.0877  73 
## Phytoplankton_strainATKR-020415              -2.6060  1.2116  -2.1510  73 
## Phytoplankton_strainBF-5                     -0.9282  0.9019  -1.0291  73 
## Phytoplankton_strainCA2                      -4.1754  1.4657  -2.8486  73 
## Phytoplankton_strainClone5                   -1.9434  1.0930  -1.7781  73 
## Phytoplankton_strainCNR AMIA5                -1.2591  0.8893  -1.4158  73 
## Phytoplankton_strainDisko 8                   0.6526  0.7349   0.8880  73 
## Phytoplankton_strainHv5                       0.4646  0.9212   0.5043  73 
## Phytoplankton_strainI1                       -1.5595  1.5649  -0.9966  73 
## Phytoplankton_strainIFR-PAU-010              -2.4180  1.3172  -1.8358  73 
## Phytoplankton_strainKci                      -2.5404  1.3953  -1.8207  73 
## Phytoplankton_strainNo.1                     -1.5423  1.0975  -1.4053  73 
## Phytoplankton_strainNo.9                     -1.0327  1.2156  -0.8495  73 
## Phytoplankton_strainP1D2                     -0.4912  1.3045  -0.3765  73 
## Phytoplankton_strainP3B2                      3.4012  1.4031   2.4241  73 
## Phytoplankton_strainP5G3                      1.3065  0.9716   1.3447  73 
## Phytoplankton_strainP6B3                     -2.2780  1.4136  -1.6115  73 
## Phytoplankton_strainPNfra2                   -0.9517  1.3388  -0.7109  73 
## Phytoplankton_strainPNfra29                  -1.6584  1.4463  -1.1466  73 
## Phytoplankton_strainPNfra31                  -2.3660  1.3240  -1.7870  73 
## Phytoplankton_strainPNpun102                 -1.1059  1.4494  -0.7630  73 
## Phytoplankton_strainPNpun103                 -2.0327  1.4120  -1.4396  73 
## Phytoplankton_strainPNpun47                  -1.9970  1.3417  -1.4885  73 
## Phytoplankton_strainPNpun66                  -2.9267  1.4287  -2.0485  73 
## Phytoplankton_strainPSH1                      0.8501  0.9811   0.8665  73 
## Phytoplankton_strainSKC620                    2.3216  1.2832   1.8092  73 
## Exp_typeResource:Phytoplankton_strainAL-1V    0.2794  0.9131   0.3061  73 
## Exp_typeResource:Phytoplankton_strainAlex2   -1.2598  1.3791  -0.9135  73 
## Exp_typeResource:Phytoplankton_strainBF-5    -2.8754  1.0145  -2.8342  73 
##                                               pval    ci.lb    ci.ub     
## intrcpt                                     0.0053   0.6892   3.7956  ** 
## Exp_typeResource                            0.2056  -0.5755   2.6294     
## Phytoplankton_strainA-11c                   0.4925  -3.7099   1.8022     
## Phytoplankton_strainAI420                   0.2108  -3.9760   0.8924     
## Phytoplankton_strainAL-1V                   0.1142  -2.8722   0.3152     
## Phytoplankton_strainAL3T                    0.0514  -5.5042   0.0166   . 
## Phytoplankton_strainAlex2                   0.1863  -3.6456   0.7219     
## Phytoplankton_strainAlex5                   0.0610  -4.2789   0.0990   . 
## Phytoplankton_strainAM89BM                  0.7464  -2.9845   2.1485     
## Phytoplankton_strainAMAD16                  0.3587  -4.3058   1.5785     
## Phytoplankton_strainAmKB02                  0.0403  -5.2071  -0.1208   * 
## Phytoplankton_strainATKR-020415             0.0348  -5.0207  -0.1914   * 
## Phytoplankton_strainBF-5                    0.3068  -2.7257   0.8693     
## Phytoplankton_strainCA2                     0.0057  -7.0966  -1.2542  ** 
## Phytoplankton_strainClone5                  0.0795  -4.1217   0.2348   . 
## Phytoplankton_strainCNR AMIA5               0.1611  -3.0315   0.5133     
## Phytoplankton_strainDisko 8                 0.3775  -0.8120   2.1172     
## Phytoplankton_strainHv5                     0.6156  -1.3714   2.3006     
## Phytoplankton_strainI1                      0.3223  -4.6782   1.5593     
## Phytoplankton_strainIFR-PAU-010             0.0705  -5.0432   0.2071   . 
## Phytoplankton_strainKci                     0.0727  -5.3211   0.2403   . 
## Phytoplankton_strainNo.1                    0.1642  -3.7296   0.6450     
## Phytoplankton_strainNo.9                    0.3984  -3.4554   1.3900     
## Phytoplankton_strainP1D2                    0.7076  -3.0911   2.1087     
## Phytoplankton_strainP3B2                    0.0178   0.6049   6.1975   * 
## Phytoplankton_strainP5G3                    0.1829  -0.6299   3.2429     
## Phytoplankton_strainP6B3                    0.1114  -5.0953   0.5392     
## Phytoplankton_strainPNfra2                  0.4794  -3.6200   1.7165     
## Phytoplankton_strainPNfra29                 0.2553  -4.5410   1.2241     
## Phytoplankton_strainPNfra31                 0.0781  -5.0047   0.2727   . 
## Phytoplankton_strainPNpun102                0.4479  -3.9946   1.7827     
## Phytoplankton_strainPNpun103                0.1543  -4.8468   0.7815     
## Phytoplankton_strainPNpun47                 0.1409  -4.6709   0.6769     
## Phytoplankton_strainPNpun66                 0.0441  -5.7741  -0.0793   * 
## Phytoplankton_strainPSH1                    0.3891  -1.1052   2.8053     
## Phytoplankton_strainSKC620                  0.0745  -0.2359   4.8791   . 
## Exp_typeResource:Phytoplankton_strainAL-1V  0.7604  -1.5403   2.0992     
## Exp_typeResource:Phytoplankton_strainAlex2  0.3640  -4.0083   1.4887     
## Exp_typeResource:Phytoplankton_strainBF-5   0.0059  -4.8974  -0.8535  ** 
## 
## ---
## Signif. codes:  0 '***' 0.001 '**' 0.01 '*' 0.05 '.' 0.1 ' ' 1
```

```
i2_ml(mod_15)
```

```
##      I2_Total I2_Article_ID 
##      98.05138      98.05138
```

```
100 - i2_ml(mod_15)[1] # Sampling variance as proportion of total unaccounted variance
```

```
## I2_Total 
## 1.948623
```

```
r2_ml(mod_15)
```

```
##    R2_marginal R2_conditional 
##      0.7408778      0.7408778
```

```
anova(mod_15, btt = c(37:39))
```

```
## 
## Test of Moderators (coefficients 37:39):
## F(df1 = 3, df2 = 73) = 5.2414, p-val = 0.0025
```

```
#orchard_plot(mod_15, 
#             mod = "Exp_type",
#             data = data_MA_full,
#             group = "Article_ID",
#             by =  "Phytoplankton_strain",
#             xlab = "lnRR (effect size)",
#             angle = 0,
#             alpha = 0.4,
#             cb = FALSE,
#             k = FALSE,
#             g = FALSE,
#             trunk.size = 6,
#             branch.size = 1.5,
#             twig.size = 0.7,
#             colour = TRUE,
#             legend.pos = "bottom.right") 

orchard_plot(mod_2,
             data = data_MA_full %>%   drop_na(Phytoplankton_strain),
             mod = "Phytoplankton_strain",
             group = "Exp_type", # Colour of the bubbles in the plot & number in parentheses.
             xlab = "lnRR (effect size)",
             angle = 0,
             alpha = 0.4,
             cb = FALSE,
             k = TRUE,
             g = TRUE,
             trunk.size = 8,
             branch.size = 1.5,
             twig.size = 0.5,
             colour = TRUE,
             fill = TRUE,
             legend.pos = "bottom.right"
             ) +
  
  scale_y_continuous(n.breaks = 10)
```

There is a significant interaction effect between driver and
phytoplankton strain (p = 0.0025), but an unknown issue with
visualisation prevents us from examining/identifying likely causes
easily. Also, many of the strains are only represented by one to four
effects, making inference very uncertain. We will attempt to capture the
effects of phytoplankton species & strain in the driver \*
phytoplankton genus interaction model instead.

#### 5.2.3 Driver x phytoplankton genus (*Alexandrium* & *Pseudo-nitzschia*)

```
mod_16 <- rma.mv(yi = delta_RR,
                V = Var_delta_RR, 
                mods = ~ 1 + (Exp_type * Phytoplankton_group), 
                method = "REML", 
                test = "t", 
                random = list(~1 | Article_ID,
                              ~1 + Exp_type| Effect_ID),
                rho = 0, 
                struc = "HCS", 
                data = data_MA_full,
                control = list(optimizer="optim", optmethod="Nelder-Mead")
                  )

summary.rma(mod_16)
```

```
## 
## Multivariate Meta-Analysis Model (k = 113; method: REML)
## 
##    logLik   Deviance        AIC        BIC       AICc   
## -158.1678   316.3356   330.3356   349.1750   331.4445   
## 
## Variance Components:
## 
##             estim    sqrt  nlvls  fixed      factor 
## sigma^2    0.3596  0.5997     37     no  Article_ID 
## 
## outer factor: Effect_ID (nlvls = 113)
## inner factor: Exp_type  (nlvls = 2)
## 
##             estim    sqrt  k.lvl  fixed     level 
## tau^2.1    0.4948  0.7034     66     no    Demand 
## tau^2.2    1.4503  1.2043     47     no  Resource 
## rho        0.0000                   yes           
## 
## Test for Residual Heterogeneity:
## QE(df = 109) = 9761.9205, p-val < .0001
## 
## Test of Moderators (coefficients 2:4):
## F(df1 = 3, df2 = 109) = 13.3954, p-val < .0001
## 
## Model Results:
## 
##                                                       estimate      se     tval 
## intrcpt                                                 0.9824  0.1909   5.1453 
## Exp_typeResource                                        0.1553  0.3185   0.4874 
## Phytoplankton_groupPseudo-nitzschia                     1.9115  0.3074   6.2188 
## Exp_typeResource:Phytoplankton_groupPseudo-nitzschia   -1.4529  0.6216  -2.3374 
##                                                        df    pval    ci.lb 
## intrcpt                                               109  <.0001   0.6040 
## Exp_typeResource                                      109  0.6269  -0.4761 
## Phytoplankton_groupPseudo-nitzschia                   109  <.0001   1.3023 
## Exp_typeResource:Phytoplankton_groupPseudo-nitzschia  109  0.0212  -2.6848 
##                                                         ci.ub      
## intrcpt                                                1.3608  *** 
## Exp_typeResource                                       0.7866      
## Phytoplankton_groupPseudo-nitzschia                    2.5208  *** 
## Exp_typeResource:Phytoplankton_groupPseudo-nitzschia  -0.2209    * 
## 
## ---
## Signif. codes:  0 '***' 0.001 '**' 0.01 '*' 0.05 '.' 0.1 ' ' 1
```

```
i2_ml(mod_16)
```

```
##      I2_Total I2_Article_ID 
##      97.77228      97.77228
```

```
100 - i2_ml(mod_16)[1] # Sampling variance as proportion of total unaccounted variance
```

```
## I2_Total 
## 2.227724
```

```
r2_ml(mod_16)
```

```
##    R2_marginal R2_conditional 
##      0.5771034      0.5771034
```

```
#Test interaction effect globally
anova(mod_16, btt = 4)
```

```
## 
## Test of Moderators (coefficient 4):
## F(df1 = 1, df2 = 109) = 5.4635, p-val = 0.0212
```

```
## Set up interaction model for post-hoc analysis
mod_16_interaction <- rma.mv(yi = delta_RR,
                V = Var_delta_RR, 
                mods = ~ 0 + Phytoplankton_group:Exp_type:Phytoplankton_group, 
                method = "REML", 
                test = "t", 
                random = list(~1 | Article_ID,
                              ~1 + Exp_type| Effect_ID),
                rho = 0, 
                struc = "HCS", 
                data = data_MA_full,
                control = list(optimizer="optim", optmethod="Nelder-Mead")
                  )

# conservative post-hoc results (with Holms adjustment)
# (1 = Alex_Demand, 2 = Pseudo_Demand, 3 = Alex_Resource, 4 = Pseudo_resource)
summary(glht(mod_16_interaction, linfct=cbind(contrMat(rep(1,4), type="Tukey"))), test=adjusted("holm"))
```

```
## 
##   Simultaneous Tests for General Linear Hypotheses
## 
## Fit: rma.mv(yi = delta_RR, V = Var_delta_RR, mods = ~0 + Phytoplankton_group:Exp_type:Phytoplankton_group, 
##     random = list(~1 | Article_ID, ~1 + Exp_type | Effect_ID), 
##     struct = "HCS", data = data_MA_full, method = "REML", test = "t", 
##     rho = 0, control = list(optimizer = "optim", optmethod = "Nelder-Mead"))
## 
## Linear Hypotheses:
##            Estimate Std. Error z value Pr(>|z|)    
## 2 - 1 == 0   1.9115     0.3074   6.219 3.01e-09 ***
## 3 - 1 == 0   0.1553     0.3185   0.487   0.8038    
## 4 - 1 == 0   0.6139     0.5115   1.200   0.6902    
## 3 - 2 == 0  -1.7563     0.3792  -4.631 1.82e-05 ***
## 4 - 2 == 0  -1.2976     0.5445  -2.383   0.0687 .  
## 4 - 3 == 0   0.4586     0.5472   0.838   0.8038    
## ---
## Signif. codes:  0 '***' 0.001 '**' 0.01 '*' 0.05 '.' 0.1 ' ' 1
## (Adjusted p values reported -- holm method)
```

```
## Extract group-wise results
res_mod_16 <-
  orchaRd::mod_results(mod_16,
                       mod = "Exp_type",
                       group = "Phytoplankton_group",
                       by = "Phytoplankton_group",
                       data = data_MA_full)$mod_table %>%  
    rename(Driver = name,
           Genus = condition)

res_mod_16
```

```
##     Driver            Genus  estimate   lowerCL  upperCL    lowerPR  upperPR
## 1   Demand      Alexandrium 0.9823805 0.6039667 1.360794 -0.8883261 2.853087
## 2 Resource      Alexandrium 1.1376476 0.5978342 1.677461 -1.5828608 3.858156
## 3   Demand Pseudo-nitzschia 2.8939178 2.3646531 3.423183  0.9869655 4.800870
## 4 Resource Pseudo-nitzschia 1.5962925 0.6557508 2.536834 -1.2311420 4.423727
```

```
#Make orchard plot
orchard_plot(mod_16, 
             mod = "Exp_type",
             data = data_MA_full,
             group = "Article_ID",
             by =  "Phytoplankton_group",
             xlab = "lnRR (effect size)",
             angle = 0,
             alpha = 0.4,
             cb = FALSE,
             k = TRUE,
             g = TRUE,
             trunk.size = 6,
             branch.size = 1.5,
             twig.size = 0.7,
             colour = TRUE,
             legend.pos = "bottom.right") +
  
  scale_y_continuous(n.breaks = 10)
```

There is a significant interaction effect between driver and
phytoplankton genus (p = 0.0212). *Pseudo-nitzschia* demand
effects differ from *Alexandrium* demand (p = 3.01e-09) and
resource (1.82e-05) effects. Both *Alexandrium* and
*Pseudo-nitzschia* toxicity are significantly induced by
increased N:P ratio and grazing pressure. There is no difference between
the genera under increased N:P ratio, but there is a trend towards
*Pseudo-nitzschia* increasing in toxicity more than
*Alexandrium*. Potential grazing also induces toxicity in both
genera, but significantly more in *Pseudo-nitzschia*. The stark
difference between the genera within each driver is the cause of the
interaction effect.

#### 5.2.4 Driver x culture type

```
mod_17 <- rma.mv(yi = delta_RR,
                V = Var_delta_RR, 
                mods = ~ 1 + (Exp_type * Culture_type), 
                method = "REML", 
                test = "t", 
                random = list(~1 | Article_ID,
                              ~1 + Exp_type| Effect_ID),
                rho = 0, 
                struc = "HCS", 
                data = data_MA_full,
                control = list(optimizer="optim", optmethod="Nelder-Mead"))

summary.rma(mod_17)
```

```
## 
## Multivariate Meta-Analysis Model (k = 113; method: REML)
## 
##    logLik   Deviance        AIC        BIC       AICc   
## -170.5966   341.1931   359.1931   383.2486   361.0488   
## 
## Variance Components:
## 
##             estim    sqrt  nlvls  fixed      factor 
## sigma^2    0.5193  0.7207     37     no  Article_ID 
## 
## outer factor: Effect_ID (nlvls = 113)
## inner factor: Exp_type  (nlvls = 2)
## 
##             estim    sqrt  k.lvl  fixed     level 
## tau^2.1    0.8689  0.9321     66     no    Demand 
## tau^2.2    1.3649  1.1683     47     no  Resource 
## rho        0.0000                   yes           
## 
## Test for Residual Heterogeneity:
## QE(df = 107) = 15600.1690, p-val < .0001
## 
## Test of Moderators (coefficients 2:6):
## F(df1 = 5, df2 = 107) = 0.5487, p-val = 0.7390
## 
## Model Results:
## 
##                                               estimate      se     tval   df 
## intrcpt                                         1.5847  0.2090   7.5820  107 
## Exp_typeResource                               -0.4626  0.3391  -1.3644  107 
## Culture_typeContinuous                          0.0468  0.9266   0.0505  107 
## Culture_typeSemi-continuous                     0.0498  1.1986   0.0415  107 
## Exp_typeResource:Culture_typeContinuous         0.7362  1.4185   0.5190  107 
## Exp_typeResource:Culture_typeSemi-continuous    0.8021  1.3672   0.5866  107 
##                                                 pval    ci.lb   ci.ub      
## intrcpt                                       <.0001   1.1704  1.9991  *** 
## Exp_typeResource                              0.1753  -1.1347  0.2095      
## Culture_typeContinuous                        0.9598  -1.7901  1.8837      
## Culture_typeSemi-continuous                   0.9669  -2.3262  2.4258      
## Exp_typeResource:Culture_typeContinuous       0.6049  -2.0759  3.5483      
## Exp_typeResource:Culture_typeSemi-continuous  0.5587  -1.9083  3.5125      
## 
## ---
## Signif. codes:  0 '***' 0.001 '**' 0.01 '*' 0.05 '.' 0.1 ' ' 1
```

```
i2_ml(mod_17)
```

```
##      I2_Total I2_Article_ID 
##      98.44681      98.44681
```

```
100 - i2_ml(mod_17)[1] # Sampling variance as proportion of total unaccounted variance
```

```
## I2_Total 
## 1.553187
```

```
r2_ml(mod_17)
```

```
##    R2_marginal R2_conditional 
##      0.1171559      0.1171559
```

```
anova(mod_17, btt = c(5:6))
```

```
## 
## Test of Moderators (coefficients 5:6):
## F(df1 = 2, df2 = 107) = 0.2897, p-val = 0.7491
```

```
orchard_plot(mod_17, 
             mod = "Exp_type",
             data = data_MA_full,
             group = "Article_ID",
             by =  "Culture_type",
             xlab = "lnRR (effect size)",
             angle = 0,
             alpha = 0.4,
             cb = FALSE,
             k = TRUE,
             g = TRUE,
             trunk.size = 6,
             branch.size = 1.5,
             twig.size = 0.7,
             colour = TRUE,
             legend.pos = "bottom.right") +
  
  scale_y_continuous(n.breaks = 10)
```

There is no interaction effect between driver and culture type (p =
0.75.

#### 5.2.5 Driver x experiment medium

```
mod_18 <- rma.mv(yi = delta_RR,
                V = Var_delta_RR, 
                mods = ~ 1 + (Exp_type * Experiment_medium), 
                method = "REML", 
                test = "t", 
                random = list(~1 | Article_ID,
                              ~1 + Exp_type| Effect_ID),
                rho = 0, 
                struc = "HCS", 
                data = data_MA_full,
                control = list(optimizer="optim", optmethod="Nelder-Mead")
                  )

summary.rma(mod_18)
```

```
## 
## Multivariate Meta-Analysis Model (k = 113; method: REML)
## 
##    logLik   Deviance        AIC        BIC       AICc   
## -141.2091   282.4181   320.4181   369.3377   330.2883   
## 
## Variance Components:
## 
##             estim    sqrt  nlvls  fixed      factor 
## sigma^2    0.3808  0.6171     37     no  Article_ID 
## 
## outer factor: Effect_ID (nlvls = 113)
## inner factor: Exp_type  (nlvls = 2)
## 
##             estim    sqrt  k.lvl  fixed     level 
## tau^2.1    0.5310  0.7287     66     no    Demand 
## tau^2.2    1.3722  1.1714     47     no  Resource 
## rho        0.0000                   yes           
## 
## Test for Residual Heterogeneity:
## QE(df = 97) = 8665.8883, p-val < .0001
## 
## Test of Moderators (coefficients 2:16):
## F(df1 = 15, df2 = 97) = 3.2205, p-val = 0.0002
## 
## Model Results:
## 
##                                                     estimate      se     tval 
## intrcpt                                               1.1578  0.9722   1.1909 
## Exp_typeResource                                      0.5913  1.3991   0.4227 
## Experiment_mediumB1 (reduced nitrogen, 80 µM NO3-)    0.3449  0.9488   0.3635 
## Experiment_mediumCustom                              -0.9641  1.2172  -0.7921 
## Experiment_mediumDiluted K                           -0.2817  1.3669  -0.2061 
## Experiment_mediumF/2                                  0.0371  1.0456   0.0355 
## Experiment_mediumF/4                                  0.2524  1.1519   0.2191 
## Experiment_mediumFSW                                  1.0383  1.2953   0.8016 
## Experiment_mediumK                                    0.3876  1.0962   0.3536 
## Experiment_mediumK/10                                -0.2703  1.0410  -0.2596 
## Experiment_mediumK/10 w 25% phosphate                -0.4038  1.2277  -0.3289 
## Experiment_mediumL1                                   2.4499  1.0573   2.3171 
## Experiment_mediumL1 + NH4                             0.7951  1.3776   0.5771 
## Experiment_mediumL1/10 + NH4                          2.1349  1.2651   1.6876 
## Experiment_mediumL1/4 + NH4                           0.7205  1.0968   0.6569 
## Exp_typeResource:Experiment_mediumCustom              0.4500  1.5845   0.2840 
##                                                     df    pval    ci.lb   ci.ub 
## intrcpt                                             97  0.2366  -0.7717  3.0872 
## Exp_typeResource                                    97  0.6735  -2.1855  3.3681 
## Experiment_mediumB1 (reduced nitrogen, 80 µM NO3-)  97  0.7170  -1.5382  2.2280 
## Experiment_mediumCustom                             97  0.4302  -3.3799  1.4516 
## Experiment_mediumDiluted K                          97  0.8372  -2.9945  2.4311 
## Experiment_mediumF/2                                97  0.9717  -2.0381  2.1124 
## Experiment_mediumF/4                                97  0.8270  -2.0338  2.5385 
## Experiment_mediumFSW                                97  0.4248  -1.5325  3.6090 
## Experiment_mediumK                                  97  0.7244  -1.7881  2.5634 
## Experiment_mediumK/10                               97  0.7957  -2.3365  1.7959 
## Experiment_mediumK/10 w 25% phosphate               97  0.7429  -2.8404  2.0328 
## Experiment_mediumL1                                 97  0.0226   0.3514  4.5485 
## Experiment_mediumL1 + NH4                           97  0.5652  -1.9392  3.5293 
## Experiment_mediumL1/10 + NH4                        97  0.0947  -0.3759  4.6457 
## Experiment_mediumL1/4 + NH4                         97  0.5128  -1.4563  2.8973 
## Exp_typeResource:Experiment_mediumCustom            97  0.7770  -2.6948  3.5947 
##                                                       
## intrcpt                                               
## Exp_typeResource                                      
## Experiment_mediumB1 (reduced nitrogen, 80 µM NO3-)    
## Experiment_mediumCustom                               
## Experiment_mediumDiluted K                            
## Experiment_mediumF/2                                  
## Experiment_mediumF/4                                  
## Experiment_mediumFSW                                  
## Experiment_mediumK                                    
## Experiment_mediumK/10                                 
## Experiment_mediumK/10 w 25% phosphate                 
## Experiment_mediumL1                                 * 
## Experiment_mediumL1 + NH4                             
## Experiment_mediumL1/10 + NH4                        . 
## Experiment_mediumL1/4 + NH4                           
## Exp_typeResource:Experiment_mediumCustom              
## 
## ---
## Signif. codes:  0 '***' 0.001 '**' 0.01 '*' 0.05 '.' 0.1 ' ' 1
```

```
i2_ml(mod_18)
```

```
##      I2_Total I2_Article_ID 
##      97.89381      97.89381
```

```
100 - i2_ml(mod_18)[1] # Sampling variance as proportion of total unaccounted variance
```

```
## I2_Total 
## 2.106192
```

```
r2_ml(mod_18)
```

```
##    R2_marginal R2_conditional 
##      0.5821369      0.5821369
```

```
#orchard_plot(mod_18, 
#             mod = "Exp_type",
#             data = data_MA_full,
#             group = "Article_ID",
#             by =  "Experiment_medium",
#             xlab = "lnRR (effect size)",
#             angle = 0,
#             alpha = 0.4,
#             cb = FALSE,
#             k = TRUE,
#             g = TRUE,
#             trunk.size = 6,
#             branch.size = 1.5,
#             twig.size = 0.7,
#             colour = TRUE,
#             legend.pos = "bottom.right")
```

There are no interaction effects between driver and experiment medium
(p = 0.77).

#### 5.2.6 Driver x illumination

```
mod_19 <- rma.mv(yi = delta_RR,
                V = Var_delta_RR, 
                mods = ~ 0 + (Exp_type * Light), 
                method = "REML", 
                test = "t", 
                random = list(~1 | Article_ID,
                              ~1 + Exp_type| Effect_ID),
                rho = 0, 
                struc = "HCS", 
                data = data_MA_full,
                control = list(optimizer="optim", optmethod="Nelder-Mead"))

summary.rma(mod_19)
```

```
## 
## Multivariate Meta-Analysis Model (k = 100; method: REML)
## 
##    logLik   Deviance        AIC        BIC       AICc   
## -145.4076   290.8151   304.8151   322.7655   306.0878   
## 
## Variance Components:
## 
##             estim    sqrt  nlvls  fixed      factor 
## sigma^2    0.2172  0.4661     33     no  Article_ID 
## 
## outer factor: Effect_ID (nlvls = 100)
## inner factor: Exp_type  (nlvls = 2)
## 
##             estim    sqrt  k.lvl  fixed     level 
## tau^2.1    0.8114  0.9008     55     no    Demand 
## tau^2.2    1.2739  1.1287     45     no  Resource 
## rho        0.0000                   yes           
## 
## Test for Residual Heterogeneity:
## QE(df = 96) = 8681.9952, p-val < .0001
## 
## Test of Moderators (coefficients 1:4):
## F(df1 = 4, df2 = 96) = 35.9436, p-val < .0001
## 
## Model Results:
## 
##                         estimate      se     tval  df    pval    ci.lb    ci.ub 
## Exp_typeDemand            4.1777  0.5858   7.1312  96  <.0001   3.0149   5.3406 
## Exp_typeResource          1.9097  0.3782   5.0490  96  <.0001   1.1589   2.6605 
## Light                    -0.0224  0.0049  -4.6005  96  <.0001  -0.0320  -0.0127 
## Exp_typeResource:Light    0.0187  0.0052   3.5764  96  0.0005   0.0083   0.0291 
##                             
## Exp_typeDemand          *** 
## Exp_typeResource        *** 
## Light                   *** 
## Exp_typeResource:Light  *** 
## 
## ---
## Signif. codes:  0 '***' 0.001 '**' 0.01 '*' 0.05 '.' 0.1 ' ' 1
```

```
i2_ml(mod_19)
```

```
##      I2_Total I2_Article_ID 
##      96.48949      96.48949
```

```
100 - i2_ml(mod_19)[1] # Sampling variance as proportion of total unaccounted variance
```

```
## I2_Total 
## 3.510506
```

```
r2_ml(mod_19)
```

```
##    R2_marginal R2_conditional 
##       0.643152       0.643152
```

```
bubble_plot(mod_19, 
            mod = "Light",
            data = data_MA_full,
            group = "Article_ID",
            cb = FALSE,
            k = TRUE,
            g = TRUE,
            legend.pos = "bottom.right",
            by = "Exp_type",
            xlab = "Illumination (µmol m^-2 s^-1)"
            )+
  
  scale_x_continuous(n.breaks = 7) +
     scale_fill_manual(breaks = c("Resource", "Demand"),
                     values = c("#1B9E77", "#D95F02"))
```

There is an significant interaction effect between driver and light
intensity (p = 0.0005). Although light negatively affects the increase
of toxicity under increased N:P ratio conditions slightly, the effect is
much stronger under pressure from grazers or their chemical cues.

#### 5.2.7 Driver x ligh-dark cycle

```
mod_20 <- rma.mv(yi = delta_RR,
                V = Var_delta_RR, 
                mods = ~ 1 + (Exp_type * LD_cycle), 
                method = "REML", 
                test = "t", 
                random = list(~1 | Article_ID,
                              ~1 + Exp_type| Effect_ID),
                rho = 0, 
                struc = "HCS", 
                data = data_MA_full,
                control = list(optimizer="optim", optmethod="Nelder-Mead"))

summary.rma(mod_20)
```

```
## 
## Multivariate Meta-Analysis Model (k = 110; method: REML)
## 
##    logLik   Deviance        AIC        BIC       AICc   
## -161.6683   323.3366   337.3366   355.9807   338.4795   
## 
## Variance Components:
## 
##             estim    sqrt  nlvls  fixed      factor 
## sigma^2    0.6344  0.7965     35     no  Article_ID 
## 
## outer factor: Effect_ID (nlvls = 110)
## inner factor: Exp_type  (nlvls = 2)
## 
##             estim    sqrt  k.lvl  fixed     level 
## tau^2.1    0.5848  0.7647     66     no    Demand 
## tau^2.2    1.3764  1.1732     44     no  Resource 
## rho        0.0000                   yes           
## 
## Test for Residual Heterogeneity:
## QE(df = 106) = 10630.3233, p-val < .0001
## 
## Test of Moderators (coefficients 2:4):
## F(df1 = 3, df2 = 106) = 5.6908, p-val = 0.0012
## 
## Model Results:
## 
##                            estimate      se     tval   df    pval    ci.lb 
## intrcpt                     -1.0509  0.7066  -1.4874  106  0.1399  -2.4518 
## Exp_typeResource             0.0587  2.0529   0.0286  106  0.9772  -4.0114 
## LD_cycle                     0.1867  0.0477   3.9149  106  0.0002   0.0922 
## Exp_typeResource:LD_cycle   -0.0094  0.1534  -0.0612  106  0.9513  -0.3135 
##                             ci.ub      
## intrcpt                    0.3499      
## Exp_typeResource           4.1289      
## LD_cycle                   0.2813  *** 
## Exp_typeResource:LD_cycle  0.2947      
## 
## ---
## Signif. codes:  0 '***' 0.001 '**' 0.01 '*' 0.05 '.' 0.1 ' ' 1
```

```
i2_ml(mod_20)
```

```
##      I2_Total I2_Article_ID 
##      98.62162      98.62162
```

```
100 - i2_ml(mod_20)[1] # Sampling variance as proportion of total unaccounted variance
```

```
## I2_Total 
## 1.378377
```

```
r2_ml(mod_20)
```

```
##    R2_marginal R2_conditional 
##       0.250405       0.250405
```

```
bubble_plot(mod_20, 
            mod = "LD_cycle",
            data = data_MA_full,
            group = "Article_ID",
            cb = TRUE,
            k = TRUE,
            g = TRUE,
            legend.pos = "bottom.right",
            by = "Exp_type",
            xlab = "Light hours day^-1"
            )+
  
  scale_x_continuous(n.breaks = 7)+
     scale_fill_manual(breaks = c("Resource", "Demand"),
                     values = c("#1B9E77", "#D95F02"))
```

There is no interaction effect between driver and light-dark cycle (p
= 0.95), both N:P ratio increase and grazer pressure predicts an
increase in effect size with almost identical magnitudes.

#### 5.2.8 Driver x temperature

```
mod_21 <- rma.mv(yi = delta_RR,
                V = Var_delta_RR, 
                mods = ~ 1 + (Exp_type * Temperature), 
                method = "REML", 
                test = "t",
                random = list(~1 | Article_ID,
                              ~1 + Exp_type| Effect_ID),
                rho = 0, 
                struc = "HCS", 
                data = data_MA_full,
                control = list(optimizer="optim", optmethod="Nelder-Mead")
                  )

summary.rma(mod_21)
```

```
## 
## Multivariate Meta-Analysis Model (k = 113; method: REML)
## 
##    logLik   Deviance        AIC        BIC       AICc   
## -161.7752   323.5505   337.5505   356.3899   338.6594   
## 
## Variance Components:
## 
##             estim    sqrt  nlvls  fixed      factor 
## sigma^2    0.3687  0.6072     37     no  Article_ID 
## 
## outer factor: Effect_ID (nlvls = 113)
## inner factor: Exp_type  (nlvls = 2)
## 
##             estim    sqrt  k.lvl  fixed     level 
## tau^2.1    0.5650  0.7516     66     no    Demand 
## tau^2.2    1.4479  1.2033     47     no  Resource 
## rho        0.0000                   yes           
## 
## Test for Residual Heterogeneity:
## QE(df = 109) = 10366.6820, p-val < .0001
## 
## Test of Moderators (coefficients 2:4):
## F(df1 = 3, df2 = 109) = 9.9750, p-val < .0001
## 
## Model Results:
## 
##                               estimate      se     tval   df    pval    ci.lb 
## intrcpt                         3.3467  0.3683   9.0875  109  <.0001   2.6168 
## Exp_typeResource               -2.0013  1.1281  -1.7739  109  0.0789  -4.2372 
## Temperature                    -0.1318  0.0245  -5.3787  109  <.0001  -0.1803 
## Exp_typeResource:Temperature    0.1264  0.0641   1.9725  109  0.0511  -0.0006 
##                                 ci.ub      
## intrcpt                        4.0766  *** 
## Exp_typeResource               0.2347    . 
## Temperature                   -0.0832  *** 
## Exp_typeResource:Temperature   0.2534    . 
## 
## ---
## Signif. codes:  0 '***' 0.001 '**' 0.01 '*' 0.05 '.' 0.1 ' ' 1
```

```
i2_ml(mod_21)
```

```
##      I2_Total I2_Article_ID 
##      97.82573      97.82573
```

```
100 - i2_ml(mod_21)[1] # Sampling variance as proportion of total unaccounted variance
```

```
## I2_Total 
## 2.174268
```

```
r2_ml(mod_21)
```

```
##    R2_marginal R2_conditional 
##      0.5301961      0.5301961
```

```
bubble_plot(mod_21, 
            mod = "Temperature",
            data = data_MA_full,
            group = "Article_ID",
            cb = TRUE,
            k = TRUE,
            g = TRUE,
            legend.pos = "bottom.right",
            by = "Exp_type",
            xlab = "Temperature (°C)"
            )+
  
  scale_x_continuous(n.breaks = 7)+
     scale_fill_manual(breaks = c("Resource", "Demand"),
                     values = c("#1B9E77", "#D95F02"))
```

There is almost a significant interaction effect between driver and
temperature (p = 0.0511). Effects due to resource availability are
unaffected by temperature, but effects due to grazer pressure are
predicted to be negatively affected by increased temperature. However,
there is considerable differences in temperature ranges used between the
two drivers.

#### 5.2.9 Driver x experimental time

```
mod_22 <- rma.mv(yi = delta_RR,
                V = Var_delta_RR, 
                mods = ~ 1 + (Exp_type * Hours), 
                method = "REML", 
                test = "t", 
                random = list(~1 | Article_ID,
                              ~1 + Exp_type| Effect_ID),
                rho = 0, 
                struc = "HCS", 
                data = data_MA_full,
                control = list(optimizer="optim", optmethod="Nelder-Mead"))

summary.rma(mod_22)
```

```
## 
## Multivariate Meta-Analysis Model (k = 104; method: REML)
## 
##    logLik   Deviance        AIC        BIC       AICc   
## -159.1337   318.2673   332.2673   350.5035   333.4847   
## 
## Variance Components:
## 
##             estim    sqrt  nlvls  fixed      factor 
## sigma^2    0.5371  0.7329     36     no  Article_ID 
## 
## outer factor: Effect_ID (nlvls = 104)
## inner factor: Exp_type  (nlvls = 2)
## 
##             estim    sqrt  k.lvl  fixed     level 
## tau^2.1    0.7951  0.8917     58     no    Demand 
## tau^2.2    1.4106  1.1877     46     no  Resource 
## rho        0.0000                   yes           
## 
## Test for Residual Heterogeneity:
## QE(df = 100) = 13780.5764, p-val < .0001
## 
## Test of Moderators (coefficients 2:4):
## F(df1 = 3, df2 = 100) = 0.7422, p-val = 0.5293
## 
## Model Results:
## 
##                         estimate      se     tval   df    pval    ci.lb   ci.ub 
## intrcpt                   1.5152  0.3409   4.4442  100  <.0001   0.8388  2.1916 
## Exp_typeResource         -0.3337  0.4298  -0.7764  100  0.4393  -1.1864  0.5190 
## Hours                     0.0012  0.0027   0.4370  100  0.6631  -0.0042  0.0066 
## Exp_typeResource:Hours   -0.0010  0.0027  -0.3679  100  0.7137  -0.0065  0.0044 
##                             
## intrcpt                 *** 
## Exp_typeResource            
## Hours                       
## Exp_typeResource:Hours      
## 
## ---
## Signif. codes:  0 '***' 0.001 '**' 0.01 '*' 0.05 '.' 0.1 ' ' 1
```

```
i2_ml(mod_22)
```

```
##      I2_Total I2_Article_ID 
##      98.54509      98.54509
```

```
100 - i2_ml(mod_22)[1] # Sampling variance as proportion of total unaccounted variance
```

```
## I2_Total 
## 1.454907
```

```
r2_ml(mod_22)
```

```
##    R2_marginal R2_conditional 
##     0.08954505     0.08954505
```

```
bubble_plot(mod_22, 
            mod = "Hours",
            data = data_MA_full,
            group = "Article_ID",
            cb = TRUE,
            k = TRUE,
            g = TRUE,
            legend.pos = "bottom.right",
            by = "Exp_type",
            xlab = "Experiment duration (hours)")+
  
  scale_x_continuous(n.breaks = 7)+
     scale_fill_manual(breaks = c("Resource", "Demand"),
                     values = c("#1B9E77", "#D95F02"))
```

```
#Removing extreme duration values (~5750 hours)
mod_22 <- rma.mv(yi = delta_RR,
                V = Var_delta_RR, 
                mods = ~ 1 + (Exp_type * Hours), 
                method = "REML", 
                test = "t", 
                random = list(~1 | Article_ID,
                              ~1 + Exp_type| Effect_ID),
                rho = 0, 
                struc = "HCS", 
                data = data_MA_full %>%  filter(Hours<1000),
                control = list(optimizer="optim", optmethod="Nelder-Mead"))
                

summary.rma(mod_22)
```

```
## 
## Multivariate Meta-Analysis Model (k = 102; method: REML)
## 
##    logLik   Deviance        AIC        BIC       AICc   
## -156.1785   312.3570   326.3570   344.4517   327.6014   
## 
## Variance Components:
## 
##             estim    sqrt  nlvls  fixed      factor 
## sigma^2    0.5504  0.7419     35     no  Article_ID 
## 
## outer factor: Effect_ID (nlvls = 102)
## inner factor: Exp_type  (nlvls = 2)
## 
##             estim    sqrt  k.lvl  fixed     level 
## tau^2.1    0.7915  0.8897     58     no    Demand 
## tau^2.2    1.4478  1.2032     44     no  Resource 
## rho        0.0000                   yes           
## 
## Test for Residual Heterogeneity:
## QE(df = 98) = 13211.5323, p-val < .0001
## 
## Test of Moderators (coefficients 2:4):
## F(df1 = 3, df2 = 98) = 0.6783, p-val = 0.5674
## 
## Model Results:
## 
##                         estimate      se     tval  df    pval    ci.lb   ci.ub 
## intrcpt                   1.5306  0.3436   4.4547  98  <.0001   0.8487  2.2124 
## Exp_typeResource         -0.0177  0.6654  -0.0265  98  0.9789  -1.3382  1.3029 
## Hours                     0.0012  0.0028   0.4202  98  0.6753  -0.0043  0.0066 
## Exp_typeResource:Hours   -0.0025  0.0036  -0.6838  98  0.4957  -0.0097  0.0047 
##                             
## intrcpt                 *** 
## Exp_typeResource            
## Hours                       
## Exp_typeResource:Hours      
## 
## ---
## Signif. codes:  0 '***' 0.001 '**' 0.01 '*' 0.05 '.' 0.1 ' ' 1
```

```
i2_ml(mod_22)
```

```
##      I2_Total I2_Article_ID 
##      98.53594      98.53594
```

```
100 - i2_ml(mod_22)[1]
```

```
## I2_Total 
## 1.464063
```

```
r2_ml(mod_22)
```

```
##    R2_marginal R2_conditional 
##     0.09437439     0.09437439
```

```
bubble_plot(mod_22, 
            mod = "Hours",
            data = data_MA_full %>%  filter(Hours<1000),
            group = "Article_ID",
            cb = TRUE,
            k = TRUE,
            g = TRUE,
            legend.pos = "bottom.right",
            by = "Exp_type",
            xlab = "Experiment duration (hours)") +
  
  scale_x_continuous(n.breaks = 7)+
     scale_fill_manual(breaks = c("Resource", "Demand"),
                     values = c("#1B9E77", "#D95F02"))
```

There is no interaction effect between driver and experiment
duration, regardless of extreme values being included (p = 0.71) or
excluded (0.495), both drivers are essentially unaffected by experiment
duration.

#### 5.2.10 Driver x publication year

```
mod_23 <- rma.mv(yi = delta_RR,
                V = Var_delta_RR, 
                mods = ~ 1 + (Exp_type * Year), 
                method = "REML", 
                test = "t", 
                random = list(~1 | Article_ID,
                              ~1 + Exp_type| Effect_ID),
                rho = 0, 
                struc = "HCS", 
                data = data_MA_full, #%>%   filter(Exp_type == "Resource"),
                control = list(optimizer="optim", optmethod="Nelder-Mead"))

summary.rma(mod_23)
```

```
## 
## Multivariate Meta-Analysis Model (k = 113; method: REML)
## 
##    logLik   Deviance        AIC        BIC       AICc   
## -169.2660   338.5319   352.5319   371.3713   353.6408   
## 
## Variance Components:
## 
##             estim    sqrt  nlvls  fixed      factor 
## sigma^2    0.4246  0.6516     37     no  Article_ID 
## 
## outer factor: Effect_ID (nlvls = 113)
## inner factor: Exp_type  (nlvls = 2)
## 
##             estim    sqrt  k.lvl  fixed     level 
## tau^2.1    0.7951  0.8917     66     no    Demand 
## tau^2.2    1.3197  1.1488     47     no  Resource 
## rho        0.0000                   yes           
## 
## Test for Residual Heterogeneity:
## QE(df = 109) = 13780.5476, p-val < .0001
## 
## Test of Moderators (coefficients 2:4):
## F(df1 = 3, df2 = 109) = 3.3896, p-val = 0.0207
## 
## Model Results:
## 
##                         estimate        se     tval   df    pval      ci.lb 
## intrcpt                -202.5494   76.3493  -2.6529  109  0.0092  -353.8712 
## Exp_typeResource        297.9596  103.8317   2.8696  109  0.0049    92.1685 
## Year                      0.1013    0.0379   2.6736  109  0.0087     0.0262 
## Exp_typeResource:Year    -0.1481    0.0516  -2.8718  109  0.0049    -0.2503 
##                           ci.ub     
## intrcpt                -51.2277  ** 
## Exp_typeResource       503.7507  ** 
## Year                     0.1764  ** 
## Exp_typeResource:Year   -0.0459  ** 
## 
## ---
## Signif. codes:  0 '***' 0.001 '**' 0.01 '*' 0.05 '.' 0.1 ' ' 1
```

```
i2_ml(mod_23)
```

```
##      I2_Total I2_Article_ID 
##      98.10672      98.10672
```

```
100 - i2_ml(mod_23)[1] # Sampling variance as proportion of total unaccounted variance
```

```
## I2_Total 
## 1.893275
```

```
r2_ml(mod_23)
```

```
##    R2_marginal R2_conditional 
##      0.3087188      0.3087188
```

```
bubble_plot(mod_23, 
            mod = "Year",
            data = data_MA_full,
            group = "Article_ID",
            cb = TRUE,
            k = TRUE,
            g = TRUE,
            legend.pos = "bottom.right",
            by = "Exp_type",
            xlab = "Experiment duration (hours)")+
  
  scale_x_continuous(n.breaks = 7)+
     scale_fill_manual(breaks = c("Resource", "Demand"),
                     values = c("#1B9E77", "#D95F02"))
```

There is no interaction effect between driver and experiment
duration, regardless of extreme values being included (p = 0.71) or
excluded (0.495), both drivers are essentially unaffected by experiment
duration despite the positive and negative trends for demand and
resource effects respectively.

#### 5.2.11 Driver x N:P ratio difference

None of the models we attempted to construct to test this interaction
reached convergence, but there is a strong indication of an interaction
effect based on the results and orchard plot from the model
`delta_RR ~ NP_ratio_diff` constructed above.

#### 5.2.12 Prey:Grazer ratio

```
mod_prey_pred_ratio <- rma.mv(yi = delta_RR,
                V = Var_delta_RR, 
                mods = ~ 1 + (Phytoplankton_group * prey_grazer_ratio_ml), 
                method = "REML", 
                test = "t",
                random = list(~1 | Article_ID,
                              ~1 + Phytoplankton_group| Effect_ID),
                rho = 0, 
                struc = "HCS", 
                data = data_MA_full %>%  drop_na(prey_grazer_ratio_ml),
                control = list(optimizer="optim", optmethod="Nelder-Mead")
                  )

summary.rma(mod_prey_pred_ratio)
```

```
## 
## Multivariate Meta-Analysis Model (k = 46; method: REML)
## 
##   logLik  Deviance       AIC       BIC      AICc   
## -49.7398   99.4796  113.4796  125.6433  116.7737   
## 
## Variance Components:
## 
##             estim    sqrt  nlvls  fixed      factor 
## sigma^2    0.3174  0.5634     16     no  Article_ID 
## 
## outer factor: Effect_ID           (nlvls = 46)
## inner factor: Phytoplankton_group (nlvls = 2)
## 
##             estim    sqrt  k.lvl  fixed             level 
## tau^2.1    0.3741  0.6117     33     no       Alexandrium 
## tau^2.2    0.4529  0.6730     13     no  Pseudo-nitzschia 
## rho        0.0000                   yes                   
## 
## Test for Residual Heterogeneity:
## QE(df = 42) = 1519.6191, p-val < .0001
## 
## Test of Moderators (coefficients 2:4):
## F(df1 = 3, df2 = 42) = 7.0258, p-val = 0.0006
## 
## Model Results:
## 
##                                                           estimate      se 
## intrcpt                                                     0.8648  0.2400 
## Phytoplankton_groupPseudo-nitzschia                         2.1831  0.4811 
## prey_grazer_ratio_ml                                        0.0000  0.0000 
## Phytoplankton_groupPseudo-nitzschia:prey_grazer_ratio_ml   -0.0000  0.0000 
##                                                              tval  df    pval 
## intrcpt                                                    3.6029  42  0.0008 
## Phytoplankton_groupPseudo-nitzschia                        4.5376  42  <.0001 
## prey_grazer_ratio_ml                                       0.5527  42  0.5834 
## Phytoplankton_groupPseudo-nitzschia:prey_grazer_ratio_ml  -1.8529  42  0.0709 
##                                                             ci.lb   ci.ub      
## intrcpt                                                    0.3804  1.3493  *** 
## Phytoplankton_groupPseudo-nitzschia                        1.2122  3.1540  *** 
## prey_grazer_ratio_ml                                      -0.0000  0.0000      
## Phytoplankton_groupPseudo-nitzschia:prey_grazer_ratio_ml  -0.0000  0.0000    . 
## 
## ---
## Signif. codes:  0 '***' 0.001 '**' 0.01 '*' 0.05 '.' 0.1 ' ' 1
```

```
i2_ml(mod_prey_pred_ratio)
```

```
##      I2_Total I2_Article_ID 
##      96.20519      96.20519
```

```
100 - i2_ml(mod_prey_pred_ratio)[1] # Sampling variance as proportion of total unaccounted variance
```

```
## I2_Total 
## 3.794806
```

```
r2_ml(mod_prey_pred_ratio)
```

```
##    R2_marginal R2_conditional 
##      0.6658648      0.6658648
```

```
## Set up interaction model for post-hoc analysis
mod_preypredratio_interaction <- rma.mv(yi = delta_RR,
                V = Var_delta_RR, 
                mods = ~ 0 + prey_grazer_ratio_ml:Phytoplankton_group, 
                method = "REML", 
                test = "t", 
                random = list(~1 | Article_ID,
                              ~1 + Exp_type| Effect_ID),
                rho = 0, 
                struc = "HCS", 
                data = data_MA_full %>%  drop_na(prey_grazer_ratio_ml),
                control = list(optimizer="optim", optmethod="Nelder-Mead")
                  )

# conservative post-hoc results (with Holms adjustment)
summary(glht(mod_preypredratio_interaction, linfct=cbind(contrMat(rep(1,2), type="Tukey"))), test=adjusted("holm"))
```

```
## 
##   Simultaneous Tests for General Linear Hypotheses
## 
## Fit: rma.mv(yi = delta_RR, V = Var_delta_RR, mods = ~0 + prey_grazer_ratio_ml:Phytoplankton_group, 
##     random = list(~1 | Article_ID, ~1 + Exp_type | Effect_ID), 
##     struct = "HCS", data = data_MA_full %>% drop_na(prey_grazer_ratio_ml), 
##     method = "REML", test = "t", rho = 0, control = list(optimizer = "optim", 
##         optmethod = "Nelder-Mead"))
## 
## Linear Hypotheses:
##              Estimate Std. Error z value Pr(>|z|)
## 2 - 1 == 0 -2.797e-06  1.732e-06  -1.615    0.106
## (Adjusted p values reported -- holm method)
```

```
## Extract group-wise results
res_mod_prey_pred_ratio <-
  orchaRd::mod_results(mod_prey_pred_ratio,
                       mod = "prey_grazer_ratio_ml",
                       group = "Phytoplankton_group",
                       by = "Phytoplankton_group",
                       data = data_MA_full)$mod_table

bubble_plot(mod_prey_pred_ratio, 
            mod = "prey_grazer_ratio_ml",
            data = data_MA_full %>% drop_na(prey_grazer_ratio_ml),
            group = "Article_ID",
            cb = TRUE,
            k = TRUE,
            g = TRUE,
            legend.pos = "bottom.right",
            by = "Phytoplankton_group",
            xlab = "Prey:Predator ratio (mL^-1)"
            )+
  
  scale_x_continuous(n.breaks = 7)
```

#### 5.2.13 Prey concentration:Grazer dry mass ratio

```
mod_prey_pred_DW_ratio_new <- rma.mv(yi = delta_RR,
                V = Var_delta_RR, 
                mods = ~ 1 + (Phytoplankton_group * Prey_conc_pred_DW_ratio), 
                method = "REML", 
                test = "t",
                random = list(~1 | Article_ID,
                              ~1 + Phytoplankton_group| Effect_ID),
                rho = 0, 
                struc = "HCS", 
                data = data_MA_full %>% 
                  #filter(Prey_conc_pred_DW_ratio <6000000) %>% 
                  drop_na(Prey_conc_pred_DW_ratio),
                control = list(optimizer="optim", optmethod="Nelder-Mead")
                  )

summary.rma(mod_prey_pred_DW_ratio_new)
```

```
## 
## Multivariate Meta-Analysis Model (k = 46; method: REML)
## 
##   logLik  Deviance       AIC       BIC      AICc   
## -51.4080  102.8160  116.8160  128.9797  120.1102   
## 
## Variance Components:
## 
##             estim    sqrt  nlvls  fixed      factor 
## sigma^2    0.2806  0.5297     16     no  Article_ID 
## 
## outer factor: Effect_ID           (nlvls = 46)
## inner factor: Phytoplankton_group (nlvls = 2)
## 
##             estim    sqrt  k.lvl  fixed             level 
## tau^2.1    0.3828  0.6187     33     no       Alexandrium 
## tau^2.2    0.7280  0.8532     13     no  Pseudo-nitzschia 
## rho        0.0000                   yes                   
## 
## Test for Residual Heterogeneity:
## QE(df = 42) = 1515.1919, p-val < .0001
## 
## Test of Moderators (coefficients 2:4):
## F(df1 = 3, df2 = 42) = 4.9298, p-val = 0.0051
## 
## Model Results:
## 
##                                                              estimate      se 
## intrcpt                                                        0.8875  0.2278 
## Phytoplankton_groupPseudo-nitzschia                            1.6759  0.4827 
## Prey_conc_pred_DW_ratio                                        0.0000  0.0000 
## Phytoplankton_groupPseudo-nitzschia:Prey_conc_pred_DW_ratio   -0.0000  0.0001 
##                                                                 tval  df 
## intrcpt                                                       3.8967  42 
## Phytoplankton_groupPseudo-nitzschia                           3.4717  42 
## Prey_conc_pred_DW_ratio                                       0.3718  42 
## Phytoplankton_groupPseudo-nitzschia:Prey_conc_pred_DW_ratio  -0.2414  42 
##                                                                pval    ci.lb 
## intrcpt                                                      0.0003   0.4279 
## Phytoplankton_groupPseudo-nitzschia                          0.0012   0.7017 
## Prey_conc_pred_DW_ratio                                      0.7119  -0.0000 
## Phytoplankton_groupPseudo-nitzschia:Prey_conc_pred_DW_ratio  0.8104  -0.0002 
##                                                               ci.ub      
## intrcpt                                                      1.3471  *** 
## Phytoplankton_groupPseudo-nitzschia                          2.6501   ** 
## Prey_conc_pred_DW_ratio                                      0.0001      
## Phytoplankton_groupPseudo-nitzschia:Prey_conc_pred_DW_ratio  0.0002      
## 
## ---
## Signif. codes:  0 '***' 0.001 '**' 0.01 '*' 0.05 '.' 0.1 ' ' 1
```

```
i2_ml(mod_prey_pred_DW_ratio_new)
```

```
##      I2_Total I2_Article_ID 
##      95.72884      95.72884
```

```
100 - i2_ml(mod_prey_pred_DW_ratio_new)[1] # Sampling variance as proportion of total unaccounted variance
```

```
## I2_Total 
## 4.271161
```

```
r2_ml(mod_prey_pred_DW_ratio_new)
```

```
##    R2_marginal R2_conditional 
##      0.6553257      0.6553257
```

```
## Set up interaction model for post-hoc analysis
mod_prey_pred_DW_ratio_interaction <- rma.mv(yi = delta_RR,
                V = Var_delta_RR, 
                mods = ~ 0 + Prey_conc_pred_DW_ratio:Phytoplankton_group, 
                method = "REML", 
                test = "t", 
                random = list(~1 | Article_ID,
                              ~1 + Exp_type| Effect_ID),
                rho = 0, 
                struc = "HCS", 
                data = data_MA_full %>% 
                  #filter(Prey_conc_pred_DW_ratio <6000000) %>% 
                  drop_na(Prey_conc_pred_DW_ratio),
                control = list(optimizer="optim", optmethod="Nelder-Mead")
                  )

# conservative post-hoc results (with Holms adjustment)
summary(glht(mod_prey_pred_DW_ratio_interaction, linfct=cbind(contrMat(rep(1,2), type="Tukey"))), test=adjusted("holm"))
```

```
## 
##   Simultaneous Tests for General Linear Hypotheses
## 
## Fit: rma.mv(yi = delta_RR, V = Var_delta_RR, mods = ~0 + Prey_conc_pred_DW_ratio:Phytoplankton_group, 
##     random = list(~1 | Article_ID, ~1 + Exp_type | Effect_ID), 
##     struct = "HCS", data = data_MA_full %>% drop_na(Prey_conc_pred_DW_ratio), 
##     method = "REML", test = "t", rho = 0, control = list(optimizer = "optim", 
##         optmethod = "Nelder-Mead"))
## 
## Linear Hypotheses:
##             Estimate Std. Error z value Pr(>|z|)
## 2 - 1 == 0 1.030e-04  9.005e-05   1.143    0.253
## (Adjusted p values reported -- holm method)
```

```
#Scatter/Bubble plot
bubble_plot(mod_prey_pred_DW_ratio_new, 
            mod = "Prey_conc_pred_DW_ratio",
            data = data_MA_full %>% 
                  drop_na(Prey_conc_pred_DW_ratio),
            group = "Article_ID",
            cb = TRUE,
            k = TRUE,
            g = TRUE,
            legend.pos = "bottom.right",
            by = "Phytoplankton_group",
            xlab = "Prey conc. (cells mL^-1) to grazer DW (µg mL^-1) ratio"
            )+
  
  scale_x_continuous(n.breaks = 7)
```

#### 5.2.14 Grazer dry mass

```
mod_pred_DW_mL <- rma.mv(yi = delta_RR,
                V = Var_delta_RR, 
                mods = ~ 1 + (Phytoplankton_group * Dry_mass_tot_µg_mL), 
                method = "REML", 
                test = "t",
                random = list(~1 | Article_ID,
                              ~1 + Phytoplankton_group| Effect_ID),
                rho = 0, 
                struc = "HCS", 
                data = data_MA_full %>% 
                  drop_na(Dry_mass_tot_µg_mL),
                control = list(optimizer="optim", optmethod="Nelder-Mead")
                  )

summary.rma(mod_pred_DW_mL)
```

```
## 
## Multivariate Meta-Analysis Model (k = 46; method: REML)
## 
##   logLik  Deviance       AIC       BIC      AICc   
## -49.1525   98.3050  112.3050  124.4686  115.5991   
## 
## Variance Components:
## 
##             estim    sqrt  nlvls  fixed      factor 
## sigma^2    0.4327  0.6578     16     no  Article_ID 
## 
## outer factor: Effect_ID           (nlvls = 46)
## inner factor: Phytoplankton_group (nlvls = 2)
## 
##             estim    sqrt  k.lvl  fixed             level 
## tau^2.1    0.3849  0.6204     33     no       Alexandrium 
## tau^2.2    0.2320  0.4817     13     no  Pseudo-nitzschia 
## rho        0.0000                   yes                   
## 
## Test for Residual Heterogeneity:
## QE(df = 42) = 1624.1354, p-val < .0001
## 
## Test of Moderators (coefficients 2:4):
## F(df1 = 3, df2 = 42) = 8.6706, p-val = 0.0001
## 
## Model Results:
## 
##                                                         estimate      se 
## intrcpt                                                   0.9700  0.3126 
## Phytoplankton_groupPseudo-nitzschia                       1.9764  0.4768 
## Dry_mass_tot_µg_mL                                       -0.0426  0.2565 
## Phytoplankton_groupPseudo-nitzschia:Dry_mass_tot_µg_mL   -0.0662  0.2589 
##                                                            tval  df    pval 
## intrcpt                                                  3.1030  42  0.0034 
## Phytoplankton_groupPseudo-nitzschia                      4.1453  42  0.0002 
## Dry_mass_tot_µg_mL                                      -0.1659  42  0.8690 
## Phytoplankton_groupPseudo-nitzschia:Dry_mass_tot_µg_mL  -0.2557  42  0.7994 
##                                                           ci.lb   ci.ub      
## intrcpt                                                  0.3391  1.6009   ** 
## Phytoplankton_groupPseudo-nitzschia                      1.0142  2.9386  *** 
## Dry_mass_tot_µg_mL                                      -0.5602  0.4751      
## Phytoplankton_groupPseudo-nitzschia:Dry_mass_tot_µg_mL  -0.5887  0.4563      
## 
## ---
## Signif. codes:  0 '***' 0.001 '**' 0.01 '*' 0.05 '.' 0.1 ' ' 1
```

```
i2_ml(mod_pred_DW_mL)
```

```
##      I2_Total I2_Article_ID 
##        97.188        97.188
```

```
100 - i2_ml(mod_pred_DW_mL)[1] # Sampling variance as proportion of total unaccounted variance
```

```
## I2_Total 
## 2.811996
```

```
r2_ml(mod_pred_DW_mL)
```

```
##    R2_marginal R2_conditional 
##      0.5967241      0.5967241
```

```
## Set up interaction model for post-hoc analysis
mod_pred_DW_interaction <- rma.mv(yi = delta_RR,
                V = Var_delta_RR, 
                mods = ~ 0 + Dry_mass_tot_µg_mL:Phytoplankton_group, 
                method = "REML", 
                test = "t", 
                random = list(~1 | Article_ID,
                              ~1 + Exp_type| Effect_ID),
                rho = 0, 
                struc = "HCS", 
                data = data_MA_full %>% 
                  drop_na(Dry_mass_tot_µg_mL),
                control = list(optimizer="optim", optmethod="Nelder-Mead")
                  )

# conservative post-hoc results (with Holms adjustment)
summary(glht(mod_pred_DW_interaction, linfct=cbind(contrMat(rep(1,2), type="Tukey"))), test=adjusted("holm"))
```

```
## 
##   Simultaneous Tests for General Linear Hypotheses
## 
## Fit: rma.mv(yi = delta_RR, V = Var_delta_RR, mods = ~0 + Dry_mass_tot_µg_mL:Phytoplankton_group, 
##     random = list(~1 | Article_ID, ~1 + Exp_type | Effect_ID), 
##     struct = "HCS", data = data_MA_full %>% drop_na(Dry_mass_tot_µg_mL), 
##     method = "REML", test = "t", rho = 0, control = list(optimizer = "optim", 
##         optmethod = "Nelder-Mead"))
## 
## Linear Hypotheses:
##            Estimate Std. Error z value Pr(>|z|)
## 2 - 1 == 0  -0.1885     0.2587  -0.729    0.466
## (Adjusted p values reported -- holm method)
```

```
#Scatter/Bubble plot
bubble_plot(mod_pred_DW_mL, 
            mod = "Dry_mass_tot_µg_mL",
            data = data_MA_full %>% 
                 # filter(Prey_conc_pred_DW_.1_ratio <6000000) %>% 
                  drop_na(Dry_mass_tot_µg_mL),
            group = "Article_ID",
            cb = TRUE,
            k = TRUE,
            g = TRUE,
            legend.pos = "bottom.right",
            by = "Phytoplankton_group",
            xlab = "Grazer dry mass (µg mL^-1)"
            )+
  
  scale_x_continuous(#limits = c(-1000, 5000000),
                     n.breaks = 7)
```

## 6. Sensitivity analyses and bias testing

### 6.1 Removing cases that violated Geary’s rule from final analysis

To determine if the effects that violate Geary’s rule distorts the
results significantly, we fit the same two final models (LRR\(^\Delta\) ~ Driver & (LRR\(^\Delta\)) ~ Driver x Phytoplankton genus)
using only the 101 cases that did not violate the rule.

#### 6.1.1 LRR\(^\Delta\) ~ Driver

```
mod_13_geary <- rma.mv(yi = delta_RR,
                V = Var_delta_RR, 
                mods = ~ 1 + Exp_type, 
                method = "REML", 
                test = "t", 
                random = list(~1 | Article_ID,
                              ~1 + Exp_type | Effect_ID),
                rho = 0, 
                struc = "HCS", 
                data = data_MA_geary,
                control = list(optimizer="optim", optmethod="Nelder-Mead")
                )

summary.rma(mod_13_geary)
```

```
## 
## Multivariate Meta-Analysis Model (k = 101; method: REML)
## 
##    logLik   Deviance        AIC        BIC       AICc   
## -150.6258   301.2516   311.2516   324.2272   311.8968   
## 
## Variance Components:
## 
##             estim    sqrt  nlvls  fixed      factor 
## sigma^2    0.5863  0.7657     37     no  Article_ID 
## 
## outer factor: Effect_ID (nlvls = 101)
## inner factor: Exp_type  (nlvls = 2)
## 
##             estim    sqrt  k.lvl  fixed     level 
## tau^2.1    0.5913  0.7690     63     no    Demand 
## tau^2.2    1.5077  1.2279     38     no  Resource 
## rho        0.0000                   yes           
## 
## Test for Residual Heterogeneity:
## QE(df = 99) = 15472.5856, p-val < .0001
## 
## Test of Moderators (coefficient 2):
## F(df1 = 1, df2 = 99) = 0.9445, p-val = 0.3335
## 
## Model Results:
## 
##                   estimate      se     tval  df    pval    ci.lb   ci.ub      
## intrcpt             1.5122  0.1997   7.5734  99  <.0001   1.1160  1.9084  *** 
## Exp_typeResource   -0.3144  0.3235  -0.9718  99  0.3335  -0.9564  0.3275      
## 
## ---
## Signif. codes:  0 '***' 0.001 '**' 0.01 '*' 0.05 '.' 0.1 ' ' 1
```

```
i2_ml(mod_13_geary)
```

```
##      I2_Total I2_Article_ID 
##      98.74824      98.74824
```

```
r2_ml(mod_13_geary)
```

```
##    R2_marginal R2_conditional 
##     0.03843213     0.03843213
```

```
# Results table
orchaRd::mod_results(mod_13_geary,
                     mod = "Exp_type",
                     group = "Exp_type",
                     data = data_MA_full)$mod_table
```

```
##       name estimate   lowerCL  upperCL    lowerPR  upperPR
## 1   Demand 1.512201 1.1160096 1.908392 -0.6771921 3.701594
## 2 Resource 1.197768 0.6540201 1.741515 -1.7245594 4.120095
```

```
# As percentage increase
100*(exp(orchaRd::mod_results(mod_13_geary,
                     mod = "Exp_type",
                     group = "Exp_type",
                     data = data_MA_full)$mod_table[2:6])-1)
```

```
##   estimate  lowerCL  upperCL   lowerPR  upperPR
## 1 353.6705 205.2649 574.2240 -49.19585 3951.183
## 2 231.2713  92.3257 470.5981 -82.17484 6056.506
```

```
# Full data results
100*(exp(res_mod_13[2:6])-1)
```

```
##   estimate  lowerCL  upperCL   lowerPR  upperPR
## 1 388.1724 230.2409 621.6316 -51.82128 4846.422
## 2 266.8875 125.1301 497.9053 -76.41500 5607.291
```

```
orchard_plot(mod_13_geary,
             data = data_MA_full,
             mod = "Exp_type",
             group = "Article_ID", # Colour of the bubbles in the plot & number in parentheses.
             xlab = bquote(LRR^~Delta),
             angle = 0,
             alpha = 0.4,
             cb = FALSE,
             k = TRUE,
             g = TRUE,
             trunk.size = 8,
             branch.size = 1.5,
             twig.size = 0.5,
             colour = TRUE,
             fill = TRUE,
             legend.pos = "bottom.right"
             ) +
  
  scale_y_continuous(n.breaks = 10)
```

Effects decrease with approximately 30 percentage units for each
driver, but does not change the overall results significantly.

#### 6.1.2 LRR\(^\Delta\) ~ Driver x phytoplankotn genus

```
mod_16_geary <- rma.mv(yi = delta_RR,
                V = Var_delta_RR, 
                mods = ~ 1 + (Exp_type * Phytoplankton_group), 
                method = "REML", 
                test = "t", 
                random = list(~1 | Article_ID,
                              ~1 + Exp_type| Effect_ID),
                rho = 0, 
                struc = "HCS", 
                data = data_MA_geary,
                control = list(optimizer="optim", optmethod="Nelder-Mead")
                  )

summary.rma(mod_16_geary)
```

```
## 
## Multivariate Meta-Analysis Model (k = 101; method: REML)
## 
##    logLik   Deviance        AIC        BIC       AICc   
## -129.4340   258.8679   272.8679   290.8909   274.1263   
## 
## Variance Components:
## 
##             estim    sqrt  nlvls  fixed      factor 
## sigma^2    0.5461  0.7390     37     no  Article_ID 
## 
## outer factor: Effect_ID (nlvls = 101)
## inner factor: Exp_type  (nlvls = 2)
## 
##             estim    sqrt  k.lvl  fixed     level 
## tau^2.1    0.2620  0.5118     63     no    Demand 
## tau^2.2    1.6275  1.2757     38     no  Resource 
## rho        0.0000                   yes           
## 
## Test for Residual Heterogeneity:
## QE(df = 97) = 9448.2964, p-val < .0001
## 
## Test of Moderators (coefficients 2:4):
## F(df1 = 3, df2 = 97) = 17.6206, p-val < .0001
## 
## Model Results:
## 
##                                                       estimate      se     tval 
## intrcpt                                                 0.8590  0.1998   4.2987 
## Exp_typeResource                                        0.1507  0.3464   0.4350 
## Phytoplankton_groupPseudo-nitzschia                     2.0650  0.2875   7.1831 
## Exp_typeResource:Phytoplankton_groupPseudo-nitzschia   -1.6439  0.7261  -2.2641 
##                                                       df    pval    ci.lb 
## intrcpt                                               97  <.0001   0.4624 
## Exp_typeResource                                      97  0.6646  -0.5369 
## Phytoplankton_groupPseudo-nitzschia                   97  <.0001   1.4945 
## Exp_typeResource:Phytoplankton_groupPseudo-nitzschia  97  0.0258  -3.0849 
##                                                         ci.ub      
## intrcpt                                                1.2556  *** 
## Exp_typeResource                                       0.8383      
## Phytoplankton_groupPseudo-nitzschia                    2.6356  *** 
## Exp_typeResource:Phytoplankton_groupPseudo-nitzschia  -0.2028    * 
## 
## ---
## Signif. codes:  0 '***' 0.001 '**' 0.01 '*' 0.05 '.' 0.1 ' ' 1
```

```
i2_ml(mod_16_geary)
```

```
##      I2_Total I2_Article_ID 
##       98.6572       98.6572
```

```
100 - i2_ml(mod_16_geary)[1] # Sampling variance as proportion of total unaccounted variance
```

```
## I2_Total 
## 1.342799
```

```
r2_ml(mod_16_geary)
```

```
##    R2_marginal R2_conditional 
##      0.5267025      0.5267025
```

```
100*(exp(orchaRd::mod_results(mod_16_geary, 
                     group = "Phytoplankton_group", 
                     mod = "Exp_type", 
                     by = "Phytoplankton_group", 
                     weights = "prop", 
                     data = data_MA_full)$mod_table[3])-1)
```

```
##    estimate
## 1  136.0719
## 2  174.4649
## 3 1761.5581
## 4  318.2076
```

```
100*(exp(res_mod_16$estimate)-1)
```

```
## [1]  167.0806  211.9422 1706.3943  393.4703
```

```
orchard_plot(mod_16_geary, 
             mod = "Exp_type",
             data = data_MA_full,
             group = "Article_ID",
             by =  "Phytoplankton_group",
             xlab = "lnRR (effect size)",
             angle = 0,
             alpha = 0.4,
             cb = FALSE,
             k = TRUE,
             g = TRUE,
             trunk.size = 6,
             branch.size = 1.5,
             twig.size = 0.7,
             colour = TRUE,
             legend.pos = "bottom.right") +
  
  scale_y_continuous(n.breaks = 10)
```

Mean effect decreases with 30-80 percentage units, but does not
change the overall results significantly.

Although the cases that violate Geary’s rule seem to inflate the
overall results somewhat, they do not change the results in a meaningful
way, and keeping them in the analysis increases it’s ecological
relevance because several of the removed cases were from experiments on
*Pseudo-nitzschia*, which seem to have very low (almost zero)
amounts of toxins in control conditions and are then hyper induced when
exposed to grazing pressure or increased N:P-ratio.

### 6.2 Publication bias

Publication bias encompass several types of bias related to the
dissemination of scientific information. Here, we focus on two main
types: outcome reporting bias, which occurs when published studies are
reported selectively, e.g., that non-significant results are less likely
to be published (i.e. “The file drawer problem” formulated by Rosenthal
(1979)), and time-lag
bias, where significant and/or large and/or positive/corroborating
results are published earlier than non-significant, small and negative
results. Two features that are extremely common in meta-analyses in
ecology and evolution, large amounts of heterogeneity and
non-independence of effects, complicate and invalidate many of the
common methods to detect and quantify bias.

Because we have substantial heterogeneity and non-independent
effects, like most meta-analyses in ecology, “standard” methods such as
regular funnel plots, trim-and-fill procedures, leave-one-out analysis
etc., are not appropriate to use. Other metrics of bias, like the many
fail-safe numbers that have been proposed to date, are problematic in
both function and interpretation. Following suggestions by Nakagawa and
colleagues (2022) we
will perform a cumulative multi-level meta-analysis to visually assess
time-lag bias with the change in mean effects over time, followed by
multilevel meta-regressions to statistically test for publication bias
and time-lag bias. It is important to note that despite the fact that
this multilevel meta-regression seem to be one of the only statistically
robust methods of identifying publication bias in data with high
heterogeneity and non-independence, simulation simulation studies have
revealed high Type II error for almost all methods, even when they have
small Type I error rates (Fernández-Castilla
*et al.*, 2021; Rodgers &
Pustejovsky, 2021). Therefore, not finding evidence for
publication bias using these test should not be interpreted as proof for
the lack of publication bias.

#### 6.2.1 Cumulative effect over time for each driver

Because the `cumul` function in the `metafor`
package does not work with mixed-effects/multi-level models constructed
by the `rma.mv` function or regular random-effects models
that include moderators,, we must construct separate models based on a
growing subset of the data iteratively.

##### 6.2.1.1 List of unique studies

```
study_list <- data_MA_full %>%   
  dplyr::select(Article_ID, Year) %>%   
  unique() %>%   
  dplyr::arrange(Year) %>%  
  as.data.frame() 

study_list <- rename(study_list, Study = Article_ID)
View(study_list)
```

##### 6.2.1.2 Individual analyses, adding one paper at a time chronologically

```
# Only first paper in dataset
mod_cumul_1 <- rma.mv(yi = delta_RR,
                 V = Var_delta_RR, 
                 mods = ~ 1 + Exp_type, 
                 method = "REML", 
                 test = "t", 
                 random = list(~1 | Article_ID,
                               ~1 + Exp_type| Effect_ID),
                 rho = 0, 
                 struc = "HCS", 
                 data = data_MA_full %>%   filter(Article_ID == "Bechemin_1999"),
                 control = list(optimizer="optim", optmethod="Nelder-Mead")
)
  
res_cumul_1 <- data.frame(Author = "Bechemin et al",
                          Year = 1999,
                          Driver = "Resource", 
                          Estimate = mod_cumul_1$b, 
                          LCL = mod_cumul_1$ci.lb, 
                          UCL = mod_cumul_1$ci.ub,
                          N = mod_cumul_1$s.nlevels,
                          K = mod_cumul_1$g.nlevels[2], 
                          row.names = NULL) 
res_cumul_1
```

```
##           Author Year   Driver Estimate        LCL      UCL N K
## 1 Bechemin et al 1999 Resource 3.608535 0.06559949 7.151471 1 2
```

```
# + 2nd paper
mod_cumul_2 <- rma.mv(yi = delta_RR,
                 V = Var_delta_RR, 
                 mods = ~ 1 + Exp_type, 
                 method = "REML", 
                 test = "t", 
                 random = list(~1 | Article_ID,
                               ~1 + Exp_type| Effect_ID),
                 rho = 0, 
                 struc = "HCS", 
                 data = data_MA_full %>%   filter(Article_ID == "Bechemin_1999" |
                                                 Article_ID == "Guisande_2002" ),
                 control = list(optimizer="optim", optmethod="Nelder-Mead"))

res_cumul_2 <- 
orchaRd::mod_results(mod_cumul_2,
                     mod = "Exp_type",
                     group = "Exp_type",
                     data = data_MA_full)$mod_table


res_cumul_2 <-
data.frame(Author = c("Guisande et al", "Guisande et al"),
           Year = c(2002, 2002),
           Driver = c("Demand", "Resource"),
           Estimate = res_cumul_2$estimate, 
           LCL = res_cumul_2$lowerCL, 
           UCL = res_cumul_2$upperCL,
           N = mod_cumul_2$s.nlevels,
           K = c(mod_cumul_2$g.levels.k[1],mod_cumul_2$g.levels.k[2]), 
           row.names = NULL)

res_cumul_2
```

```
##           Author Year   Driver  Estimate         LCL        UCL N K
## 1 Guisande et al 2002   Demand 0.8760613 -8020.02225 8021.77438 2 1
## 2 Guisande et al 2002 Resource 3.6085351   -38.89678   46.11385 2 2
```

```
# + 3rd paper
mod_cumul_3 <- rma.mv(yi = delta_RR,
                 V = Var_delta_RR, 
                 mods = ~ 1 + Exp_type, 
                 method = "REML", 
                 test = "t", 
                 random = list(~1 | Article_ID,
                               ~1 + Exp_type| Effect_ID),
                 rho = 0, 
                 struc = "HCS", 
                 data = data_MA_full %>%   filter(Article_ID == "Bechemin_1999" |
                                                 Article_ID == "Guisande_2002" |
                                                 Article_ID ==  "Grzebyk_2003" ),
                 control = list(optimizer="optim", optmethod="Nelder-Mead"))

res_cumul_3 <- 
orchaRd::mod_results(mod_cumul_3,
                     mod = "Exp_type",
                     group = "Exp_type",
                     data = data_MA_full)$mod_table

res_cumul_3 <-
data.frame(Author = c("Grzebyk et al", "Grzebyk et al"),
           Year = c(2003, 2003),
           Driver = c("Demand", "Resource"),
           Estimate = res_cumul_3$estimate, 
           LCL = res_cumul_3$lowerCL, 
           UCL = res_cumul_3$upperCL,
           N = mod_cumul_3$s.nlevels,
           K = c(mod_cumul_3$g.levels.k[1],mod_cumul_3$g.levels.k[2]),
           row.names = NULL)

res_cumul_3
```

```
##          Author Year   Driver  Estimate         LCL        UCL N K
## 1 Grzebyk et al 2003   Demand 0.8760613 -348.946558 350.698681 3 1
## 2 Grzebyk et al 2003 Resource 2.7184780   -1.061648   6.498604 3 3
```

```
# + 4th paper
mod_cumul_4 <- rma.mv(yi = delta_RR,
                 V = Var_delta_RR, 
                 mods = ~ 1 + Exp_type, 
                 method = "REML", 
                 test = "t", 
                 random = list(~1 | Article_ID,
                               ~1 + Exp_type| Effect_ID),
                 rho = 0, 
                 struc = "HCS", 
                 data = data_MA_full %>%   filter(Article_ID == "Bechemin_1999" |
                                                 Article_ID == "Guisande_2002" |
                                                 Article_ID ==  "Grzebyk_2003" |
                                                 Article_ID == "Lippemeier_2003"),
                 control = list(optimizer="optim", optmethod="Nelder-Mead"))


res_cumul_4 <- 
orchaRd::mod_results(mod_cumul_4,
                     mod = "Exp_type",
                     group = "Exp_type",
                     data = data_MA_full)$mod_table

res_cumul_4 <-
data.frame(Author = c("Lippemeier et al", "Lippemeier et al"),
           Year = c(2003, 2003),
           Driver = c("Demand", "Resource"),
           Estimate = res_cumul_4$estimate, 
           LCL = res_cumul_4$lowerCL, 
           UCL = res_cumul_4$upperCL,
           N = mod_cumul_4$s.nlevels,
           K = c(mod_cumul_4$g.levels.k[1],mod_cumul_4$g.levels.k[2]),
           row.names = NULL)

res_cumul_4
```

```
##             Author Year   Driver  Estimate           LCL        UCL N K
## 1 Lippemeier et al 2003   Demand 0.8760613 -5643.8862843 5645.63841 4 1
## 2 Lippemeier et al 2003 Resource 2.4437159     0.6252922    4.26214 4 4
```

```
# + 5th paper
mod_cumul_5 <- rma.mv(yi = delta_RR,
                 V = Var_delta_RR, 
                 mods = ~ 1 + Exp_type, 
                 method = "REML", 
                 test = "t", 
                 random = list(~1 | Article_ID,
                               ~1 + Exp_type| Effect_ID),
                 rho = 0, 
                 struc = "HCS", 
                 data = data_MA_full %>%   filter(Article_ID == "Bechemin_1999" |
                                                 Article_ID == "Guisande_2002" |
                                                 Article_ID ==  "Grzebyk_2003" |
                                                 Article_ID == "Lippemeier_2003" |
                                                 Article_ID == "Leong_2004"),
                 control = list(optimizer="optim", optmethod="Nelder-Mead"))


res_cumul_5 <- 
orchaRd::mod_results(mod_cumul_5,
                     mod = "Exp_type",
                     group = "Exp_type",
                     data = data_MA_full)$mod_table

res_cumul_5 <-
data.frame(Author = c("Leong et al", "Leong et al"),
           Year = c(2004, 2004),
           Driver = c("Demand", "Resource"),
           Estimate = res_cumul_5$estimate, 
           LCL = res_cumul_5$lowerCL, 
           UCL = res_cumul_5$upperCL,
           N = mod_cumul_5$s.nlevels,
           K = c(mod_cumul_5$g.levels.k[1],mod_cumul_5$g.levels.k[2]),
           row.names = NULL)

res_cumul_5
```

```
##        Author Year   Driver  Estimate        LCL      UCL N K
## 1 Leong et al 2004   Demand 0.8760613 -2.3811614 4.133284 5 1
## 2 Leong et al 2004 Resource 2.0364503  0.4276732 3.645227 5 7
```

```
# + 6th paper
mod_cumul_6 <- rma.mv(yi = delta_RR,
                 V = Var_delta_RR, 
                 mods = ~ 1 + Exp_type, 
                 method = "REML", 
                 test = "t", 
                 random = list(~1 | Article_ID,
                               ~1 + Exp_type| Effect_ID),
                 rho = 0, 
                 struc = "HCS", 
                 data = data_MA_full %>%   filter(Article_ID == "Bechemin_1999" |
                                                 Article_ID == "Guisande_2002" |
                                                 Article_ID ==  "Grzebyk_2003" |
                                                 Article_ID == "Lippemeier_2003" |
                                                 Article_ID == "Leong_2004" |
                                                 Article_ID == "Selander_2006" ),
                 
                 control = list(optimizer="optim", optmethod="Nelder-Mead"))


res_cumul_6 <- 
orchaRd::mod_results(mod_cumul_6,
                     mod = "Exp_type",
                     group = "Exp_type",
                     data = data_MA_full)$mod_table

res_cumul_6 <-
data.frame(Author = c("Selander et al", "Selander et al"),
           Year = c(2006, 2006),
           Driver = c("Demand", "Resource"),
           Estimate = res_cumul_6$estimate, 
           LCL = res_cumul_6$lowerCL, 
           UCL = res_cumul_6$upperCL,
           N = mod_cumul_6$s.nlevels,
           K = c(mod_cumul_6$g.levels.k[1],mod_cumul_6$g.levels.k[2]),
           row.names = NULL)

res_cumul_6
```

```
##           Author Year   Driver  Estimate        LCL      UCL N K
## 1 Selander et al 2006   Demand 0.8103843 -0.4287220 2.049491 6 4
## 2 Selander et al 2006 Resource 2.0088509  0.7795152 3.238187 6 7
```

```
# + 7th paper
mod_cumul_7 <- rma.mv(yi = delta_RR,
                 V = Var_delta_RR, 
                 mods = ~ 1 + Exp_type, 
                 method = "REML", 
                 test = "t", 
                 random = list(~1 | Article_ID,
                               ~1 + Exp_type| Effect_ID),
                 rho = 0, 
                 struc = "HCS", 
                 data = data_MA_full %>%   filter(Article_ID == "Bechemin_1999" |
                                                 Article_ID == "Guisande_2002" |
                                                 Article_ID ==  "Grzebyk_2003" |
                                                 Article_ID == "Lippemeier_2003" |
                                                 Article_ID == "Leong_2004" |
                                                 Article_ID == "Selander_2006" |
                                                 Article_ID == "Hu_2006"  ),
                 
                 control = list(optimizer="optim", optmethod="Nelder-Mead"))


res_cumul_7 <- 
orchaRd::mod_results(mod_cumul_7,
                     mod = "Exp_type",
                     group = "Exp_type",
                     data = data_MA_full)$mod_table

res_cumul_7 <-
data.frame(Author = c("Hu et al", "Hu et al"),
           Year = c(2006, 2006),
           Driver = c("Demand", "Resource"),
           Estimate = res_cumul_7$estimate, 
           LCL = res_cumul_7$lowerCL, 
           UCL = res_cumul_7$upperCL,
           N = mod_cumul_7$s.nlevels,
           K = c(mod_cumul_7$g.levels.k[1],mod_cumul_7$g.levels.k[2]),
           row.names = NULL)

res_cumul_7
```

```
##     Author Year   Driver  Estimate       LCL       UCL N K
## 1 Hu et al 2006   Demand 0.7831827 0.6495303 0.9168352 7 4
## 2 Hu et al 2006 Resource 1.7374208 0.6513326 2.8235090 7 8
```

```
# + 8th paper
mod_cumul_8 <- rma.mv(yi = delta_RR,
                 V = Var_delta_RR, 
                 mods = ~ 1 + Exp_type, 
                 method = "REML", 
                 test = "t", 
                 random = list(~1 | Article_ID,
                               ~1 + Exp_type| Effect_ID),
                 rho = 0, 
                 struc = "HCS", 
                 data = data_MA_full %>%   filter(Article_ID == "Bechemin_1999" |
                                                 Article_ID == "Guisande_2002" |
                                                 Article_ID ==  "Grzebyk_2003" |
                                                 Article_ID == "Lippemeier_2003" |
                                                 Article_ID == "Leong_2004" |
                                                 Article_ID == "Selander_2006" |
                                                 Article_ID == "Hu_2006"  |
                                                 Article_ID ==  "Murata_2006"),
                 
                 control = list(optimizer="optim", optmethod="Nelder-Mead"))


res_cumul_8 <- 
orchaRd::mod_results(mod_cumul_8,
                     mod = "Exp_type",
                     group = "Exp_type",
                     data = data_MA_full)$mod_table

res_cumul_8 <-
data.frame(Author = c("Murata et al", "Murata et al"),
           Year = c(2006, 2006),
           Driver = c("Demand", "Resource"),
           Estimate = res_cumul_8$estimate, 
           LCL = res_cumul_8$lowerCL, 
           UCL = res_cumul_8$upperCL,
           N = mod_cumul_8$s.nlevels,
           K = c(mod_cumul_8$g.levels.k[1],mod_cumul_8$g.levels.k[2]),
           row.names = NULL)

res_cumul_8
```

```
##         Author Year   Driver  Estimate        LCL      UCL N K
## 1 Murata et al 2006   Demand 0.8103142 -0.3029027 1.923531 8 4
## 2 Murata et al 2006 Resource 1.6005285  0.6134657 2.587591 8 9
```

```
# + 9th paper
mod_cumul_9 <- rma.mv(yi = delta_RR,
                 V = Var_delta_RR, 
                 mods = ~ 1 + Exp_type, 
                 method = "REML", 
                 test = "t", 
                 random = list(~1 | Article_ID,
                               ~1 + Exp_type| Effect_ID),
                 rho = 0, 
                 struc = "HCS", 
                 data = data_MA_full %>%   filter(Article_ID == "Bechemin_1999" |
                                                 Article_ID == "Guisande_2002" |
                                                 Article_ID ==  "Grzebyk_2003" |
                                                 Article_ID == "Lippemeier_2003" |
                                                 Article_ID == "Leong_2004" |
                                                 Article_ID == "Selander_2006" |
                                                 Article_ID == "Hu_2006"  |
                                                 Article_ID ==  "Murata_2006" | 
                                                 Article_ID ==  "Bergkvist_2008" ),
                 
                 control = list(optimizer="optim", optmethod="Nelder-Mead"))


res_cumul_9 <- 
orchaRd::mod_results(mod_cumul_9,
                     mod = "Exp_type",
                     group = "Exp_type",
                     data = data_MA_full)$mod_table

res_cumul_9 <-
data.frame(Author = c("Bergkvist et al", "Bergkvist et al"),
           Year = c(2008, 2008),
           Driver = c("Demand", "Resource"),
           Estimate = res_cumul_9$estimate, 
           LCL = res_cumul_9$lowerCL, 
           UCL = res_cumul_9$upperCL,
           N = mod_cumul_9$s.nlevels,
           K = c(mod_cumul_9$g.levels.k[1],mod_cumul_9$g.levels.k[2]),
           row.names = NULL)

res_cumul_9
```

```
##            Author Year   Driver  Estimate        LCL      UCL N  K
## 1 Bergkvist et al 2008   Demand 0.8857121 -0.1767122 1.948136 9 10
## 2 Bergkvist et al 2008 Resource 1.6036124  0.6733197 2.533905 9  9
```

```
# + 10th paper
mod_cumul_10 <- rma.mv(yi = delta_RR,
                 V = Var_delta_RR, 
                 mods = ~ 1 + Exp_type, 
                 method = "REML", 
                 test = "t", 
                 random = list(~1 | Article_ID,
                               ~1 + Exp_type| Effect_ID),
                 rho = 0, 
                 struc = "HCS", 
                 data = data_MA_full %>%   filter(Article_ID == "Bechemin_1999" |
                                                 Article_ID == "Guisande_2002" |
                                                 Article_ID ==  "Grzebyk_2003" |
                                                 Article_ID == "Lippemeier_2003" |
                                                 Article_ID == "Leong_2004" |
                                                 Article_ID == "Selander_2006" |
                                                 Article_ID == "Hu_2006"  |
                                                 Article_ID ==  "Murata_2006" | 
                                                 Article_ID ==  "Bergkvist_2008" |
                                                 Article_ID ==  "Selander_2008"),
                 
                 control = list(optimizer="optim", optmethod="Nelder-Mead"))


res_cumul_10 <- 
orchaRd::mod_results(mod_cumul_10,
                     mod = "Exp_type",
                     group = "Exp_type",
                     data = data_MA_full)$mod_table

res_cumul_10 <-
data.frame(Author = c("Selander et al", "Selander et al"),
           Year = c(2008, 2008),
           Driver = c("Demand", "Resource"),
           Estimate = res_cumul_10$estimate, 
           LCL = res_cumul_10$lowerCL, 
           UCL = res_cumul_10$upperCL,
           N = mod_cumul_10$s.nlevels,
           K = c(mod_cumul_10$g.levels.k[1],mod_cumul_10$g.levels.k[2]),
           row.names = NULL)

res_cumul_10
```

```
##           Author Year   Driver  Estimate       LCL     UCL  N  K
## 1 Selander et al 2008   Demand 0.8534312 0.3613329 1.34553 10 12
## 2 Selander et al 2008 Resource 1.7538522 0.9645943 2.54311 10 11
```

```
# + 11th paper
mod_cumul_11 <- rma.mv(yi = delta_RR,
                 V = Var_delta_RR, 
                 mods = ~ 1 + Exp_type, 
                 method = "REML", 
                 test = "t", 
                 random = list(~1 | Article_ID,
                               ~1 + Exp_type| Effect_ID),
                 rho = 0, 
                 struc = "HCS", 
                 data = data_MA_full %>%   filter(Article_ID == "Bechemin_1999" |
                                                 Article_ID == "Guisande_2002" |
                                                 Article_ID ==  "Grzebyk_2003" |
                                                 Article_ID == "Lippemeier_2003" |
                                                 Article_ID == "Leong_2004" |
                                                 Article_ID == "Selander_2006" |
                                                 Article_ID == "Hu_2006"  |
                                                 Article_ID ==  "Murata_2006" | 
                                                 Article_ID ==  "Bergkvist_2008" |
                                                 Article_ID ==  "Selander_2008" |
                                                 Article_ID == "Wohlrab_2010" ),
                 
                 control = list(optimizer="optim", optmethod="Nelder-Mead"))


res_cumul_11 <- 
orchaRd::mod_results(mod_cumul_11,
                     mod = "Exp_type",
                     group = "Exp_type",
                     data = data_MA_full)$mod_table

res_cumul_11 <-
data.frame(Author = c("Wohlrab et al", "Wohlrab et al"),
           Year = c(2010, 2010),
           Driver = c("Demand", "Resource"),
           Estimate = res_cumul_11$estimate, 
           LCL = res_cumul_11$lowerCL, 
           UCL = res_cumul_11$upperCL,
           N = mod_cumul_11$s.nlevels,
           K = c(mod_cumul_11$g.levels.k[1],mod_cumul_11$g.levels.k[2]),
           row.names = NULL)

res_cumul_11
```

```
##          Author Year   Driver  Estimate       LCL      UCL  N  K
## 1 Wohlrab et al 2010   Demand 0.7371963 0.3336534 1.140739 11 15
## 2 Wohlrab et al 2010 Resource 1.7538517 0.9705647 2.537139 11 11
```

```
# + 12th paper
mod_cumul_12 <- rma.mv(yi = delta_RR,
                 V = Var_delta_RR, 
                 mods = ~ 1 + Exp_type, 
                 method = "REML", 
                 test = "t", 
                 random = list(~1 | Article_ID,
                               ~1 + Exp_type| Effect_ID),
                 rho = 0, 
                 struc = "HCS", 
                 data = data_MA_full %>%   filter(Article_ID == "Bechemin_1999" |
                                                 Article_ID == "Guisande_2002" |
                                                 Article_ID ==  "Grzebyk_2003" |
                                                 Article_ID == "Lippemeier_2003" |
                                                 Article_ID == "Leong_2004" |
                                                 Article_ID == "Selander_2006" |
                                                 Article_ID == "Hu_2006"  |
                                                 Article_ID ==  "Murata_2006" | 
                                                 Article_ID ==  "Bergkvist_2008" |
                                                 Article_ID ==  "Selander_2008" |
                                                 Article_ID == "Wohlrab_2010" |
                                                 Article_ID == "Yang_2011a" ),
                 
                 control = list(optimizer="optim", optmethod="Nelder-Mead"))


res_cumul_12 <- 
orchaRd::mod_results(mod_cumul_12,
                     mod = "Exp_type",
                     group = "Exp_type",
                     data = data_MA_full)$mod_table

res_cumul_12 <-
data.frame(Author = c("Yang et al (a)", "Yang et al (a)"),
           Year = c(2011, 2011),
           Driver = c("Demand", "Resource"),
           Estimate = res_cumul_12$estimate, 
           LCL = res_cumul_12$lowerCL, 
           UCL = res_cumul_12$upperCL,
           N = mod_cumul_12$s.nlevels,
           K = c(mod_cumul_12$g.levels.k[1],mod_cumul_12$g.levels.k[2]),
           row.names = NULL)

res_cumul_12
```

```
##           Author Year   Driver  Estimate       LCL      UCL  N  K
## 1 Yang et al (a) 2011   Demand 0.7956737 0.3993416 1.192006 12 16
## 2 Yang et al (a) 2011 Resource 1.7538562 0.9721674 2.535545 12 11
```

```
# + 13th paper
mod_cumul_13 <- rma.mv(yi = delta_RR,
                 V = Var_delta_RR, 
                 mods = ~ 1 + Exp_type, 
                 method = "REML", 
                 test = "t", 
                 random = list(~1 | Article_ID,
                               ~1 + Exp_type| Effect_ID),
                 rho = 0, 
                 struc = "HCS", 
                 data = data_MA_full %>%   filter(Article_ID == "Bechemin_1999" |
                                                 Article_ID == "Guisande_2002" |
                                                 Article_ID ==  "Grzebyk_2003" |
                                                 Article_ID == "Lippemeier_2003" |
                                                 Article_ID == "Leong_2004" |
                                                 Article_ID == "Selander_2006" |
                                                 Article_ID == "Hu_2006"  |
                                                 Article_ID ==  "Murata_2006" | 
                                                 Article_ID ==  "Bergkvist_2008" |
                                                 Article_ID ==  "Selander_2008" |
                                                 Article_ID == "Wohlrab_2010" |
                                                 Article_ID == "Yang_2011a" |
                                                 Article_ID == "Yang_2011b" ),
                 
                 control = list(optimizer="optim", optmethod="Nelder-Mead"))


res_cumul_13 <- 
orchaRd::mod_results(mod_cumul_13,
                     mod = "Exp_type",
                     group = "Exp_type",
                     data = data_MA_full)$mod_table

res_cumul_13 <-
data.frame(Author = c("Yang et al (b)", "Yang et al (b)"),
           Year = c(2011, 2011),
           Driver = c("Demand", "Resource"),
           Estimate = res_cumul_13$estimate, 
           LCL = res_cumul_13$lowerCL, 
           UCL = res_cumul_13$upperCL,
           N = mod_cumul_13$s.nlevels,
           K = c(mod_cumul_13$g.levels.k[1],mod_cumul_13$g.levels.k[2]),
           row.names = NULL)

res_cumul_13
```

```
##           Author Year   Driver  Estimate       LCL      UCL  N  K
## 1 Yang et al (b) 2011   Demand 0.7956723 0.4008373 1.190507 13 16
## 2 Yang et al (b) 2011 Resource 1.5611450 0.8461807 2.276109 13 13
```

```
# + 14th paper
mod_cumul_14 <- rma.mv(yi = delta_RR,
                 V = Var_delta_RR, 
                 mods = ~ 1 + Exp_type, 
                 method = "REML", 
                 test = "t", 
                 random = list(~1 | Article_ID,
                               ~1 + Exp_type| Effect_ID),
                 rho = 0, 
                 struc = "HCS", 
                 data = data_MA_full %>%   filter(Article_ID == "Bechemin_1999" |
                                                 Article_ID == "Guisande_2002" |
                                                 Article_ID ==  "Grzebyk_2003" |
                                                 Article_ID == "Lippemeier_2003" |
                                                 Article_ID == "Leong_2004" |
                                                 Article_ID == "Selander_2006" |
                                                 Article_ID == "Hu_2006"  |
                                                 Article_ID ==  "Murata_2006" | 
                                                 Article_ID ==  "Bergkvist_2008" |
                                                 Article_ID ==  "Selander_2008" |
                                                 Article_ID == "Wohlrab_2010" |
                                                 Article_ID == "Yang_2011a" |
                                                 Article_ID == "Yang_2011b" |
                                                 Article_ID == "Selander_2012" ),
                 
                 control = list(optimizer="optim", optmethod="Nelder-Mead"))


res_cumul_14 <- 
orchaRd::mod_results(mod_cumul_14,
                     mod = "Exp_type",
                     group = "Exp_type",
                     data = data_MA_full)$mod_table

res_cumul_14 <-
data.frame(Author = c("Selander et al", "Selander et al"),
           Year = c(2012, 2012),
           Driver = c("Demand", "Resource"),
           Estimate = res_cumul_14$estimate, 
           LCL = res_cumul_14$lowerCL, 
           UCL = res_cumul_14$upperCL,
           N = mod_cumul_14$s.nlevels,
           K = c(mod_cumul_14$g.levels.k[1],mod_cumul_14$g.levels.k[2]),
           row.names = NULL)

res_cumul_14
```

```
##           Author Year   Driver  Estimate       LCL      UCL  N  K
## 1 Selander et al 2012   Demand 0.7980185 0.4870335 1.109004 14 20
## 2 Selander et al 2012 Resource 1.5611473 0.8504474 2.271847 14 13
```

```
# + 15th paper
mod_cumul_15 <- rma.mv(yi = delta_RR,
                 V = Var_delta_RR, 
                 mods = ~ 1 + Exp_type, 
                 method = "REML", 
                 test = "t", 
                 random = list(~1 | Article_ID,
                               ~1 + Exp_type| Effect_ID),
                 rho = 0, 
                 struc = "HCS", 
                 data = data_MA_full %>%   filter(Article_ID == "Bechemin_1999" |
                                                 Article_ID == "Guisande_2002" |
                                                 Article_ID ==  "Grzebyk_2003" |
                                                 Article_ID == "Lippemeier_2003" |
                                                 Article_ID == "Leong_2004" |
                                                 Article_ID == "Selander_2006" |
                                                 Article_ID == "Hu_2006"  |
                                                 Article_ID ==  "Murata_2006" | 
                                                 Article_ID ==  "Bergkvist_2008" |
                                                 Article_ID ==  "Selander_2008" |
                                                 Article_ID == "Wohlrab_2010" |
                                                 Article_ID == "Yang_2011a" |
                                                 Article_ID == "Yang_2011b" |
                                                 Article_ID == "Selander_2012" |
                                                 Article_ID == "Lee_2012" ),
                 
                 control = list(optimizer="optim", optmethod="Nelder-Mead"))


res_cumul_15 <- 
orchaRd::mod_results(mod_cumul_15,
                     mod = "Exp_type",
                     group = "Exp_type",
                     data = data_MA_full)$mod_table

res_cumul_15 <-
data.frame(Author = c("Lee et al", "Lee et al"),
           Year = c(2012, 2012),
           Driver = c("Demand", "Resource"),
           Estimate = res_cumul_15$estimate, 
           LCL = res_cumul_15$lowerCL, 
           UCL = res_cumul_15$upperCL,
           N = mod_cumul_15$s.nlevels,
           K = c(mod_cumul_15$g.levels.k[1],mod_cumul_15$g.levels.k[2]),
           row.names = NULL)

res_cumul_15
```

```
##      Author Year   Driver Estimate       LCL      UCL  N  K
## 1 Lee et al 2012   Demand 0.798015 0.4878224 1.108208 15 20
## 2 Lee et al 2012 Resource 1.447486 0.8154464 2.079526 15 15
```

```
# + 16th paper
mod_cumul_16 <- rma.mv(yi = delta_RR,
                 V = Var_delta_RR, 
                 mods = ~ 1 + Exp_type, 
                 method = "REML", 
                 test = "t", 
                 random = list(~1 | Article_ID,
                               ~1 + Exp_type| Effect_ID),
                 rho = 0, 
                 struc = "HCS", 
                 data = data_MA_full %>%   filter(Article_ID == "Bechemin_1999" |
                                                 Article_ID == "Guisande_2002" |
                                                 Article_ID ==  "Grzebyk_2003" |
                                                 Article_ID == "Lippemeier_2003" |
                                                 Article_ID == "Leong_2004" |
                                                 Article_ID == "Selander_2006" |
                                                 Article_ID == "Hu_2006"  |
                                                 Article_ID ==  "Murata_2006" | 
                                                 Article_ID ==  "Bergkvist_2008" |
                                                 Article_ID ==  "Selander_2008" |
                                                 Article_ID == "Wohlrab_2010" |
                                                 Article_ID == "Yang_2011a" |
                                                 Article_ID == "Yang_2011b" |
                                                 Article_ID == "Selander_2012" |
                                                 Article_ID == "Lee_2012" |
                                                 Article_ID == "Murata_2012" ),
                 
                 control = list(optimizer="optim", optmethod="Nelder-Mead"))


res_cumul_16 <- 
orchaRd::mod_results(mod_cumul_16,
                     mod = "Exp_type",
                     group = "Exp_type",
                     data = data_MA_full)$mod_table

res_cumul_16 <-
data.frame(Author = c("Murata et al", "Murata et al"),
           Year = c(2012, 2012),
           Driver = c("Demand", "Resource"),
           Estimate = res_cumul_16$estimate, 
           LCL = res_cumul_16$lowerCL, 
           UCL = res_cumul_16$upperCL,
           N = mod_cumul_16$s.nlevels,
           K = c(mod_cumul_16$g.levels.k[1],mod_cumul_16$g.levels.k[2]),
           row.names = NULL)

res_cumul_16
```

```
##         Author Year   Driver  Estimate       LCL      UCL  N  K
## 1 Murata et al 2012   Demand 0.7980219 0.4890386 1.107005 16 20
## 2 Murata et al 2012 Resource 1.2579353 0.7338564 1.782014 16 19
```

```
# + 17th paper
mod_cumul_17 <- rma.mv(yi = delta_RR,
                 V = Var_delta_RR, 
                 mods = ~ 1 + Exp_type, 
                 method = "REML", 
                 test = "t", 
                 random = list(~1 | Article_ID,
                               ~1 + Exp_type| Effect_ID),
                 rho = 0, 
                 struc = "HCS", 
                 data = data_MA_full %>%   filter(Article_ID == "Bechemin_1999" |
                                                 Article_ID == "Guisande_2002" |
                                                 Article_ID ==  "Grzebyk_2003" |
                                                 Article_ID == "Lippemeier_2003" |
                                                 Article_ID == "Leong_2004" |
                                                 Article_ID == "Selander_2006" |
                                                 Article_ID == "Hu_2006"  |
                                                 Article_ID ==  "Murata_2006" | 
                                                 Article_ID ==  "Bergkvist_2008" |
                                                 Article_ID ==  "Selander_2008" |
                                                 Article_ID == "Wohlrab_2010" |
                                                 Article_ID == "Yang_2011a" |
                                                 Article_ID == "Yang_2011b" |
                                                 Article_ID == "Selander_2012" |
                                                 Article_ID == "Lee_2012" |
                                                 Article_ID == "Murata_2012" |
                                                 Article_ID == "Tatters_2013" ),
                 
                 control = list(optimizer="optim", optmethod="Nelder-Mead"))


res_cumul_17 <- 
orchaRd::mod_results(mod_cumul_17,
                     mod = "Exp_type",
                     group = "Exp_type",
                     data = data_MA_full)$mod_table

res_cumul_17 <-
data.frame(Author = c("Tatters et al", "Tatters et al"),
           Year = c(2013, 2013),
           Driver = c("Demand", "Resource"),
           Estimate = res_cumul_17$estimate, 
           LCL = res_cumul_17$lowerCL, 
           UCL = res_cumul_17$upperCL,
           N = mod_cumul_17$s.nlevels,
           K = c(mod_cumul_17$g.levels.k[1],mod_cumul_17$g.levels.k[2]),
           row.names = NULL)

res_cumul_17
```

```
##          Author Year   Driver  Estimate       LCL      UCL  N  K
## 1 Tatters et al 2013   Demand 0.7980118 0.4896483 1.106375 17 20
## 2 Tatters et al 2013 Resource 1.3615093 0.8616344 1.861384 17 21
```

```
# + 18th paper
mod_cumul_18 <- rma.mv(yi = delta_RR,
                 V = Var_delta_RR, 
                 mods = ~ 1 + Exp_type, 
                 method = "REML", 
                 test = "t", 
                 random = list(~1 | Article_ID,
                               ~1 + Exp_type| Effect_ID),
                 rho = 0, 
                 struc = "HCS", 
                 data = data_MA_full %>%   filter(Article_ID == "Bechemin_1999" |
                                                 Article_ID == "Guisande_2002" |
                                                 Article_ID ==  "Grzebyk_2003" |
                                                 Article_ID == "Lippemeier_2003" |
                                                 Article_ID == "Leong_2004" |
                                                 Article_ID == "Selander_2006" |
                                                 Article_ID == "Hu_2006"  |
                                                 Article_ID ==  "Murata_2006" | 
                                                 Article_ID ==  "Bergkvist_2008" |
                                                 Article_ID ==  "Selander_2008" |
                                                 Article_ID == "Wohlrab_2010" |
                                                 Article_ID == "Yang_2011a" |
                                                 Article_ID == "Yang_2011b" |
                                                 Article_ID == "Selander_2012" |
                                                 Article_ID == "Lee_2012" |
                                                 Article_ID == "Murata_2012" |
                                                 Article_ID == "Tatters_2013" |
                                                 Article_ID == "VanDeWaal_2013" ),
                 
                 control = list(optimizer="optim", optmethod="Nelder-Mead"))


res_cumul_18 <- 
orchaRd::mod_results(mod_cumul_18,
                     mod = "Exp_type",
                     group = "Exp_type",
                     data = data_MA_full)$mod_table

res_cumul_18 <-
data.frame(Author = c("Van de Waal et al", "Van de Waal et al"),
           Year = c(2013, 2013),
           Driver = c("Demand", "Resource"),
           Estimate = res_cumul_18$estimate, 
           LCL = res_cumul_18$lowerCL, 
           UCL = res_cumul_18$upperCL,
           N = mod_cumul_18$s.nlevels,
           K = c(mod_cumul_18$g.levels.k[1],mod_cumul_18$g.levels.k[2]),
           row.names = NULL)

res_cumul_18
```

```
##              Author Year   Driver  Estimate       LCL      UCL  N  K
## 1 Van de Waal et al 2013   Demand 0.7087629 0.1554969 1.262029 18 20
## 2 Van de Waal et al 2013 Resource 1.3851502 0.9030122 1.867288 18 23
```

```
# + 19th paper
mod_cumul_19 <- rma.mv(yi = delta_RR,
                 V = Var_delta_RR, 
                 mods = ~ 1 + Exp_type, 
                 method = "REML", 
                 test = "t", 
                 random = list(~1 | Article_ID,
                               ~1 + Exp_type| Effect_ID),
                 rho = 0, 
                 struc = "HCS", 
                 data = data_MA_full %>%   filter(Article_ID == "Bechemin_1999" |
                                                 Article_ID == "Guisande_2002" |
                                                 Article_ID ==  "Grzebyk_2003" |
                                                 Article_ID == "Lippemeier_2003" |
                                                 Article_ID == "Leong_2004" |
                                                 Article_ID == "Selander_2006" |
                                                 Article_ID == "Hu_2006"  |
                                                 Article_ID ==  "Murata_2006" | 
                                                 Article_ID ==  "Bergkvist_2008" |
                                                 Article_ID ==  "Selander_2008" |
                                                 Article_ID == "Wohlrab_2010" |
                                                 Article_ID == "Yang_2011a" |
                                                 Article_ID == "Yang_2011b" |
                                                 Article_ID == "Selander_2012" |
                                                 Article_ID == "Lee_2012" |
                                                 Article_ID == "Murata_2012" |
                                                 Article_ID == "Tatters_2013" |
                                                 Article_ID == "VanDeWaal_2013" |
                                                 Article_ID == "Selander_2015"  ),
                 
                 control = list(optimizer="optim", optmethod="Nelder-Mead"))


res_cumul_19 <- 
orchaRd::mod_results(mod_cumul_19,
                     mod = "Exp_type",
                     group = "Exp_type",
                     data = data_MA_full)$mod_table

res_cumul_19 <-
data.frame(Author = c("Selander et al", "Selander et al"),
           Year = c(2015, 2015),
           Driver = c("Demand", "Resource"),
           Estimate = res_cumul_19$estimate, 
           LCL = res_cumul_19$lowerCL, 
           UCL = res_cumul_19$upperCL,
           N = mod_cumul_19$s.nlevels,
           K = c(mod_cumul_19$g.levels.k[1],mod_cumul_19$g.levels.k[2]),
           row.names = NULL)

res_cumul_19
```

```
##           Author Year   Driver  Estimate       LCL      UCL  N  K
## 1 Selander et al 2015   Demand 0.8752472 0.2657182 1.484776 19 26
## 2 Selander et al 2015 Resource 1.4885767 0.9385316 2.038622 19 23
```

```
# + 20th paper
mod_cumul_20 <- rma.mv(yi = delta_RR,
                 V = Var_delta_RR, 
                 mods = ~ 1 + Exp_type, 
                 method = "REML", 
                 test = "t", 
                 random = list(~1 | Article_ID,
                               ~1 + Exp_type| Effect_ID),
                 rho = 0, 
                 struc = "HCS", 
                 data = data_MA_full %>%   filter(Article_ID == "Bechemin_1999" |
                                                 Article_ID == "Guisande_2002" |
                                                 Article_ID ==  "Grzebyk_2003" |
                                                 Article_ID == "Lippemeier_2003" |
                                                 Article_ID == "Leong_2004" |
                                                 Article_ID == "Selander_2006" |
                                                 Article_ID == "Hu_2006"  |
                                                 Article_ID ==  "Murata_2006" | 
                                                 Article_ID ==  "Bergkvist_2008" |
                                                 Article_ID ==  "Selander_2008" |
                                                 Article_ID == "Wohlrab_2010" |
                                                 Article_ID == "Yang_2011a" |
                                                 Article_ID == "Yang_2011b" |
                                                 Article_ID == "Selander_2012" |
                                                 Article_ID == "Lee_2012" |
                                                 Article_ID == "Murata_2012" |
                                                 Article_ID == "Tatters_2013" |
                                                 Article_ID == "VanDeWaal_2013" |
                                                 Article_ID == "Selander_2015" | 
                                                 Article_ID == "SenftBatoh_2015a" ),
                 
                 control = list(optimizer="optim", optmethod="Nelder-Mead"))


res_cumul_20 <- 
orchaRd::mod_results(mod_cumul_20,
                     mod = "Exp_type",
                     group = "Exp_type",
                     data = data_MA_full)$mod_table

res_cumul_20 <-
data.frame(Author = c("Senft-Batoh et al (a)", "Senft-Batoh et al (a)"),
           Year = c(2015, 2015),
           Driver = c("Demand", "Resource"),
           Estimate = res_cumul_20$estimate, 
           LCL = res_cumul_20$lowerCL, 
           UCL = res_cumul_20$upperCL,
           N = mod_cumul_20$s.nlevels,
           K = c(mod_cumul_20$g.levels.k[1],mod_cumul_20$g.levels.k[2]),
           row.names = NULL)


res_cumul_20
```

```
##                  Author Year   Driver  Estimate       LCL      UCL  N  K
## 1 Senft-Batoh et al (a) 2015   Demand 0.8698852 0.3136828 1.426088 20 28
## 2 Senft-Batoh et al (a) 2015 Resource 1.4793530 0.9420062 2.016700 20 23
```

```
# + 21st paper
mod_cumul_21 <- rma.mv(yi = delta_RR,
                 V = Var_delta_RR, 
                 mods = ~ 1 + Exp_type, 
                 method = "REML", 
                 test = "t", 
                 random = list(~1 | Article_ID,
                               ~1 + Exp_type| Effect_ID),
                 rho = 0, 
                 struc = "HCS", 
                 data = data_MA_full %>%   filter(Article_ID == "Bechemin_1999" |
                                                 Article_ID == "Guisande_2002" |
                                                 Article_ID ==  "Grzebyk_2003" |
                                                 Article_ID == "Lippemeier_2003" |
                                                 Article_ID == "Leong_2004" |
                                                 Article_ID == "Selander_2006" |
                                                 Article_ID == "Hu_2006"  |
                                                 Article_ID ==  "Murata_2006" | 
                                                 Article_ID ==  "Bergkvist_2008" |
                                                 Article_ID ==  "Selander_2008" |
                                                 Article_ID == "Wohlrab_2010" |
                                                 Article_ID == "Yang_2011a" |
                                                 Article_ID == "Yang_2011b" |
                                                 Article_ID == "Selander_2012" |
                                                 Article_ID == "Lee_2012" |
                                                 Article_ID == "Murata_2012" |
                                                 Article_ID == "Tatters_2013" |
                                                 Article_ID == "VanDeWaal_2013" |
                                                 Article_ID == "Selander_2015" | 
                                                 Article_ID == "SenftBatoh_2015a" |
                                                 Article_ID == "SenftBatoh_2015b" ),
                 
                 control = list(optimizer="optim", optmethod="Nelder-Mead"))


res_cumul_21 <- 
orchaRd::mod_results(mod_cumul_21,
                     mod = "Exp_type",
                     group = "Exp_type",
                     data = data_MA_full)$mod_table

res_cumul_21 <-
data.frame(Author = c("Senft-Batoh et al (b)", "Senft-Batoh et al (b)"),
           Year = c(2015, 2015),
           Driver = c("Demand", "Resource"),
           Estimate = res_cumul_21$estimate, 
           LCL = res_cumul_21$lowerCL, 
           UCL = res_cumul_21$upperCL,
           N = mod_cumul_21$s.nlevels,
           K = c(mod_cumul_21$g.levels.k[1], mod_cumul_21$g.levels.k[2]),
           row.names = NULL)

res_cumul_21
```

```
##                  Author Year   Driver  Estimate       LCL      UCL  N  K
## 1 Senft-Batoh et al (b) 2015   Demand 0.8918182 0.3829274 1.400709 21 32
## 2 Senft-Batoh et al (b) 2015 Resource 1.4635319 0.9405662 1.986498 21 23
```

```
# + paper 22
mod_cumul_22 <- rma.mv(yi = delta_RR,
                 V = Var_delta_RR, 
                 mods = ~ 1 + Exp_type, 
                 method = "REML", 
                 test = "t", 
                 random = list(~1 | Article_ID,
                               ~1 + Exp_type| Effect_ID),
                 rho = 0, 
                 struc = "HCS", 
                 data = data_MA_full %>%   filter(Article_ID == "Bechemin_1999" |
                                                 Article_ID == "Guisande_2002" |
                                                 Article_ID ==  "Grzebyk_2003" |
                                                 Article_ID == "Lippemeier_2003" |
                                                 Article_ID == "Leong_2004" |
                                                 Article_ID == "Selander_2006" |
                                                 Article_ID == "Hu_2006"  |
                                                 Article_ID ==  "Murata_2006" | 
                                                 Article_ID ==  "Bergkvist_2008" |
                                                 Article_ID ==  "Selander_2008" |
                                                 Article_ID == "Wohlrab_2010" |
                                                 Article_ID == "Yang_2011a" |
                                                 Article_ID == "Yang_2011b" |
                                                 Article_ID == "Selander_2012" |
                                                 Article_ID == "Lee_2012" |
                                                 Article_ID == "Murata_2012" |
                                                 Article_ID == "Tatters_2013" |
                                                 Article_ID == "VanDeWaal_2013" |
                                                 Article_ID == "Selander_2015" | 
                                                 Article_ID == "SenftBatoh_2015a" |
                                                 Article_ID == "SenftBatoh_2015b" |
                                                 Article_ID == "Hardardottir_2015" ),
                 
                 control = list(optimizer="optim", optmethod="Nelder-Mead"))


res_cumul_22 <- 
orchaRd::mod_results(mod_cumul_22,
                     mod = "Exp_type",
                     group = "Exp_type",
                     data = data_MA_full)$mod_table

res_cumul_22 <-
data.frame(Author = c("Harðardóttir et al", "Harðardóttir et al"),
           Year = c(2015, 2015),
           Driver = c("Demand", "Resource"),
           Estimate = res_cumul_22$estimate, 
           LCL = res_cumul_22$lowerCL, 
           UCL = res_cumul_22$upperCL,
           N = mod_cumul_22$s.nlevels,
           K = c(mod_cumul_22$g.levels.k[1], mod_cumul_22$g.levels.k[2]),
           row.names = NULL)

res_cumul_22
```

```
##               Author Year   Driver Estimate       LCL      UCL  N  K
## 1 Harðardóttir et al 2015   Demand 1.034964 0.4788149 1.591114 22 34
## 2 Harðardóttir et al 2015 Resource 1.538799 0.9635658 2.114032 22 23
```

```
# + paper 23
mod_cumul_23 <- rma.mv(yi = delta_RR,
                 V = Var_delta_RR, 
                 mods = ~ 1 + Exp_type, 
                 method = "REML", 
                 test = "t", 
                 random = list(~1 | Article_ID,
                               ~1 + Exp_type| Effect_ID),
                 rho = 0, 
                 struc = "HCS", 
                 data = data_MA_full %>%   filter(Article_ID == "Bechemin_1999" |
                                                 Article_ID == "Guisande_2002" |
                                                 Article_ID ==  "Grzebyk_2003" |
                                                 Article_ID == "Lippemeier_2003" |
                                                 Article_ID == "Leong_2004" |
                                                 Article_ID == "Selander_2006" |
                                                 Article_ID == "Hu_2006"  |
                                                 Article_ID ==  "Murata_2006" | 
                                                 Article_ID ==  "Bergkvist_2008" |
                                                 Article_ID ==  "Selander_2008" |
                                                 Article_ID == "Wohlrab_2010" |
                                                 Article_ID == "Yang_2011a" |
                                                 Article_ID == "Yang_2011b" |
                                                 Article_ID == "Selander_2012" |
                                                 Article_ID == "Lee_2012" |
                                                 Article_ID == "Murata_2012" |
                                                 Article_ID == "Tatters_2013" |
                                                 Article_ID == "VanDeWaal_2013" |
                                                 Article_ID == "Selander_2015" | 
                                                 Article_ID == "SenftBatoh_2015a" |
                                                 Article_ID == "SenftBatoh_2015b" |
                                                 Article_ID == "Hardardottir_2015" |
                                                 Article_ID == "Tammilehto_2015"),
                 
                 control = list(optimizer="optim", optmethod="Nelder-Mead"))


res_cumul_23 <- 
orchaRd::mod_results(mod_cumul_23,
                     mod = "Exp_type",
                     group = "Exp_type",
                     data = data_MA_full)$mod_table

res_cumul_23 <-
data.frame(Author = c("Tammilehto et al", "Tammilehto et al"),
           Year = c(2015, 2015),
           Driver = c("Demand", "Resource"),
           Estimate = res_cumul_23$estimate, 
           LCL = res_cumul_23$lowerCL, 
           UCL = res_cumul_23$upperCL,
           N = mod_cumul_23$s.nlevels,
           K = c(mod_cumul_23$g.levels.k[1], mod_cumul_23$g.levels.k[2]),
           row.names = NULL)

res_cumul_23
```

```
##             Author Year   Driver Estimate       LCL      UCL  N  K
## 1 Tammilehto et al 2015   Demand 1.184251 0.5841636 1.784338 23 36
## 2 Tammilehto et al 2015 Resource 1.626095 0.9938527 2.258338 23 23
```

```
# + paper 24
mod_cumul_24 <- rma.mv(yi = delta_RR,
                 V = Var_delta_RR, 
                 mods = ~ 1 + Exp_type, 
                 method = "REML", 
                 test = "t", 
                 random = list(~1 | Article_ID,
                               ~1 + Exp_type| Effect_ID),
                 rho = 0, 
                 struc = "HCS", 
                 data = data_MA_full %>%   filter(Article_ID == "Bechemin_1999" |
                                                 Article_ID == "Guisande_2002" |
                                                 Article_ID ==  "Grzebyk_2003" |
                                                 Article_ID == "Lippemeier_2003" |
                                                 Article_ID == "Leong_2004" |
                                                 Article_ID == "Selander_2006" |
                                                 Article_ID == "Hu_2006"  |
                                                 Article_ID ==  "Murata_2006" | 
                                                 Article_ID ==  "Bergkvist_2008" |
                                                 Article_ID ==  "Selander_2008" |
                                                 Article_ID == "Wohlrab_2010" |
                                                 Article_ID == "Yang_2011a" |
                                                 Article_ID == "Yang_2011b" |
                                                 Article_ID == "Selander_2012" |
                                                 Article_ID == "Lee_2012" |
                                                 Article_ID == "Murata_2012" |
                                                 Article_ID == "Tatters_2013" |
                                                 Article_ID == "VanDeWaal_2013" |
                                                 Article_ID == "Selander_2015" | 
                                                 Article_ID == "SenftBatoh_2015a" |
                                                 Article_ID == "SenftBatoh_2015b" |
                                                 Article_ID == "Hardardottir_2015" |
                                                 Article_ID == "Tammilehto_2015" |
                                                 Article_ID == "Hii_2016" ),
                 
                 control = list(optimizer="optim", optmethod="Nelder-Mead"))


res_cumul_24 <- 
orchaRd::mod_results(mod_cumul_24,
                     mod = "Exp_type",
                     group = "Exp_type",
                     data = data_MA_full)$mod_table

res_cumul_24 <-
data.frame(Author = c("Hii et al", "Hii et al"),
           Year = c(2016, 2016),
           Driver = c("Demand", "Resource"),
           Estimate = res_cumul_24$estimate, 
           LCL = res_cumul_24$lowerCL, 
           UCL = res_cumul_24$upperCL,
           N = mod_cumul_24$s.nlevels,
           K = c(mod_cumul_24$g.levels.k[1], mod_cumul_24$g.levels.k[2]),
           row.names = NULL)


res_cumul_24
```

```
##      Author Year   Driver Estimate       LCL      UCL  N  K
## 1 Hii et al 2016   Demand 1.179239 0.5881361 1.770342 24 36
## 2 Hii et al 2016 Resource 1.543447 0.9429290 2.143965 24 25
```

```
# + paper 25
mod_cumul_25 <- rma.mv(yi = delta_RR,
                 V = Var_delta_RR, 
                 mods = ~ 1 + Exp_type, 
                 method = "REML", 
                 test = "t", 
                 random = list(~1 | Article_ID,
                               ~1 + Exp_type| Effect_ID),
                 rho = 0, 
                 struc = "HCS", 
                 data = data_MA_full %>%   filter(Article_ID == "Bechemin_1999" |
                                                 Article_ID == "Guisande_2002" |
                                                 Article_ID ==  "Grzebyk_2003" |
                                                 Article_ID == "Lippemeier_2003" |
                                                 Article_ID == "Leong_2004" |
                                                 Article_ID == "Selander_2006" |
                                                 Article_ID == "Hu_2006"  |
                                                 Article_ID ==  "Murata_2006" | 
                                                 Article_ID ==  "Bergkvist_2008" |
                                                 Article_ID ==  "Selander_2008" |
                                                 Article_ID == "Wohlrab_2010" |
                                                 Article_ID == "Yang_2011a" |
                                                 Article_ID == "Yang_2011b" |
                                                 Article_ID == "Selander_2012" |
                                                 Article_ID == "Lee_2012" |
                                                 Article_ID == "Murata_2012" |
                                                 Article_ID == "Tatters_2013" |
                                                 Article_ID == "VanDeWaal_2013" |
                                                 Article_ID == "Selander_2015" | 
                                                 Article_ID == "SenftBatoh_2015a" |
                                                 Article_ID == "SenftBatoh_2015b" |
                                                 Article_ID == "Hardardottir_2015" |
                                                 Article_ID == "Tammilehto_2015" |
                                                 Article_ID == "Hii_2016" |
                                                 Article_ID == "Wohlrab_2017"),
                 
                 control = list(optimizer="optim", optmethod="Nelder-Mead"))


res_cumul_25 <- 
orchaRd::mod_results(mod_cumul_25,
                     mod = "Exp_type",
                     group = "Exp_type",
                     data = data_MA_full)$mod_table

res_cumul_25 <-
data.frame(Author = c("Wohlrab et al", "Wohlrab et al"),
           Year = c(2017, 2017),
           Driver = c("Demand", "Resource"),
           Estimate = res_cumul_25$estimate, 
           LCL = res_cumul_25$lowerCL, 
           UCL = res_cumul_25$upperCL,
           N = mod_cumul_25$s.nlevels,
           K = c(mod_cumul_25$g.levels.k[1], mod_cumul_25$g.levels.k[2]),
           row.names = NULL)

res_cumul_25
```

```
##          Author Year   Driver Estimate       LCL      UCL  N  K
## 1 Wohlrab et al 2017   Demand 1.115400 0.5547347 1.676066 25 42
## 2 Wohlrab et al 2017 Resource 1.549433 0.9512726 2.147593 25 25
```

```
# + paper 26
mod_cumul_26 <- rma.mv(yi = delta_RR,
                 V = Var_delta_RR, 
                 mods = ~ 1 + Exp_type, 
                 method = "REML", 
                 test = "t", 
                 random = list(~1 | Article_ID,
                               ~1 + Exp_type| Effect_ID),
                 rho = 0, 
                 struc = "HCS", 
                 data = data_MA_full %>%   filter(Article_ID == "Bechemin_1999" |
                                                 Article_ID == "Guisande_2002" |
                                                 Article_ID ==  "Grzebyk_2003" |
                                                 Article_ID == "Lippemeier_2003" |
                                                 Article_ID == "Leong_2004" |
                                                 Article_ID == "Selander_2006" |
                                                 Article_ID == "Hu_2006"  |
                                                 Article_ID ==  "Murata_2006" | 
                                                 Article_ID ==  "Bergkvist_2008" |
                                                 Article_ID ==  "Selander_2008" |
                                                 Article_ID == "Wohlrab_2010" |
                                                 Article_ID == "Yang_2011a" |
                                                 Article_ID == "Yang_2011b" |
                                                 Article_ID == "Selander_2012" |
                                                 Article_ID == "Lee_2012" |
                                                 Article_ID == "Murata_2012" |
                                                 Article_ID == "Tatters_2013" |
                                                 Article_ID == "VanDeWaal_2013" |
                                                 Article_ID == "Selander_2015" | 
                                                 Article_ID == "SenftBatoh_2015a" |
                                                 Article_ID == "SenftBatoh_2015b" |
                                                 Article_ID == "Hardardottir_2015" |
                                                 Article_ID == "Tammilehto_2015" |
                                                 Article_ID == "Hii_2016" |
                                                 Article_ID == "Wohlrab_2017" |
                                                 Article_ID == "Lema_2017" ),
                 
                 control = list(optimizer="optim", optmethod="Nelder-Mead"))

res_cumul_26 <- 
orchaRd::mod_results(mod_cumul_26,
                     mod = "Exp_type",
                     group = "Exp_type",
                     data = data_MA_full)$mod_table

res_cumul_26 <-
data.frame(Author = c("Lema et al", "Lema et al"),
           Year = c(2017, 2017),
           Driver = c("Demand", "Resource"),
           Estimate = res_cumul_26$estimate, 
           LCL = res_cumul_26$lowerCL, 
           UCL = res_cumul_26$upperCL,
           N = mod_cumul_26$s.nlevels,
           K = c(mod_cumul_26$g.levels.k[1], mod_cumul_26$g.levels.k[2]),
           row.names = NULL)

res_cumul_26
```

```
##       Author Year   Driver Estimate       LCL      UCL  N  K
## 1 Lema et al 2017   Demand 1.117467 0.5691786 1.665755 26 42
## 2 Lema et al 2017 Resource 1.564642 1.0059091 2.123374 26 34
```

```
# + paper 27
mod_cumul_27 <- rma.mv(yi = delta_RR,
                 V = Var_delta_RR, 
                 mods = ~ 1 + Exp_type, 
                 method = "REML", 
                 test = "t", 
                 random = list(~1 | Article_ID,
                               ~1 + Exp_type| Effect_ID),
                 rho = 0, 
                 struc = "HCS", 
                 data = data_MA_full %>%   filter(Article_ID == "Bechemin_1999" |
                                                 Article_ID == "Guisande_2002" |
                                                 Article_ID ==  "Grzebyk_2003" |
                                                 Article_ID == "Lippemeier_2003" |
                                                 Article_ID == "Leong_2004" |
                                                 Article_ID == "Selander_2006" |
                                                 Article_ID == "Hu_2006"  |
                                                 Article_ID ==  "Murata_2006" | 
                                                 Article_ID ==  "Bergkvist_2008" |
                                                 Article_ID ==  "Selander_2008" |
                                                 Article_ID == "Wohlrab_2010" |
                                                 Article_ID == "Yang_2011a" |
                                                 Article_ID == "Yang_2011b" |
                                                 Article_ID == "Selander_2012" |
                                                 Article_ID == "Lee_2012" |
                                                 Article_ID == "Murata_2012" |
                                                 Article_ID == "Tatters_2013" |
                                                 Article_ID == "VanDeWaal_2013" |
                                                 Article_ID == "Selander_2015" | 
                                                 Article_ID == "SenftBatoh_2015a" |
                                                 Article_ID == "SenftBatoh_2015b" |
                                                 Article_ID == "Hardardottir_2015" |
                                                 Article_ID == "Tammilehto_2015" |
                                                 Article_ID == "Hii_2016" |
                                                 Article_ID == "Wohlrab_2017" |
                                                 Article_ID == "Lema_2017" |
                                                 Article_ID == "Lundholm_2018" ),
                 
                 control = list(optimizer="optim", optmethod="Nelder-Mead"))

res_cumul_27 <- 
orchaRd::mod_results(mod_cumul_27,
                     mod = "Exp_type",
                     group = "Exp_type",
                     data = data_MA_full)$mod_table

res_cumul_27 <-
data.frame(Author = c("Lundholm et al", "Lundholm et al"),
           Year = c(2018, 2018),
           Driver = c("Demand", "Resource"),
           Estimate = res_cumul_27$estimate, 
           LCL = res_cumul_27$lowerCL, 
           UCL = res_cumul_27$upperCL,
           N = mod_cumul_27$s.nlevels,
           K = c(mod_cumul_27$g.levels.k[1], mod_cumul_27$g.levels.k[2]),
           row.names = NULL)

res_cumul_27
```

```
##           Author Year   Driver Estimate       LCL      UCL  N  K
## 1 Lundholm et al 2018   Demand 1.166217 0.6491172 1.683316 27 49
## 2 Lundholm et al 2018 Resource 1.534526 0.9938186 2.075233 27 34
```

```
# + paper 28
mod_cumul_28 <- rma.mv(yi = delta_RR,
                 V = Var_delta_RR, 
                 mods = ~ 1 + Exp_type, 
                 method = "REML", 
                 test = "t", 
                 random = list(~1 | Article_ID,
                               ~1 + Exp_type| Effect_ID),
                 rho = 0, 
                 struc = "HCS", 
                 data = data_MA_full %>%   filter(Article_ID == "Bechemin_1999" |
                                                 Article_ID == "Guisande_2002" |
                                                 Article_ID ==  "Grzebyk_2003" |
                                                 Article_ID == "Lippemeier_2003" |
                                                 Article_ID == "Leong_2004" |
                                                 Article_ID == "Selander_2006" |
                                                 Article_ID == "Hu_2006"  |
                                                 Article_ID ==  "Murata_2006" | 
                                                 Article_ID ==  "Bergkvist_2008" |
                                                 Article_ID ==  "Selander_2008" |
                                                 Article_ID == "Wohlrab_2010" |
                                                 Article_ID == "Yang_2011a" |
                                                 Article_ID == "Yang_2011b" |
                                                 Article_ID == "Selander_2012" |
                                                 Article_ID == "Lee_2012" |
                                                 Article_ID == "Murata_2012" |
                                                 Article_ID == "Tatters_2013" |
                                                 Article_ID == "VanDeWaal_2013" |
                                                 Article_ID == "Selander_2015" | 
                                                 Article_ID == "SenftBatoh_2015a" |
                                                 Article_ID == "SenftBatoh_2015b" |
                                                 Article_ID == "Hardardottir_2015" |
                                                 Article_ID == "Tammilehto_2015" |
                                                 Article_ID == "Hii_2016" |
                                                 Article_ID == "Wohlrab_2017" |
                                                 Article_ID == "Lema_2017" |
                                                 Article_ID == "Lundholm_2018" |
                                                 Article_ID == "Griffin_2019"),
                 
                 control = list(optimizer="optim", optmethod="Nelder-Mead"))

res_cumul_28 <- 
orchaRd::mod_results(mod_cumul_28,
                     mod = "Exp_type",
                     group = "Exp_type",
                     data = data_MA_full)$mod_table

res_cumul_28 <-
data.frame(Author = c("Griffin et al", "Griffin et al"),
           Year = c(2019, 2019),
           Driver = c("Demand", "Resource"),
           Estimate = res_cumul_28$estimate, 
           LCL = res_cumul_28$lowerCL, 
           UCL = res_cumul_28$upperCL,
           N = mod_cumul_28$s.nlevels,
           K = c(mod_cumul_28$g.levels.k[1], mod_cumul_28$g.levels.k[2]),
           row.names = NULL)

res_cumul_28
```

```
##          Author Year   Driver Estimate       LCL      UCL  N  K
## 1 Griffin et al 2019   Demand 1.416134 0.9504813 1.881787 28 51
## 2 Griffin et al 2019 Resource 1.254697 0.7547739 1.754620 28 36
```

```
# + paper 29
mod_cumul_29 <- rma.mv(yi = delta_RR,
                 V = Var_delta_RR, 
                 mods = ~ 1 + Exp_type, 
                 method = "REML", 
                 test = "t", 
                 random = list(~1 | Article_ID,
                               ~1 + Exp_type| Effect_ID),
                 rho = 0, 
                 struc = "HCS", 
                 data = data_MA_full %>%   filter(Article_ID == "Bechemin_1999" |
                                                 Article_ID == "Guisande_2002" |
                                                 Article_ID ==  "Grzebyk_2003" |
                                                 Article_ID == "Lippemeier_2003" |
                                                 Article_ID == "Leong_2004" |
                                                 Article_ID == "Selander_2006" |
                                                 Article_ID == "Hu_2006"  |
                                                 Article_ID ==  "Murata_2006" | 
                                                 Article_ID ==  "Bergkvist_2008" |
                                                 Article_ID ==  "Selander_2008" |
                                                 Article_ID == "Wohlrab_2010" |
                                                 Article_ID == "Yang_2011a" |
                                                 Article_ID == "Yang_2011b" |
                                                 Article_ID == "Selander_2012" |
                                                 Article_ID == "Lee_2012" |
                                                 Article_ID == "Murata_2012" |
                                                 Article_ID == "Tatters_2013" |
                                                 Article_ID == "VanDeWaal_2013" |
                                                 Article_ID == "Selander_2015" | 
                                                 Article_ID == "SenftBatoh_2015a" |
                                                 Article_ID == "SenftBatoh_2015b" |
                                                 Article_ID == "Hardardottir_2015" |
                                                 Article_ID == "Tammilehto_2015" |
                                                 Article_ID == "Hii_2016" |
                                                 Article_ID == "Wohlrab_2017" |
                                                 Article_ID == "Lema_2017" |
                                                 Article_ID == "Lundholm_2018" |
                                                 Article_ID == "Griffin_2019" |
                                                 Article_ID == "Hii_2019"),
                 
                 control = list(optimizer="optim", optmethod="Nelder-Mead"))

res_cumul_29 <- 
orchaRd::mod_results(mod_cumul_29,
                     mod = "Exp_type",
                     group = "Exp_type",
                     data = data_MA_full)$mod_table

res_cumul_29 <-
data.frame(Author = c("Hii et al", "Hii et al"),
           Year = c(2019, 2019),
           Driver = c("Demand", "Resource"),
           Estimate = res_cumul_29$estimate, 
           LCL = res_cumul_29$lowerCL, 
           UCL = res_cumul_29$upperCL,
           N = mod_cumul_29$s.nlevels,
           K = c(mod_cumul_29$g.levels.k[1], mod_cumul_29$g.levels.k[2]),
           row.names = NULL)

res_cumul_29
```

```
##      Author Year   Driver Estimate       LCL      UCL  N  K
## 1 Hii et al 2019   Demand 1.410962 0.9498126 1.872112 29 51
## 2 Hii et al 2019 Resource 1.226099 0.7432107 1.708988 29 37
```

```
# + paper 30
mod_cumul_30 <- rma.mv(yi = delta_RR,
                 V = Var_delta_RR, 
                 mods = ~ 1 + Exp_type, 
                 method = "REML", 
                 test = "t", 
                 random = list(~1 | Article_ID,
                               ~1 + Exp_type| Effect_ID),
                 rho = 0, 
                 struc = "HCS", 
                 data = data_MA_full %>%   filter(Article_ID == "Bechemin_1999" |
                                                 Article_ID == "Guisande_2002" |
                                                 Article_ID ==  "Grzebyk_2003" |
                                                 Article_ID == "Lippemeier_2003" |
                                                 Article_ID == "Leong_2004" |
                                                 Article_ID == "Selander_2006" |
                                                 Article_ID == "Hu_2006"  |
                                                 Article_ID ==  "Murata_2006" | 
                                                 Article_ID ==  "Bergkvist_2008" |
                                                 Article_ID ==  "Selander_2008" |
                                                 Article_ID == "Wohlrab_2010" |
                                                 Article_ID == "Yang_2011a" |
                                                 Article_ID == "Yang_2011b" |
                                                 Article_ID == "Selander_2012" |
                                                 Article_ID == "Lee_2012" |
                                                 Article_ID == "Murata_2012" |
                                                 Article_ID == "Tatters_2013" |
                                                 Article_ID == "VanDeWaal_2013" |
                                                 Article_ID == "Selander_2015" | 
                                                 Article_ID == "SenftBatoh_2015a" |
                                                 Article_ID == "SenftBatoh_2015b" |
                                                 Article_ID == "Hardardottir_2015" |
                                                 Article_ID == "Tammilehto_2015" |
                                                 Article_ID == "Hii_2016" |
                                                 Article_ID == "Wohlrab_2017" |
                                                 Article_ID == "Lema_2017" |
                                                 Article_ID == "Lundholm_2018" |
                                                 Article_ID == "Griffin_2019" |
                                                 Article_ID == "Hii_2019" |
                                                 Article_ID == "Hardardottir_2019a"),
                 
                 control = list(optimizer="optim", optmethod="Nelder-Mead"))

res_cumul_30 <- 
orchaRd::mod_results(mod_cumul_30,
                     mod = "Exp_type",
                     group = "Exp_type",
                     data = data_MA_full)$mod_table

res_cumul_30 <-
data.frame(Author = c("Harðardóttir et al (a)", "Harðardóttir et al (a)"),
           Year = c(2019, 2019),
           Driver = c("Demand", "Resource"),
           Estimate = res_cumul_30$estimate, 
           LCL = res_cumul_30$lowerCL, 
           UCL = res_cumul_30$upperCL,
           N = mod_cumul_30$s.nlevels,
           K = c(mod_cumul_30$g.levels.k[1], mod_cumul_30$g.levels.k[2]),
           row.names = NULL)

res_cumul_30
```

```
##                   Author Year   Driver Estimate       LCL      UCL  N  K
## 1 Harðardóttir et al (a) 2019   Demand 1.470959 1.0159065 1.926010 30 52
## 2 Harðardóttir et al (a) 2019 Resource 1.236597 0.7509924 1.722202 30 37
```

```
# + paper 31
mod_cumul_31 <- rma.mv(yi = delta_RR,
                 V = Var_delta_RR, 
                 mods = ~ 1 + Exp_type, 
                 method = "REML", 
                 test = "t", 
                 random = list(~1 | Article_ID,
                               ~1 + Exp_type| Effect_ID),
                 rho = 0, 
                 struc = "HCS", 
                 data = data_MA_full %>%   filter(Article_ID == "Bechemin_1999" |
                                                 Article_ID == "Guisande_2002" |
                                                 Article_ID ==  "Grzebyk_2003" |
                                                 Article_ID == "Lippemeier_2003" |
                                                 Article_ID == "Leong_2004" |
                                                 Article_ID == "Selander_2006" |
                                                 Article_ID == "Hu_2006"  |
                                                 Article_ID ==  "Murata_2006" | 
                                                 Article_ID ==  "Bergkvist_2008" |
                                                 Article_ID ==  "Selander_2008" |
                                                 Article_ID == "Wohlrab_2010" |
                                                 Article_ID == "Yang_2011a" |
                                                 Article_ID == "Yang_2011b" |
                                                 Article_ID == "Selander_2012" |
                                                 Article_ID == "Lee_2012" |
                                                 Article_ID == "Murata_2012" |
                                                 Article_ID == "Tatters_2013" |
                                                 Article_ID == "VanDeWaal_2013" |
                                                 Article_ID == "Selander_2015" | 
                                                 Article_ID == "SenftBatoh_2015a" |
                                                 Article_ID == "SenftBatoh_2015b" |
                                                 Article_ID == "Hardardottir_2015" |
                                                 Article_ID == "Tammilehto_2015" |
                                                 Article_ID == "Hii_2016" |
                                                 Article_ID == "Wohlrab_2017" |
                                                 Article_ID == "Lema_2017" |
                                                 Article_ID == "Lundholm_2018" |
                                                 Article_ID == "Griffin_2019" |
                                                 Article_ID == "Hii_2019" |
                                                 Article_ID == "Hardardottir_2019a" |
                                                 Article_ID == "Hardardottir_2019b"),
                 
                 control = list(optimizer="optim", optmethod="Nelder-Mead"))

res_cumul_31 <- 
orchaRd::mod_results(mod_cumul_31,
                     mod = "Exp_type",
                     group = "Exp_type",
                     data = data_MA_full)$mod_table

res_cumul_31 <-
data.frame(Author = c("Harðardóttir et al (b)", "Harðardóttir et al (b)"),
           Year = c(2019, 2019),
           Driver = c("Demand", "Resource"),
           Estimate = res_cumul_31$estimate, 
           LCL = res_cumul_31$lowerCL, 
           UCL = res_cumul_31$upperCL,
           N = mod_cumul_31$s.nlevels,
           K = c(mod_cumul_31$g.levels.k[1], mod_cumul_31$g.levels.k[2]),
           row.names = NULL)

res_cumul_31
```

```
##                   Author Year   Driver Estimate       LCL      UCL  N  K
## 1 Harðardóttir et al (b) 2019   Demand 1.490254 1.0494656 1.931042 31 53
## 2 Harðardóttir et al (b) 2019 Resource 1.239736 0.7580002 1.721472 31 37
```

```
# + paper 32
mod_cumul_32 <- rma.mv(yi = delta_RR,
                 V = Var_delta_RR, 
                 mods = ~ 1 + Exp_type, 
                 method = "REML", 
                 test = "t", 
                 random = list(~1 | Article_ID,
                               ~1 + Exp_type| Effect_ID),
                 rho = 0, 
                 struc = "HCS", 
                 data = data_MA_full %>%   filter(Article_ID == "Bechemin_1999" |
                                                 Article_ID == "Guisande_2002" |
                                                 Article_ID ==  "Grzebyk_2003" |
                                                 Article_ID == "Lippemeier_2003" |
                                                 Article_ID == "Leong_2004" |
                                                 Article_ID == "Selander_2006" |
                                                 Article_ID == "Hu_2006"  |
                                                 Article_ID ==  "Murata_2006" | 
                                                 Article_ID ==  "Bergkvist_2008" |
                                                 Article_ID ==  "Selander_2008" |
                                                 Article_ID == "Wohlrab_2010" |
                                                 Article_ID == "Yang_2011a" |
                                                 Article_ID == "Yang_2011b" |
                                                 Article_ID == "Selander_2012" |
                                                 Article_ID == "Lee_2012" |
                                                 Article_ID == "Murata_2012" |
                                                 Article_ID == "Tatters_2013" |
                                                 Article_ID == "VanDeWaal_2013" |
                                                 Article_ID == "Selander_2015" | 
                                                 Article_ID == "SenftBatoh_2015a" |
                                                 Article_ID == "SenftBatoh_2015b" |
                                                 Article_ID == "Hardardottir_2015" |
                                                 Article_ID == "Tammilehto_2015" |
                                                 Article_ID == "Hii_2016" |
                                                 Article_ID == "Wohlrab_2017" |
                                                 Article_ID == "Lema_2017" |
                                                 Article_ID == "Lundholm_2018" |
                                                 Article_ID == "Griffin_2019" |
                                                 Article_ID == "Hii_2019" |
                                                 Article_ID == "Hardardottir_2019a" |
                                                 Article_ID == "Hardardottir_2019b" |
                                                 Article_ID == "Grebner_2019" ),
                 
                 control = list(optimizer="optim", optmethod="Nelder-Mead"))

res_cumul_32 <- 
orchaRd::mod_results(mod_cumul_32,
                     mod = "Exp_type",
                     group = "Exp_type",
                     data = data_MA_full)$mod_table

res_cumul_32 <-
data.frame(Author = c("Grebner et al", "Grebner et al"),
           Year = c(2019, 2019),
           Driver = c("Demand", "Resource"),
           Estimate = res_cumul_32$estimate, 
           LCL = res_cumul_32$lowerCL, 
           UCL = res_cumul_32$upperCL,
           N = mod_cumul_32$s.nlevels,
           K = c(mod_cumul_32$g.levels.k[1], mod_cumul_32$g.levels.k[2]),
           row.names = NULL)

res_cumul_32
```

```
##          Author Year   Driver Estimate       LCL      UCL  N  K
## 1 Grebner et al 2019   Demand 1.538284 1.0921990 1.984370 32 60
## 2 Grebner et al 2019 Resource 1.242182 0.7811239 1.703241 32 37
```

```
# + paper 33
mod_cumul_33 <- rma.mv(yi = delta_RR,
                 V = Var_delta_RR, 
                 mods = ~ 1 + Exp_type, 
                 method = "REML", 
                 test = "t", 
                 random = list(~1 | Article_ID,
                               ~1 + Exp_type| Effect_ID),
                 rho = 0, 
                 struc = "HCS", 
                 data = data_MA_full %>%   filter(Article_ID == "Bechemin_1999" |
                                                 Article_ID == "Guisande_2002" |
                                                 Article_ID ==  "Grzebyk_2003" |
                                                 Article_ID == "Lippemeier_2003" |
                                                 Article_ID == "Leong_2004" |
                                                 Article_ID == "Selander_2006" |
                                                 Article_ID == "Hu_2006"  |
                                                 Article_ID ==  "Murata_2006" | 
                                                 Article_ID ==  "Bergkvist_2008" |
                                                 Article_ID ==  "Selander_2008" |
                                                 Article_ID == "Wohlrab_2010" |
                                                 Article_ID == "Yang_2011a" |
                                                 Article_ID == "Yang_2011b" |
                                                 Article_ID == "Selander_2012" |
                                                 Article_ID == "Lee_2012" |
                                                 Article_ID == "Murata_2012" |
                                                 Article_ID == "Tatters_2013" |
                                                 Article_ID == "VanDeWaal_2013" |
                                                 Article_ID == "Selander_2015" | 
                                                 Article_ID == "SenftBatoh_2015a" |
                                                 Article_ID == "SenftBatoh_2015b" |
                                                 Article_ID == "Hardardottir_2015" |
                                                 Article_ID == "Tammilehto_2015" |
                                                 Article_ID == "Hii_2016" |
                                                 Article_ID == "Wohlrab_2017" |
                                                 Article_ID == "Lema_2017" |
                                                 Article_ID == "Lundholm_2018" |
                                                 Article_ID == "Griffin_2019" |
                                                 Article_ID == "Hii_2019" |
                                                 Article_ID == "Hardardottir_2019a" |
                                                 Article_ID == "Hardardottir_2019b" |
                                                 Article_ID == "Grebner_2019" |
                                                 Article_ID == "Selander_2019" ),
                 
                 control = list(optimizer="optim", optmethod="Nelder-Mead"))

res_cumul_33 <- 
orchaRd::mod_results(mod_cumul_33,
                     mod = "Exp_type",
                     group = "Exp_type",
                     data = data_MA_full)$mod_table

res_cumul_33 <-
data.frame(Author = c("Selander et al", "Selander et al"),
           Year = c(2019, 2019),
           Driver = c("Demand", "Resource"),
           Estimate = res_cumul_33$estimate, 
           LCL = res_cumul_33$lowerCL, 
           UCL = res_cumul_33$upperCL,
           N = mod_cumul_33$s.nlevels,
           K = c(mod_cumul_33$g.levels.k[1], mod_cumul_33$g.levels.k[2]),
           row.names = NULL)

res_cumul_33
```

```
##           Author Year   Driver Estimate       LCL      UCL  N  K
## 1 Selander et al 2019   Demand 1.566655 1.1307319 2.002577 33 61
## 2 Selander et al 2019 Resource 1.245527 0.7864253 1.704630 33 37
```

```
# + paper 34
mod_cumul_34 <- rma.mv(yi = delta_RR,
                 V = Var_delta_RR, 
                 mods = ~ 1 + Exp_type, 
                 method = "REML", 
                 test = "t", 
                 random = list(~1 | Article_ID,
                               ~1 + Exp_type| Effect_ID),
                 rho = 0, 
                 struc = "HCS", 
                 data = data_MA_full %>%   filter(Article_ID == "Bechemin_1999" |
                                                 Article_ID == "Guisande_2002" |
                                                 Article_ID ==  "Grzebyk_2003" |
                                                 Article_ID == "Lippemeier_2003" |
                                                 Article_ID == "Leong_2004" |
                                                 Article_ID == "Selander_2006" |
                                                 Article_ID == "Hu_2006"  |
                                                 Article_ID ==  "Murata_2006" | 
                                                 Article_ID ==  "Bergkvist_2008" |
                                                 Article_ID ==  "Selander_2008" |
                                                 Article_ID == "Wohlrab_2010" |
                                                 Article_ID == "Yang_2011a" |
                                                 Article_ID == "Yang_2011b" |
                                                 Article_ID == "Selander_2012" |
                                                 Article_ID == "Lee_2012" |
                                                 Article_ID == "Murata_2012" |
                                                 Article_ID == "Tatters_2013" |
                                                 Article_ID == "VanDeWaal_2013" |
                                                 Article_ID == "Selander_2015" | 
                                                 Article_ID == "SenftBatoh_2015a" |
                                                 Article_ID == "SenftBatoh_2015b" |
                                                 Article_ID == "Hardardottir_2015" |
                                                 Article_ID == "Tammilehto_2015" |
                                                 Article_ID == "Hii_2016" |
                                                 Article_ID == "Wohlrab_2017" |
                                                 Article_ID == "Lema_2017" |
                                                 Article_ID == "Lundholm_2018" |
                                                 Article_ID == "Griffin_2019" |
                                                 Article_ID == "Hii_2019" |
                                                 Article_ID == "Hardardottir_2019a" |
                                                 Article_ID == "Hardardottir_2019b" |
                                                 Article_ID == "Grebner_2019" |
                                                 Article_ID == "Selander_2019" |
                                                 Article_ID == "Lema_2019"),
                 
                 control = list(optimizer="optim", optmethod="Nelder-Mead"))

res_cumul_34 <- 
orchaRd::mod_results(mod_cumul_34,
                     mod = "Exp_type",
                     group = "Exp_type",
                     data = data_MA_full)$mod_table

res_cumul_34 <-
data.frame(Author = c("Lema et al", "Lema et al"),
           Year = c(2019, 2019),
           Driver = c("Demand", "Resource"),
           Estimate = res_cumul_34$estimate, 
           LCL = res_cumul_34$lowerCL, 
           UCL = res_cumul_34$upperCL,
           N = mod_cumul_34$s.nlevels,
           K = c(mod_cumul_34$g.levels.k[1], mod_cumul_34$g.levels.k[2]),
           row.names = NULL)

res_cumul_34
```

```
##       Author Year   Driver Estimate       LCL      UCL  N  K
## 1 Lema et al 2019   Demand 1.576146 1.1567140 1.995578 34 61
## 2 Lema et al 2019 Resource 1.326341 0.8017004 1.850981 34 44
```

```
# + paper 35
mod_cumul_35 <- rma.mv(yi = delta_RR,
                 V = Var_delta_RR, 
                 mods = ~ 1 + Exp_type, 
                 method = "REML", 
                 test = "t", 
                 random = list(~1 | Article_ID,
                               ~1 + Exp_type| Effect_ID),
                 rho = 0, 
                 struc = "HCS", 
                 data = data_MA_full %>%   filter(Article_ID == "Bechemin_1999" |
                                                 Article_ID == "Guisande_2002" |
                                                 Article_ID ==  "Grzebyk_2003" |
                                                 Article_ID == "Lippemeier_2003" |
                                                 Article_ID == "Leong_2004" |
                                                 Article_ID == "Selander_2006" |
                                                 Article_ID == "Hu_2006"  |
                                                 Article_ID ==  "Murata_2006" | 
                                                 Article_ID ==  "Bergkvist_2008" |
                                                 Article_ID ==  "Selander_2008" |
                                                 Article_ID == "Wohlrab_2010" |
                                                 Article_ID == "Yang_2011a" |
                                                 Article_ID == "Yang_2011b" |
                                                 Article_ID == "Selander_2012" |
                                                 Article_ID == "Lee_2012" |
                                                 Article_ID == "Murata_2012" |
                                                 Article_ID == "Tatters_2013" |
                                                 Article_ID == "VanDeWaal_2013" |
                                                 Article_ID == "Selander_2015" | 
                                                 Article_ID == "SenftBatoh_2015a" |
                                                 Article_ID == "SenftBatoh_2015b" |
                                                 Article_ID == "Hardardottir_2015" |
                                                 Article_ID == "Tammilehto_2015" |
                                                 Article_ID == "Hii_2016" |
                                                 Article_ID == "Wohlrab_2017" |
                                                 Article_ID == "Lema_2017" |
                                                 Article_ID == "Lundholm_2018" |
                                                 Article_ID == "Griffin_2019" |
                                                 Article_ID == "Hii_2019" |
                                                 Article_ID == "Hardardottir_2019a" |
                                                 Article_ID == "Hardardottir_2019b" |
                                                 Article_ID == "Grebner_2019" |
                                                 Article_ID == "Selander_2019" |
                                                 Article_ID == "Lema_2019" |
                                                 Article_ID == "Olesen_2020"),
                 
                 control = list(optimizer="optim", optmethod="Nelder-Mead"))

res_cumul_35 <- 
orchaRd::mod_results(mod_cumul_35,
                     mod = "Exp_type",
                     group = "Exp_type",
                     data = data_MA_full)$mod_table

res_cumul_35 <-
data.frame(Author = c("Olesen et al", "Olesen et al"),
           Year = c(2020, 2020),
           Driver = c("Demand", "Resource"),
           Estimate = res_cumul_35$estimate, 
           LCL = res_cumul_35$lowerCL, 
           UCL = res_cumul_35$upperCL,
           N = mod_cumul_35$s.nlevels,
           K = c(mod_cumul_35$g.levels.k[1], mod_cumul_35$g.levels.k[2]),
           row.names = NULL)

res_cumul_35
```

```
##         Author Year   Driver Estimate       LCL      UCL  N  K
## 1 Olesen et al 2020   Demand 1.573143 1.1547436 1.991543 35 61
## 2 Olesen et al 2020 Resource 1.274446 0.7596084 1.789284 35 46
```

```
# + paper 36
mod_cumul_36 <- rma.mv(yi = delta_RR,
                 V = Var_delta_RR, 
                 mods = ~ 1 + Exp_type, 
                 method = "REML", 
                 test = "t", 
                 random = list(~1 | Article_ID,
                               ~1 + Exp_type| Effect_ID),
                 rho = 0, 
                 struc = "HCS", 
                 data = data_MA_full %>%   filter(Article_ID == "Bechemin_1999" |
                                                 Article_ID == "Guisande_2002" |
                                                 Article_ID ==  "Grzebyk_2003" |
                                                 Article_ID == "Lippemeier_2003" |
                                                 Article_ID == "Leong_2004" |
                                                 Article_ID == "Selander_2006" |
                                                 Article_ID == "Hu_2006"  |
                                                 Article_ID ==  "Murata_2006" | 
                                                 Article_ID ==  "Bergkvist_2008" |
                                                 Article_ID ==  "Selander_2008" |
                                                 Article_ID == "Wohlrab_2010" |
                                                 Article_ID == "Yang_2011a" |
                                                 Article_ID == "Yang_2011b" |
                                                 Article_ID == "Selander_2012" |
                                                 Article_ID == "Lee_2012" |
                                                 Article_ID == "Murata_2012" |
                                                 Article_ID == "Tatters_2013" |
                                                 Article_ID == "VanDeWaal_2013" |
                                                 Article_ID == "Selander_2015" | 
                                                 Article_ID == "SenftBatoh_2015a" |
                                                 Article_ID == "SenftBatoh_2015b" |
                                                 Article_ID == "Hardardottir_2015" |
                                                 Article_ID == "Tammilehto_2015" |
                                                 Article_ID == "Hii_2016" |
                                                 Article_ID == "Wohlrab_2017" |
                                                 Article_ID == "Lema_2017" |
                                                 Article_ID == "Lundholm_2018" |
                                                 Article_ID == "Griffin_2019" |
                                                 Article_ID == "Hii_2019" |
                                                 Article_ID == "Hardardottir_2019a" |
                                                 Article_ID == "Hardardottir_2019b" |
                                                 Article_ID == "Grebner_2019" |
                                                 Article_ID == "Selander_2019" |
                                                 Article_ID == "Lema_2019" |
                                                 Article_ID == "Olesen_2020" |
                                                 Article_ID == "Ryderheim_2021"),
                 
                 control = list(optimizer="optim", optmethod="Nelder-Mead"))

res_cumul_36 <- 
orchaRd::mod_results(mod_cumul_36,
                     mod = "Exp_type",
                     group = "Exp_type",
                     data = data_MA_full)$mod_table

res_cumul_36 <-
data.frame(Author = c("Ryderheim et al", "Ryderheim et al"),
           Year = c(2021, 2021),
           Driver = c("Demand", "Resource"),
           Estimate = res_cumul_36$estimate, 
           LCL = res_cumul_36$lowerCL, 
           UCL = res_cumul_36$upperCL,
           N = mod_cumul_36$s.nlevels,
           K = c(mod_cumul_36$g.levels.k[1], mod_cumul_36$g.levels.k[2]),
           row.names = NULL)

res_cumul_36
```

```
##            Author Year   Driver Estimate       LCL      UCL  N  K
## 1 Ryderheim et al 2021   Demand 1.553850 1.1563647 1.951336 36 64
## 2 Ryderheim et al 2021 Resource 1.296935 0.7990987 1.794772 36 47
```

```
# + paper 37
mod_cumul_37 <- rma.mv(yi = delta_RR,
                 V = Var_delta_RR, 
                 mods = ~ 1 + Exp_type, 
                 method = "REML", 
                 test = "t", 
                 random = list(~1 | Article_ID,
                               ~1 + Exp_type| Effect_ID),
                 rho = 0, 
                 struc = "HCS", 
                 data = data_MA_full %>%   filter(Article_ID == "Bechemin_1999" |
                                                 Article_ID == "Guisande_2002" |
                                                 Article_ID ==  "Grzebyk_2003" |
                                                 Article_ID == "Lippemeier_2003" |
                                                 Article_ID == "Leong_2004" |
                                                 Article_ID == "Selander_2006" |
                                                 Article_ID == "Hu_2006"  |
                                                 Article_ID ==  "Murata_2006" | 
                                                 Article_ID ==  "Bergkvist_2008" |
                                                 Article_ID ==  "Selander_2008" |
                                                 Article_ID == "Wohlrab_2010" |
                                                 Article_ID == "Yang_2011a" |
                                                 Article_ID == "Yang_2011b" |
                                                 Article_ID == "Selander_2012" |
                                                 Article_ID == "Lee_2012" |
                                                 Article_ID == "Murata_2012" |
                                                 Article_ID == "Tatters_2013" |
                                                 Article_ID == "VanDeWaal_2013" |
                                                 Article_ID == "Selander_2015" | 
                                                 Article_ID == "SenftBatoh_2015a" |
                                                 Article_ID == "SenftBatoh_2015b" |
                                                 Article_ID == "Hardardottir_2015" |
                                                 Article_ID == "Tammilehto_2015" |
                                                 Article_ID == "Hii_2016" |
                                                 Article_ID == "Wohlrab_2017" |
                                                 Article_ID == "Lema_2017" |
                                                 Article_ID == "Lundholm_2018" |
                                                 Article_ID == "Griffin_2019" |
                                                 Article_ID == "Hii_2019" |
                                                 Article_ID == "Hardardottir_2019a" |
                                                 Article_ID == "Hardardottir_2019b" |
                                                 Article_ID == "Grebner_2019" |
                                                 Article_ID == "Selander_2019" |
                                                 Article_ID == "Lema_2019" |
                                                 Article_ID == "Olesen_2020" |
                                                 Article_ID == "Ryderheim_2021" |
                                                 Article_ID == "Olesen_2022"),
                 
                 control = list(optimizer="optim", optmethod="Nelder-Mead"))

res_cumul_37 <- 
orchaRd::mod_results(mod_cumul_37,
                     mod = "Exp_type",
                     group = "Exp_type",
                     weights = "prop",
                     data = data_MA_full)$mod_table

res_cumul_37 <-
data.frame(Author = c("Olesen et al", "Olesen et al"),
           Year = c(2022, 2022),
           Driver = c("Demand", "Resource"),
           Estimate = res_cumul_37$estimate, 
           LCL = res_cumul_37$lowerCL, 
           UCL = res_cumul_37$upperCL,
           N = mod_cumul_37$s.nlevels,
           K = c(mod_cumul_37$g.levels.k[1], mod_cumul_37$g.levels.k[2]),
           row.names = NULL)

res_cumul_37
```

```
##         Author Year   Driver Estimate       LCL      UCL  N  K
## 1 Olesen et al 2022   Demand 1.585498 1.1946523 1.976345 37 66
## 2 Olesen et al 2022 Resource 1.299885 0.8115082 1.788262 37 47
```

##### 6.2.1.3 Combine individual analyses into a dataframe with chronologically arranged cumulative effects

```
# Adding all data frames into one
cumul_results <-
bind_rows(res_cumul_1, res_cumul_2, res_cumul_3, res_cumul_4, res_cumul_5, 
          res_cumul_6, res_cumul_7, res_cumul_8, res_cumul_9, res_cumul_10,
          res_cumul_11, res_cumul_12, res_cumul_13, res_cumul_14, res_cumul_15,
          res_cumul_16, res_cumul_17, res_cumul_18, res_cumul_19, res_cumul_20,
          res_cumul_21, res_cumul_22, res_cumul_23, res_cumul_24, res_cumul_25,
          res_cumul_26, res_cumul_27, res_cumul_28, res_cumul_29, res_cumul_30, 
          res_cumul_31, res_cumul_32, res_cumul_33, res_cumul_34, res_cumul_35,
          res_cumul_36, res_cumul_37)


# Create new column by separating Author and Year
cumul_results <- 
  cumul_results %>%  
  
  unite("Study",
         c(Author, Year),
         sep = ", ",
         remove = FALSE)

# Encode this column as a factor
cumul_results$Study <- as_factor(cumul_results$Study)


# Create columns of transformed mean + lower and upper confidence limits
cumul_results <- 
  cumul_results %>%  
  
  mutate(percent_est = round(100*(exp(Estimate)-1),
                             digits = 0),
         
         percent_LCL = round(100*(exp(LCL)-1),
                             digits = 0),
         
         percent_UCL = round(if_else(100*(exp(UCL)-1) < 2000000, #condition
                                     100*(exp(UCL)-1), #if condition is met
                                     Inf), #if condition is not met
                             digits = 0)) 


# Create character column with custom format: [ LowerCL, UpperCL]
cumul_results$percent_conf_int  <- 
paste("[ ", cumul_results$percent_LCL, ", ", cumul_results$percent_UCL, " ]", sep = "")


# ---- Display labels: replace minus with en-dash (Windows-safe) ----
cumul_results <-
  cumul_results %>%
  
  mutate(
    percent_est_label =
      gsub("-", "–", as.character(percent_est)),
    
    percent_conf_int_label =
      gsub("-", "–", percent_conf_int)
  )


# Arrange dataframe by article N (First by year, then alphabetically within years)
cumul_results <- cumul_results %>%  arrange(N)


# ---- Manual study labels
study_label_map <- c("Bechemin et al, 1999" = "Bechemin <i>et al.</i>, 1999",
                     "Guisande et al, 2002" = "Guisande <i>et al.</i>, 2002*",
                     "Grzebyk et al, 2003" = "Grzebyk <i>et al.</i>, 2003",
                     "Lippemeier et al, 2003" = "Lippemeier <i>et al.</i>, 2003",
                     "Leong et al, 2004" = "Leong <i>et al.</i>, 2004",
                     "Selander et al, 2006" = "Selander <i>et al.</i>, 2006",
                     "Hu et al, 2006" = "Hu <i>et al.</i>, 2006",
                     "Murata et al, 2006" = "Murata <i>et al.</i>, 2006",
                     "Bergkvist et al, 2008" = "Bergkvist <i>et al.</i>, 2008",
                     "Selander et al, 2008" = "Selander <i>et al.</i>, 2008*",
                     "Wohlrab et al, 2010" = "Wohlrab <i>et al.</i>, 2010",
                     "Yang et al (a), 2011" = "Yang <i>et al.</i>, 2011<i>a</i>",
                     "Yang et al (b), 2011" = "Yang <i>et al.</i>, 2011<i>b</i>",
                     "Selander et al, 2012" = "Selander <i>et al.</i>, 2012",
                     "Lee et al, 2012" = "Lee <i>et al.</i>, 2012",
                     "Murata et al, 2012" = "Murata <i>et al.</i>, 2012",
                     "Tatters et al, 2013" = "Tatters <i>et al.</i>, 2013",
                     "Van de Waal et al, 2013" = "Van de Waal <i>et al.</i>, 2013",
                     "Selander et al, 2015" = "Selander <i>et al.</i>, 2015",
                     "Senft-Batoh et al (a), 2015" = "Senft-Batoh <i>et al.</i>, 2015<i>a</i>",
                     "Senft-Batoh et al (b), 2015" = "Senft-Batoh <i>et al.</i>, 2015<i>b</i>",
                     "Harðardóttir et al, 2015" = "Harðardóttir <i>et al.</i>, 2015",
                     "Tammilehto et al, 2015" = "Tammilehto <i>et al.</i>, 2015",
                     "Hii et al, 2016" = "Hii <i>et al.</i>, 2016",
                     "Wohlrab et al, 2017" = "Wohlrab <i>et al.</i>, 2017",
                     "Lema et al, 2017" = "Lema <i>et al.</i>, 2017",
                     "Lundholm et al, 2018" = "Lundholm <i>et al.</i>, 2018",
                     "Griffin et al, 2019" = "Griffin <i>et al.</i>, 2019*",
                     "Hii et al, 2019" = "Hii <i>et al.</i>, 2019",
                     "Harðardóttir et al (a), 2019" = "Harðardóttir <i>et al.</i>, 2019<i>a</i>",
                     "Harðardóttir et al (b), 2019" = "Harðardóttir <i>et al.</i>, 2019<i>b</i>",
                     "Grebner et al, 2019" = "Grebner <i>et al.</i>, 2019",
                     "Selander et al, 2019" = "Selander <i>et al.</i>, 2019",
                     "Lema et al, 2019" = "Lema <i>et al.</i>, 2019",
                     "Olesen et al, 2020" = "Olesen <i>et al.</i>, 2020",
                     "Ryderheim et al, 2021" = "Ryderheim <i>et al.</i>, 2021*",
                     "Olesen et al, 2022" = "Olesen <i>et al.</i>, 2022"
                     )

missing_keys <- setdiff(unique(as.character(cumul_results$Study)), names(study_label_map))
if (length(missing_keys) > 0) {
  stop(paste("Missing labels for:\n", paste(missing_keys, collapse = "\n")))
}
```

#### 6.2.2 Plot of cumulative effects over time/as new papers were published

##### 6.2.2.1 Forest plot

```
cumul_forest <-
 cumul_results %>% 
  mutate(Stripe = factor(ifelse(N %% 2 == 0, 1, 0))) %>%  
   
   ggplot(aes(y = fct_reorder(Study, desc(N)), 
              x = Estimate,
              group = Driver)) +
   
  geom_point(aes(x = Estimate,
                 shape = Driver,
                 colour = Driver),
             size = 1.5,
             position = position_dodge(width = 0.6)) +
   
   scale_shape_manual(breaks = c("Resource", "Demand"),
                      values = c(15, 19))+
   
   scale_colour_manual(breaks = c("Resource", "Demand"),
                     values = c("#CDC08C","#78744B"))+
  
   
   scale_x_continuous(breaks = c(-3:8),
                      expand = c(0,0))+
 
   geom_errorbarh(aes(xmin = LCL, 
                     xmax = UCL,
                     colour = Driver), 
                  height = 0,
                  linewidth = 0.5,
                  position = position_dodge(width = 0.6),
                  show.legend = FALSE) +
   
   coord_cartesian(xlim = c(-1, 4.1))+
    
   geom_vline(xintercept = 0, 
              color = "black",
              linetype = "dashed", 
              cex = 0.7, 
              alpha = 0.5) +
   
   labs(x = bquote(LRR^~Delta), 
        y = "") +
   
   geom_stripes() +
 
   theme_classic() +
 
   theme(
     axis.text.y = element_blank(),
     axis.ticks.y = element_blank(),
     legend.position = #c(0.81, 0.035),
                        "top",
     legend.box.background = element_rect(fill = "transparent", 
                                          colour = "transparent"),
     legend.background = element_rect(fill = "transparent", 
                                          colour = "transparent"),
     
     legend.key = element_rect(fill = "transparent"), # Make legend key background transparent
     legend.key.size = unit(3, unit = "mm"),
     legend.title = element_blank(),
     legend.text = element_text(size = 9),
     axis.line.y = element_blank(),
     axis.title.x = element_text(vjust = 0,
                                 size = 10),
     
     legend.spacing.x = unit(0, units = "mm"),
     
     legend.margin = margin(t = -1,
                            r = 1,
                            b = -12, 
                            l = -1,
                            unit = "mm"),
     
     plot.margin = margin(t = 0,
                          r = 0,
                          b = 0, 
                          l = 0, 
                          unit = "cm") ) +
   
    guides(shape = guide_legend(override.aes = list(size = 3)),  # Increase shape size in legend
          colour = guide_legend(override.aes = list(size = 3)))  # Increase color size in legend
```

##### 6.2.2.2 List of author and publication year

```
data_table_author <-
  
  ggplot(data = cumul_results,
         aes(y = fct_reorder(Study, desc(N)))) +
  
  ggtext::geom_richtext(
    aes(x = -0.58,
        label = unname(study_label_map[as.character(Study)]),
        hjust = 0),
    size = 2.5,
    fill = NA,
    label.color = NA
  ) +
  
  geom_text(data = dplyr::filter(cumul_results, Driver == "Resource"),
            aes(x = -0.59,
                label = N,
                hjust = 0),
            size = 2.5) +
  
  geom_text(aes(x = -0.595,
                label = "+",
                hjust = 0),
            size = 2.5) +
  
  scale_x_continuous(position = "top",
                     breaks = c(-0.59, -0.58),
                     labels = c("<i>N</i>", "Author(s), Year")) +
  
  theme_classic() +
  
  coord_cartesian(xlim = c(-0.61, -0.52)) +
  
  theme(
    axis.text.y = element_blank(),
    axis.ticks.y = element_blank(),
    axis.title.y = element_blank(),
    
    axis.text.x = element_markdown(hjust = 0,
                               face = "bold",
                               colour = "black",
                               size = 8),
    
    axis.ticks.x = element_blank(),
    axis.title.x = element_blank(),
    
    axis.line.y = element_blank(),
    axis.line.x.top = element_blank(),
    
    plot.margin = margin(t = 0,
                         r = -2,
                         b = 0,
                         l = -2,
                         unit = "cm")
  )
```

##### 6.2.2.3 List of transformed effect & CIs as percentage increase

```
data_table_values <-
ggplot(data = cumul_results,
         aes(y = fct_reorder(Study, desc(N)))) +
  
   geom_text(aes(x = -0.4 , 
                label = K, 
                hjust = 0),
            size = 2.4) +
  
  geom_text(aes(x = -0.3, 
                label = percent_est_label, 
                hjust = 0),
            size = 2.4) +
  
    geom_text(aes(x = -0.15, 
                label = percent_conf_int_label, 
                hjust = 0),
            size = 2.4) +
  
  
  facet_wrap( ~ fct_reorder(Driver, Driver))+

  scale_x_continuous(position = "top", 
                     breaks = c( -0.4, -0.27, -0.15), 
                     labels = c("<i>k</i>", "%", "[95% CI]")) +
  
  coord_cartesian(xlim = c(-0.4, 0.2))+

  theme_classic() +
    
  theme(
    axis.text.y = element_blank(),
    axis.ticks.y = element_blank(),
    axis.title.y = element_blank(),
    
    axis.text.x = ggtext::element_markdown(hjust = 0,
                                           face = "bold",
                                           colour = "black",
                                           size = 8),
    
    axis.ticks.x = element_blank(),
    axis.title.x = element_blank(),
    
    strip.background = element_blank(),
    
    strip.text = element_blank(),
    
    axis.line.y = element_blank(),
    axis.line.x.top = element_blank(),
    
    plot.margin = margin(t = 0,
                         r = 0,
                         b = 0,
                         l = -2, 
                         unit = "cm"))
```

##### 6.2.2.4 Adding the three components into one plot

```
data_table_author + cumul_forest + data_table_values+
  patchwork::plot_layout(ncol = 3, 
              widths = c(2, 1.9, 2.5))
```

```
# ggsave(path = "figures_manuscript", "Cumul_plot_final.png", width = 160, height = 200, units = "mm", dpi=1500)
```

### 6.3 Statistical analyses of bias

#### 6.3.1 Data wrangling

```
data_MA_full <-
  data_MA_full %>% 
  mutate(inv_effective_n = (n_control+n_ex)/(n_control*n_ex), # inverse effective sample size
         sqrt_inv_eff_n = sqrt(inv_effective_n), # square root of inverse sample size
         year_c = scale(Year, scale = FALSE)) # centered year
```

#### 6.3.2 Analysis

```
# Publication bias

publication_bias = rma.mv(yi = delta_RR,
                    V = Var_delta_RR,
                    mod = ~0 + Exp_type + Phytoplankton_group + sqrt_inv_eff_n, 
                    random = rand, 
                    method = "REML",
                    test = "t",
                    data = data_MA_full)
summary.rma(publication_bias)
```

```
## 
## Multivariate Meta-Analysis Model (k = 113; method: REML)
## 
##    logLik   Deviance        AIC        BIC       AICc   
## -165.4888   330.9776   342.9776   359.1257   343.8012   
## 
## Variance Components:
## 
##             estim    sqrt  nlvls  fixed                factor 
## sigma^2.1  0.3308  0.5751     37     no            Article_ID 
## sigma^2.2  0.9465  0.9729    113     no  Article_ID/Effect_ID 
## 
## Test for Residual Heterogeneity:
## QE(df = 109) = 9701.5323, p-val < .0001
## 
## Test of Moderators (coefficients 1:4):
## F(df1 = 4, df2 = 109) = 31.8526, p-val < .0001
## 
## Model Results:
## 
##                                      estimate      se     tval   df    pval 
## Exp_typeDemand                         2.1630  1.3512   1.6008  109  0.1123 
## Exp_typeResource                       2.0047  1.3980   1.4340  109  0.1544 
## Phytoplankton_groupPseudo-nitzschia    1.3211  0.2954   4.4730  109  <.0001 
## sqrt_inv_eff_n                        -1.3301  1.7638  -0.7541  109  0.4524 
##                                        ci.lb   ci.ub      
## Exp_typeDemand                       -0.5150  4.8411      
## Exp_typeResource                     -0.7661  4.7754      
## Phytoplankton_groupPseudo-nitzschia   0.7358  1.9065  *** 
## sqrt_inv_eff_n                       -4.8258  2.1657      
## 
## ---
## Signif. codes:  0 '***' 0.001 '**' 0.01 '*' 0.05 '.' 0.1 ' ' 1
```

```
r2_ml(publication_bias)
```

```
##    R2_marginal R2_conditional 
##      0.2370411      0.4346208
```

No statistical evidence of publication bias.

```
# Time-lag bias

time_lag_bias = rma.mv(yi = delta_RR,
                          V = Var_delta_RR,
                          mod = ~ Exp_type + Phytoplankton_group + year_c, 
                          random = rand, 
                          method = "REML",
                          test = "t",
                          data = data_MA_full)
time_lag_bias
```

```
## 
## Multivariate Meta-Analysis Model (k = 113; method: REML)
## 
## Variance Components:
## 
##             estim    sqrt  nlvls  fixed                factor 
## sigma^2.1  0.3595  0.5996     37     no            Article_ID 
## sigma^2.2  0.9071  0.9524    113     no  Article_ID/Effect_ID 
## 
## Test for Residual Heterogeneity:
## QE(df = 109) = 9917.2638, p-val < .0001
## 
## Test of Moderators (coefficients 2:4):
## F(df1 = 3, df2 = 109) = 8.1014, p-val < .0001
## 
## Model Results:
## 
##                                      estimate      se     tval   df    pval 
## intrcpt                                1.0616  0.2131   4.9812  109  <.0001 
## Exp_typeResource                      -0.2797  0.2737  -1.0217  109  0.3092 
## Phytoplankton_groupPseudo-nitzschia    1.6352  0.3429   4.7691  109  <.0001 
## year_c                                -0.0541  0.0298  -1.8147  109  0.0723 
##                                        ci.lb   ci.ub      
## intrcpt                               0.6392  1.4840  *** 
## Exp_typeResource                     -0.8222  0.2629      
## Phytoplankton_groupPseudo-nitzschia   0.9556  2.3148  *** 
## year_c                               -0.1131  0.0050    . 
## 
## ---
## Signif. codes:  0 '***' 0.001 '**' 0.01 '*' 0.05 '.' 0.1 ' ' 1
```

```
r2_ml(time_lag_bias)
```

```
##    R2_marginal R2_conditional 
##      0.2567011      0.4676823
```

No statistical evidence of time-lag bias.

```
# Combined model, as suggested by Nakagawa and colleagues (2022)

bias_all = rma.mv(yi = delta_RR,
                       V = Var_delta_RR,
                       mod = ~ 0 + 
                         sqrt_inv_eff_n + 
                         year_c +
                         Exp_type + 
                         Phytoplankton_group,
                          
                       random = rand, 
                       method = "REML",
                       test = "t",
                       data = data_MA_full)

bias_all
```

```
## 
## Multivariate Meta-Analysis Model (k = 113; method: REML)
## 
## Variance Components:
## 
##             estim    sqrt  nlvls  fixed                factor 
## sigma^2.1  0.3708  0.6089     37     no            Article_ID 
## sigma^2.2  0.9126  0.9553    113     no  Article_ID/Effect_ID 
## 
## Test for Residual Heterogeneity:
## QE(df = 108) = 9701.3932, p-val < .0001
## 
## Test of Moderators (coefficients 1:5):
## F(df1 = 5, df2 = 108) = 25.1489, p-val < .0001
## 
## Model Results:
## 
##                                      estimate      se     tval   df    pval 
## sqrt_inv_eff_n                        -0.1858  1.9050  -0.0975  108  0.9225 
## year_c                                -0.0533  0.0322  -1.6558  108  0.1007 
## Exp_typeDemand                         1.2021  1.4766   0.8141  108  0.4174 
## Exp_typeResource                       0.9298  1.5483   0.6005  108  0.5494 
## Phytoplankton_groupPseudo-nitzschia    1.6369  0.3480   4.7036  108  <.0001 
##                                        ci.lb   ci.ub      
## sqrt_inv_eff_n                       -3.9618  3.5902      
## year_c                               -0.1170  0.0105      
## Exp_typeDemand                       -1.7249  4.1290      
## Exp_typeResource                     -2.1393  3.9988      
## Phytoplankton_groupPseudo-nitzschia   0.9471  2.3267  *** 
## 
## ---
## Signif. codes:  0 '***' 0.001 '**' 0.01 '*' 0.05 '.' 0.1 ' ' 1
```

```
r2_ml(bias_all)
```

```
##    R2_marginal R2_conditional 
##      0.2559909      0.4709498
```

No evidence of publication or time-lag bias.

## 7. Final orchard plots

### 7.1 Data preparation

```
## Mod_13
res_mod_13_data  <-
  orchaRd::mod_results(mod_13,
                       mod = "Exp_type",
                       group = "Exp_type",
                       data = data_MA_full)$data %>%  
    rename(Driver = stdy)


### Mod_16

res_mod_16_data  <-
  orchaRd::mod_results(mod_16,
                       mod = "Exp_type",
                       group = "Phytoplankton_group",
                       by = "Phytoplankton_group",
                       data = data_MA_full)$data %>%  
    rename(Genus = stdy)
```

### 7.2 Moderator: Driver

#### 7.2.1 Manually constructing the plot

##### 7.2.1.1 Horisontal

```
x_pos <- 5.8
annot_size <- 3

ggplot(data = res_mod_13, aes(x = estimate, y = Driver)) +
  
  # 95 %prediction interval (PI): twigs
  ggplot2::geom_errorbar(aes(xmin = lowerPR, 
                             xmax = upperPR,
                             y = Driver,),
                         width = 0, 
                         show.legend = FALSE, 
                         size = 1.2, 
                         alpha = 0.8) +
  
  # 95 %CI: branches
  ggplot2::geom_errorbar(aes(xmin = lowerCL, 
                              xmax = upperCL,
                              y = Driver),  
                          width = 0, 
                          position = position_dodge(width = 0.6),
                          show.legend = FALSE, 
                          size = 4, 
                          alpha = 0.9) +
  
  # Dashed line at 0
  ggplot2::geom_vline(xintercept = 0, linetype = 2, colour = "black", alpha = 0.5) +
  
  
  # pieces of fruit (bee-swarm and bubbles)
  ggbeeswarm::geom_quasirandom(data = res_mod_13_data, 
                               aes(y = moderator, 
                                   x = yi, 
                                   size = 1/(sqrt(vi))), 
                               colour = "grey10",
                               alpha= 0.2,
                               width = 0.4) +
  
# Mean effect (diamond inside trunks)
  ggplot2::geom_point(aes(x = estimate),
                      shape = 23,
                      fill = "white",
                      size = 2.8,
                      position = position_dodge(width = 0.6)) +
  
  scale_x_continuous(limits = c(-1.6, 6.8),
                     breaks = seq(-1, 6, by = 1),
                     minor_breaks = NULL) +  
  
  guides(colour = guide_legend(override.aes = list(size=4)))+

  
  ggplot2::labs(x = bquote(LRR^~Delta),
                y = "",
                colour = "Genus", 
                size = "Precision (1/SE)") +
  
  ggplot2::theme_bw() +
  
  ggplot2::theme(legend.position= c(0.98, 0.01), 
                 legend.justification = c(1, 0),
                 legend.direction = "horizontal",
                 legend.margin = margin(t = -3,
                                        r = 0,
                                        b = -0.4,
                                        l = 0,
                                        unit = "mm"),
                 legend.key.size = unit(2, "mm"),
                 legend.background = element_rect(fill='white', 
                                                  colour = 'white'), #transparent legend bg
                 
                 legend.box.background = element_rect(fill='white', 
                                                      colour = 'white'), #transparent legend panel))) 
                 
                 legend.key = element_rect(fill = 'transparent'), # Make legend key background transparent
          plot.margin = margin(t = 1,
                             r = 1,
                             b = 2,
                             l = -4,
                             unit = "mm"),
          
          legend.title = element_text(size = 8.5,),
          legend.text = element_text(size = 8, face = "plain"),
          panel.background = element_rect(fill = "white"),
          plot.background = element_rect(fill = "white",
                                                colour = "white"),
          panel.border = element_blank(),
          axis.line = element_line(colour = "black"),
          axis.text.y = element_text(size = 11, 
                                            colour ="black", 
                                            hjust = 0.5, 
                                            angle = 90),
          axis.text.x = element_text(size = 10, 
                                            colour ="black", 
                                            hjust = 0.5),
          axis.title.y = element_text(size = 12, 
                                            colour ="black", 
                                            hjust = 0.5,
                                            vjust = 3,
                                            angle = 90),
          panel.grid.major.y = element_line(colour = "grey98"),
          panel.grid.major.x = element_line(colour = "grey98"))+

  
   annotate(geom = "label", #Estimates demand
           y = 1.4, 
           x = x_pos,
           fill = "transparent",
           colour = "black",
           label.size = NA,
           label = paste0(round(100*((exp(res_mod_13$estimate[1]))-1),0), "% increase", 
                          "\n", 
                          "[95% CI: ", round(100*((exp(res_mod_13$lowerCL[1]))-1),0),
                          " - ",
                          round(100*((exp(res_mod_13$upperCL[1]))-1),0), "]",  
                          "\n",
                          "[95% PI: ", round(100*((exp(res_mod_13$lowerPR[1]))-1),0), 
                          " - ", 
                          round(100*((exp(res_mod_13$upperPR[1]))-1),0), "]"),
           parse = FALSE,
           size = annot_size) +
  

  
  annotate(geom = "label", # Estimates Resource
           y = 2.42, 
           x = x_pos,
           fill = "transparent",
           colour = "black",
           label.size = NA,
           label = paste0(round(100*((exp(res_mod_13$estimate[2]))-1),0), "% increase", 
                          "\n", 
                          "[95% CI: ", round(100*((exp(res_mod_13$lowerCL[2]))-1),0),
                          " - ",
                          round(100*((exp(res_mod_13$upperCL[2]))-1),0), "]",  
                          "\n",
                          "[95% PI: ", round(100*((exp(res_mod_13$lowerPR[2]))-1),0), 
                          " - ", 
                          round(100*((exp(res_mod_13$upperPR[2]))-1),0), "]"),
           parse = FALSE,
           size = annot_size)+
  
  
# k (N) label — Demand (italics only for k and N)
annotate(geom = "label",
         y = 1.16,
         x = x_pos,
         fill = "transparent",
         colour = "black",
         label.size = NA,

         label = "italic(k)~'(' * italic(N) * ') = 66 (21)'",  # plotmath
         parse = TRUE,                                         # parse plotmath

         size = annot_size) +


# k (N) label — Resource (italics only for k and N)
annotate(geom = "label",
         y = 2.2,
         x = x_pos,
         fill = "transparent",
         colour = "black",
         label.size = NA,

         label = "italic(k)~'(' * italic(N) * ') = 47 (19)'",  # plotmath
         parse = TRUE,                                         # parse plotmath

         size = annot_size)
```

```
# ggsave(path = "figures_manuscript", "orchard_manual_driver_hori.tiff", width = 110, height =  100, units = "mm", dpi=900)
```

##### 7.2.1.2 Vertical

```
y_pos <- 6.3
annot_size <- 2.8

ggplot(data = res_mod_13, aes(x = Driver, y = estimate)) +
  
  # 95 %prediction interval (PI): twigs
  ggplot2::geom_errorbar(aes(ymin = lowerPR, 
                              ymax = upperPR,
                              x = Driver),
                          width = 0, 
                          show.legend = FALSE, 
                          size = 1.2, 
                          alpha = 1) +
  
  # 95 %CI: branches
  ggplot2::geom_errorbar(aes(ymin = lowerCL, 
                              ymax = upperCL,
                              x = Driver),  
                          width = 0, 
                          position = position_dodge(width = 0.6),
                          show.legend = FALSE, 
                          size = 4, 
                          alpha = 1) +
  
    # pieces of fruit (bee-swarm and bubbles)
  ggbeeswarm::geom_quasirandom(data = res_mod_13_data, 
                               aes(y = yi, 
                                   x = moderator, 
                                   size = 1/(sqrt(vi))), 
                               colour = "grey10",
                               alpha= 0.2,
                               width = 0.2,)+ 
  
  
  
  # Dashed line at 0
  ggplot2::geom_hline(yintercept = 0, 
                      linetype = 2, 
                      colour = "black", 
                      alpha = 0.5) +
  
# creating dots for truncks
  ggplot2::geom_point(aes(y = estimate),
                      shape = 23,
                      fill = "white",
                      size = 3,
                      position = position_dodge(width = 0.6)) +

    

  ggplot2::theme_bw() +
  
  ggplot2::theme(legend.position= c(0.75, 0.01), 
                 legend.justification = c(1, 0),
                 legend.key.size = unit(0.5, "mm"),
                 legend.margin = margin(t = -3,
                                        r = 0,
                                        b = -0.4,
                                        l = 0,
                                        unit = "mm"),
                 legend.background = element_rect(fill='transparent', 
                                                  colour = 'transparent'), #transparent legend bg
                 
                 legend.box.background = element_rect(fill='transparent', 
                                                      colour = 'transparent'), #transparent legend panel))) 
          plot.margin = margin(t = 0,
                             r = 0,
                             b = -3,
                             l = 4,
                             unit = "mm"))+
  
  ggplot2::theme(legend.title = element_text(size = 9),
                 legend.text = element_text(size = 8),
                 legend.direction = "horizontal",
                 legend.background = element_blank(),
                 panel.background = element_rect(fill = "white"),
                 plot.background = element_rect(fill = "white",
                                                colour = "white"),
                 panel.border = element_blank(),
                 axis.line = element_line(colour = "black"))+

  ggplot2::labs(y = bquote(LRR^~Delta),
                x = "",
                colour = "Genus", 
                size = "Precision (1/SE)") +
  ggplot2::theme(axis.text.y = element_text(size = 10, 
                                            colour ="black", 
                                            hjust = 0.5, 
                                            angle = 0),
                 
                 axis.text.x = element_text(size = 12, 
                                            colour ="black", 
                                            hjust = 0.5),
                 
                 axis.title.y = element_text(size = 13, 
                                            colour ="black", 
                                            hjust = 0.5,
                                            vjust = 3,
                                            angle = 90),
          panel.grid.major.y = element_line(colour = "grey98"),
          panel.grid.major.x = element_line(colour = "grey98"))+
  
  scale_y_continuous(limits = c(-2, 6.5),
                     breaks = seq(-1, 6, by = 1),
                     minor_breaks = NULL) +
  
   annotate(geom = "label", #Estimates demand
           x = 0.85, 
           y = y_pos,
           fill = "white",
           colour = "black",
           label.size = NA,
           label = paste0(round(100*((exp(res_mod_13$estimate[1]))-1),0), "% increase", 
                          "\n", 
                          "[95% CI: ", round(100*((exp(res_mod_13$lowerCL[1]))-1),0),
                          " - ",
                          round(100*((exp(res_mod_13$upperCL[1]))-1),0), "]",  
                          "\n",
                          "[95% PI: ", round(100*((exp(res_mod_13$lowerPR[1]))-1),0), 
                          " - ", 
                          round(100*((exp(res_mod_13$upperPR[1]))-1),0), "]"),
           parse = FALSE,
           size = annot_size) +
  
  annotate(geom = "label", ## K (n) Demand
           x = 0.85, 
           y = y_pos - 0.85, 
           fill = "white",
           colour = "black",
           label.size = NA,
           label = "k (n) = 66 (21)", 
           color = "black", 
           parse = FALSE, 
           size = annot_size) +
  
  annotate(geom = "label", # Estimates Resource
           x = 1.72, 
           y = y_pos,
           fill = "white",
           colour = "black",
           label.size = NA,
           label = paste0(round(100*((exp(res_mod_13$estimate[2]))-1),0), "% increase", 
                          "\n", 
                          "[95% CI: ", round(100*((exp(res_mod_13$lowerCL[2]))-1),0),
                          " - ",
                          round(100*((exp(res_mod_13$upperCL[2]))-1),0), "]",  
                          "\n",
                          "[95% PI: ", round(100*((exp(res_mod_13$lowerPR[2]))-1),0), 
                          " - ", 
                          round(100*((exp(res_mod_13$upperPR[2]))-1),0), "]"),
           parse = FALSE,
           size = annot_size)+
  
  
  annotate(geom = "label", # K (n) Resource
           x = 1.72, 
           y = y_pos - 0.85, 
           fill = "white",
           colour = "black",
           label.size = NA,
           label = "k (n) = 47 (19)", 
           color = "black", 
           parse = FALSE, 
           size = annot_size)
```

```
# ggsave(path = "figures_manuscript", "orchard_manual_driver_vert.tiff", width = 110, height = 100, units = "mm", dpi=700)
```

#### 7.2.2 Using `orchard_plot`

```
y_pos <- 5.6
annot_size <- 2.5

orchard_plot(mod_13, 
             mod = "Exp_type",
             data = data_MA_full,
             group = "Phytoplankton_group",
             xlab = bquote(LRR^~Delta),
             angle = 0,
             alpha = 0.3,
             cb = TRUE,
             k = FALSE,
             g = FALSE,
             branch.size = 2,
             twig.size = 0.7,
             trunk.size = 5.7,
             colour = TRUE,
             fill = TRUE,
             legend.pos = "bottom.right")+
    
  scale_y_continuous(
    limits = c(-1.5, 7),
    breaks = c(-1:7),
    minor_breaks = NULL,
   # labels = c("-65", "0", "170", "640", "1900", "5 350", "14 750", "40 200")
    )+
  
  
  annotate(geom = "label", #Estimates demand
           x = 1.4, 
           y = y_pos,
           fill = "white",
           colour = "black",
           label.size = NA,
           label = paste0(round(100*((exp(res_mod_13$estimate[1]))-1),0), "% increase", 
                          "\n", 
                          "[95% CI: ", round(100*((exp(res_mod_13$lowerCL[1]))-1),0),
                          " - ",
                          round(100*((exp(res_mod_13$upperCL[1]))-1),0), "]",  
                          "\n",
                          "[95% PI: ", round(100*((exp(res_mod_13$lowerPR[1]))-1),0), 
                          " - ", 
                          round(100*((exp(res_mod_13$upperPR[1]))-1),0), "]"),
           parse = FALSE,
           size = annot_size) +
  
  annotate(geom = "label", ## K (n) Demand
           x = 1.23, 
           y = y_pos, 
           fill = "white",
           colour = "black",
           label.size = NA,
           label = "K (N) = 66 (21)", 
           color = "black", 
           parse = FALSE, 
           size = annot_size) +
  
  annotate(geom = "label", # Estimates Resource
           x = 2.4, 
           y = y_pos,
           fill = "white",
           colour = "black",
           label.size = NA,
           label = paste0(round(100*((exp(res_mod_13$estimate[2]))-1),0), "% increase", 
                          "\n", 
                          "[95% CI: ", round(100*((exp(res_mod_13$lowerCL[2]))-1),0),
                          " - ",
                          round(100*((exp(res_mod_13$upperCL[2]))-1),0), "]",  
                          "\n",
                          "[95% PI: ", round(100*((exp(res_mod_13$lowerPR[2]))-1),0), 
                          " - ", 
                          round(100*((exp(res_mod_13$upperPR[2]))-1),0), "]"),
           parse = FALSE,
           size = annot_size)+
  
  
  annotate(geom = "label", # K (n) Resource
           x = 2.23, 
           y = y_pos, 
           fill = "white",
           colour = "black",
           label.size = NA,
           label = "K (N = 47 (19)", 
           color = "black", 
           parse = FALSE, 
           size = annot_size)  +
  
   
  scale_fill_manual(breaks = c("Pseudo-nitzschia", "Alexandrium"),
                    values = c("#1B9E77", "#D95F02"))+
  
  scale_colour_manual(breaks = c("Pseudo-nitzschia", "Alexandrium"),
                    values = c("#1B9E77", "#D95F02"))+
  
  #theme_classic()+

  
  theme(axis.text.x  = element_text(colour = "black",
                                    size = 8),
        
        axis.title.x = element_text(colour = "black",
                                    size = 9,
                                    vjust = -0.2),
        
        axis.text.y = element_text(colour = "black",
                                   size = 9, 
                                   angle = 90),
        
        legend.position = c(0.98,0.005),
        legend.margin = margin(t = 0,
                               r = 0,
                               b = 0,
                               l = 0,
                               unit = "mm"),
        
        plot.margin = margin(t = 0,
                             r = 0,
                             b = 2,
                             l = -4,
                             unit = "mm"),
        
        panel.border = element_blank(),
        
        axis.line = element_line(colour = "black"),
        
        legend.text = element_text(size = 7,
                                   colour = "black"),
        
        legend.title = element_text(size = 7,
                                    colour = "black"),
        
        legend.background = element_rect(fill='white', 
                                         colour = 'transparent'), #transparent legend bg
        
        legend.box.background = element_rect(fill='transparent', 
                                             colour = 'transparent')) #transparent legend panel))
```

```
# ggsave(path = "figures_manuscript", "orchard_driver.tiff", width = 100, height = 100, units = "mm", dpi=700)
```

### 7.3 Moderator : Driver \* Phytoplankton genus

#### 7.3.1 Manually constructing the plot

##### 7.3.1.1 Horisontal

```
x_pos <- 6
annot_size <- 3

ggplot(data = res_mod_16, aes(y = Driver, x = estimate)) +
    
  # 95 %prediction interval (PI): twigs
  ggplot2::geom_errorbar(aes(xmin = lowerPR, 
                              xmax = upperPR,
                              y = Driver,
                              group = Genus),
                          width = 0, 
                          position = position_dodge(width = 0.6),
                          show.legend = FALSE, 
                          size = 1.2, 
                          alpha = 0.8) +
  
  # 95 %CI: branches
  ggplot2::geom_errorbar(aes(xmin = lowerCL, 
                              xmax = upperCL,
                              y = Driver,
                              group = Genus),  
                          width = 0, 
                          position = position_dodge(width = 0.6),
                          show.legend = FALSE, 
                          size = 4, 
                          alpha = 0.9) +
  
  # Estimate points for trunks
  ggplot2::geom_point(aes(x = estimate,
                          shape = Genus,
                          fill = Genus),
                      size = 3.5,
                      position = position_dodge(width = 0.6)) +
  
  # Dashed line at 0
  ggplot2::geom_vline(xintercept = 0, 
                      linetype = 2, 
                      colour = "black", 
                      alpha = 0.5) +
  
  
  # pieces of fruit (bee-swarm and bubbles)
  ggbeeswarm::geom_quasirandom(data = res_mod_16_data, 
                               aes(x = yi, 
                                   y = moderator, 
                                   size = 1/(sqrt(vi)), 
                                   colour = Genus,
                                   fill = Genus), 
                               alpha= 0.3,
                               width = 0.2,
                               dodge.width = 0.6)+
  
  scale_color_manual(breaks = c("Alexandrium", "Pseudo-nitzschia"),
                     values=c("#D55E00", "#009E73")) +
  
  
  scale_shape_manual(values = c(22,23))+
  scale_fill_manual(breaks = c("Alexandrium", "Pseudo-nitzschia"),
                     values=c("#D55E00", "#009E73")) +
    

 ggplot2::theme_bw() +
  
  ggplot2::theme(legend.position= c(0.98, 0.005), 
                 legend.justification = c(1, 0),
                 legend.margin = margin(t = -3,
                                        r = 0,
                                        b = -0.4,
                                        l = 0,
                                        unit = "mm"),
                 legend.key.size = unit(2, "mm"),
                 legend.background = element_rect(fill='white', 
                                                  colour = 'white'), #transparent legend bg
                 
                 legend.box.background = element_rect(fill='white', 
                                                      colour = 'white'), #transparent legend panel))) 
          plot.margin = margin(t = 1,
                             r = 1,
                             b = 2,
                             l = -4,
                             unit = "mm"))+
  
  ggplot2::theme(legend.title = element_text(size = 8.5,),
                 legend.text = element_text(size = 8, face = "plain"),
                 legend.direction = "horizontal",
                 legend.background = element_blank(),
                 panel.background = element_rect(fill = "white"),
                 plot.background = element_rect(fill = "white",
                                                colour = "white"),
                 panel.border = element_blank(),
                 axis.line = element_line(colour = "black"))+

  ggplot2::labs(x = bquote(LRR^~Delta),
                y = "",
                colour = "Genus", 
                size = "Precision (1/SE)") +
  guides(colour = guide_legend(override.aes = list(size = 3.5),    # optional: legend key size
      
                               label.theme  = ggplot2::element_text(face = "italic")  # Genus labels italic
    ),
    
         size = guide_legend(label.theme  = ggplot2::element_text(face = "plain")   # Precision labels plain
    ))+
  
  ggplot2::theme(axis.text.y = element_text(size = 11, 
                                            colour ="black", 
                                            hjust = 0.5, 
                                            angle = 90),
                 
                 axis.text.x = element_text(size = 10, 
                                            colour ="black", 
                                            hjust = 0.5),
                 
                 axis.title.y = element_text(size = 12, 
                                            colour ="black", 
                                            hjust = 0.5,
                                            vjust = 3,
                                            angle = 90),
                 panel.grid.major.x = element_line(colour = "grey98"),
                 panel.grid.major.y = element_line(colour = "grey98"))+
  
  scale_x_continuous(limits = c(-1.6, 6.8),
                     breaks = seq(-1, 6, by = 1),
                     minor_breaks = NULL)+
  
  annotate(geom = "label", #Estimates Demand Alexandrium
           y = 0.88, 
           x = x_pos,
           fill = "transparent",
           colour = "black",
           label.size = NA,
           label = paste0(round(100*((exp(res_mod_16$estimate[1]))-1),0), "% increase", 
                          "\n", 
                          "[95% CI: ", round(100*((exp(res_mod_16$lowerCL[1]))-1),0), 
                         # "%",
                          " - ",
                          round(100*((exp(res_mod_16$upperCL[1]))-1),0), "]",  
                          "\n",
                          "[95% PI: ", round(100*((exp(res_mod_16$lowerPR[1]))-1),0), 
                         # "%",
                          " - ", 
                          round(100*((exp(res_mod_16$upperPR[1]))-1),0), "]"
                         ),
           parse = FALSE,
           size = annot_size) +
  
    annotate(geom = "label",  # k (n) Demand Alexandrium
         y = 0.7,
         x = x_pos,
         fill = "transparent",
         colour = "black",
         label.size = NA,

         label = "italic(k)~'(' * italic(N) * ') = 46 (14)'",
         parse = TRUE,

         size = annot_size) +
  
  annotate(geom = "label", #Estimates Demand Pseudo-nitzschia
           y = 1.35, 
           x = x_pos, 
           fill = "transparent",
           colour = "black",
           label.size = NA,
           label = paste0(round(100*((exp(res_mod_16$estimate[3]))-1),0), "% increase", 
                          "\n", 
                          "[95% CI: ", round(100*((exp(res_mod_16$lowerCL[3]))-1),0), 
                         # "%",
                          " - ",
                          round(100*((exp(res_mod_16$upperCL[3]))-1),0), "]",  
                          "\n",
                          "[95% PI: ", round(100*((exp(res_mod_16$lowerPR[3]))-1),0), 
                         # "%",
                          " - ", 
                          round(100*((exp(res_mod_16$upperPR[3]))-1),0), "]"
                         ),
           parse = FALSE,
           size = annot_size) +
  
  
  annotate(geom = "label",  # k (n) Demand Pseudo-nitzschia
         y = 1.17,
         x = x_pos,
         fill = "transparent",
         colour = "black",
         label.size = NA,

         label = "italic(k)~'(' * italic(N) * ') = 20 (8)'",
         parse = TRUE,

         size = annot_size) +
  
  annotate(geom = "label", #Estimates resource Alexandrium
           y = 1.87, 
           x = x_pos, 
           fill = "transparent",
           colour = "black",
           label.size = NA,
           label = paste0(round(100*((exp(res_mod_16$estimate[2]))-1),0), "% increase", 
                          "\n", 
                          "[95% CI: ", round(100*((exp(res_mod_16$lowerCL[2]))-1),0), 
                         # "%",
                          " - ",
                          round(100*((exp(res_mod_16$upperCL[2]))-1),0), "]",  
                          "\n",
                          "[95% PI: ", round(100*((exp(res_mod_16$lowerPR[2]))-1),0), 
                         # "%",
                          " - ", 
                          round(100*((exp(res_mod_16$upperPR[2]))-1),0), "]"
                         ),
           parse = FALSE,
           size = annot_size) +
  
  annotate(geom = "label",  # k (n) Resource Alexandrium
         y = 1.69,
         x = x_pos,
         fill = "transparent",
         colour = "black",
         label.size = NA,

         label = "italic(k)~'(' * italic(N) * ') = 29 (16)'",
         parse = TRUE,

         size = annot_size) +
  
  annotate(geom = "label", #Estimates resource Pseudo-nitzschia
           y = 2.4, 
           x = x_pos, 
           fill = "transparent",
           colour = "black",
           label.size = NA,
           label = paste0(round(100*((exp(res_mod_16$estimate[4]))-1),0), "% increase", 
                          "\n", 
                          "[95% CI: ", round(100*((exp(res_mod_16$lowerCL[4]))-1),0), 
                         # "%",
                          " - ",
                          round(100*((exp(res_mod_16$upperCL[4]))-1),0), "]",  
                          "\n",
                          "[95% PI: ", round(100*((exp(res_mod_16$lowerPR[4]))-1),0), 
                         # "%",
                          " - ", 
                          round(100*((exp(res_mod_16$upperPR[4]))-1),0), "]"
                         ),
           parse = FALSE,
           size = annot_size) +
  
  annotate(geom = "label",  # k (n) Resource Pseudo-nitzschia
         y = 2.22,
         x = x_pos,
         fill = "transparent",
         colour = "black",
         label.size = NA,

         label = "italic(k)~'(' * italic(N) * ') = 18 (3)'",
         parse = TRUE,

         size = annot_size)
```

```
#  ggsave(path = "figures_manuscript", "orchard_manual_DriverGenus_hori.png", width = 120, height = 120, units = "mm", dpi=1500)
```

##### 7.3.1.2 Vertical

```
y_pos <- 6.3 
annot_size <- 2.8

ggplot(data = res_mod_16, aes(x = Driver, y = estimate)) +
  
  # 95 %prediction interval (PI): twigs
  ggplot2::geom_errorbar(aes(ymin = lowerPR, 
                              ymax = upperPR,
                              x = Driver,
                              group = Genus),
                          width = 0, 
                          position = position_dodge(width = 0.6),
                          show.legend = FALSE, 
                          size = 1.2, 
                          alpha = 1) +
  
  # 95 %CI: branches
  ggplot2::geom_errorbar(aes(ymin = lowerCL, 
                              ymax = upperCL,
                              x = Driver,
                              group = Genus),  
                          width = 0, 
                          position = position_dodge(width = 0.6),
                          show.legend = FALSE, 
                          size = 3.5, 
                          alpha = 1) +
  
    # pieces of fruit (bee-swarm and bubbles)
  ggbeeswarm::geom_quasirandom(data = res_mod_16_data, 
                               aes(y = yi, 
                                   x = moderator, 
                                   size = 1/(sqrt(vi)), 
                                   colour = Genus,
                                   fill = Genus), 
                               alpha= 0.2,
                               width = 0.2,
                               dodge.width = 0.6) +
  
  scale_color_manual(breaks = c("Alexandrium", "Pseudo-nitzschia"),
                     values=c("#D55E00", "#009E73")) +
  
  
  # Dashed line at 0
  ggplot2::geom_hline(yintercept = 0, linetype = 2, colour = "black", alpha = 0.5) +
  
# Estimate points for trunks
  ggplot2::geom_point(aes(y = estimate,
                          shape = Genus,
                          fill = Genus),
                      size = 3,
                      position = position_dodge(width = 0.6)) +
  
  scale_shape_manual(values = c(22,23))+
  scale_fill_manual(breaks = c("Alexandrium", "Pseudo-nitzschia"),
                     values=c("#D55E00", "#009E73")) +
    

  ggplot2::theme_bw() +
  #ggplot2::guides(fill = "none", colour = "none") +
  ggplot2::theme(legend.position= c(0.6, 0.01), 
                 legend.justification = c(1, 0),
                 legend.margin = margin(t = -3,
                                        r = 0,
                                        b = -0.4,
                                        l = 0,
                                        unit = "mm"),
                 legend.background = element_rect(fill='white', 
                                                  colour = 'white'), #transparent legend bg
                 
                 legend.box.background = element_rect(fill='white', 
                                                      colour = 'white'), #transparent legend panel))) 
          plot.margin = margin(t = 0,
                             r = 0,
                             b = -3,
                             l = 4,
                             unit = "mm"))+
  
  ggplot2::theme(legend.title = element_text(size = 9)) +
  ggplot2::theme(legend.direction="horizontal") +
  ggplot2::theme(legend.background = element_blank()) +
  ggplot2::labs(y = bquote(LRR^~Delta),
                x = "",
                colour = "Genus", 
                size = "Precision (1/SE)") +
  ggplot2::theme(axis.text.y = element_text(size = 10, 
                                            colour ="black", 
                                            hjust = 0.5, 
                                            angle = 0),
                 
                 axis.text.x = element_text(size = 12, 
                                            colour ="black", 
                                            hjust = 0.5),
                 
                 axis.title.y = element_text(size = 13, 
                                            colour ="black", 
                                            hjust = 0.5,
                                            vjust = 2,
                                            angle = 90),
                 panel.grid.major.y = element_line(colour = "grey98"),
                 panel.grid.major.x = element_blank())+
  
  scale_y_continuous(limits = c(-2, 6.8),
                     breaks = seq(-1, 7, by = 1),
                     minor_breaks = NULL)+
  
  annotate(geom = "label", #Estimates Demand Alexandrium
           x = 0.7, 
           y = y_pos,
           fill = "white",
           colour = "black",
           label.size = NA,
           label = paste0(round(100*((exp(res_mod_16$estimate[1]))-1),0), "% increase", 
                          "\n", 
                          "[95% CI: ", round(100*((exp(res_mod_16$lowerCL[1]))-1),0), 
                         # "%",
                          " - ",
                          round(100*((exp(res_mod_16$upperCL[1]))-1),0), "]",  
                          "\n",
                          "[95% PI: ", round(100*((exp(res_mod_16$lowerPR[1]))-1),0), 
                         # "%",
                          " - ", 
                          round(100*((exp(res_mod_16$upperPR[1]))-1),0), "]"
                         ),
           parse = FALSE,
           size = annot_size)+ 
  
    annotate(geom = "label", ## K (n) Demand Alexandrium
           x = 0.7, 
           y = y_pos - 0.85, 
           fill = "white",
           colour = "black",
           label.size = NA,
           label = "K (N) = 14 (46)", 
           color = "black", 
           parse = FALSE, 
           size = annot_size)+
  
  annotate(geom = "label", #Estimates Demand Pseudo-nitzschia
           x = 1.25, 
           y = y_pos, 
           fill = "white",
           colour = "black",
           label.size = NA,
           label = paste0(round(100*((exp(res_mod_16$estimate[3]))-1),0), "% increase", 
                          "\n", 
                          "[95% CI: ", round(100*((exp(res_mod_16$lowerCL[3]))-1),0), 
                         # "%",
                          " - ",
                          round(100*((exp(res_mod_16$upperCL[3]))-1),0), "]",  
                          "\n",
                          "[95% PI: ", round(100*((exp(res_mod_16$lowerPR[3]))-1),0), 
                         # "%",
                          " - ", 
                          round(100*((exp(res_mod_16$upperPR[3]))-1),0), "]"
                         ),
           parse = FALSE,
           size = annot_size) +
  
  
  annotate(geom = "label", ## K (n) Demand Pseudo-nitzschia
           x = 1.25, 
           y = y_pos-0.85, 
           fill = "white",
           colour = "black",
           label.size = NA,
           label = "K (N) = 8 (20)", 
           size = annot_size) +
  
  annotate(geom = "label", #Estimates resource Alexandrium
           x = 1.8, 
           y = y_pos, 
           fill = "white",
           colour = "black",
           label.size = NA,
           label = paste0(round(100*((exp(res_mod_16$estimate[2]))-1),0), "% increase", 
                          "\n", 
                          "[95% CI: ", round(100*((exp(res_mod_16$lowerCL[2]))-1),0), 
                         # "%",
                          " - ",
                          round(100*((exp(res_mod_16$upperCL[2]))-1),0), "]",  
                          "\n",
                          "[95% PI: ", round(100*((exp(res_mod_16$lowerPR[2]))-1),0), 
                         # "%",
                          " - ", 
                          round(100*((exp(res_mod_16$upperPR[2]))-1),0), "]"
                         ),
           parse = FALSE,
           size = annot_size) +
  
  annotate(geom = "label", ## K (n) resource Alexandrium
           x = 1.8, 
           y = y_pos-0.85, 
           fill = "white",
           colour = "black",
           label.size = NA,
           label = "K (N) = 16 (29)", 
           color = "black", 
           parse = FALSE, 
           size = annot_size)+
  
  annotate(geom = "label", #Estimates resource Pseudo-nitzschia
           x = 2.38, 
           y = y_pos, 
           fill = "white",
           colour = "black",
           label.size = NA,
           label = paste0(round(100*((exp(res_mod_16$estimate[4]))-1),0), "% increase", 
                          "\n", 
                          "[95% CI: ", round(100*((exp(res_mod_16$lowerCL[4]))-1),0), 
                         # "%",
                          " - ",
                          round(100*((exp(res_mod_16$upperCL[4]))-1),0), "]",  
                          "\n",
                          "[95% PI: ", round(100*((exp(res_mod_16$lowerPR[4]))-1),0), 
                         # "%",
                          " - ", 
                          round(100*((exp(res_mod_16$upperPR[4]))-1),0), "]"
                         ),
           parse = FALSE,
           size = annot_size) +
  
  annotate(geom = "label", ## K (n) resource Pseudo-nitzschia
           x = 2.38, 
           y = y_pos-0.85, 
           fill = "white",
           colour = "black",
           label.size = NA,
           label = "K (N) = 3 (18)", 
           size = annot_size)
```

```
#  ggsave(path = "figures_manuscript", "orchard_manual_DriverGenus_vert.tiff", width = 150, height = 130, units = "mm", dpi=700)
```

#### 7.3.2 Using `orchard_plot`

```
# OrchaRd plot using the package
y_pos <- 5.75 
annot_size <- 2.5 

orchard_plot(mod_16, 
             mod = "Exp_type",
             by = "Phytoplankton_group",
             data = data_MA_full,
             group = "Phytoplankton_group",
             xlab = bquote(LRR^~Delta),
             angle = 0,
             alpha = 0.3,
             cb = TRUE,
             k = FALSE,
             g = FALSE,
             branch.size = 2,
             twig.size = 0.7,
             trunk.size = 5.9,
             colour = TRUE,
             fill = TRUE,
             legend.pos = "bottom.right")+

    
  scale_y_continuous(
    limits = c(-1.7, 7),
    breaks = c(-1:7),
    minor_breaks = NULL,
    #labels = c("65", "0", "170", "640", "1900", "5 350", "14 750", "40 200", "")
    )+
  
   
  scale_fill_manual(breaks = c("Pseudo-nitzschia", "Alexandrium"),
                    values = c("#1B9E77", "#D95F02"))+
  
  scale_colour_manual(breaks = c("Pseudo-nitzschia", "Alexandrium"),
                    values = c("#1B9E77", "#D95F02"))+
  
  #theme_classic()+

  
  theme(axis.text.x  = element_text(colour = "black",
                                    size = 8),
        
        axis.title.x = element_text(colour = "black",
                                    size = 9,
                                    vjust = -0.2),
        
        axis.text.y = element_text(colour = "black",
                                   size = 9, 
                                   angle = 90),
        
        legend.position = c(0.98,0.91),
        legend.margin = margin(t = -5,
                               r = 0,
                               b = -0.4,
                               l = 0,
                               unit = "mm"),
        
        legend.box.margin = margin(t = 6,
                                   unit = "mm"),
        
        plot.margin = margin(t = 0,
                             r = 0,
                             b = 2,
                             l = -4,
                             unit = "mm"),
        
        panel.border = element_blank(),
        
        axis.line = element_line(colour = "black"),
        
        legend.text = element_text(size = 7,
                                   colour = "black"),
        
        legend.title = element_text(size = 7,
                                    colour = "black"),
      
       # legend.position = c(0.31,0.005),
       # legend.direction = "vertical",
        
        legend.background = element_rect(fill='white', 
                                         colour = 'white'), #transparent legend bg
        
        legend.box.background = element_rect(fill='white', 
                                             colour = 'white'))+ #transparent legend panel))

    annotate(geom = "label", #Estimates Demand Alexandrium
           x = 0.73, 
           y = y_pos,
           fill = "white",
           colour = "black",
           label.size = NA,
           label = paste0(round(100*((exp(res_mod_16$estimate[1]))-1),0), "% increase", 
                          "\n", 
                          "[95% CI: ", round(100*((exp(res_mod_16$lowerCL[1]))-1),0), 
                         # "%",
                          " - ",
                          round(100*((exp(res_mod_16$upperCL[1]))-1),0), "]",  
                          "\n",
                          "[95% PI: ", round(100*((exp(res_mod_16$lowerPR[1]))-1),0), 
                         # "%",
                          " - ", 
                          round(100*((exp(res_mod_16$upperPR[1]))-1),0), "]"
                         ),
           parse = FALSE,
           size = annot_size) +
  
  annotate(geom = "label", ## K (n) Demand Alexandrium
           x = 0.6, 
           y = y_pos, 
           fill = "white",
           colour = "black",
           label.size = NA,
           label = "K (N) = 14 (46)", 
           color = "black", 
           parse = FALSE, 
           size = annot_size) +
  
      annotate(geom = "label", #Estimates resource Alexandrium
           x = 1.84, 
           y = y_pos, 
           fill = "white",
           colour = "black",
           label.size = NA,
           label = paste0(round(100*((exp(res_mod_16$estimate[2]))-1),0), "% increase", 
                          "\n", 
                          "[95% CI: ", round(100*((exp(res_mod_16$lowerCL[2]))-1),0), 
                         # "%",
                          " - ",
                          round(100*((exp(res_mod_16$upperCL[2]))-1),0), "]",  
                          "\n",
                          "[95% PI: ", round(100*((exp(res_mod_16$lowerPR[2]))-1),0), 
                         # "%",
                          " - ", 
                          round(100*((exp(res_mod_16$upperPR[2]))-1),0), "]"
                         ),
           parse = FALSE,
           size = annot_size) +
  
  annotate(geom = "label", ## K (n) resource Alexandrium
           x = 1.71, 
           y = y_pos, 
           fill = "white",
           colour = "black",
           label.size = NA,
           label = "K (N) = 16 (29)", 
           color = "black", 
           parse = FALSE, 
           size = annot_size) +
  
  
      annotate(geom = "label", #Estimates Demand Pseudo-nitzschia
           x = 1.31, 
           y = y_pos, 
           fill = "white",
           colour = "black",
           label.size = NA,
           label = paste0(round(100*((exp(res_mod_16$estimate[3]))-1),0), "% increase", 
                          "\n", 
                          "[95% CI: ", round(100*((exp(res_mod_16$lowerCL[3]))-1),0), 
                         # "%",
                          " - ",
                          round(100*((exp(res_mod_16$upperCL[3]))-1),0), "]",  
                          "\n",
                          "[95% PI: ", round(100*((exp(res_mod_16$lowerPR[3]))-1),0), 
                         # "%",
                          " - ", 
                          round(100*((exp(res_mod_16$upperPR[3]))-1),0), "]"
                         ),
           parse = FALSE,
           size = annot_size) +
  
  annotate(geom = "label", ## K (n) Demand Pseudo-nitzschia
           x = 1.18, 
           y = y_pos, 
           fill = "white",
           colour = "black",
           label.size = NA,
           label = "K (N) = 8 (20)", 
           size = annot_size)+
  
        annotate(geom = "label", #Estimates resource Pseudo-nitzschia
           x = 2.25, 
           y = y_pos, 
           fill = "white",
           colour = "black",
           label.size = NA,
           label = paste0(round(100*((exp(res_mod_16$estimate[4]))-1),0), "% increase", 
                          "\n", 
                          "[95% CI: ", round(100*((exp(res_mod_16$lowerCL[4]))-1),0), 
                         # "%",
                          " - ",
                          round(100*((exp(res_mod_16$upperCL[4]))-1),0), "]",  
                          "\n",
                          "[95% PI: ", round(100*((exp(res_mod_16$lowerPR[4]))-1),0), 
                         # "%",
                          " - ", 
                          round(100*((exp(res_mod_16$upperPR[4]))-1),0), "]"
                         ),
           parse = FALSE,
           size = annot_size) +
  
  annotate(geom = "label", ## K (n) resource Pseudo-nitzschia
           x = 2.12, 
           y = y_pos, 
           fill = "white",
           colour = "black",
           label.size = NA,
           label = "K (N) = 3 (18)", 
           size = annot_size)
```

```
# ggsave(path = "figures_manuscript", "orchard_driver_genus.tiff", width = 120, height = 130, units = "mm", dpi=700)
```

## 8. Bar chart of screened papers

### 8.1 Load data

```
# import data
data_screened_papers <- read_delim("data_screened.csv", 
                                   delim = ";", 
                                   escape_double = FALSE, 
                                   trim_ws = TRUE)
```

### 8.2 Construct the plot

#### 8.2.1 Vertical

```
data_screened_papers %>% 
  pivot_longer(cols = c("Resource", "Demand", "Both"), 
               names_to = "Type", 
               values_to = "Count") %>% 
  ggplot(aes(x = Year, y = Count, fill = Type)) + 
  geom_col(
    #colour = "white", 
    #linewidth = 0.05
    ) +
  scale_fill_manual(values = c("grey30", "#78744B", "#CDC08C")) +  # Custom colors
  

  # Corrected y-axis scale
  scale_y_continuous(expand = c(0, 0),
                     limits = c(0, 100),
                     breaks = seq(0, 100, by = 20)) +  # Corrected breaks
  
  # Corrected x-axis scale
  scale_x_reverse(expand = c(0,0),
                     breaks = seq(1991, 2022, by = 1)) +  # Corrected breaks
  


  theme_classic()+
  labs(
       fill = "",
       x = "",
       y = "Count")+
  theme(legend.position = c(0.55,0.95),
        axis.text.x = element_text(colour = "black",
                                   size = 11,
                                   vjust = 0.4),
        axis.title.x = element_text(size = 12, 
                                    colour = "black"),
        
        axis.text.y = element_text(colour = "black",
                                   size = 10,
                                   vjust = 0.4),
        
        legend.text = element_text(size = 10,
                                   colour = "black",
                                   vjust = 0.6),
        
        legend.key.size = unit(5, units = "mm"),
        
        legend.direction = "horizontal",

        plot.margin = unit(c(t = 0.5,
                             r = 0.75,
                             b = 0.2,
                             l = 0), 
                           units = "cm"))+
  coord_flip()+
  guides(fill = guide_legend(reverse = TRUE))  # Reverse the legend order
```

```
# ggsave(path = "figures_manuscript", "barchart_screened_papers_vert.tiff", width = 95, height = 130, units = "mm", dpi=700)
```

#### 8.2.2 Horizontal

```
data_screened_papers %>% 
  pivot_longer(cols = c("Resource", "Demand", "Both"), 
               names_to = "Type", 
               values_to = "Count") %>% 
  ggplot(aes(x = Year, y = Count, fill = Type)) + 
  geom_col(
    #colour = "white", 
    #linewidth = 0.05
    ) +
  scale_fill_manual(values = c("grey30", "#78744B", "#CDC08C")) +  # Custom colors
  


  # Corrected y-axis scale
  scale_y_continuous(position = "right",
                     expand = c(0, 0),
                     limits = c(0, 100),
                     breaks = seq(0, 100, by = 20),  # Corrected breaks
                     ) +  
  
  # Corrected x-axis scale
  scale_x_continuous(expand = c(0,0),
                     breaks = seq(1992, 2022, by = 2)) +  # Corrected breaks
  
  


  theme_classic()+
  labs(
       fill = "",
       x = "",
       y = "Count")+
  theme(legend.position = c(0.1,0.92),
        axis.text.x = element_text(colour = "black",
                                   size = 10,
                                   vjust = 0.6,
                                   #angle = 45
                                   ),
        
        axis.title.y = element_text(size = 11, 
                                    colour = "black",
                                    hjust = 0.5, 
                                    vjust = 0.1),
        
        axis.text.y = element_text(colour = "black",
                                   size = 10,
                                   vjust = 0.4),
        
        legend.text = element_text(size = 9,
                                   colour = "black",
                                   vjust = 0.6),

        
        legend.key.size = unit(5, units = "mm"),
       

        plot.margin = unit(c(t = 0.5,
                             r = 0.4,
                             b = -0.3,
                             l = 0.5), 
                           units = "cm"))
```

```
# ggsave(path = "figures_manuscript", "barchart_screened_papers_hori.png", width = 180, height = 70, units = "mm", dpi=900)
```

## 9. Supplementary figures

### 9.1 Phytoplankton genus

```
data_MA_full %>%
  group_by(Phytoplankton_group) %>% 
  count(Phytoplankton_group) %>% 
  summarise(sum = sum(n))
```

```
## # A tibble: 2 × 2
##   Phytoplankton_group   sum
##   <fct>               <int>
## 1 Alexandrium            75
## 2 Pseudo-nitzschia       38
```

```
data_MA_full %>% 
  count(Phytoplankton_group) %>%  # Count occurrences of each Exp_type
  mutate(Phytoplankton_group = fct_reorder(Phytoplankton_group, n, .desc = FALSE),  # Reorder by count (descending)
         percentage = n / sum(n) * 100) %>%  # Calculate percentages
  ggplot(aes(x = Phytoplankton_group, y = n, fill = Phytoplankton_group)) + 
  geom_bar(stat = "identity", width = 0.7) +  # Use `identity` for precomputed counts
  geom_text(aes(label = paste0(round(percentage, 0), "%")),  # Add percentage labels
            hjust = -0.2, size = 4, color = "black") +  # Adjust horizontal position and size
  scale_fill_cyclical(values = c("grey40", "grey80")) +
  scale_y_continuous(expand = c(0,0),
                     limits = c(0,100),
                     breaks = c(0, 20, 40, 60, 80, 100))+
  
    # Apply custom labels with ggtext formatting
  scale_x_discrete(labels = c(
    "Alexandrium" = "*Alexandrium*",
    "Pseudo-nitzschia" = "*Pseudo-nitzschia*"))+
  
  labs(title = "Phytoplankton genus: *k* = 113",
       x = "",
       y = "Effect size count (*k*)") +
  coord_flip() +  # Flip the coordinates
  theme_classic() +
  theme(text = element_text(size = 12.5, colour = "black"),
        axis.text = element_markdown(size = 11, colour = "black"),
        axis.title = element_text(size = 12, colour = "black"),
        title = element_markdown(size = 10, colour = "black"),
        plot.title = element_markdown(),
        
        plot.margin = unit(c(0.2, 0.7, 0.2, 0), "cm"))
```

```
# ggsave(path = "figures_supplementary", "1_Supp_desc_genus.png", width = 100, height = 35, units = "mm", dpi=700)
```

### 9.2 Driver

```
data_MA_full %>% 
  count(Exp_type, Phytoplankton_group) %>%  # Count occurrences of each Exp_type and group
  group_by(Exp_type) %>%
  mutate(total_n = sum(n)) %>%  # Total count for each Exp_type
  ungroup() %>%
  mutate(
    overall_percentage = total_n / sum(total_n/2) * 100,  # Percentage for the entire bar
    Exp_type = fct_reorder(Exp_type, total_n, .desc = FALSE)  # Reorder by total counts
  ) %>%
  ggplot(aes(x = Exp_type, y = n, fill = Phytoplankton_group)) + 
  geom_bar(stat = "identity", width = 0.7) +  # Stacked bar chart
  geom_text(data = . %>% distinct(Exp_type, total_n, overall_percentage),  # Use distinct data for percentages
            aes(x = Exp_type, y = total_n, label = paste0(round(overall_percentage, 0), "%")), 
            inherit.aes = FALSE, hjust = -0.2, size = 4, color = "black") +  # Place text outside the bar
  scale_fill_manual(values = c("grey40", "grey80"),
                    labels = c("Alexandrium" = "*Alexandrium*",
                               "Pseudo-nitzschia" = "*Pseudo-nitzschia*")) +
  scale_y_continuous(expand = c(0, 0),
                     limits = c(0, 80), 
                     breaks = seq(0, 80, by = 20)) +
  
  
  labs(title = "Driver: *k* = 113",
       x = "",
       y = "Effect size count (*k*)",
       fill = "") +
  coord_flip() +  # Flip the coordinates
  theme_classic() +
  theme(text = element_text(size = 12.5, colour = "black"),
        axis.text = element_text(size = 12, colour = "black"),
        axis.title = element_markdown(size = 12, colour = "black"), 
        plot.title = element_markdown(),
        title = element_text(size = 10, colour = "black"),
        legend.position = c("bottom"),
        legend.text = element_markdown(size = 10, colour = "black"),
        legend.key.size = unit(12, 'pt'), # Size of legend keys
        plot.margin = unit(c(0.2, 0.7, 0.2, 1.4), "cm"))
```

```
# ggsave(path = "figures_supplementary", "2_Supp_desc_driver.png", width = 100, height = 45, units = "mm", dpi=700)
```

### 9.3 Phytoplankton strain

```
data_MA_full %>%
  count(Phytoplankton_strain) %>% 
  summarise(sum = sum(n))
```

```
##   sum
## 1 113
```

```
data_MA_full %>%
  count(Phytoplankton_strain, .drop = FALSE) %>%  # Include NA values
  mutate(Phytoplankton_strain = fct_reorder(
           fct_explicit_na(Phytoplankton_strain, na_level = "Missing"), 
           n, 
           .desc = FALSE
         ),  # Explicitly handle NA and reorder
         percentage = n / sum(n) * 100) %>%  # Calculate percentages
  ggplot(aes(x = Phytoplankton_strain, y = n, fill = Phytoplankton_strain)) + 
  geom_bar(stat = "identity", width = 0.7) +  # Use `identity` for precomputed counts
  geom_text(aes(label = paste0(round(percentage, 0), "%")),  # Add percentage labels
            hjust = -0.2, size = 4, color = "black") +  # Adjust text position and size
  scale_fill_cyclical(values = c("grey40", "grey80")) +
  scale_y_continuous(expand = c(0, 0),
                     limits = c(0, 30),
                     breaks = c(0, 10, 20, 30)) +
  labs(title = "Phytoplankton strain: *k* = 113",
       x = "",
       y = "Effect size count (*k*)") +
  coord_flip() +  # Flip the coordinates

  theme_classic() +
  theme(text = element_text(size = 12.5, colour = "black"),
        axis.text = element_text(size = 12, colour = "black"),
        axis.title = element_markdown(size = 12, colour = "black"), 
        plot.title = element_markdown(),        
        title = element_text(size = 10, colour = "black"),
        plot.margin = unit(c(0.2, 0.7, 0.2, 0.5), "cm"))
```

```
# ggsave(path = "figures_supplementary", "3_Supp_desc_PhytoStrain.png", width = 100, height = 200, units = "mm", dpi=700)
```

### 9.4 Phytoplankton species

```
data_MA_full %>%
  group_by(Phytoplankton_species) %>% 
  count(Phytoplankton_species) %>% 
  summarise(sum = sum(n))
```

```
## # A tibble: 9 × 2
##   Phytoplankton_species          sum
##   <fct>                        <int>
## 1 Alexandrium catenella           12
## 2 Alexandrium fundyense            9
## 3 Alexandrium minutum             37
## 4 Alexandrium tamarense           17
## 5 Pseudo-nitzschia australis       6
## 6 Pseudo-nitzschia fraudulenta     5
## 7 Pseudo-nitzschia obtusa          2
## 8 Pseudo-nitzschia pungens         5
## 9 Pseudo-nitzschia seriata        20
```

```
data_MA_full %>%
  count(Phytoplankton_species, .drop = FALSE) %>%  # Include NA values
  mutate(
    # Handle NA values explicitly
    Phytoplankton_species = fct_reorder(
      fct_explicit_na(Phytoplankton_species, na_level = "Missing"), 
      n, 
      .desc = FALSE
    ),
    percentage = n / sum(n) * 100  # Calculate percentages
  ) %>% 
  ggplot(aes(x = Phytoplankton_species, y = n, fill = Phytoplankton_species)) + 
  geom_bar(stat = "identity", width = 0.7) +  # Use `identity` for precomputed counts
  geom_text(aes(label = paste0(round(percentage, 0), "%")),  # Add percentage labels
            hjust = -0.2, size = 4, color = "black") +  # Adjust text position and size

  scale_fill_cyclical(values = c("grey40", "grey80")) +
  scale_y_continuous(expand = c(0, 0),
                     limits = c(0, 50),
                     breaks = c(0, 10, 20, 30, 40, 50)) +
  labs(title = "Phytoplankton species: *k* = 113",
       x = "",
       y = "Effect size count (*k*)") +
  coord_flip() +  # Flip the coordinates
  theme_classic() +
  theme(text = element_text(size = 12.5, colour = "black"),
        axis.text = element_text(size = 12, colour = "black"),
        axis.text.y = element_markdown(size = 12, colour = "black", face = "italic"),
        axis.title = element_markdown(size = 12, colour = "black"), 
        plot.title = element_markdown(),        
        title = element_text(size = 10, colour = "black"),
        plot.margin = unit(c(0.2, 0.7, 0.2, 0.5), "cm"))
```

```
# ggsave(path = "figures_supplementary", "4_Supp_desc_PhytoSpecies.png", width = 140, height = 65, units = "mm", dpi=700)
```

### 9.5 Zooplankton species

```
data_MA_full %>%
  group_by(Zooplankton_species) %>% 
  count(Zooplankton_species) %>% 
  summarise(sum = sum(n))
```

```
## # A tibble: 14 × 2
##    Zooplankton_species      sum
##    <fct>                  <int>
##  1 Acartia clausi             4
##  2 Acartia hudsonica          6
##  3 Acartia tonsa              7
##  4 Calanus finmarchicus       8
##  5 Calanus helgolandicus      1
##  6 Calanus hyperboreus        1
##  7 Calanus sp                 3
##  8 Centropages hamatus        3
##  9 Centropages typicus       11
## 10 Eurytemora herdmani        2
## 11 Oithona similis            1
## 12 Paraeuchaeta norvegica     1
## 13 Pseudocalanus sp           2
## 14 <NA>                      63
```

```
data_MA_full %>%
  filter(!is.na(Zooplankton_species)) %>%  # Exclude NA values
  count(Zooplankton_species, .drop = FALSE) %>%  # Count occurrences
mutate(
    # Handle NA values explicitly
    Zooplankton_species = fct_reorder(
      fct_explicit_na(Zooplankton_species, na_level = "Missing"), 
      n, 
      .desc = FALSE
    ),
    percentage = n / sum(n) * 100  # Calculate percentages
  ) %>% 
  ggplot(aes(x = Zooplankton_species, y = n, fill = Zooplankton_species)) + 
  geom_bar(stat = "identity", width = 0.7) +  # Use `identity` for precomputed counts
  geom_text(aes(label = paste0(round(percentage, 1), "%")),  # Add percentage labels
            hjust = -0.2, size = 4, color = "black") +  # Adjust text position and size
  scale_fill_cyclical(values = c("grey40", "grey80")) +
  scale_y_continuous(expand = c(0, 0),
                     limits = c(0, 15),
                     breaks = c(0, 3, 6, 9, 12, 15)) +
  
    scale_x_discrete(labels = c("Acartia clausi" = "*Acartia clausi*",
                              "Acartia hudsonica" = "*Acartia hudsonica*",
                              "Acartia tonsa" = "*Acartia tonsa*",
                              "Calanus finmarchicus" = "*Calanus finmarchicus*",
                              "Calanus helgolandicus" = "*Calanus helgolandicus*",
                              "Calanus hyperboreus" = "*Calanus hyperboreus*",
                              "Calanus sp" = "*Calanus* sp.",
                              "Centropages hamatus" = "*Centropages hamatus*",
                              "Centropages typicus" = "*Centropages typicus*",
                              "Eurytemora herdmani" = "*Eurytemora herdmani*",
                              "Oithona similis" = "*Oithona similis*",
                              "Paraeuchaeta norvegica" = "*Paraeuchaeta norvegica*",
                              "Pseudocalanus sp" = "*Pseudocalanus* sp."))+
  labs(
       title = paste(
         "Zooplankton species: *k* =", 
         sum(data_MA_full %>% 
               filter(!is.na(Zooplankton_species)) %>%  # Exclude NA values again
               count(Zooplankton_species) %>% 
               pull(n))  # Dynamically calculate total
       ),
       x = "",
       y = "Effect size count (*k*)") +
  coord_flip() +  # Flip the coordinates
  theme_classic() +
  theme(text = element_text(size = 12.5, colour = "black"),
        axis.text = element_text(size = 12, colour = "black"),
        axis.text.y = element_markdown(size = 12, colour = "black"),
        axis.title = element_markdown(size = 12, colour = "black"), 
        plot.title = element_markdown(),
        title = element_text(size = 10, colour = "black"),
        plot.margin = unit(c(0.2, 0.7, 0.2, 0.2), "cm"))
```

```
# ggsave(path = "figures_supplementary", "5_Supp_desc_Zoo_Species.png", width = 140, height = 90, units = "mm", dpi=700)
```

### 9.6 Year

```
grand_total_year <- data_MA_full %>%
  filter(!is.na(Year)) %>%
  count(Year, Exp_type) %>%
  pull(n) %>%
  sum()

data_MA_full %>%
  filter(!is.na(Year)) %>%  # Exclude NA values for Year
  count(Year, Exp_type) %>%  # Count occurrences for each Year and Exp_type
  complete(Year = seq(min(Year), max(Year)), Exp_type, fill = list(n = 0)) %>%  # Add missing years and fill with 0
  group_by(Year) %>%
  mutate(
    Year = fct_rev(factor(Year)),  # Reverse the order of Year
    year_total = sum(n),  # Total for the year
    year_percentage = year_total / grand_total_year * 100  # Year total as percentage of grand total
  ) %>%
  ggplot(aes(x = Year, y = n, fill = Exp_type)) +  # Stack bars by Exp_type
  geom_bar(stat = "identity", width = 0.7) +  # Use `identity` for precomputed counts
  geom_text(data = . %>% group_by(Year) %>% summarize(year_total = unique(year_total), 
                                                      year_percentage = unique(year_percentage)),  # Summary for labels
            aes(x = Year, y = year_total, label = paste0(round(year_percentage, 1), "%")),
            inherit.aes = FALSE, hjust = -0.3, size = 4, color = "black") +  # Add total percentage per year
  scale_y_continuous(expand = c(0, 0),
                     limits = c(0, 30),
                     breaks = c(0, 5, 10, 15, 20, 25, 30)) + 
  scale_fill_manual(values = c("grey40", "grey80"))+
  labs(
       title = paste(
         "Publication year: *k* =", 
         grand_total_year  # Use grand total for the title
       ),
       x = "",
       y = "Effect size count (*k*)",
       fill = "") +  # Add legend title for Exp_type
  coord_flip() +  # Flip the coordinates
  theme_classic() +
  theme(text = element_text(size = 12.5, colour = "black"),
        axis.text = element_text(size = 12, colour = "black"),
        axis.title = element_markdown(size = 12, colour = "black"), 
        plot.title = element_markdown(),
        title = element_text(size = 10, colour = "black"),
        plot.margin = unit(c(0.2, 0.7, 0.2, 2.2), "cm"),
        legend.key.size = unit(12, 'pt'), # Size of legend keys
        legend.position = c(0.8, 0.2))
```

```
# ggsave(path = "figures_supplementary", "6_Supp_desc_Year.png", width = 100, height = 140, units = "mm", dpi=700)
```

### 9.7 Sample size

#### 9.7.1 Ordered by sample size

```
data_MA_full %>%
  filter(!is.na(Article_ID)) %>%  # Exclude rows without Article_ID
  group_by(Article_ID) %>%  # Group by paper (Article_ID)
  summarize(
    avg_control = mean(n_control, na.rm = TRUE),  # Average sample size for controls
    avg_experimental = mean(n_ex, na.rm = TRUE)  # Average sample size for experimental groups
  ) %>%
  mutate(
    # Replace underscores with spaces in Article_ID
    # Replace underscores with spaces in Article_ID
  # and fix some labels  ----
    Article_ID = str_replace_all(Article_ID, "_", " <i>et al.</i>, "),

  # ---- Simple + robust: replace the surname wherever it appears ----
  Article_ID = str_replace(Article_ID, "Hardardottir", "Harðardóttir"),
  Article_ID = str_replace(Article_ID, "VanDeWaal", "Van de Waal"),
  Article_ID = str_replace(Article_ID, "SenftBatoh", "Senft-Batoh"),
  
    # Calculate the mean of control and experimental sample sizes
    mean_avg_sample_size = (avg_control + avg_experimental) / 2,
    # Calculate the total height of the bar
    total_avg_sample_size = avg_control + avg_experimental
  ) %>%
  arrange(mean_avg_sample_size) %>%  # Order by mean average sample size (ascending)
  mutate(
    Article_ID = factor(Article_ID, levels = Article_ID)  # Order Article_ID as a factor
  ) %>%
  pivot_longer(cols = c(avg_control, avg_experimental),  # Reshape for stacked bar chart
               names_to = "Group",
               values_to = "Average_Sample_Size") %>%
  ggplot(aes(x = Article_ID, y = Average_Sample_Size, fill = Group)) +  # Stacked bar chart
  geom_bar(stat = "identity", width = 0.7) +  # Stacked bar
  geom_text(data = . %>% distinct(Article_ID, total_avg_sample_size, mean_avg_sample_size),  # Use distinct data for labels
            aes(x = Article_ID, y = total_avg_sample_size,  # Position labels at bar tops
                label = round(mean_avg_sample_size, 1)),
            inherit.aes = FALSE, hjust = -0.3, size = 4, color = "black") +  # Adjust label position
  scale_fill_manual(values = c("avg_control" = "grey40", "avg_experimental" = "grey80"),
                    labels = c("Control", "Treatment")) +  # Custom fill colors and legend labels
  scale_y_continuous(expand = c(0, 0),
                     limits = c(0, 40),
                     breaks = c(0, 5, 10, 15, 20, 25, 30, 35, 40)) + 

  labs(
       title = "Sample size",
       x = "",
       y = "Sample size",
       fill = "") +  # Legend title
  coord_flip() +  # Flip coordinates for horizontal bars
  theme_classic() +
  theme(text = element_text(size = 12.5, colour = "black"),
        axis.text = element_markdown(size = 12, colour = "black"),
        axis.title = element_markdown(size = 12, colour = "black"),        
        title = element_text(size = 11, colour = "black"),
        plot.margin = unit(c(0.2, 0.7, 0.2, 0.2), "cm"),        
        legend.text = element_text(size = 10,
                                   colour = "black"),
        legend.key.size = unit(12, 'pt'), # Size of legend keys
        legend.position = c(0.8, 0.2))
```

```
# ggsave(path = "figures_supplementary", "7_Supp_desc_SampleSize.png", width = 110, height = 170, units = "mm", dpi=700)
```

#### 9.7.2 Ordered by year

```
data_MA_full %>%
  filter(!is.na(Article_ID)) %>%  # Exclude rows without Article_ID
  group_by(Article_ID) %>%  # Group by paper (Article_ID)
  summarize(
    Year = first(Year),  # Assuming each Article_ID has a unique Year
    avg_control = mean(n_control, na.rm = TRUE),  # Average sample size for controls
    avg_experimental = mean(n_ex, na.rm = TRUE)  # Average sample size for experimental groups
  ) %>%
  mutate(
    # Replace underscores with spaces in Article_ID
    Article_ID = str_replace_all(Article_ID, "_", " "),
    # Calculate the mean of control and experimental sample sizes
    mean_avg_sample_size = (avg_control + avg_experimental) / 2,
    # Calculate the total height of the bar
    total_avg_sample_size = avg_control + avg_experimental
  ) %>%
  arrange(Year) %>%  # Sort by publication year (ascending)
  mutate(
    Article_ID = factor(Article_ID, levels = Article_ID)  # Order Article_ID as a factor
  ) %>%
  pivot_longer(cols = c(avg_control, avg_experimental),  # Reshape for stacked bar chart
               names_to = "Group",
               values_to = "Average_Sample_Size") %>%
  ggplot(aes(x = Article_ID, y = Average_Sample_Size, fill = Group)) +  # Stacked bar chart
  geom_bar(stat = "identity", width = 0.7) +  # Stacked bar
  geom_text(data = . %>% distinct(Article_ID, total_avg_sample_size, mean_avg_sample_size),  # Use distinct data for labels
            aes(x = Article_ID, y = total_avg_sample_size,  # Position labels at bar tops
                label = round(mean_avg_sample_size, 1)),
            inherit.aes = FALSE, hjust = -0.3, size = 4, color = "black") +  # Adjust label position
  scale_fill_manual(values = c("avg_control" = "grey40", "avg_experimental" = "grey80"),
                    labels = c("Control", "Treatment")) +  # Custom fill colors and legend labels
  scale_y_continuous(expand = c(0, 0),
                     limits = c(0, 35),
                     breaks = c(0, 5, 10, 15, 20, 25, 30, 35)) + 
  labs(
       title = "Sample size",
       x = "",
       y = "Average Sample Size",
       fill = "") +  # Legend title
  coord_flip() +  # Flip coordinates for horizontal bars
  theme_classic() +
  theme(text = element_text(size = 12.5, colour = "black"),
        axis.text = element_text(size = 12, colour = "black"),
        axis.title = element_text(size = 13, colour = "black"),
        plot.margin = unit(c(0.2, 0.7, 0.2, -0.5), "cm"),
        legend.text = element_text(size = 10,
                                   colour = "black"),
        legend.key.size = unit(12, 'pt'), # Size of legend keys
        legend.position = c(0.8, 0.2))
```

### 9.8 Culture type

```
### Calculate the grand total for the percentages
grand_total_cult <- data_MA_full %>%
  filter(!is.na(Culture_type)) %>%
  count(Culture_type, Phytoplankton_group) %>%
  pull(n) %>%
  sum()

data_MA_full %>%
  filter(!is.na(Culture_type)) %>%  # Exclude rows with missing Culture_type
  count(Culture_type, Phytoplankton_group) %>%  # Count occurrences for each Culture_type and Phytoplankton_group
  complete(Culture_type, Phytoplankton_group, fill = list(n = 0)) %>%  # Add missing combinations with 0 counts
  group_by(Culture_type) %>%
  mutate(
    # Calculate total count for each Culture_type
    culture_total = sum(n),
    # Calculate percentage of total count for each Culture_type
    culture_percentage = culture_total / grand_total_cult * 100
  ) %>%
  ungroup() %>%  # Remove grouping to modify Culture_type levels
  mutate(
    # Order Culture_type by total count (descending)
    Culture_type = factor(Culture_type, levels = unique(Culture_type[order(culture_total)]))
  ) %>%
  ggplot(aes(x = Culture_type, y = n, fill = Phytoplankton_group)) +  # Stacked bar chart
  geom_bar(stat = "identity", width = 0.7) +  # Use `identity` for precomputed counts
  geom_text(data = . %>% distinct(Culture_type, culture_total, culture_percentage),  # Use distinct data for labels
            aes(x = Culture_type, y = culture_total, 
                label = paste0(round(culture_percentage, 0), "%")),
            inherit.aes = FALSE, hjust = -0.3, size = 4, color = "black") +  # Add percentages next to bars
  scale_y_continuous(expand = c(0, 0),
                     limits = c(0, 130),
                     n.breaks = 8) +  # Dynamic y-axis limit
  scale_fill_manual(values = c("Alexandrium" = "grey40", "Pseudo-nitzschia" = "grey80"))+
  labs(
       title = paste(
         "Culture type: *k* =", 
         grand_total_cult  # Display grand total in the title
       ),
       x = "",
       y = "Effect size count (*k*)",
       fill = "") +  # Legend title
  coord_flip() +  # Flip coordinates for horizontal bars
  theme_classic() +
  theme(text = element_text(size = 12.5, colour = "black"),
        axis.text = element_text(size = 12, colour = "black"),
        axis.title = element_markdown(size = 12, colour = "black"),   
        plot.title = element_markdown(),
        title = element_text(size = 10, colour = "black"),
        plot.margin = unit(c(0.2, 0.7, 0.2, 0.2), "cm"),
        legend.position = c(0.7, 0.4),
        legend.text = element_text(size = 10,
                                   colour = "black",
                                   face = "italic"),
        legend.box.background = element_rect(
          fill = 'transparent', # Transparent legend panel
          colour = 'transparent' # Transparent border
        ),
        legend.background = element_rect(
          fill = 'transparent', # Transparent background
          colour = 'transparent' # Transparent border
        ),
        legend.key.size = unit(12, 'pt') # Size of legend keys
  )
```

```
# ggsave(path = "figures_supplementary", "8_Supp_desc_Culturing.png", width = 100, height = 40, units = "mm", dpi=700)
```

### 9.9 Panel plot hist. cont. moderators

#### 9.9.1 Illumination

```
# Calculate the grand total for the percentages
grand_total_LR <- data_MA_full %>%
  filter(!is.na(Light)) %>%
  count(Light) %>%
  pull(n) %>%
  sum()

SI_9_A <-
data_MA_full %>% 
  ggplot(aes(x = Light, fill = Exp_type)) + 
  geom_histogram(binwidth = 25, color = "black", size = 0.3, position = "stack") +  # Adjust bin width and style
  scale_fill_manual(values = c("grey40", "grey80")) +  # Apply color scheme
  scale_y_continuous(expand = c(0, 0),
                     limits = c(0, 35),  # Dynamic y-axis limits
                     breaks = seq(0, 35, by = 5)) +  # Adjust breaks dynamically
  scale_x_continuous(expand = c(0, 0),
                     #limits = c(0, 400),  # Dynamic y-axis limits
                     breaks = seq(0, 400, by = 25))+
  labs(
       title = paste(
         "*k* =", 
         grand_total_LR  # Display grand total in the title
       ),
       x = bquote(Illumination~(µmol~photons~m^-2~s^-1)),
       y = "Count") +
  theme_classic() +
  theme(text = element_text(size = 12.5, colour = "black"),
        axis.text = element_text(size = 10, colour = "black"),
        axis.title = element_text(size = 12, colour = "black"), 
        plot.title = element_markdown(size = 11, colour = "black"),    
    plot.margin = unit(c(0, 0.7, 0.1, 0.5), "cm"),
    legend.position = c(0.9,0.8),  # Adjust legend position
    legend.title = element_blank(),  # Remove legend title
    legend.key.size = unit(12, 'pt'), # Size of legend keys
    legend.text = element_text(size = 10)  # Adjust legend text size
  )

# ggsave(path = "figures_supplementary", "Supp_desc_Light_illum.png", width = 140, height = 50, units = "mm", dpi=700)
```

#### 9.9.2 Light:dark cycle

```
# Calculate the grand total for the percentages
grand_total_LD <- data_MA_full %>%
  filter(!is.na(LD_cycle)) %>%
  count(LD_cycle) %>%
  pull(n) %>%
  sum()


SI_9_B <-
data_MA_full %>% 
  ggplot(aes(x = LD_cycle, fill = Exp_type)) + 
  geom_histogram(binwidth = 1, color = "black", size = 0.3, position = "stack") +  # Adjust bin width and style
  scale_fill_manual(values = c("grey40", "grey80")) +  # Apply color scheme
  scale_y_continuous(expand = c(0, 0),
                     limits = c(0, 60),  # Dynamic y-axis limits
                     breaks = seq(0, 60, by = 10)) +  # Adjust breaks dynamically
  scale_x_continuous(expand = c(0, 0),
                    # limits = c(10, 24),  # Dynamic y-axis limits
                     breaks = seq(12, 22, by = 1))+
  labs(
       title = paste(
         "*k* =", 
         grand_total_LD  # Display grand total in the title
       ),
       x = "Light hours (h)",
       y = "Count") +
  theme_classic() +
  theme(text = element_text(size = 12.5, colour = "black"),
        axis.text = element_text(size = 10, colour = "black"),
        axis.title = element_text(size = 12, colour = "black"),        
        plot.title = element_markdown(size = 11, colour = "black"),    
    plot.margin = unit(c(0, 0.7, 0.1, 0.5), "cm"),
    legend.position = c(0.9,0.8),  # Adjust legend position
    legend.title = element_blank(),  # Remove legend title
    legend.key.size = unit(12, 'pt'), # Size of legend keys
    legend.text = element_text(size = 10)  # Adjust legend text size
  )

# ggsave(path = "figures_supplementary", "Supp_desc_Light_Dark.png", width = 140, height = 50, units = "mm", dpi=700)
```

#### 9.9.3 Temperature

```
# Calculate the grand total for the percentages
grand_total_temp <- data_MA_full %>%
  filter(!is.na(Temperature)) %>%
  count(Temperature) %>%
  pull(n) %>%
  sum()

SI_9_C <-
data_MA_full %>% 
  ggplot(aes(x = Temperature, fill = Exp_type)) + 
  geom_histogram(binwidth = 2, color = "black", size = 0.3, position = "stack") +  # Adjust bin width and style
  scale_fill_manual(values = c("grey40", "grey80")) +  # Apply color scheme
  scale_y_continuous(expand = c(0, 0),
                     limits = c(0, 50),  # Dynamic y-axis limits
                     breaks = seq(0, 60, by = 10)) +  # Adjust breaks dynamically
  scale_x_continuous(expand = c(0, 0),
                    # limits = c(10, 24),  # Dynamic y-axis limits
                     breaks = seq(0, 30, by = 2))+
  labs(
       title = paste(
         "*k* =", 
         grand_total_temp  # Display grand total in the title
       ),
       x = "Temperature (°C)",
       y = "Count") +
  theme_classic() +
  theme(text = element_text(size = 12.5, colour = "black"),
        axis.text = element_text(size = 10, colour = "black"),
        axis.title = element_text(size = 12, colour = "black"),        
        plot.title = element_markdown(size = 11, colour = "black"),    
    plot.margin = unit(c(0, 0.7, 0.1, 0.5), "cm"),
    legend.position = c(0.9,0.8),  # Adjust legend position
    legend.title = element_blank(),  # Remove legend title
    legend.key.size = unit(12, 'pt'), # Size of legend keys
    legend.text = element_text(size = 10)  # Adjust legend text size
  )

# ggsave(path = "figures_supplementary", "Supp_desc_Temp.png", width = 140, height = 50, units = "mm", dpi=700)
```

#### 9.9.4 Salinity

```
# Calculate the grand total for the percentages
grand_total_sal <- data_MA_full %>%
  filter(!is.na(Salinity)) %>%
  count(Salinity) %>%
  pull(n) %>%
  sum()

SI_9_D <-
data_MA_full %>% 
  ggplot(aes(x = Salinity, fill = Exp_type)) + 
  geom_histogram(binwidth = 2, color = "black", size = 0.3, position = "stack") +  # Adjust bin width and style
  scale_fill_manual(values = c("grey40", "grey80")) +  # Apply color scheme
  scale_y_continuous(expand = c(0, 0),
                     limits = c(0, 40),  # Dynamic y-axis limits
                     breaks = seq(0, 40, by = 10)) +  # Adjust breaks dynamically
  scale_x_continuous(expand = c(0, 0),
                    # limits = c(10, 24),  # Dynamic y-axis limits
                     breaks = seq(16, 36, by = 2))+
  labs(
       title = paste(
         "*k* =", 
         grand_total_sal  # Display grand total in the title
       ),
       x = "Salinity (PSU)",
       y = "Count") +
  theme_classic() +
  theme(text = element_text(size = 12.5, colour = "black"),
        axis.text = element_text(size = 10, colour = "black"),
        axis.title = element_text(size = 12, colour = "black"),        
        plot.title = element_markdown(size = 11, colour = "black"),    
    plot.margin = unit(c(0, 0.7, 0.1, 0), "cm"),
    legend.position = c(0.2,0.6),  # Adjust legend position
    legend.title = element_blank(),  # Remove legend title
    legend.key.size = unit(12, 'pt'), # Size of legend keys
    legend.text = element_text(size = 10)  # Adjust legend text size
  )

# ggsave(path = "figures_supplementary", "Supp_desc_Salinity.png", width = 140, height = 50, units = "mm", dpi=700)
```

#### 9.9.5 Experimental duration

```
# Calculate the grand total for the percentages
grand_total_hours <- data_MA_full %>%
  filter(!is.na(Hours)) %>%
  count(Hours) %>%
  pull(n) %>%
  sum()

SI_9_E <-
data_MA_full %>% 
  ggplot(aes(x = Hours, fill = Exp_type)) + 
  geom_histogram(binwidth = 60, color = "black", size = 0.3, position = "stack") +  # Adjust bin width and style
  scale_fill_manual(values = c("grey40", "grey80")) +  # Apply color scheme
  scale_y_continuous(expand = c(0, 0),
                     limits = c(0, 50),  # Dynamic y-axis limits
                     breaks = seq(0, 50, by = 10)) +  # Adjust breaks dynamically
  scale_x_continuous(expand = c(0, 0),
                     limits = c(0, 540),  # Dynamic y-axis limits
                     breaks = seq(0, 540, by = 60))+
  labs(
       title = paste(
         "*k* =", 
         grand_total_hours  # Display grand total in the title
       ),
       caption = "N.B: Two effect sizes have value 5760 h, but are omitted here.",
       x = "Experiment duration (h)",
       y = "Count") +
  theme_classic() +
  #coord_flip()+
  theme(text = element_text(size = 12.5, colour = "black"),
        axis.text = element_text(size = 10, colour = "black"),
        axis.title = element_text(size = 12, colour = "black"),        
        plot.title = element_markdown(size = 11, colour = "black"),    
    plot.margin = unit(c(0, 0.7, 0, 0.5), "cm"),
    legend.position = c(0.9,0.8),  # Adjust legend position
    legend.title = element_blank(),  # Remove legend title
    legend.key.size = unit(12, 'pt'), # Size of legend keys
    legend.text = element_text(size = 10)  # Adjust legend text size
  )

# ggsave(path = "figures_supplementary", "Supp_desc_Duration.png", width = 140, height = 50, units = "mm", dpi=700)
```

#### 9.9.6 Putting them together

```
(
  SI_9_A /
  SI_9_B /
  SI_9_C /
  SI_9_D /
  SI_9_E
) +
  plot_layout(heights = c(1, 1, 1, 1, 1)) +
  plot_annotation(
    tag_levels = "A",
    tag_prefix = "",
    tag_suffix = ""
  ) +
  ggplot2::theme(
    plot.tag.position = c(0, 1),                 # top-left corner anchor
    plot.tag = ggplot2::element_text(
      vjust = 1                                  # move tag DOWN (tune this)
    )
  )
```

```
# ggsave(path = "figures_supplementary", "9_Supp_desc_panel.png", width = 150, height = 250, units = "mm", dpi=1500)
```

#### 9.15 Panel bubble plot illum. and temp

```
  (SI_15A + coord_cartesian(ylim = c(-3,6))+ theme(legend.position = "inside")) /
  (SI_15B + coord_cartesian(ylim = c(-3,6))+ theme(legend.position = "inside")) +
  
  plot_layout(heights = c(1, 1))+
  plot_annotation(
    tag_levels = "A",
    tag_prefix = "",
    tag_suffix = "")+
  
  theme(plot.tag.position = c(0, 1), 
        plot.tag = element_text(vjust = 1.5),
        plot.margin = margin(2, 3, 2, 3, unit = "mm"))
```

```
# ggsave(path = "figures_supplementary", "15_Supp_desc_panel_bubble.png", width = 180, height = 200, units = "mm", dpi=1500)
```

## 10. R session information

```
devtools::session_info()
```

```
## ─ Session info ───────────────────────────────────────────────────────────────
##  setting  value
##  version  R version 4.4.1 (2024-06-14 ucrt)
##  os       Windows 11 x64 (build 22631)
##  system   x86_64, mingw32
##  ui       RTerm
##  language (EN)
##  collate  Swedish_Sweden.utf8
##  ctype    Swedish_Sweden.utf8
##  tz       Europe/Stockholm
##  date     2026-01-12
##  pandoc   3.1.11 @ C:/Program Files/RStudio/resources/app/bin/quarto/bin/tools/ (via rmarkdown)
## 
## ─ Packages ───────────────────────────────────────────────────────────────────
##  ! package      * version    date (UTC) lib source
##    backports      1.5.0      2024-05-23 [1] CRAN (R 4.4.0)
##    beeswarm       0.4.0      2021-06-01 [1] CRAN (R 4.4.0)
##    broom          1.0.6      2024-05-17 [1] CRAN (R 4.4.1)
##    bslib          0.7.0      2024-03-29 [1] CRAN (R 4.4.0)
##    cachem         1.1.0      2024-05-16 [1] CRAN (R 4.4.0)
##    cli            3.6.1      2023-03-23 [1] CRAN (R 4.2.3)
##    coda           0.19-4.1   2024-01-31 [1] CRAN (R 4.4.0)
##    codetools      0.2-20     2024-03-31 [1] CRAN (R 4.4.0)
##  D colorspace     2.1-0      2023-01-23 [1] CRAN (R 4.3.2)
##    commonmark     1.9.1      2024-01-30 [1] CRAN (R 4.4.0)
##    devtools     * 2.4.5      2022-10-11 [1] CRAN (R 4.4.1)
##    digest         0.6.36     2024-06-23 [1] CRAN (R 4.4.1)
##    dplyr        * 1.1.4      2023-11-17 [1] CRAN (R 4.4.0)
##  D ellipsis       0.3.2      2021-04-29 [1] CRAN (R 4.4.1)
##    emmeans      * 1.10.3     2024-07-01 [1] CRAN (R 4.4.1)
##    estimability   1.5.1      2024-05-12 [1] CRAN (R 4.4.0)
##    evaluate       0.24.0     2024-06-10 [1] CRAN (R 4.4.0)
##    fansi          1.0.6      2023-12-08 [1] CRAN (R 4.4.0)
##    farver         2.1.2      2024-05-13 [1] CRAN (R 4.4.0)
##    fastmap        1.2.0      2024-05-15 [1] CRAN (R 4.4.0)
##    forcats      * 1.0.0      2023-01-29 [1] CRAN (R 4.4.0)
##    fs             1.6.4      2024-04-25 [1] CRAN (R 4.4.0)
##    generics       0.1.3      2022-07-05 [1] CRAN (R 4.4.0)
##    ggbeeswarm     0.7.2      2023-04-29 [1] CRAN (R 4.4.0)
##    ggforestplot * 0.1.0      2023-03-29 [1] Github (NightingaleHealth/ggforestplot@547617e)
##    ggplot2      * 3.5.1      2024-04-23 [1] CRAN (R 4.4.0)
##    ggridges     * 0.5.6      2024-01-23 [1] CRAN (R 4.4.0)
##    ggtext       * 0.1.2      2022-09-16 [1] CRAN (R 4.4.0)
##    glmulti      * 1.0.8      2020-05-26 [1] CRAN (R 4.4.0)
##    glue           1.6.2      2022-02-24 [1] CRAN (R 4.1.3)
##    gridtext       0.1.5      2022-09-16 [1] CRAN (R 4.4.0)
##    gtable         0.3.5      2024-04-22 [1] CRAN (R 4.4.0)
##    highr          0.11       2024-05-26 [1] CRAN (R 4.4.0)
##    hms            1.1.3      2023-03-21 [1] CRAN (R 4.4.1)
##    htmltools      0.5.8.1    2024-04-04 [1] CRAN (R 4.4.0)
##    htmlwidgets    1.6.4      2023-12-06 [1] CRAN (R 4.4.1)
##    httpuv         1.6.15     2024-03-26 [1] CRAN (R 4.4.0)
##    jquerylib      0.1.4      2021-04-26 [1] CRAN (R 4.4.1)
##    jsonlite       1.8.8      2023-12-04 [1] CRAN (R 4.4.1)
##    knitr          1.48       2024-07-07 [1] CRAN (R 4.4.1)
##    labeling       0.4.3      2023-08-29 [1] CRAN (R 4.4.0)
##    later          1.3.2      2023-12-06 [1] CRAN (R 4.3.2)
##    latex2exp      0.9.6      2022-11-28 [1] CRAN (R 4.4.1)
##    lattice        0.22-6     2024-03-20 [2] CRAN (R 4.4.1)
##  D lazyeval       0.2.2      2019-03-15 [1] CRAN (R 4.4.1)
##    leaps        * 3.2        2024-06-10 [1] CRAN (R 4.4.0)
##    lifecycle      1.0.4      2023-11-07 [1] CRAN (R 4.4.1)
##    lubridate    * 1.9.3      2023-09-27 [1] CRAN (R 4.4.1)
##    magrittr       2.0.3      2022-03-30 [1] CRAN (R 4.1.3)
##    markdown       1.13       2024-06-04 [1] CRAN (R 4.4.0)
##    MASS         * 7.3-60     2023-05-04 [1] CRAN (R 4.3.2)
##    mathjaxr       1.6-0      2022-02-28 [1] CRAN (R 4.4.1)
##    Matrix       * 1.7-0      2024-03-22 [1] CRAN (R 4.4.1)
##    memoise        2.0.1      2021-11-26 [1] CRAN (R 4.4.1)
##    metadat      * 1.4-0      2025-02-04 [1] CRAN (R 4.4.2)
##    metafor      * 4.6-0      2024-03-28 [1] CRAN (R 4.4.2)
##    mgcv           1.9-1      2023-12-21 [1] CRAN (R 4.4.0)
##  D mime           0.12       2021-09-28 [1] CRAN (R 4.3.1)
##    miniUI         0.1.1.1    2018-05-18 [1] CRAN (R 4.4.1)
##    multcomp     * 1.4-25     2023-06-20 [1] CRAN (R 4.4.1)
##    munsell        0.5.1      2024-04-01 [1] CRAN (R 4.4.0)
##  D mvtnorm      * 1.2-5      2024-05-21 [1] CRAN (R 4.4.1)
##    nlme           3.1-165    2024-06-06 [1] CRAN (R 4.4.1)
##    numDeriv     * 2016.8-1.1 2019-06-06 [1] CRAN (R 4.4.0)
##    orchaRd      * 2.0        2022-08-17 [1] Github (daniel1noble/orchaRd@a7de0d5)
##    pacman       * 0.5.1      2019-03-11 [1] CRAN (R 4.4.1)
##    patchwork    * 1.2.0      2024-01-08 [1] CRAN (R 4.4.0)
##    pillar         1.9.0      2023-03-22 [1] CRAN (R 4.3.2)
##    pkgbuild       1.4.4      2024-03-17 [1] CRAN (R 4.4.0)
##    pkgconfig      2.0.3      2019-09-22 [1] CRAN (R 4.3.2)
##    pkgload        1.4.0      2024-06-28 [1] CRAN (R 4.4.1)
##    profvis        0.3.8      2023-05-02 [1] CRAN (R 4.3.2)
##    promises       1.3.0      2024-04-05 [1] CRAN (R 4.4.0)
##    purrr        * 1.0.2      2023-08-10 [1] CRAN (R 4.4.0)
##    R6             2.5.1      2021-08-19 [1] CRAN (R 4.3.2)
##    Rcpp           1.0.12     2024-01-09 [1] CRAN (R 4.4.0)
##    readr        * 2.1.5      2024-01-10 [1] CRAN (R 4.4.1)
##    remotes        2.5.0      2024-03-17 [1] CRAN (R 4.4.0)
##  D rJava        * 1.0-11     2024-01-26 [1] CRAN (R 4.4.0)
##    rlang          1.1.4      2024-06-04 [1] CRAN (R 4.4.1)
##    rmarkdown      2.27       2024-05-17 [1] CRAN (R 4.4.0)
##    rstudioapi     0.16.0     2024-03-24 [1] CRAN (R 4.4.0)
##    sandwich       3.1-0      2023-12-11 [1] CRAN (R 4.3.2)
##    sass           0.4.9      2024-03-15 [1] CRAN (R 4.4.0)
##    scales         1.3.0      2023-11-28 [1] CRAN (R 4.3.2)
##    sessioninfo    1.2.2      2021-12-06 [1] CRAN (R 4.3.2)
##    shiny          1.8.1.1    2024-04-02 [1] CRAN (R 4.4.0)
##    stringi        1.7.12     2023-01-11 [1] CRAN (R 4.2.2)
##    stringr      * 1.5.1      2023-11-14 [1] CRAN (R 4.3.2)
##    survival     * 3.7-0      2024-06-05 [1] CRAN (R 4.4.1)
##    TH.data      * 1.1-2      2023-04-17 [1] CRAN (R 4.3.2)
##    tibble       * 3.2.1      2023-03-20 [1] CRAN (R 4.2.3)
##    tidyr        * 1.3.1      2024-01-24 [1] CRAN (R 4.4.0)
##    tidyselect     1.2.1      2024-03-11 [1] CRAN (R 4.4.0)
##    tidyverse    * 2.0.0      2023-02-22 [1] CRAN (R 4.4.1)
##    timechange     0.3.0      2024-01-18 [1] CRAN (R 4.4.0)
##  D tzdb           0.4.0      2023-05-12 [1] CRAN (R 4.3.2)
##    urlchecker     1.0.1      2021-11-30 [1] CRAN (R 4.3.2)
##    usethis      * 2.2.3      2024-02-19 [1] CRAN (R 4.4.0)
##    utf8           1.2.4      2023-10-22 [1] CRAN (R 4.4.0)
##    vctrs          0.6.4      2023-10-12 [1] CRAN (R 4.2.3)
##    vipor          0.4.7      2023-12-18 [1] CRAN (R 4.3.2)
##    withr          3.0.0      2024-01-16 [1] CRAN (R 4.4.0)
##    xfun           0.45       2024-06-16 [1] CRAN (R 4.4.1)
##    xml2           1.3.6      2023-12-04 [1] CRAN (R 4.4.0)
##    xtable         1.8-4      2019-04-21 [1] CRAN (R 4.3.2)
##  D yaml           2.3.9      2024-07-05 [1] CRAN (R 4.4.1)
##    zoo            1.8-12     2023-04-13 [1] CRAN (R 4.3.2)
## 
##  [1] C:/Users/xpoumi/OneDrive/Dokument/R/win-library/4.0
##  [2] C:/Program Files/R/R-4.4.1/library
## 
##  D ── DLL MD5 mismatch, broken installation.
## 
## ──────────────────────────────────────────────────────────────────────────────
```

## 11. Package references

```
citation("orchaRd")
```

```
## To cite orchaRd in publications use:
## 
## 
## A BibTeX entry for LaTeX users is
## 
##   @Article{,
##     title = {The orchard plot: Cultivating a forest plot for use in ecology, evolution, and beyond},
##     author = {Shinichi Nakagawa and Malgorzata Lagisz and Rose E O'Dea and Joanna Rutkowska and Yefeng Yang and Daniel WA Noble and Alistair M Senior},
##     journal = {Research Synthesis Methods},
##     year = {2021},
##     volume = {12},
##     pages = {4-12},
##     doi = {DOI: 10.1002/jrsm.1424},
##   }
```

```
citation("ggforestplot")
```

```
## To cite package 'ggforestplot' in publications use:
## 
##   Scheinin I, Kalimeri M, Jagerroos V, Parkkinen J, Tikkanen E, Würtz
##   P, Kangas A (2023). _ggforestplot: Forestplots of Measures of Effects
##   and Their Confidence Intervals_. R package version 0.1.0, commit
##   547617e63fa481a5f28ffc56c07d46be4af688b2,
##   <https://github.com/NightingaleHealth/ggforestplot>.
## 
## A BibTeX entry for LaTeX users is
## 
##   @Manual{,
##     title = {ggforestplot: Forestplots of Measures of Effects and Their Confidence
## Intervals},
##     author = {Ilari Scheinin and Maria Kalimeri and Vilma Jagerroos and Juuso Parkkinen and Emmi Tikkanen and Peter Würtz and Antti Kangas},
##     year = {2023},
##     note = {R package version 0.1.0, commit 547617e63fa481a5f28ffc56c07d46be4af688b2},
##     url = {https://github.com/NightingaleHealth/ggforestplot},
##   }
```

```
citation("tidyverse")
```

```
## To cite package 'tidyverse' in publications use:
## 
##   Wickham H, Averick M, Bryan J, Chang W, McGowan LD, François R,
##   Grolemund G, Hayes A, Henry L, Hester J, Kuhn M, Pedersen TL, Miller
##   E, Bache SM, Müller K, Ooms J, Robinson D, Seidel DP, Spinu V,
##   Takahashi K, Vaughan D, Wilke C, Woo K, Yutani H (2019). "Welcome to
##   the tidyverse." _Journal of Open Source Software_, *4*(43), 1686.
##   doi:10.21105/joss.01686 <https://doi.org/10.21105/joss.01686>.
## 
## A BibTeX entry for LaTeX users is
## 
##   @Article{,
##     title = {Welcome to the {tidyverse}},
##     author = {Hadley Wickham and Mara Averick and Jennifer Bryan and Winston Chang and Lucy D'Agostino McGowan and Romain François and Garrett Grolemund and Alex Hayes and Lionel Henry and Jim Hester and Max Kuhn and Thomas Lin Pedersen and Evan Miller and Stephan Milton Bache and Kirill Müller and Jeroen Ooms and David Robinson and Dana Paige Seidel and Vitalie Spinu and Kohske Takahashi and Davis Vaughan and Claus Wilke and Kara Woo and Hiroaki Yutani},
##     year = {2019},
##     journal = {Journal of Open Source Software},
##     volume = {4},
##     number = {43},
##     pages = {1686},
##     doi = {10.21105/joss.01686},
##   }
```

```
citation("readr")
```

```
## To cite package 'readr' in publications use:
## 
##   Wickham H, Hester J, Bryan J (2024). _readr: Read Rectangular Text
##   Data_. R package version 2.1.5,
##   <https://CRAN.R-project.org/package=readr>.
## 
## A BibTeX entry for LaTeX users is
## 
##   @Manual{,
##     title = {readr: Read Rectangular Text Data},
##     author = {Hadley Wickham and Jim Hester and Jennifer Bryan},
##     year = {2024},
##     note = {R package version 2.1.5},
##     url = {https://CRAN.R-project.org/package=readr},
##   }
```

```
citation("devtools")
```

```
## To cite package 'devtools' in publications use:
## 
##   Wickham H, Hester J, Chang W, Bryan J (2022). _devtools: Tools to
##   Make Developing R Packages Easier_. R package version 2.4.5,
##   <https://CRAN.R-project.org/package=devtools>.
## 
## A BibTeX entry for LaTeX users is
## 
##   @Manual{,
##     title = {devtools: Tools to Make Developing R Packages Easier},
##     author = {Hadley Wickham and Jim Hester and Winston Chang and Jennifer Bryan},
##     year = {2022},
##     note = {R package version 2.4.5},
##     url = {https://CRAN.R-project.org/package=devtools},
##   }
```

```
citation("glmulti")
```

```
## To cite package 'glmulti' in publications use:
## 
##   Calcagno V (2020). _glmulti: Model Selection and Multimodel Inference
##   Made Easy_. R package version 1.0.8,
##   <https://CRAN.R-project.org/package=glmulti>.
## 
## A BibTeX entry for LaTeX users is
## 
##   @Manual{,
##     title = {glmulti: Model Selection and Multimodel Inference Made Easy},
##     author = {Vincent Calcagno},
##     year = {2020},
##     note = {R package version 1.0.8},
##     url = {https://CRAN.R-project.org/package=glmulti},
##   }
```

```
citation("patchwork")
```

```
## To cite package 'patchwork' in publications use:
## 
##   Pedersen T (2024). _patchwork: The Composer of Plots_. R package
##   version 1.2.0, <https://CRAN.R-project.org/package=patchwork>.
## 
## A BibTeX entry for LaTeX users is
## 
##   @Manual{,
##     title = {patchwork: The Composer of Plots},
##     author = {Thomas Lin Pedersen},
##     year = {2024},
##     note = {R package version 1.2.0},
##     url = {https://CRAN.R-project.org/package=patchwork},
##   }
```

```
citation("multcomp")
```

```
## Please cite the multcomp package by the following reference:
## 
##   Hothorn T, Bretz F, Westfall P (2008). "Simultaneous Inference in
##   General Parametric Models." _Biometrical Journal_, *50*(3), 346-363.
## 
## A BibTeX entry for LaTeX users is
## 
##   @Article{,
##     title = {Simultaneous Inference in General Parametric Models},
##     author = {Torsten Hothorn and Frank Bretz and Peter Westfall},
##     journal = {Biometrical Journal},
##     year = {2008},
##     volume = {50},
##     number = {3},
##     pages = {346--363},
##   }
```

```
citation("emmeans")
```

```
## To cite package 'emmeans' in publications use:
## 
##   Lenth R (2024). _emmeans: Estimated Marginal Means, aka Least-Squares
##   Means_. R package version 1.10.3,
##   <https://CRAN.R-project.org/package=emmeans>.
## 
## A BibTeX entry for LaTeX users is
## 
##   @Manual{,
##     title = {emmeans: Estimated Marginal Means, aka Least-Squares Means},
##     author = {Russell V. Lenth},
##     year = {2024},
##     note = {R package version 1.10.3},
##     url = {https://CRAN.R-project.org/package=emmeans},
##   }
```

```
citation("metafor")
```

```
## To cite the metafor package in publications, please use:
## 
##   Viechtbauer, W. (2010). Conducting meta-analyses in R with the
##   metafor package. Journal of Statistical Software, 36(3), 1-48.
##   https://doi.org/10.18637/jss.v036.i03
## 
## A BibTeX entry for LaTeX users is
## 
##   @Article{,
##     title = {Conducting meta-analyses in {R} with the {metafor} package},
##     author = {Wolfgang Viechtbauer},
##     journal = {Journal of Statistical Software},
##     year = {2010},
##     volume = {36},
##     number = {3},
##     pages = {1--48},
##     doi = {10.18637/jss.v036.i03},
##   }
```

```
citation("ggtext")
```

```
## To cite package 'ggtext' in publications use:
## 
##   Wilke C, Wiernik B (2022). _ggtext: Improved Text Rendering Support
##   for 'ggplot2'_. R package version 0.1.2,
##   <https://CRAN.R-project.org/package=ggtext>.
## 
## A BibTeX entry for LaTeX users is
## 
##   @Manual{,
##     title = {ggtext: Improved Text Rendering Support for 'ggplot2'},
##     author = {Claus O. Wilke and Brenton M. Wiernik},
##     year = {2022},
##     note = {R package version 0.1.2},
##     url = {https://CRAN.R-project.org/package=ggtext},
##   }
```

```
citation("ggridges")
```

```
## To cite package 'ggridges' in publications use:
## 
##   Wilke C (2024). _ggridges: Ridgeline Plots in 'ggplot2'_. R package
##   version 0.5.6, <https://CRAN.R-project.org/package=ggridges>.
## 
## A BibTeX entry for LaTeX users is
## 
##   @Manual{,
##     title = {ggridges: Ridgeline Plots in 'ggplot2'},
##     author = {Claus O. Wilke},
##     year = {2024},
##     note = {R package version 0.5.6},
##     url = {https://CRAN.R-project.org/package=ggridges},
##   }
```

## References

Fernández-Castilla, B., Declercq, L., Jamshidi,
L., Beretvas, S.N., Onghena, P. & Van
den Noortgate, W. (2021) Detecting
selection bias in meta-analyses with multiple outcomes: A simulation
study. *The Journal of Experimental Education*
**89**, 125–144.

Hedges, L.V. (1981) Distribution
theory for glass’s estimator of effect size and related estimators.
*journal of Educational Statistics* **6**, 107–128.

Lajeunesse, M.J. (2015) Bias and correction for
the log response ratio in ecological meta-analysis. *Ecology*
**96**, 2056–2063.

Nakagawa, S., Lagisz, M., Jennions,
M.D., Koricheva, J., Noble, D.W., Parker,
T.H., Sánchez-Tójar, A., Yang, Y. & O’Dea,
R.E. (2022) Methods for testing
publication bias in ecological and evolutionary meta-analyses.
*Methods in Ecology and Evolution* **13**, 4–21.

Nakagawa, S., Lagisz, M., O’Dea,
R.E., Pottier, P., Rutkowska, J., Yang,
Y., Senior, A.M. & Noble, D.W.A. (2023) orchaRd 2.0: An R package for drawing ‘orchard’ plots
(and ‘caterpillars’ plots) from meta-analyses and
meta-regressions. Https://daniel1noble.github.io/orchaRd/.

Nakagawa, S., Lagisz, M., O’Dea,
R.E., Rutkowska, J., Yang, Y., Noble,
D.W. & Senior, A.M. (2021) The orchard plot:
Cultivating a forest plot for use in ecology, evolution, and beyond.
*Research Synthesis Methods* **12**, 4–12.

Rodgers, M.A. & Pustejovsky, J.E. (2021) Evaluating meta-analytic
methods to detect selective reporting in the presence of dependent
effect sizes. *Psychological methods* **26**,
141.

Rosenthal, R. (1979) The file drawer
problem and tolerance for null results. *Psychological
bulletin* **86**, 638–641.
