# Supplementary material for: Fear of grazing rivals the toxin‐inducing effects of nutrients in two marine harmful algae – a meta‐analysis
Source: Biol Rev Camb Philos Soc. 2026 Mar 8;101(4):1904–21. doi: 10.1002/brv.70153 (PMC13326770; doi:10.1002/brv.70153)
Supplement: Supplementary file 3 — Fig. S1. Distribution of effect sizes (k) distributed between the two phytoplankton genera Alexandrium and Pseudo‐nitzschia. Fig. S2. Distribution of effect sizes (k) between levels of moderator driver (demand = elevated grazing risk; resource = relative nitrogen enrichment), coloured by phytoplankton genus (Alexandrium and Pseudo‐nitzschia). Fig. S3. Distribution of effect sizes (k) among phytoplankton strains. Fig. S4. Distribution of effect sizes (k) among phytoplankton species. Fig. S5. Distribution of effect sizes (k) from studies that exposed phytoplankton to live zooplankton among zooplankton (copepod) species. Fig. S6. Distribution of effect sizes (k) over publication year, coloured by experiment type/driver (demand = elevated grazing risk; resource = resource = relative nitrogen enrichment). Fig. S7. Total sample size of studies included in the analysis, coloured by experimental group (control and treatment). Fig. S8. Distribution of effect sizes (k) between levels of moderator culture type, partitioned by phytoplankton genus (Alexandrium and Pseudo‐nitzschia). Fig. S9. Stacked histogram of effect sizes (k) distributed over continuous moderators (A) light intensity, (B) light: dark cycle, (C) temperature, (D) salinity, (E) and duration of experiment. Fig. S10. Pooled effects of relative nitrogen enrichment (resource) and elevated grazing risk (demand) on phycotoxin induction (LRRΔ), shown separately for each genus. Fig. S11. Effects of relative nitrogen enrichment (resource) or elevated grazing risk (demand) on phycotoxin induction (LRRΔ), separated by phytoplankton species within genera Alexandrium and Pseudo‐nitzschia. Fig. S12. Effects of relative nitrogen enrichment (resource) or elevated grazing risk (demand) on phycotoxin induction (LRRΔ), partitioned among phytoplankton strains. Fig. S13. Effects of relative nitrogen enrichment (resource) or elevated grazing risk (demand) on phycotoxin induction (LRRΔ), separated by culture medium used. Fig. S14. Eff [file BRV-101-1904-s003.docx]

**Supporting information for:**

**Fear of grazing rivals the toxin-inducing effects of nutrients in two marine harmful algae – a meta-analysis**


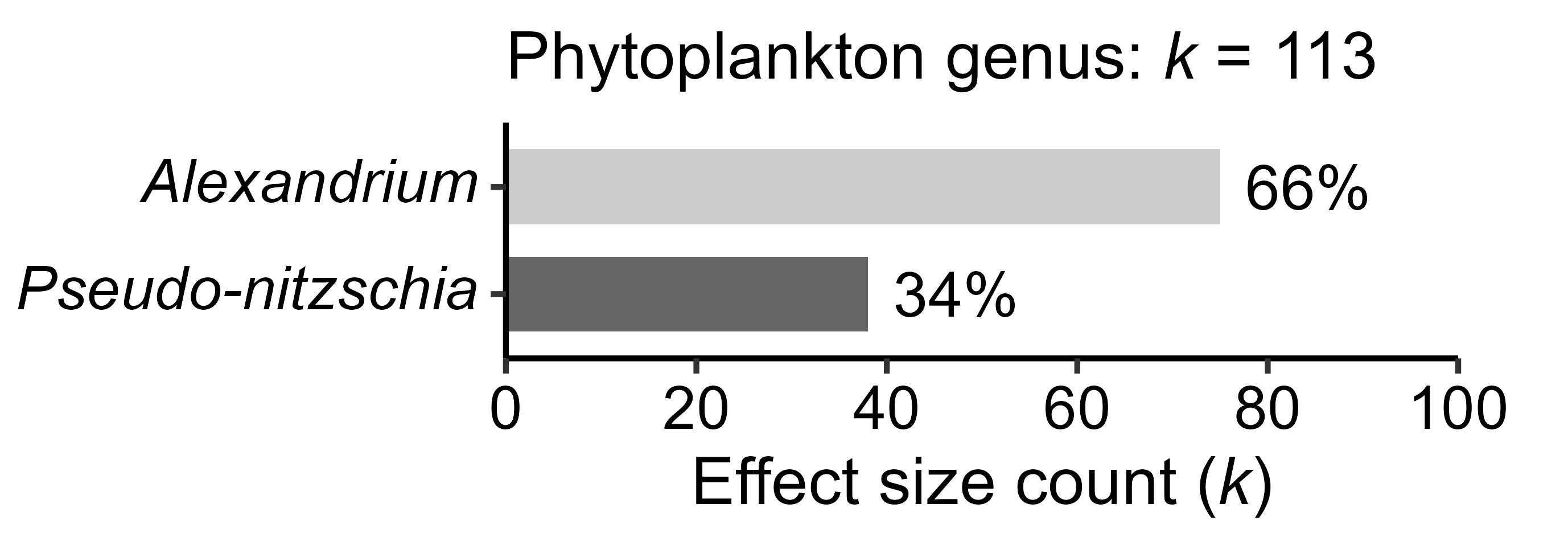


**Fig. S1.** Distribution of effect sizes (*k*) between the two phytoplankton genera, the dinoflagellate *Alexandrium* and the diatom *Pseudo-nitzschia*.


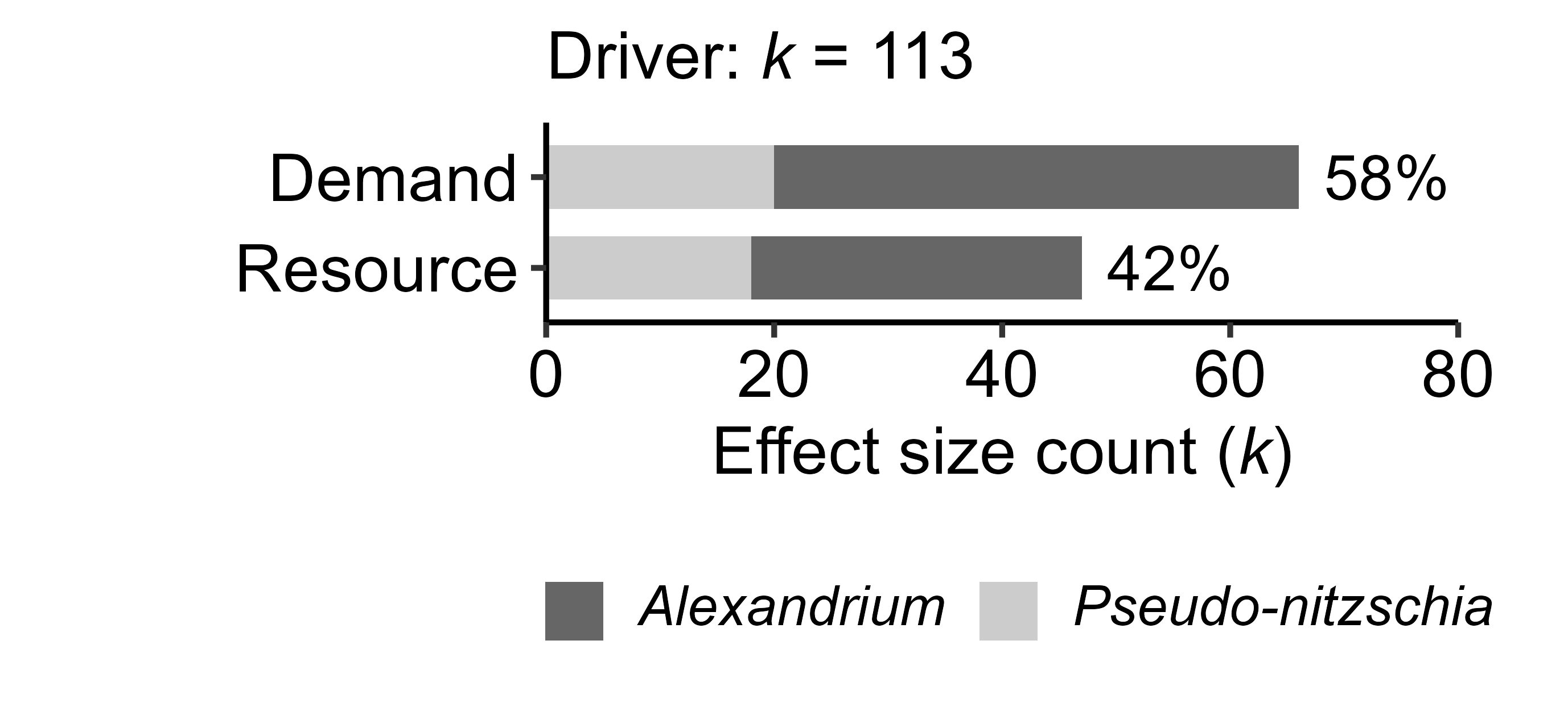


**Fig. S2.** Distribution of effect sizes (*k*) between levels of moderator driver (demand = elevated grazing risk; resource = relative nitrogen enrichment), coloured by phytoplankton genus (*Alexandrium* and *Pseudo-nitzschia*).


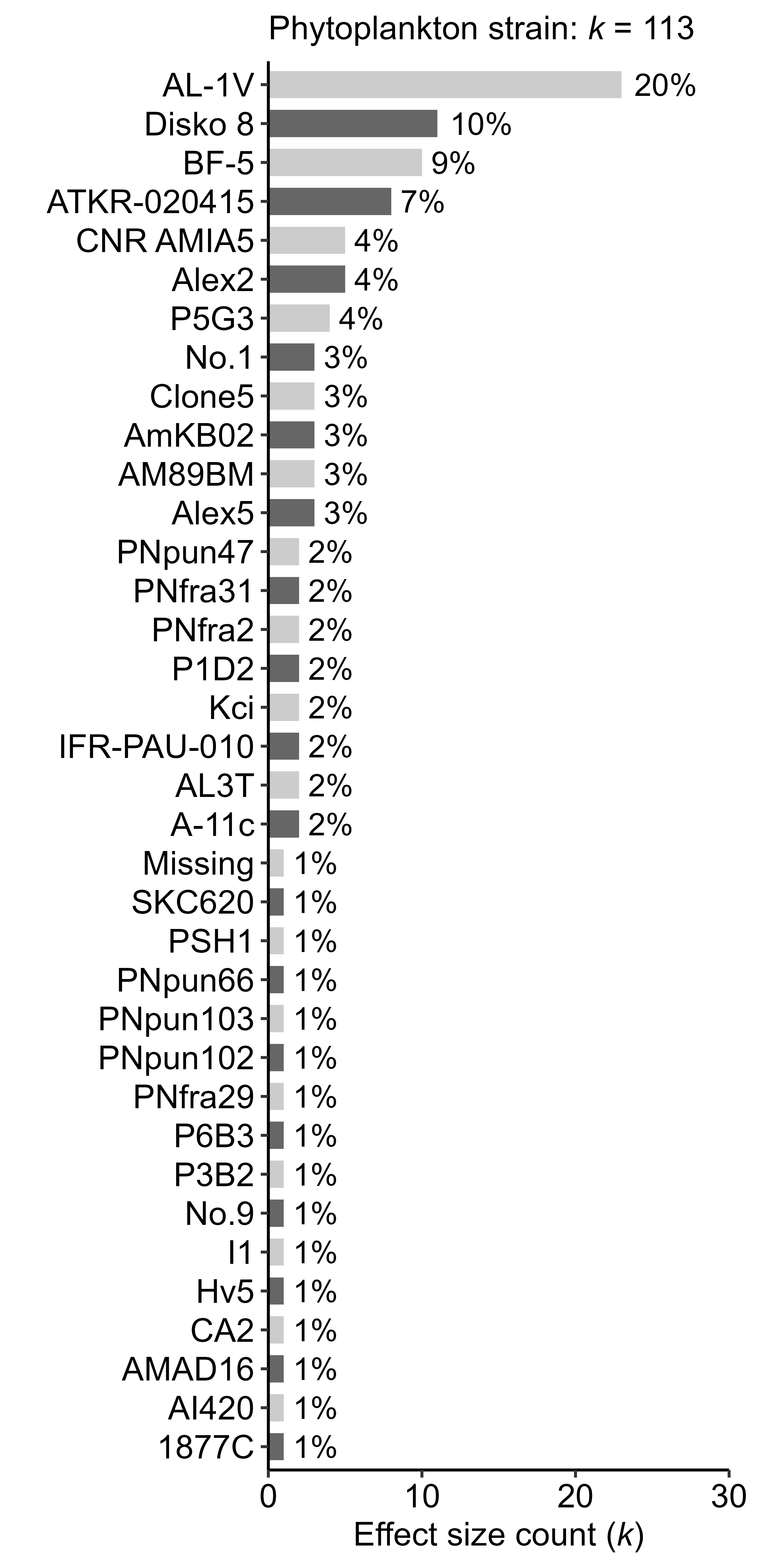


**Fig. S3.** Distribution of effect sizes (*k*) among phytoplankton strains.


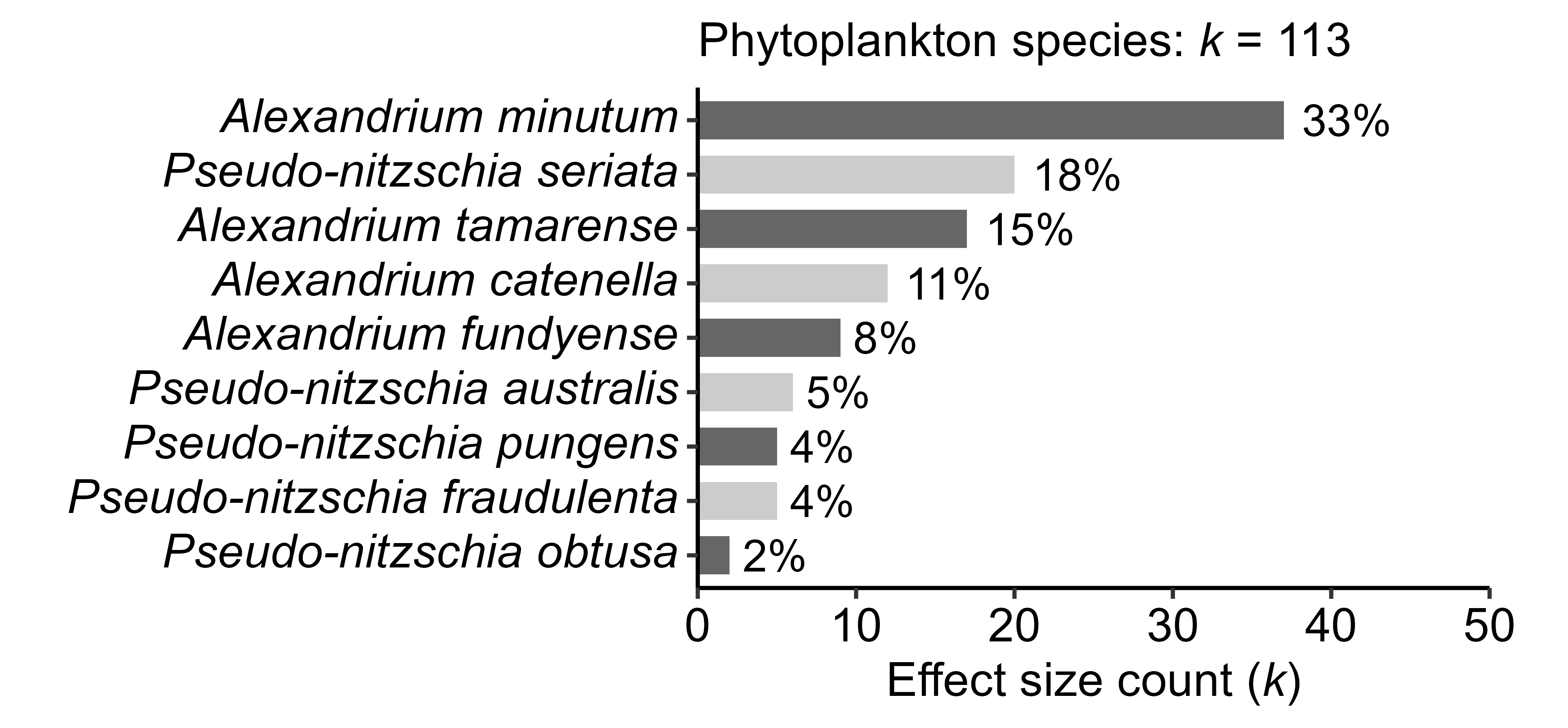


**Fig. S4.** Distribution of effect sizes (*k*) among phytoplankton species.


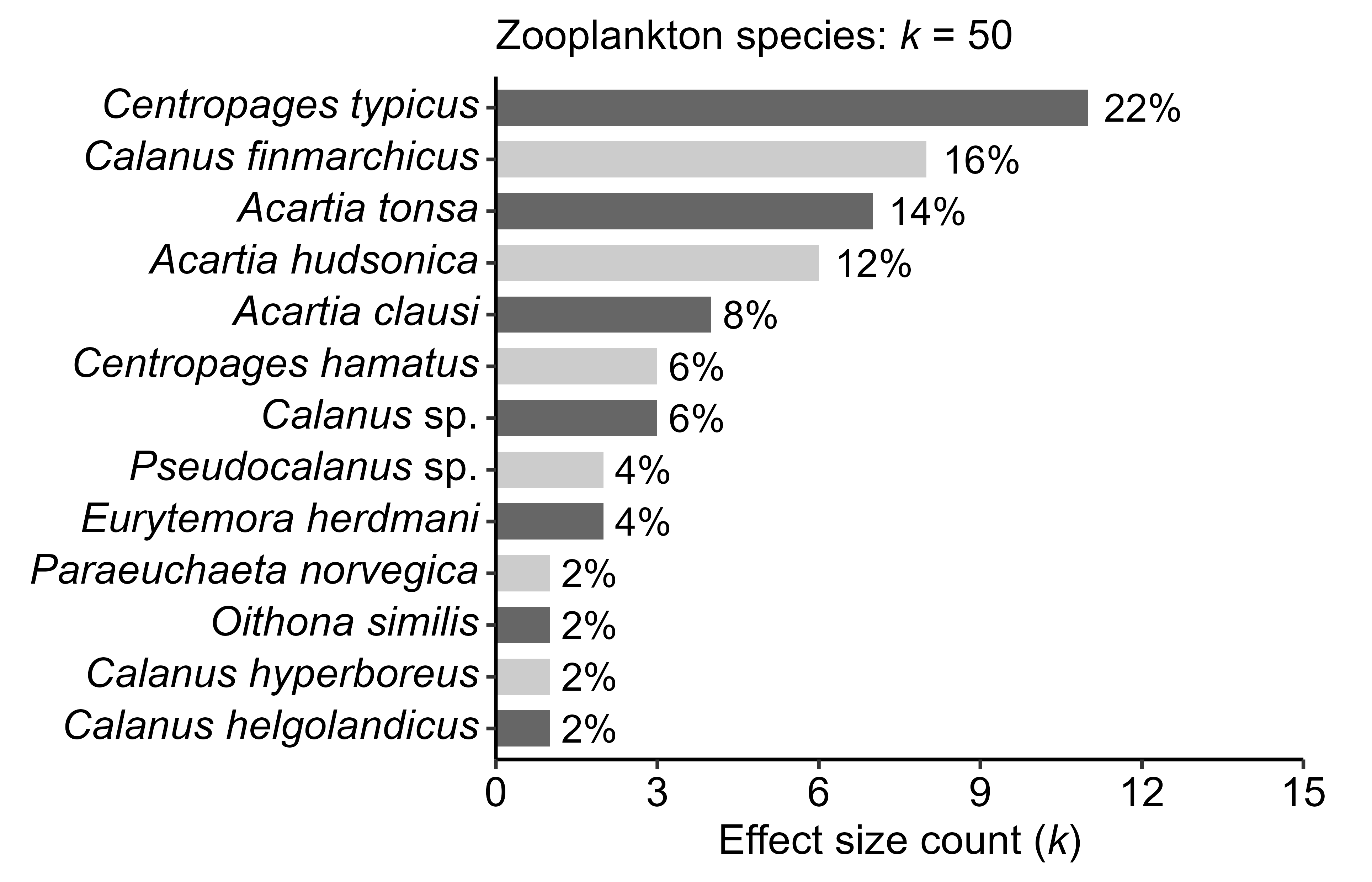


**Fig. S5.** Distribution of effect sizes (*k*) from studies that exposed phytoplankton to live zooplankton among zooplankton (copepod) species.


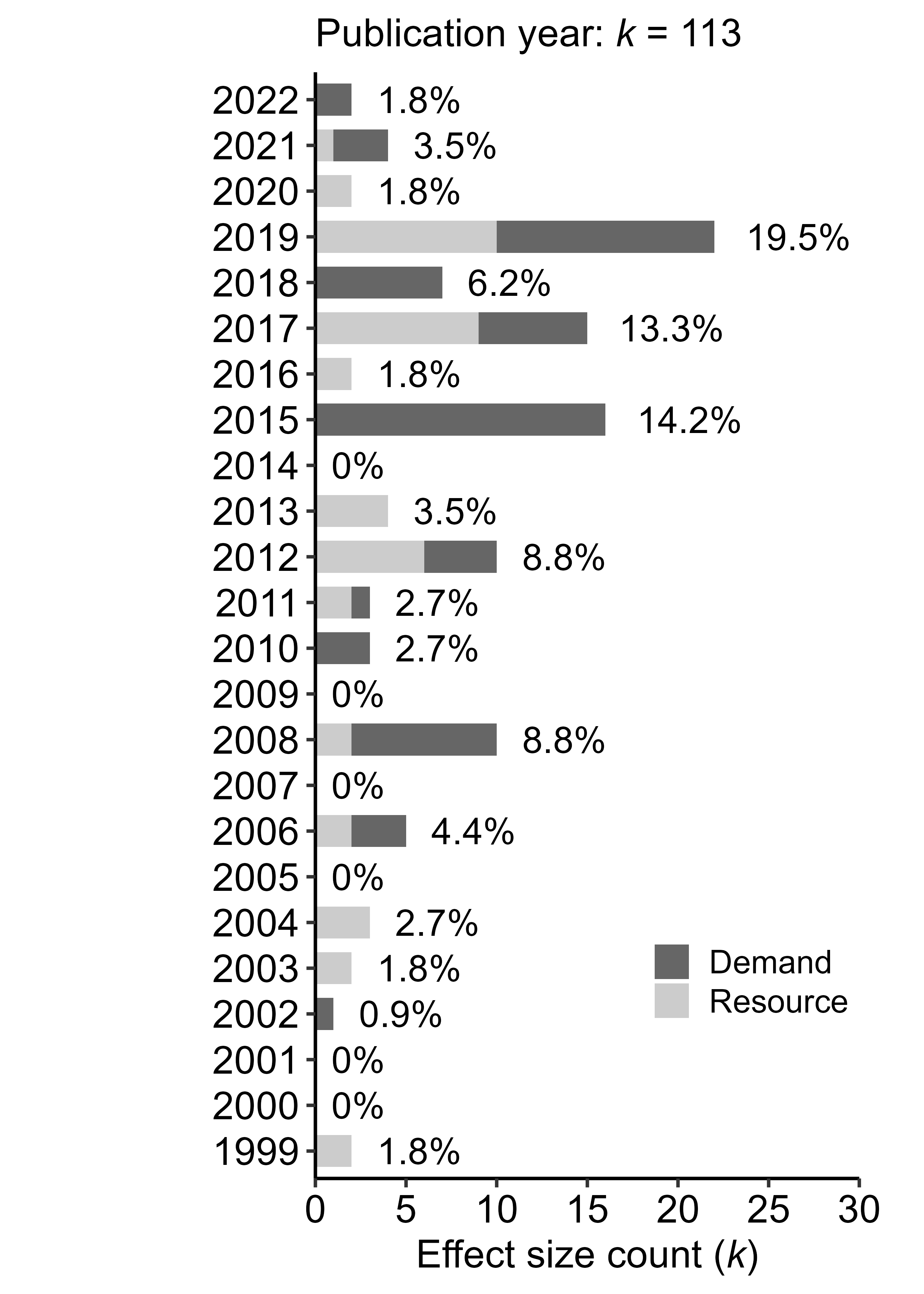


**Fig. S6.** Distribution of effect sizes (*k*) over publication year, coloured by experiment type/driver (demand = elevated grazing risk; resource = relative nitrogen enrichment).


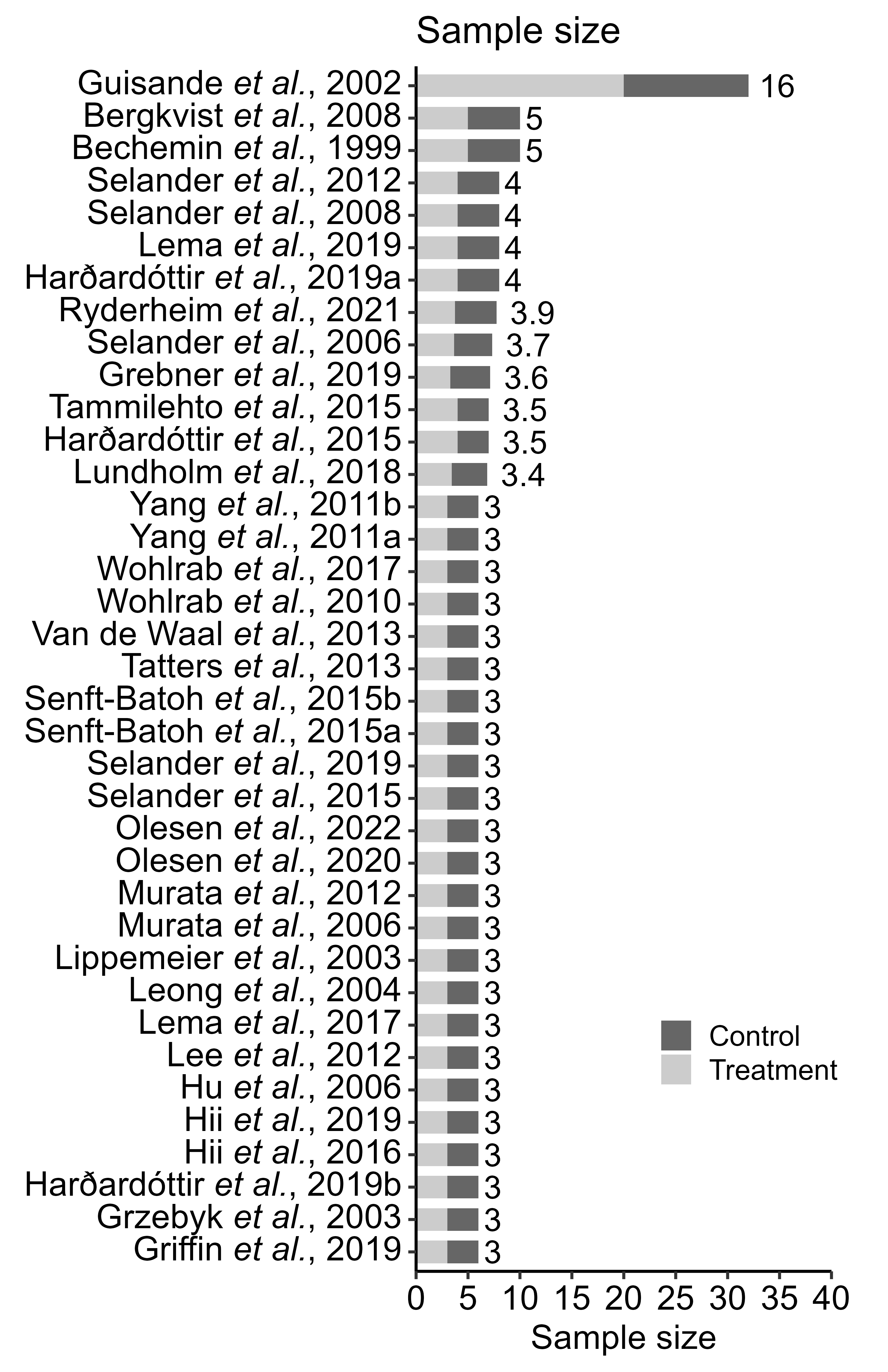


**Fig. S7.** Total sample size of studies included in the analysis, coloured by experimental group (control and treatment). For experimental contrasts between different nutrient levels (yielding different N:P ratios) of resource papers/effects, the treatment with the lower N:P ratio was consistently defined as the control. The numbers next to each bar indicate the average sample size for controls and treatments in each study.


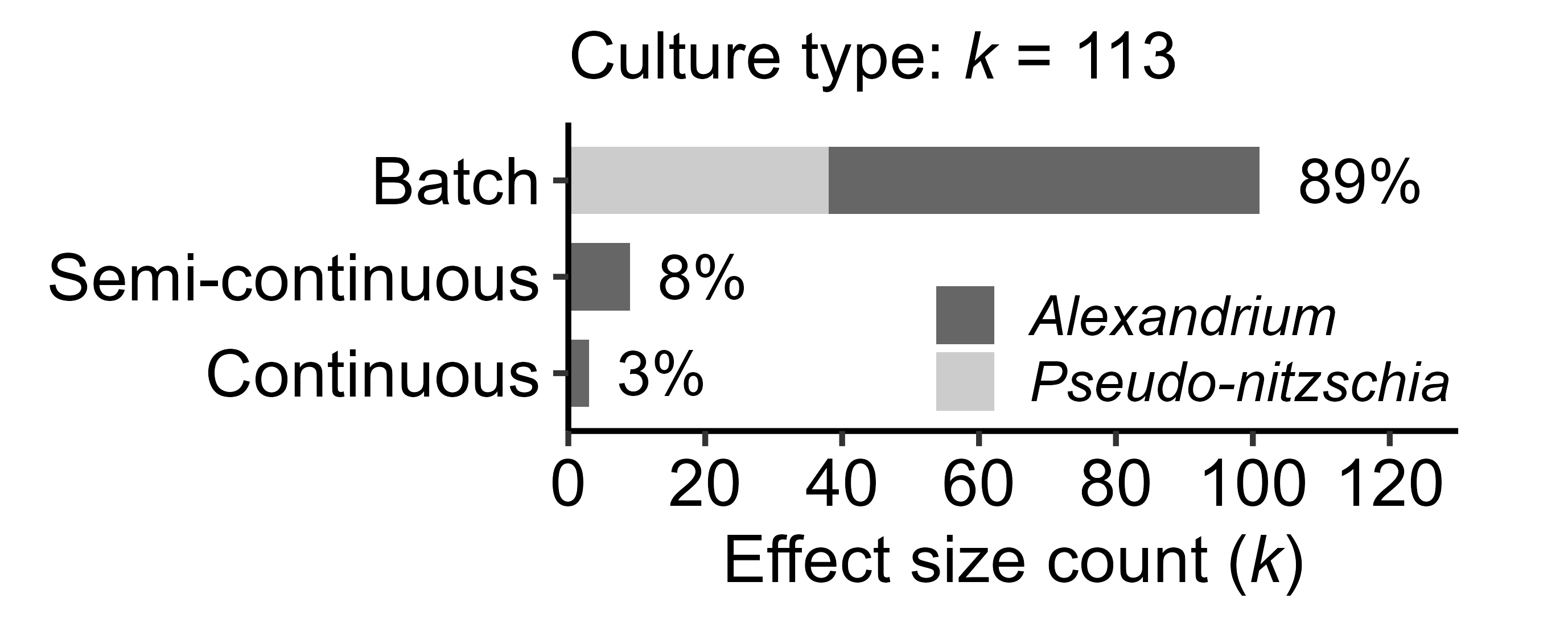


**Fig. S8.** Distribution of effect sizes (*k*) between levels of moderator culture type, partitioned by phytoplankton genus (*Alexandrium* and *Pseudo-nitzschia*).





**Fig. S9.** Stacked histogram of effect sizes (*k*) distributed over continuous moderators (A) light intensity, (B) light:dark cycle, (C) temperature, (D) salinity, (E) and duration of experiment. Bars are coloured by experiment type/driver (demand = elevated grazing risk; resource = relative nitrogen enrichment). Note that two cases were omitted from E as they ran for more than tenfold longer (5760 h) than the second longest experiments (480 h).


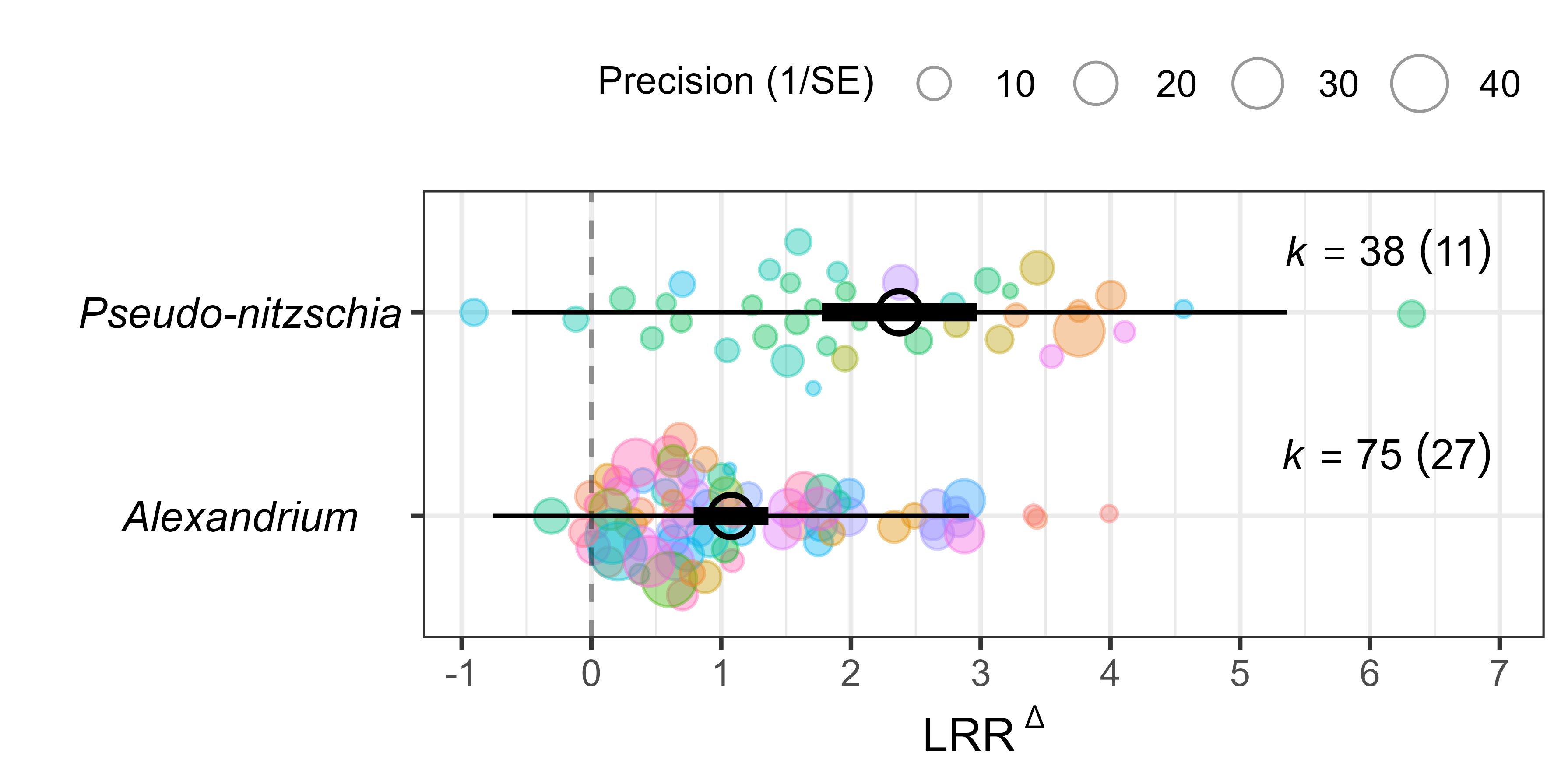

**Fig. S10.** Pooled effects of relative nitrogen enrichment (resource) and elevated grazing risk (demand) on phycotoxin induction (LRR^Δ^), shown separately for each genus. Data from both drivers were combined to calculate an overall mean effect size for each genus. LRR^Δ^ is the small sample bias-corrected log response ratio proposed by Lajeunesse (2015). Empty black circles represent mean effect sizes, thick black lines denote 95% confidence intervals (95% CI) for the mean effects, and thin black lines indicate 95% prediction intervals (95% PI; where 95% of new effect sizes are expected to fall with repeated sampling of the literature). Coloured circles denote individual effect sizes (*k*) from a given number of studies (*N*), sized inversely proportional to their sampling error (1/SE), and coloured by their study ID.


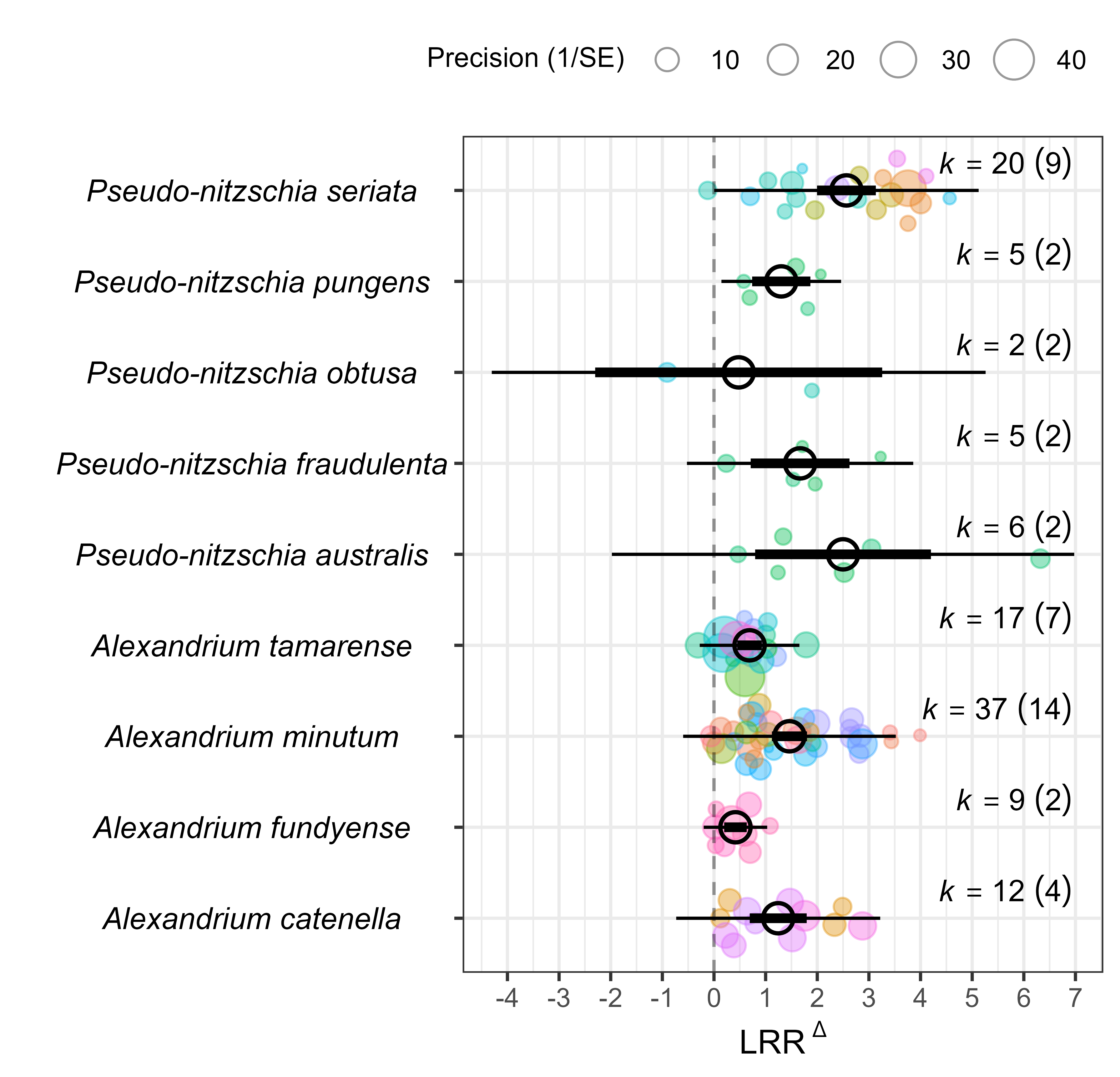

**Fig. S11.** Effects of relative nitrogen enrichment (resource) or elevated grazing risk (demand) on phycotoxin induction (LRR^Δ^), separated by phytoplankton species within genera *Alexandrium* and *Pseudo-nitzschia*. LRR^Δ^ is the small sample bias-corrected log response ratio proposed by Lajeunesse (2015). Empty black circles represent mean effect sizes, thick black lines denote 95% confidence intervals (95% CI) for the mean effects, and thin black lines indicate 95% prediction intervals (95% PI; where 95% of new effect sizes are expected to fall with repeated sampling of the literature). Coloured circles denote individual effect sizes (*k*) from a given number of studies (*N*), sized inversely proportional to their sampling error (1/SE), and coloured by their study ID.


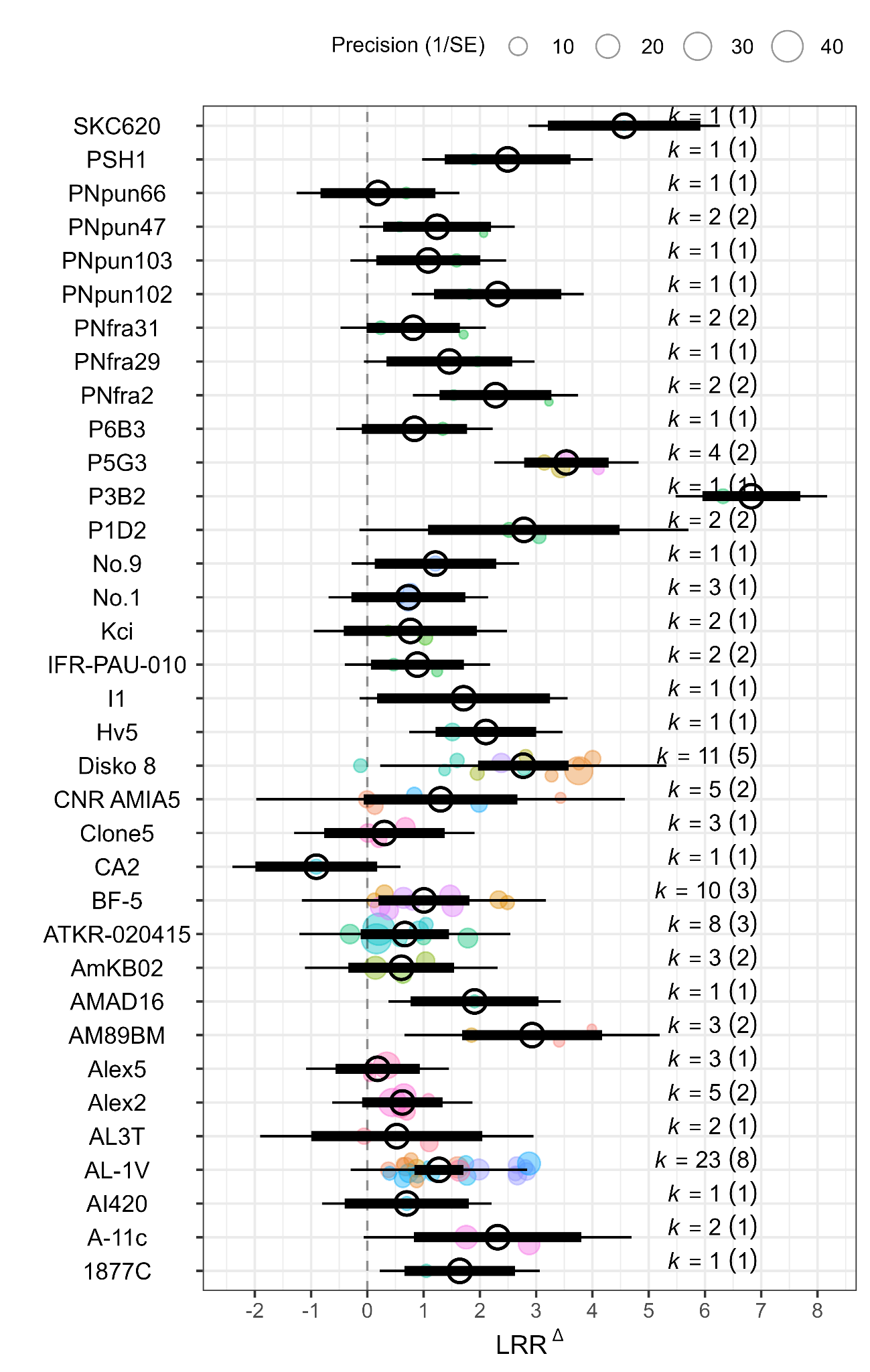

**Fig. S12.** Effects of relative nitrogen enrichment (resource) or elevated grazing risk (demand) on phycotoxin induction (LRR^Δ^), partitioned among phytoplankton strains. LRR^Δ^ is the small sample bias-corrected log response ratio proposed by Lajeunesse (2015). Empty black circles represent mean effect sizes, thick black lines denote 95% confidence intervals (95% CI) for the mean effects, and thin black lines indicate 95% prediction intervals (95% PI; where 95% of new effect sizes are expected to fall with repeated sampling of the literature). Coloured circles denote individual effect sizes (*k*) from a given number of studies (*N*), sized inversely proportional to their sampling error (1/SE), and coloured by their study ID.


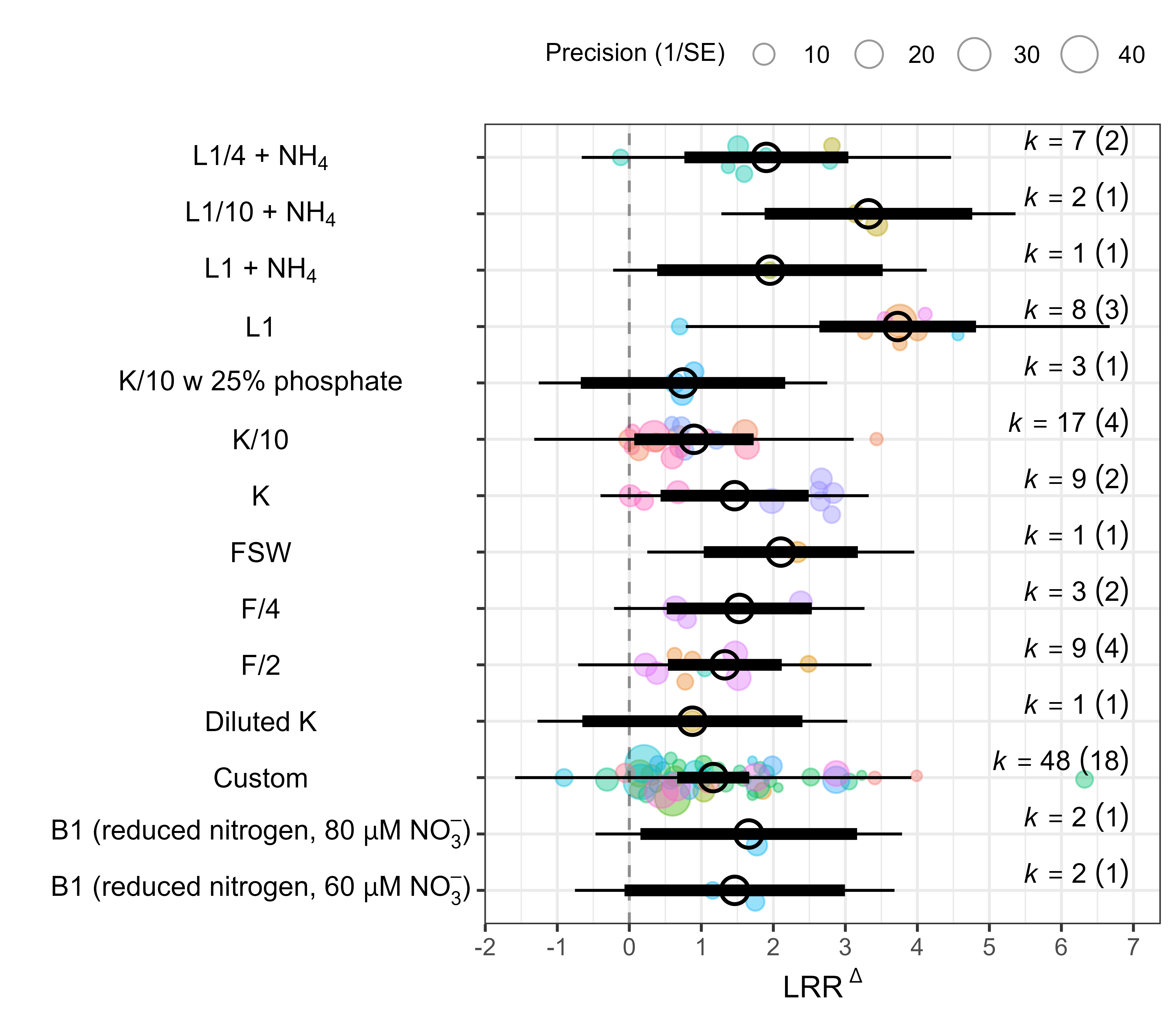

**Fig. S13.** Effects of relative nitrogen enrichment (resource) or elevated grazing risk (demand) on phycotoxin induction (LRR^Δ^), separated by culture medium used. LRR^Δ^ is the small sample bias-corrected log response ratio proposed by Lajeunesse (2015). Empty black circles represent mean effect sizes, thick black lines denote 95% confidence intervals (95% CI) for the mean effects, and thin black lines indicate 95% prediction intervals (95% PI; where 95% of new effect sizes are expected to fall with repeated sampling of the literature). Coloured circles denote individual effect sizes (*k*) from a given number of studies (*N*), sized inversely proportional to their sampling error (1/SE), and coloured by their study ID.


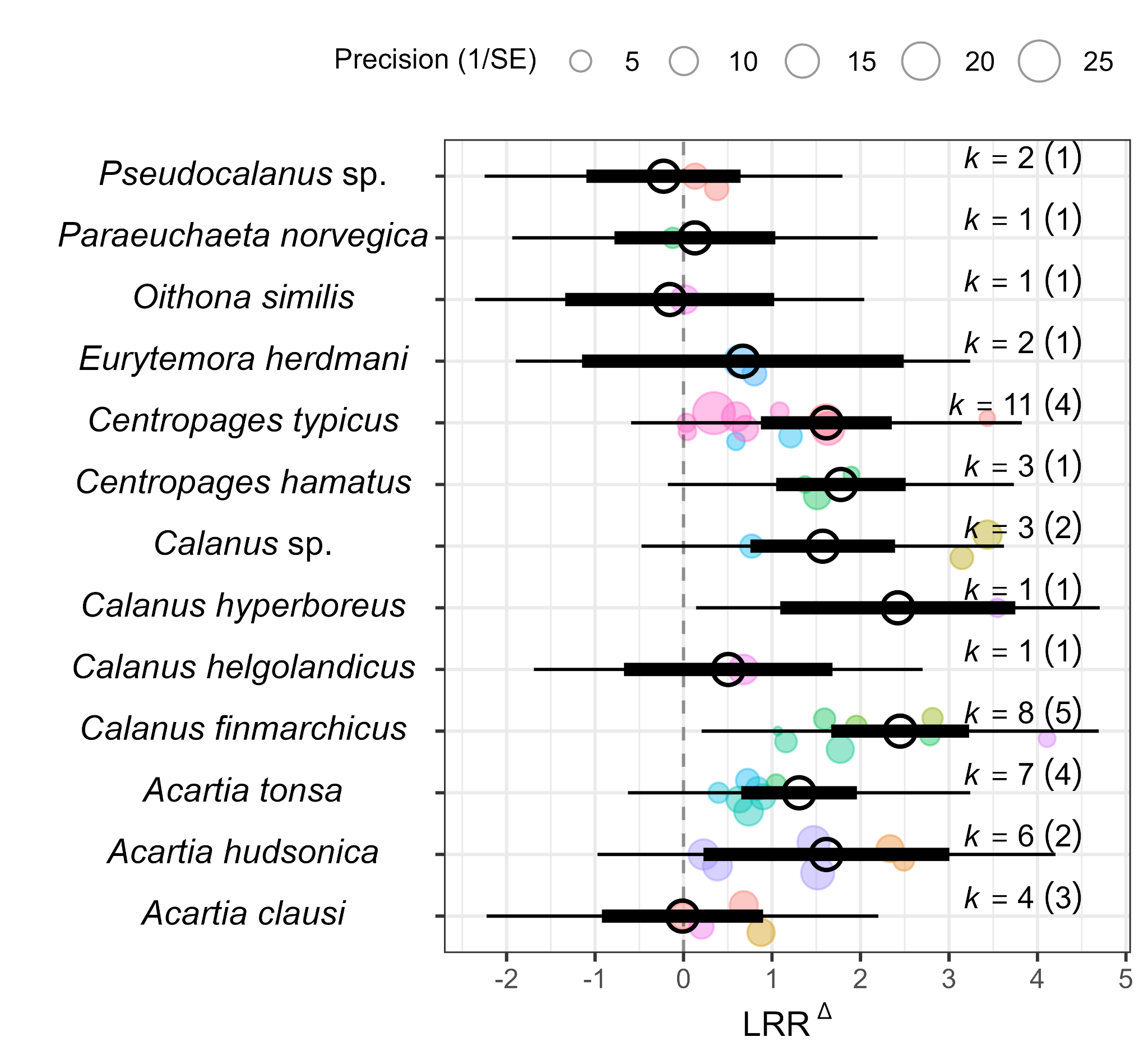

**Fig. S14.** Effects of elevated grazing risk (demand) on phycotoxin induction (LRR^Δ^), separated by the copepod species used. LRR^Δ^ is the small sample bias-corrected log response ratio proposed by Lajeunesse (2015). Empty black circles represent mean effect sizes, thick black lines denote 95% confidence intervals (95% CI) for the mean effects, and thin black lines indicate 95% prediction intervals (95% PI; where 95% of new effect sizes are expected to fall with repeated sampling of the literature). Coloured circles denote individual effect sizes (*k*) from a given number of studies (*N*), sized inversely proportional to their sampling error (1/SE), and coloured by their study ID.




**Fig. S15.** Effects of relative nitrogen enrichment (resource) or elevated grazing risk (demand) on phycotoxin induction (LRR^Δ^), as a function of (A) experimental light intensity (B) and temperature. LRR^Δ^ is the small sample bias-corrected log response ratio proposed by Lajeunesse (2015). The solid black line is the fitted linear relationship from meta-regression, thick dashed lines denote 95% confidence interval (95% CI) for the fit, and the thin dotted lines indicate 95% prediction interval (95% PI; where 95% of new effect sizes are expected to fall with repeated sampling of the literature). Circles denote individual effect sizes (*k*) from a given number of studies (*N*), sized inversely proportional to their sampling error (1/SE).
